# Supplementary figures and images for: In silico screening by AlphaFold2 program revealed the potential binding partners of nuage-localizing proteins and piRNA-related proteins (part 1 of 2)
Source: eLife. 2025 Apr 22;13:RP101967. doi: 10.7554/eLife.101967 (PMC12014135; doi:10.7554/eLife.101967)

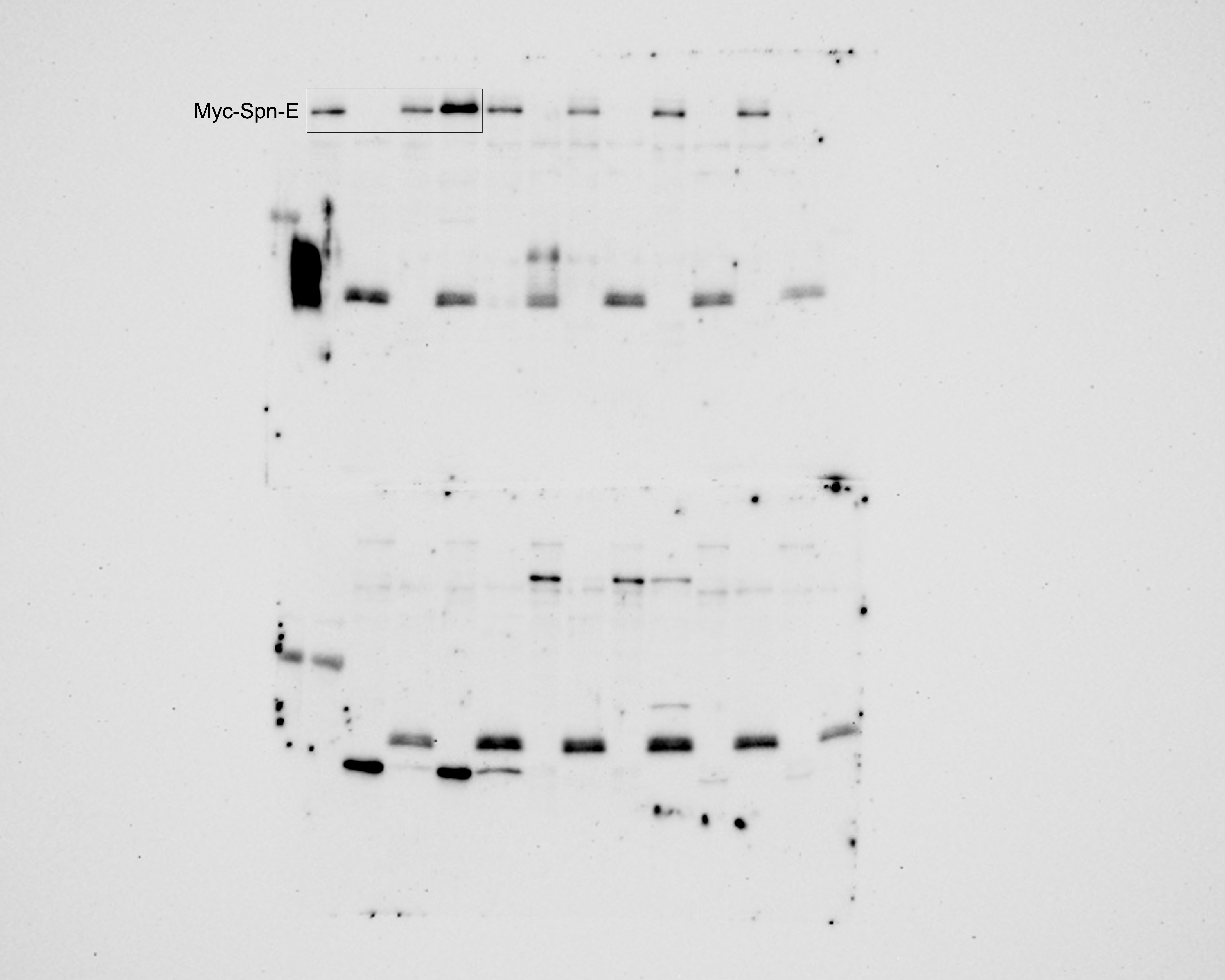

Supplement: Figure 1—source data 2. [file elife-101967-fig1-data2.zip › Figure 1-Source Data 2/Fig1D-i_rep2_Myc_label_2023-08-17.tiff]

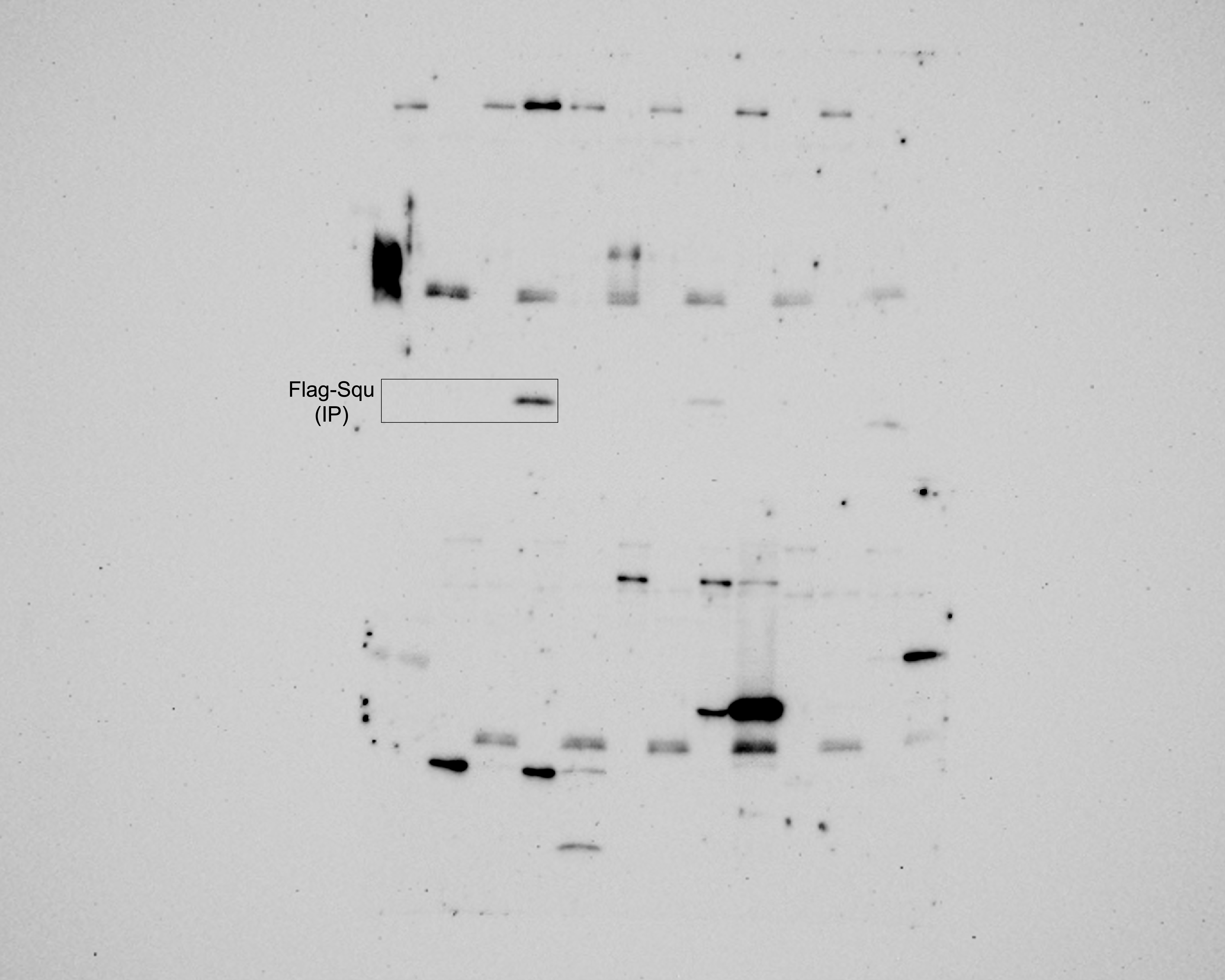

Supplement: Figure 1—source data 2. [file elife-101967-fig1-data2.zip › Figure 1-Source Data 2/Fig1D-i_rep2_FLAG_label_2023-08-17.tiff]

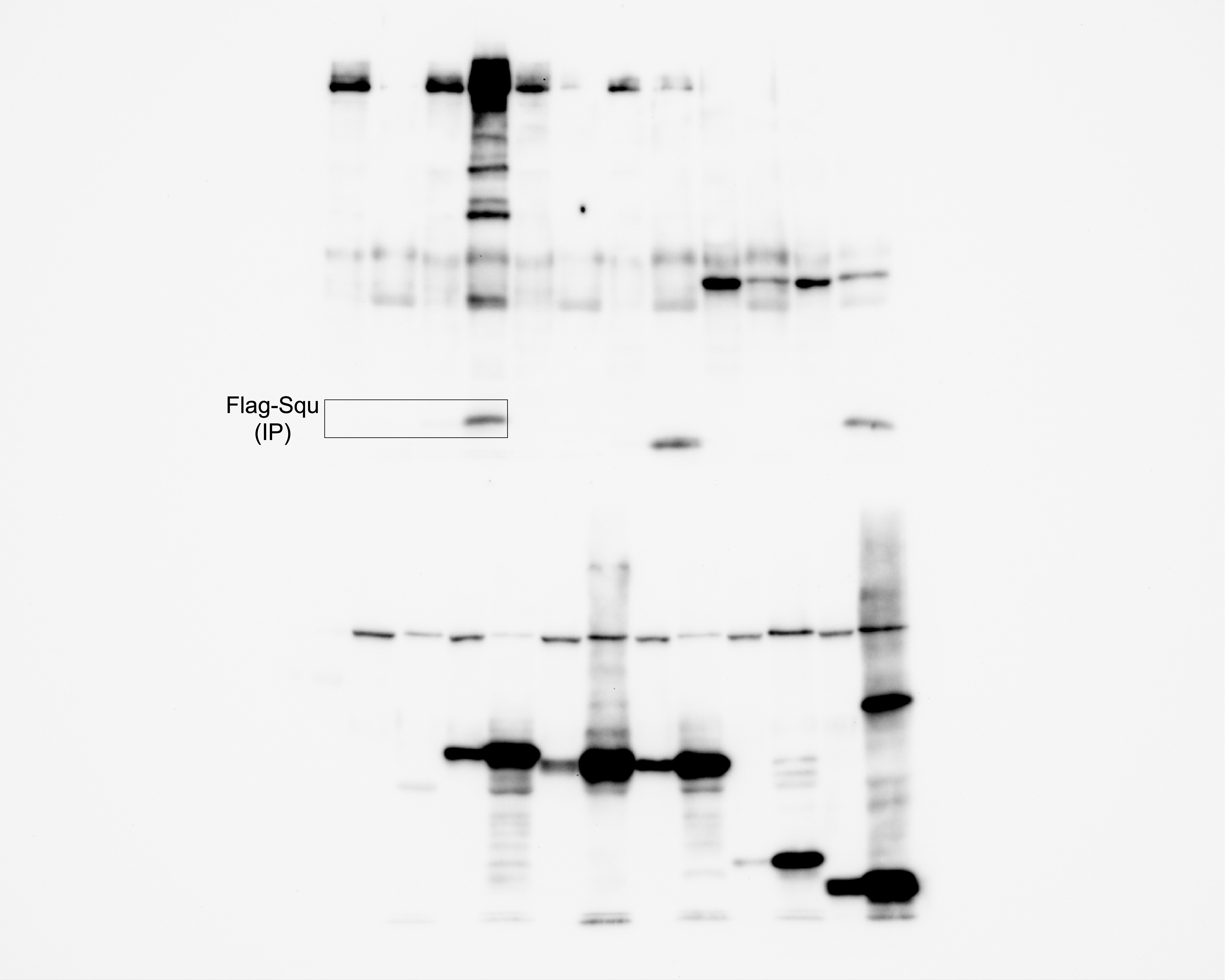

Supplement: Figure 1—source data 2. [file elife-101967-fig1-data2.zip › Figure 1-Source Data 2/Fig1D-i_rep3_FLAG_label_2023-09-27.tiff]

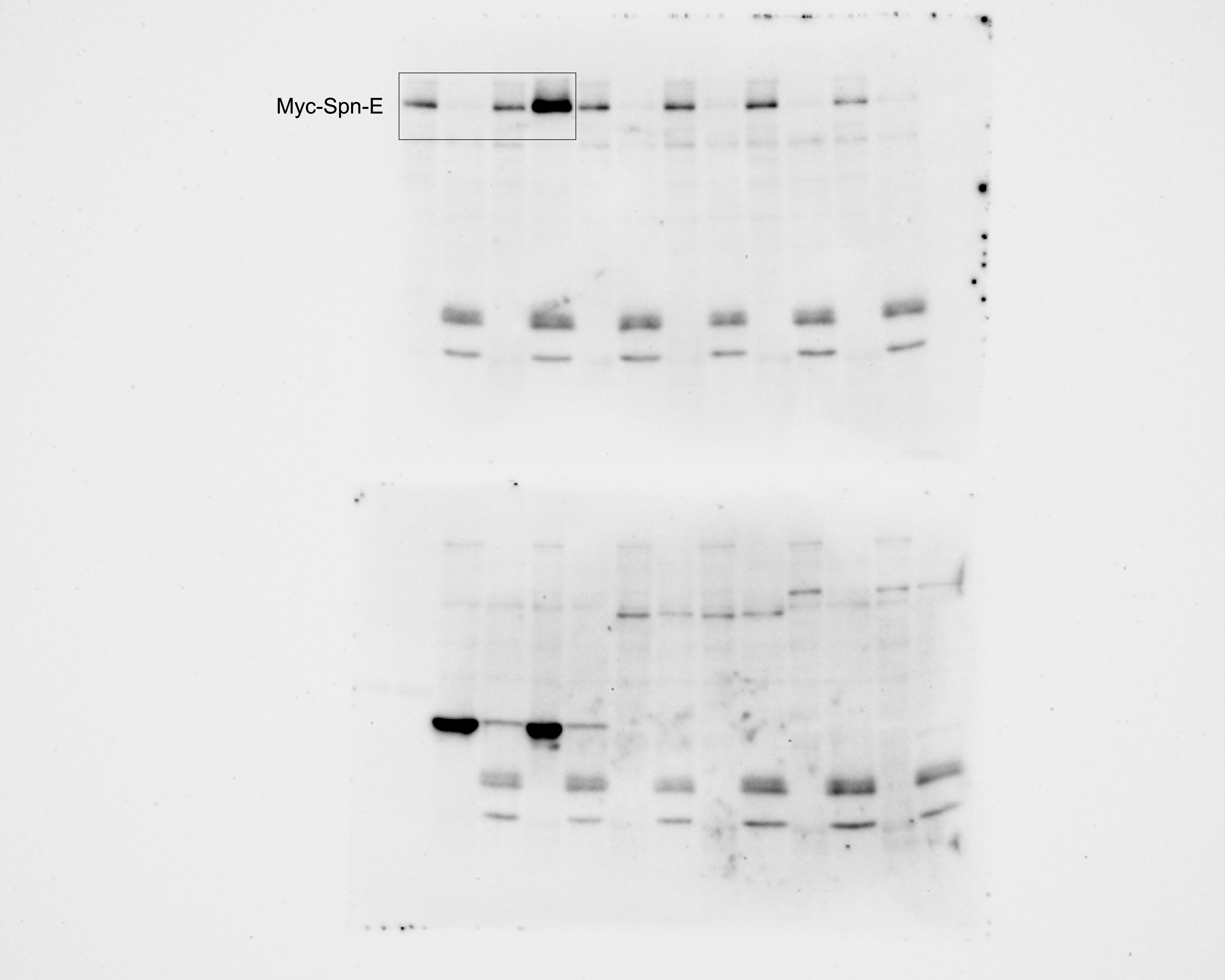

Supplement: Figure 1—source data 2. [file elife-101967-fig1-data2.zip › Figure 1-Source Data 2/Fig1D-i_rep1_Myc_label_2023-08-01.tiff]

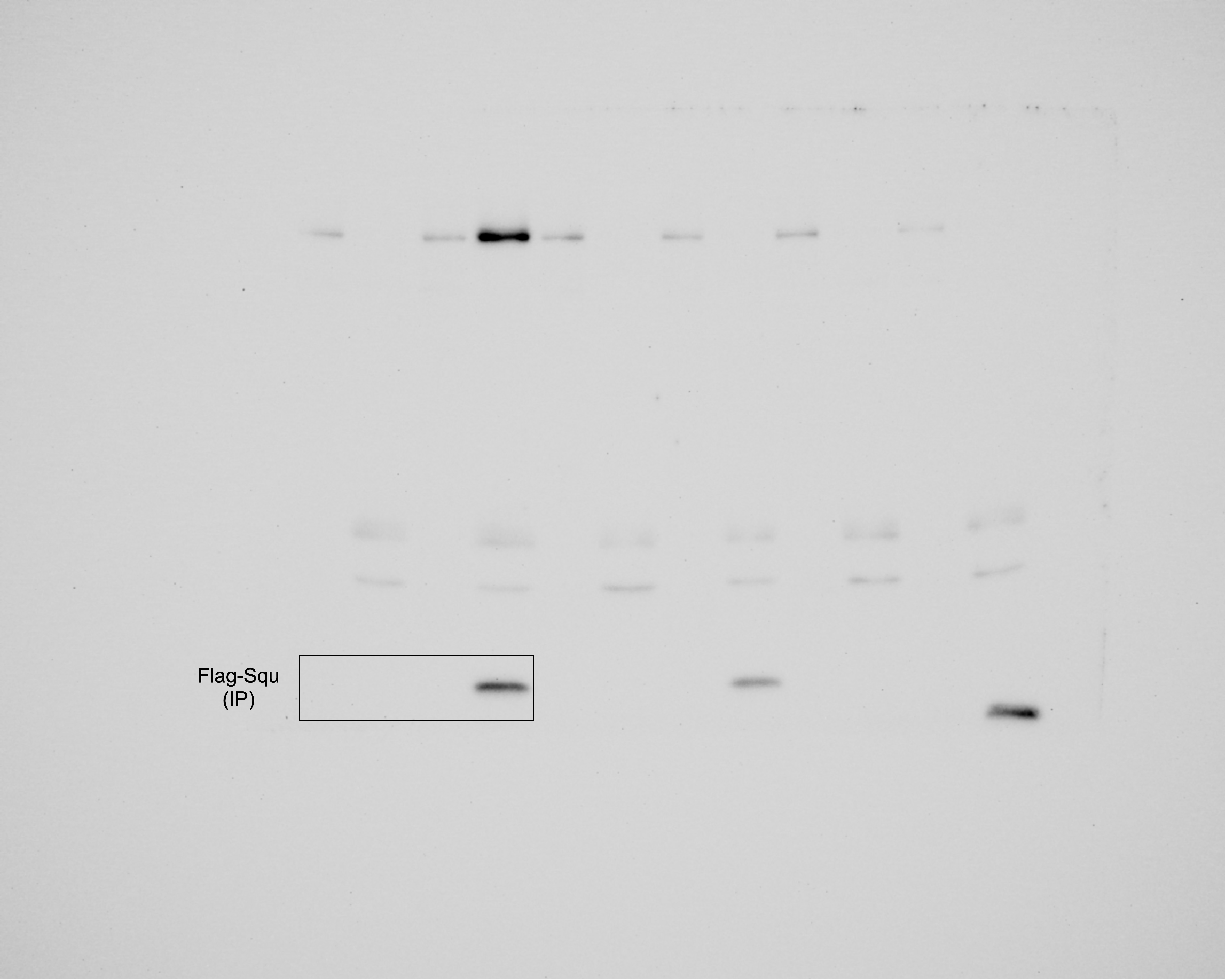

Supplement: Figure 1—source data 2. [file elife-101967-fig1-data2.zip › Figure 1-Source Data 2/Fig1D-i_rep1_FLAG_label_2023-08-02.tiff]

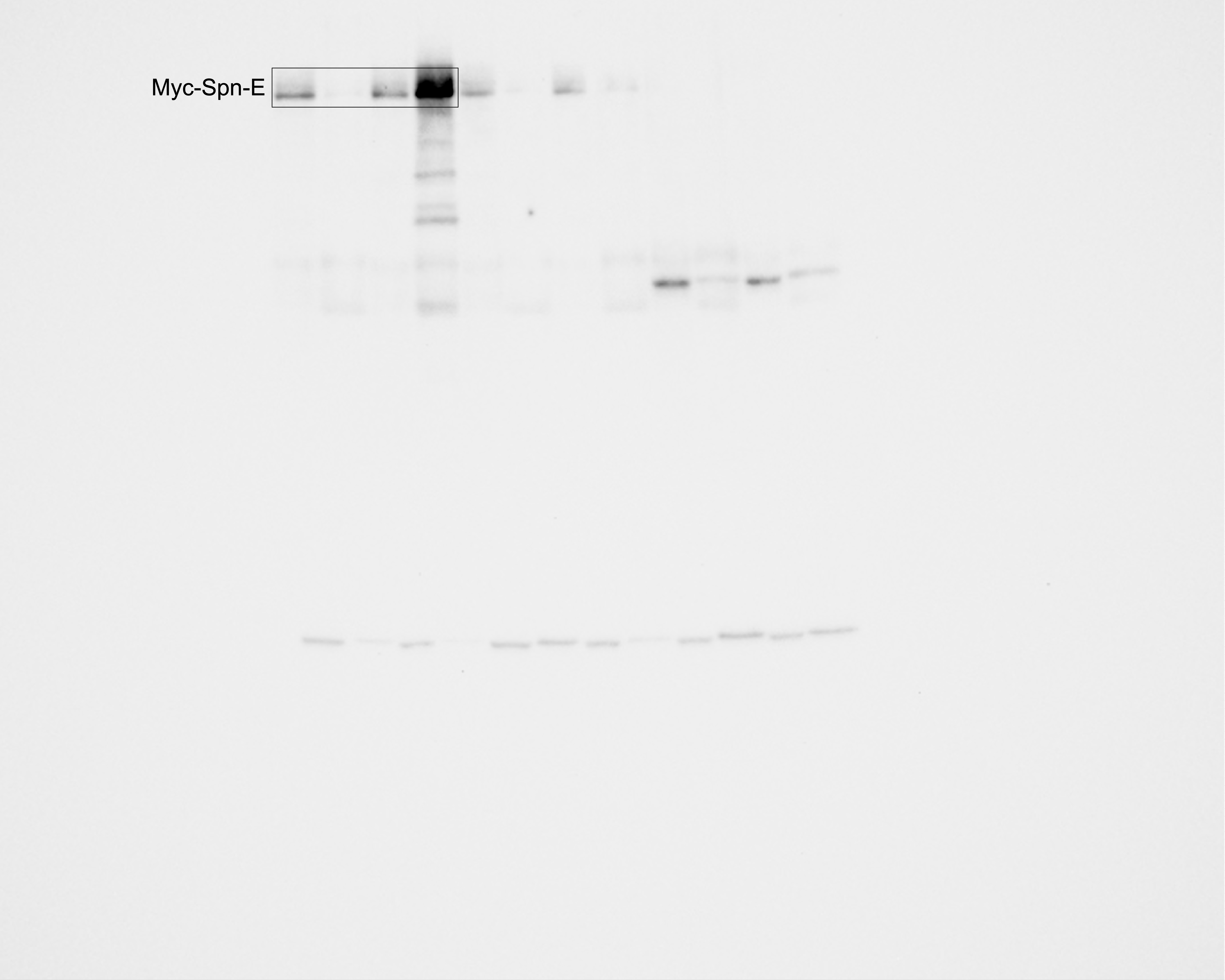

Supplement: Figure 1—source data 2. [file elife-101967-fig1-data2.zip › Figure 1-Source Data 2/Fig1D-i_rep3_Myc_label_2023-09-27.tiff]

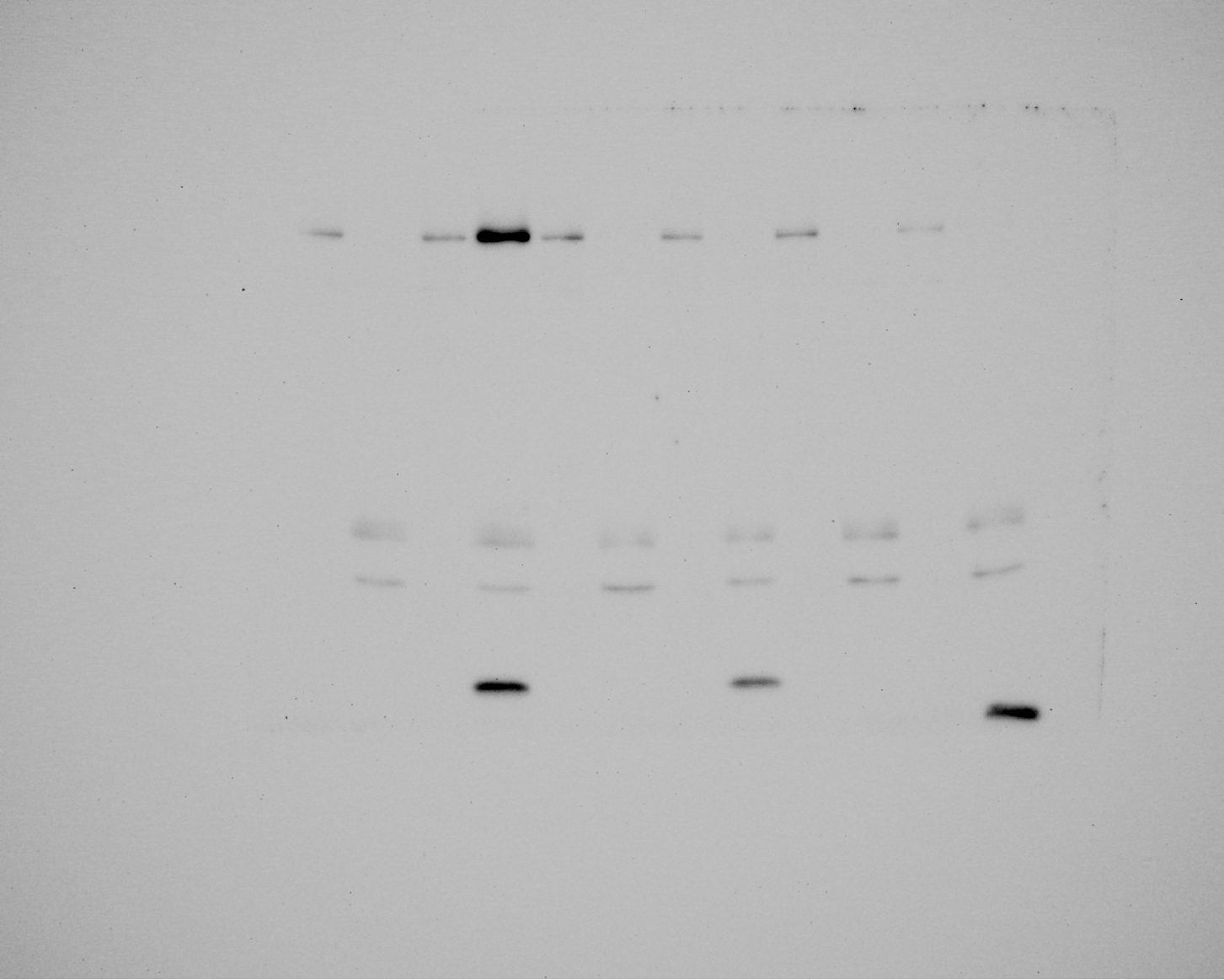

Supplement: Figure 1—source data 3. [file elife-101967-fig1-data3.zip › Figure 1-Source Data 3/Fig1D-i_rep1_FLAG_original_2023-08-02_300dpi.tif]

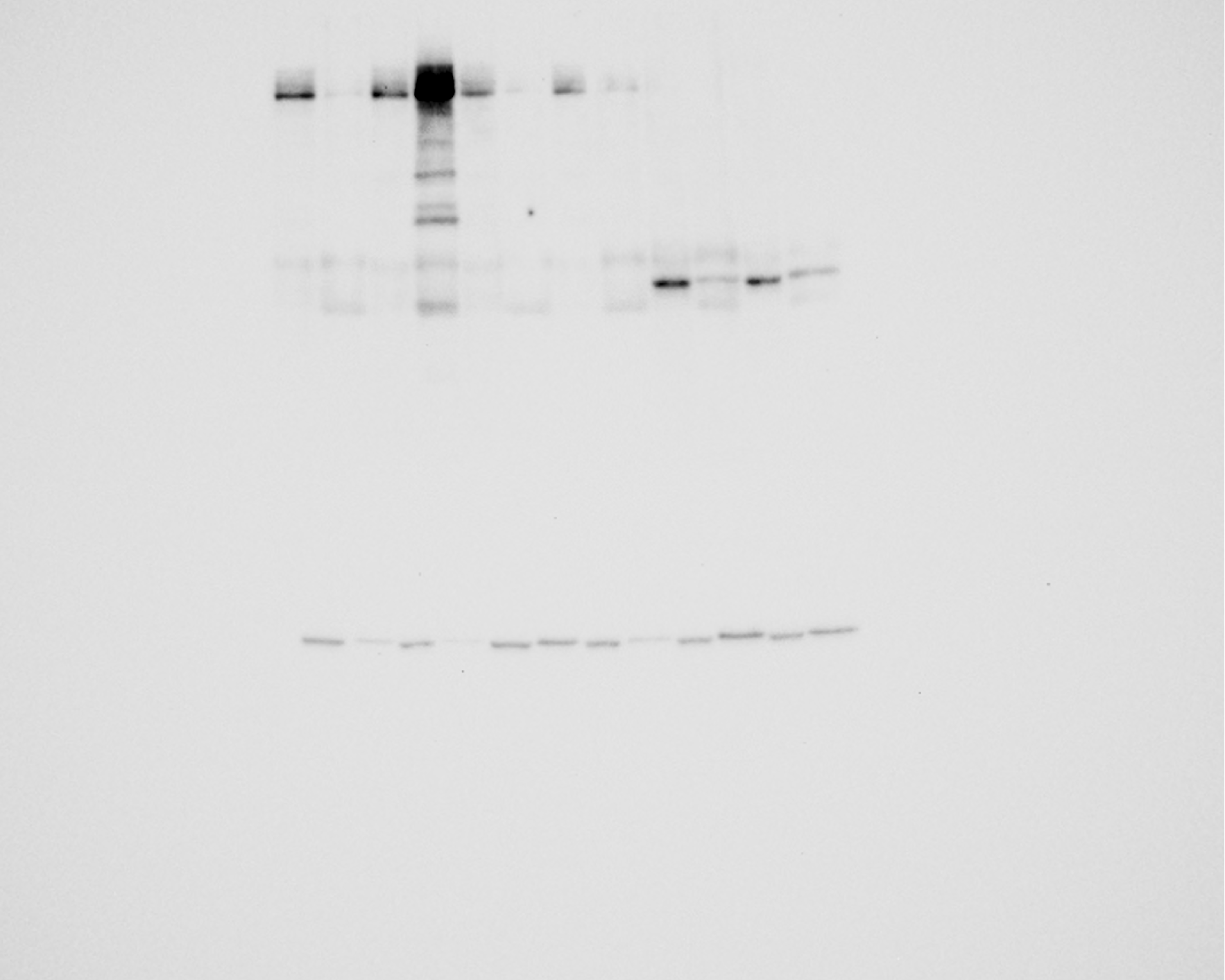

Supplement: Figure 1—source data 3. [file elife-101967-fig1-data3.zip › Figure 1-Source Data 3/Fig1D-i_rep3_Myc_original_2023-09-27.tif]

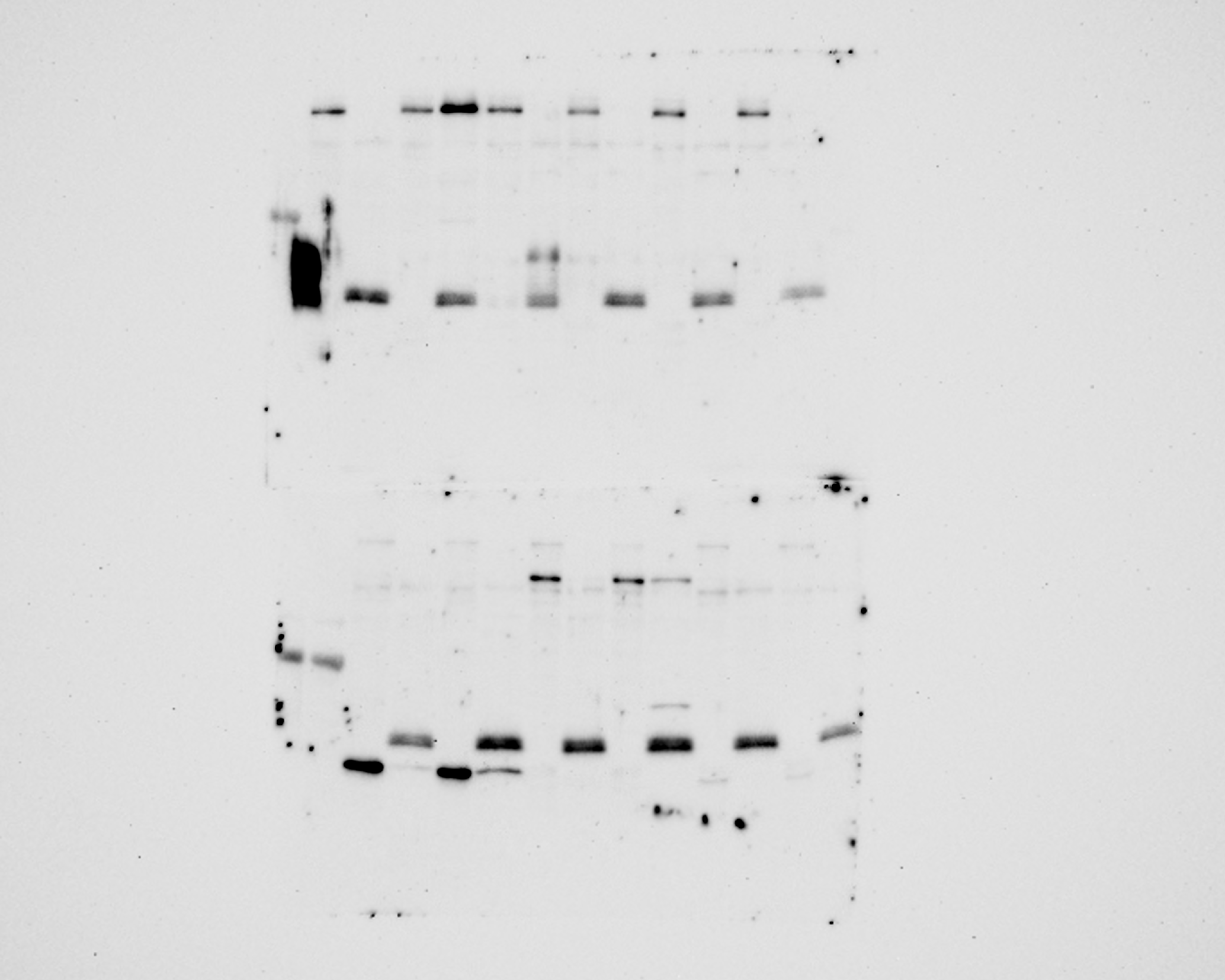

Supplement: Figure 1—source data 3. [file elife-101967-fig1-data3.zip › Figure 1-Source Data 3/Fig1D-i_rep2_Myc_original_2023-08-17.tif]

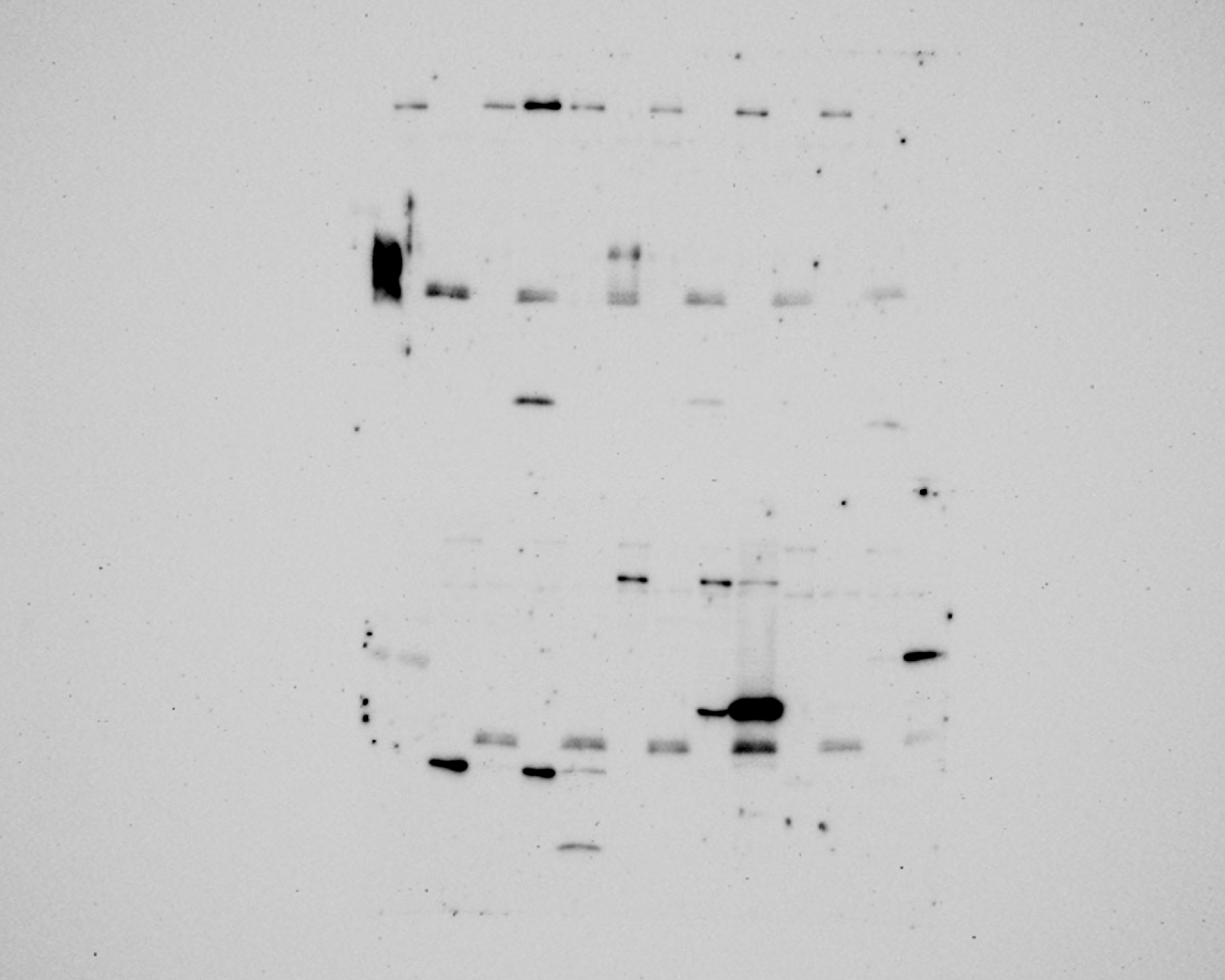

Supplement: Figure 1—source data 3. [file elife-101967-fig1-data3.zip › Figure 1-Source Data 3/Fig1D-i_rep2_FLAG_original_2023-08-17.tif]

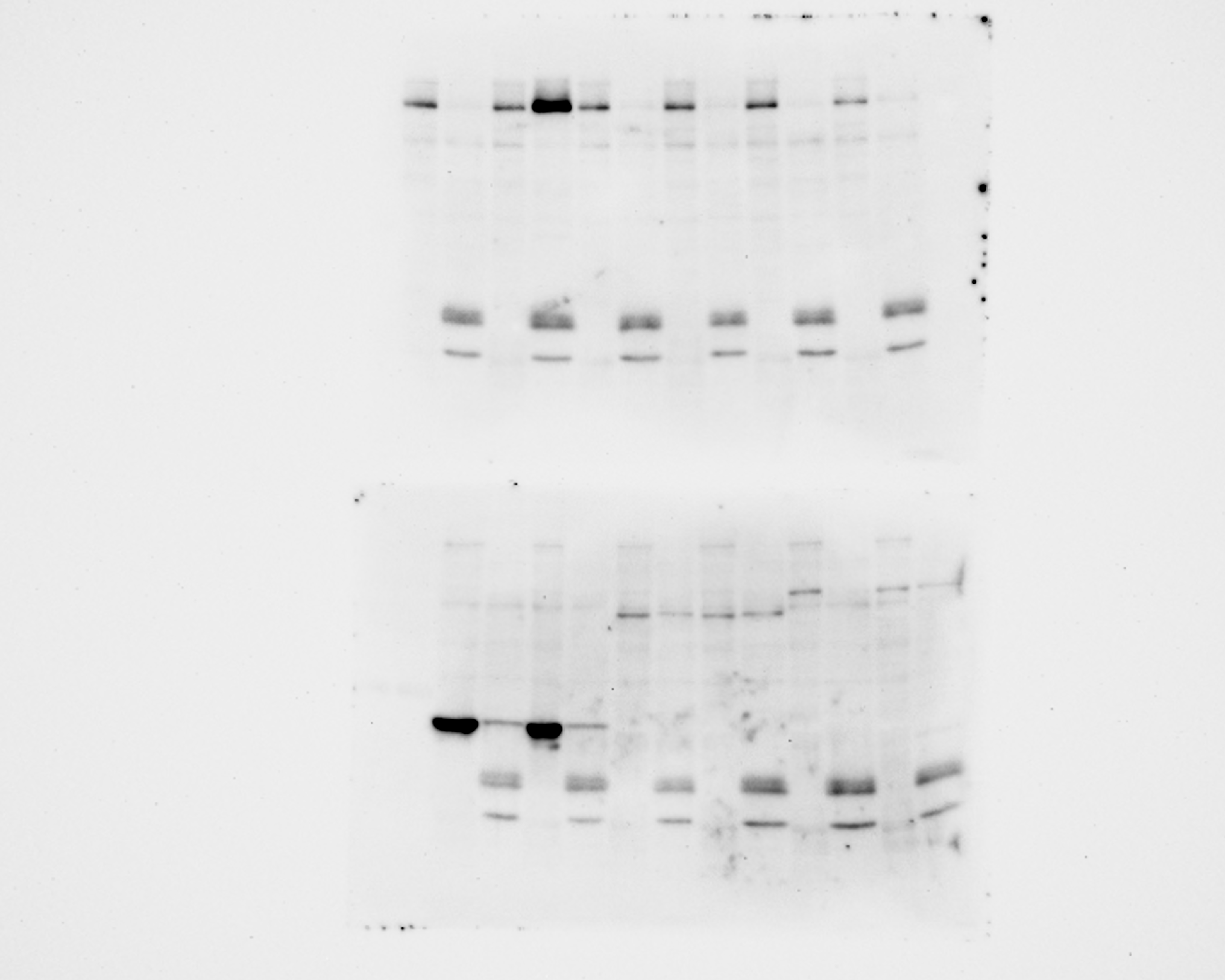

Supplement: Figure 1—source data 3. [file elife-101967-fig1-data3.zip › Figure 1-Source Data 3/Fig1D-i_rep1_Myc_original_2023-08-01_300dpi.tif]

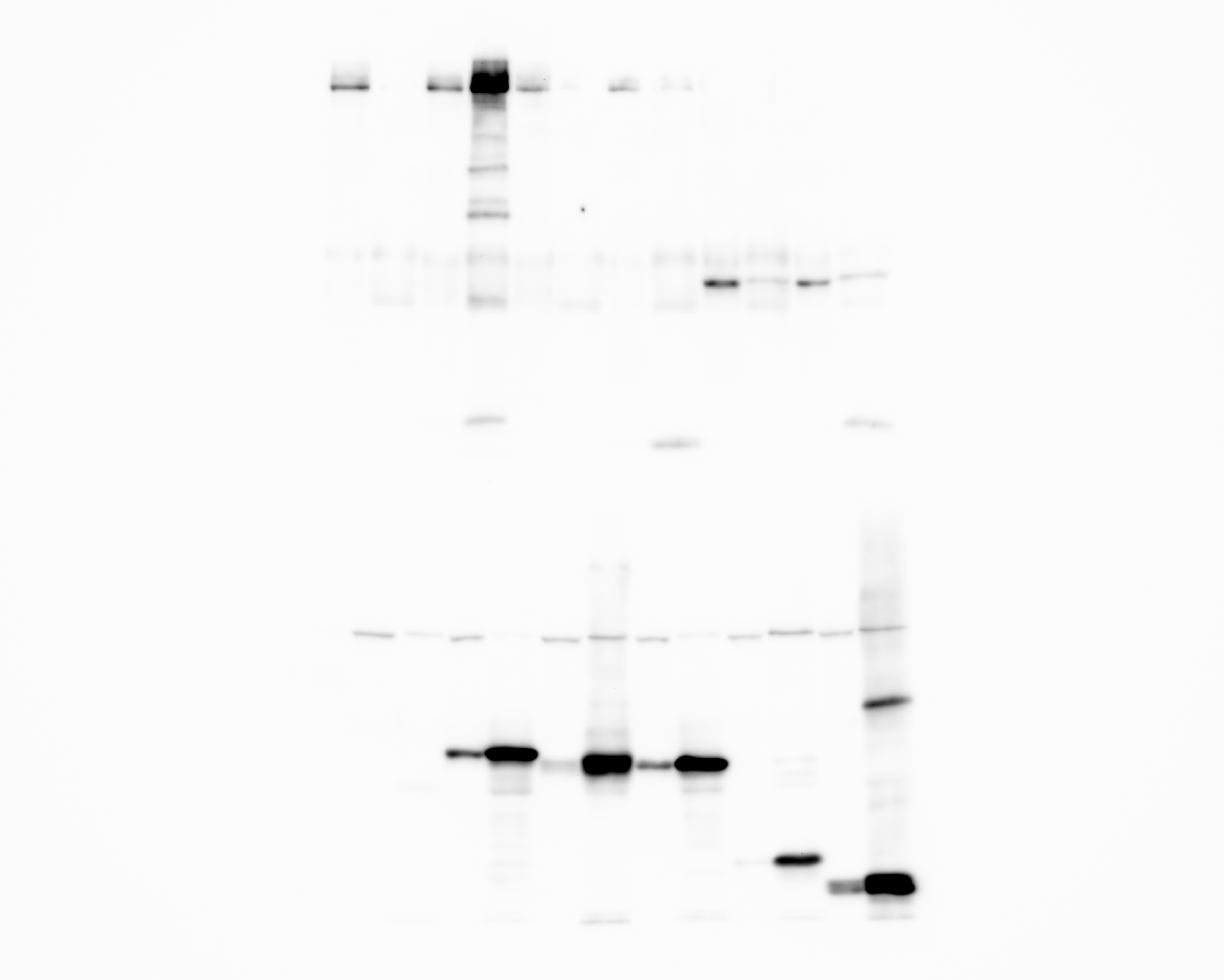

Supplement: Figure 1—source data 3. [file elife-101967-fig1-data3.zip › Figure 1-Source Data 3/Fig1D-i_rep3_FLAG_original_2023-09-27.tif]

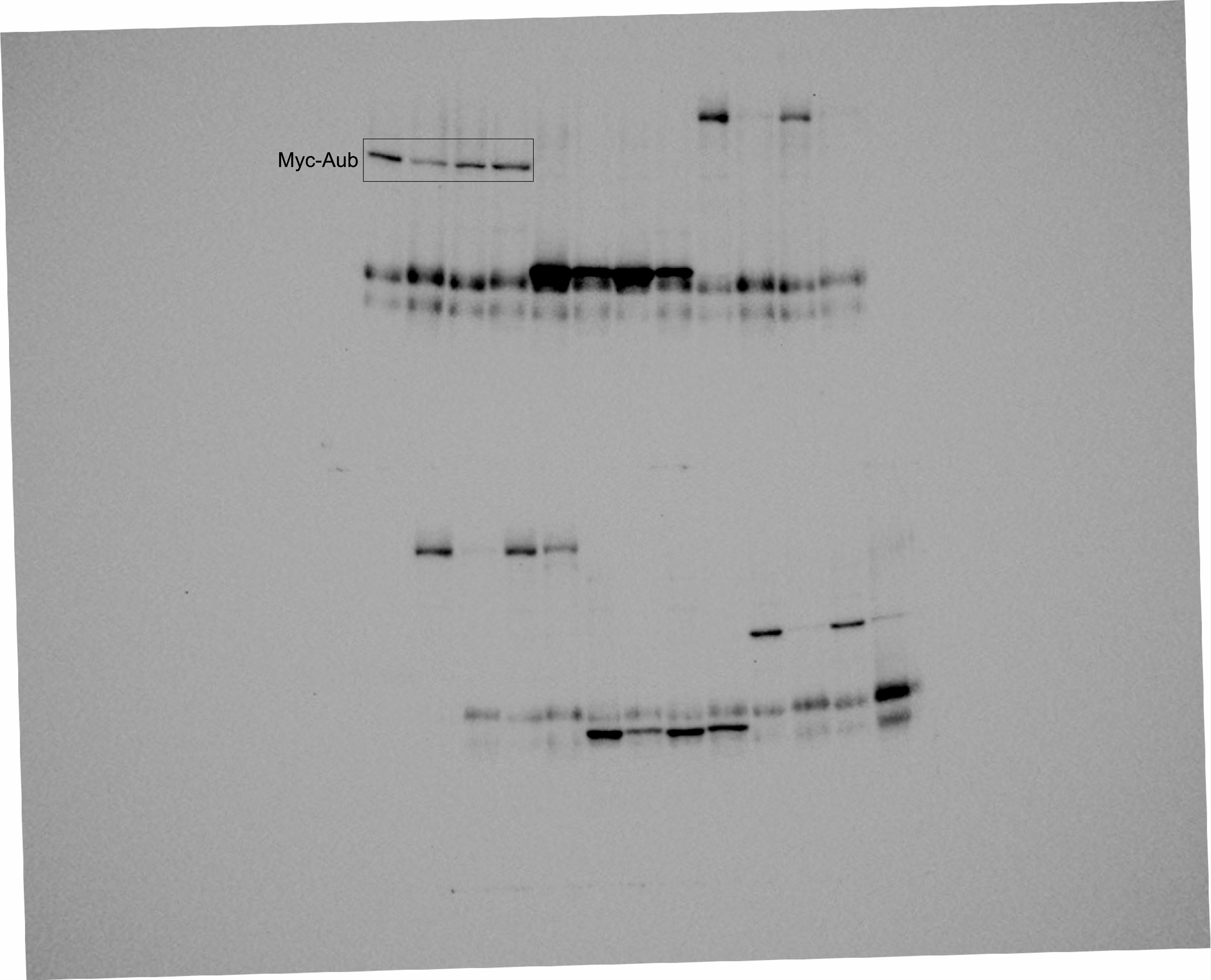

Supplement: Figure 1—source data 4. [file elife-101967-fig1-data4.zip › Figure 1-Source Data 4/Fig1D-ii_rep3_Myc_label_2023-09-01.tiff]

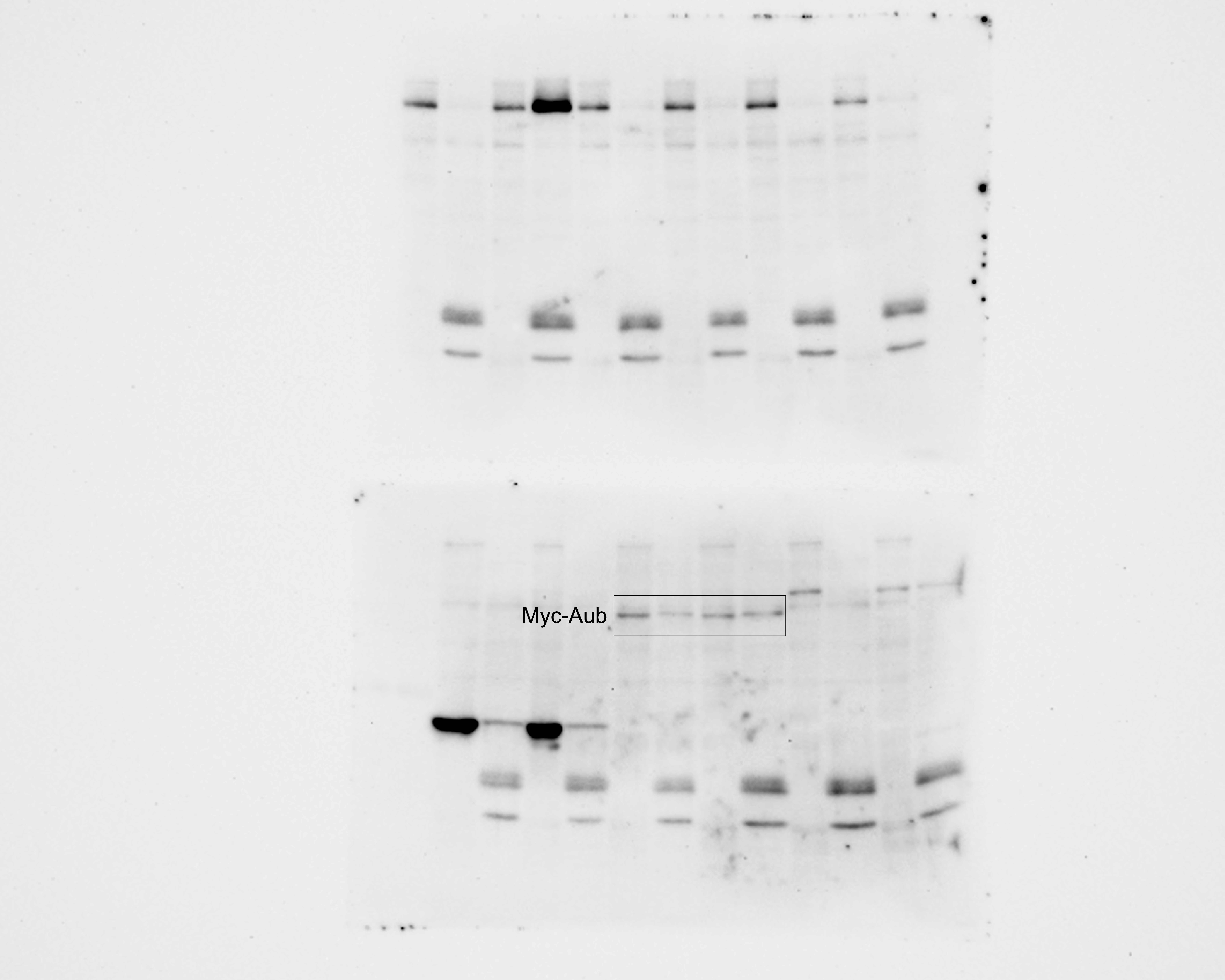

Supplement: Figure 1—source data 4. [file elife-101967-fig1-data4.zip › Figure 1-Source Data 4/Fig1D-ii_rep1_Myc_label_2023-08-01.tiff]

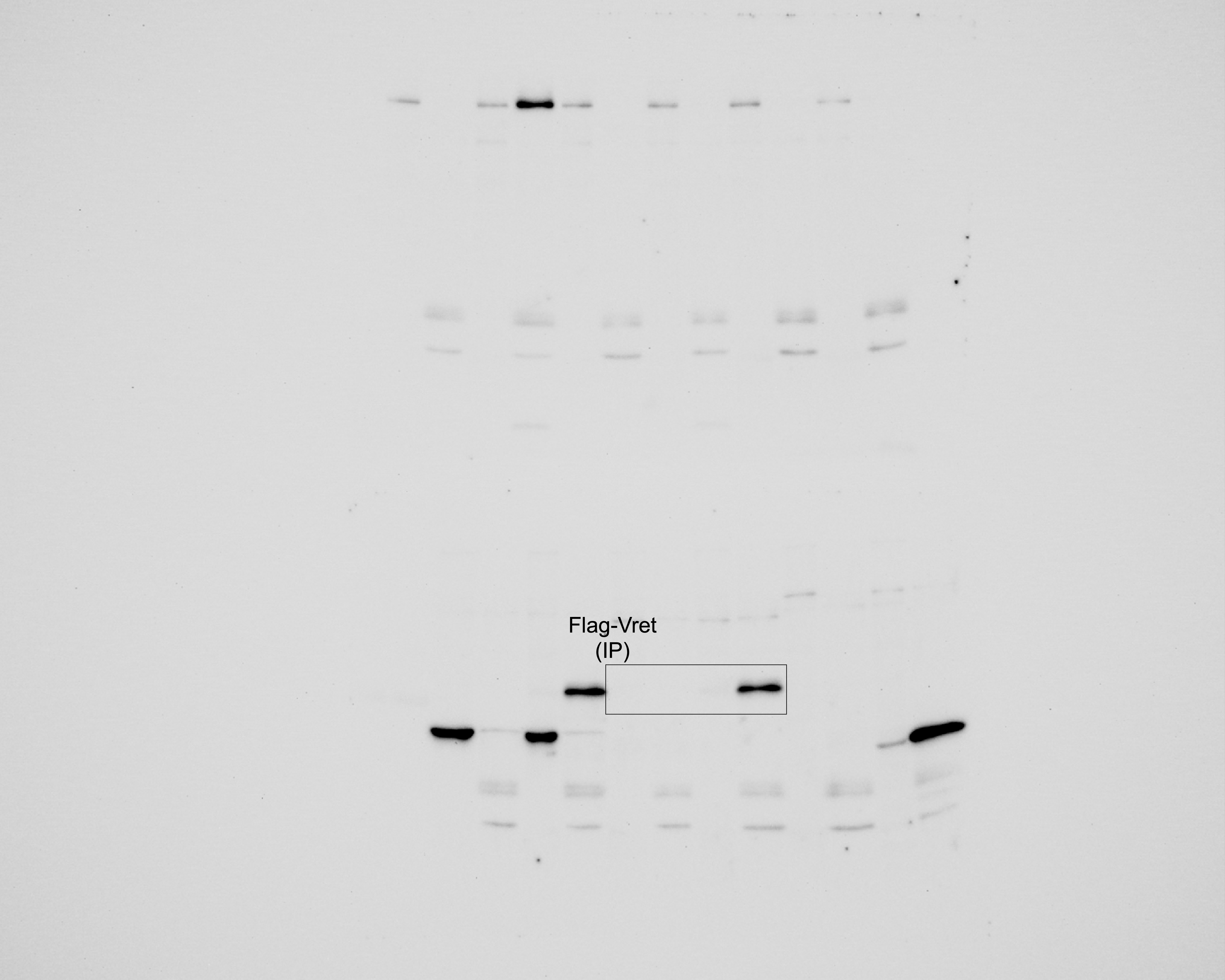

Supplement: Figure 1—source data 4. [file elife-101967-fig1-data4.zip › Figure 1-Source Data 4/Fig1D-ii_rep1_FLAG_label_2023-08-01.tiff]

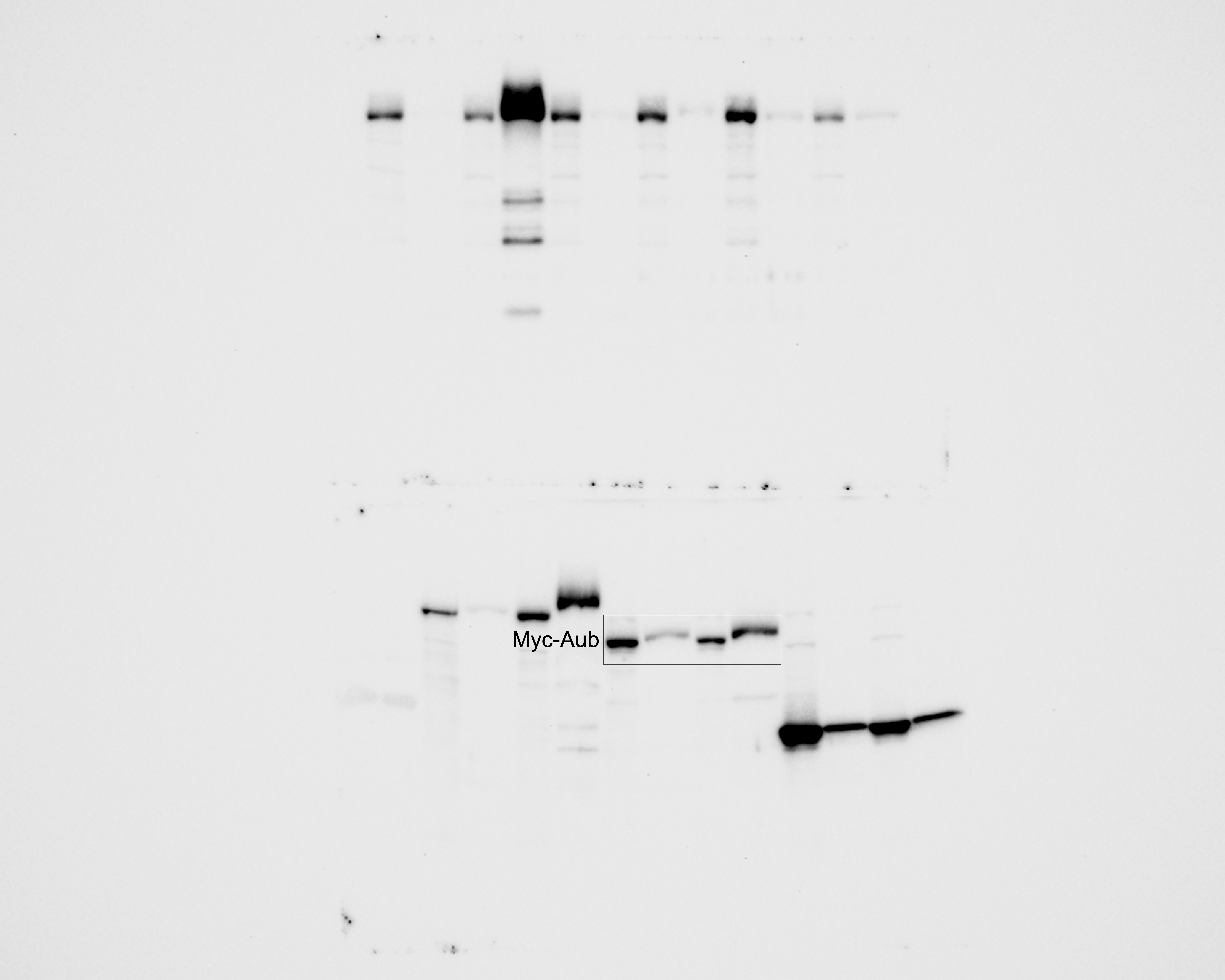

Supplement: Figure 1—source data 4. [file elife-101967-fig1-data4.zip › Figure 1-Source Data 4/Fig1D-ii_rep2_Myc_label_2023-08-25.tiff]

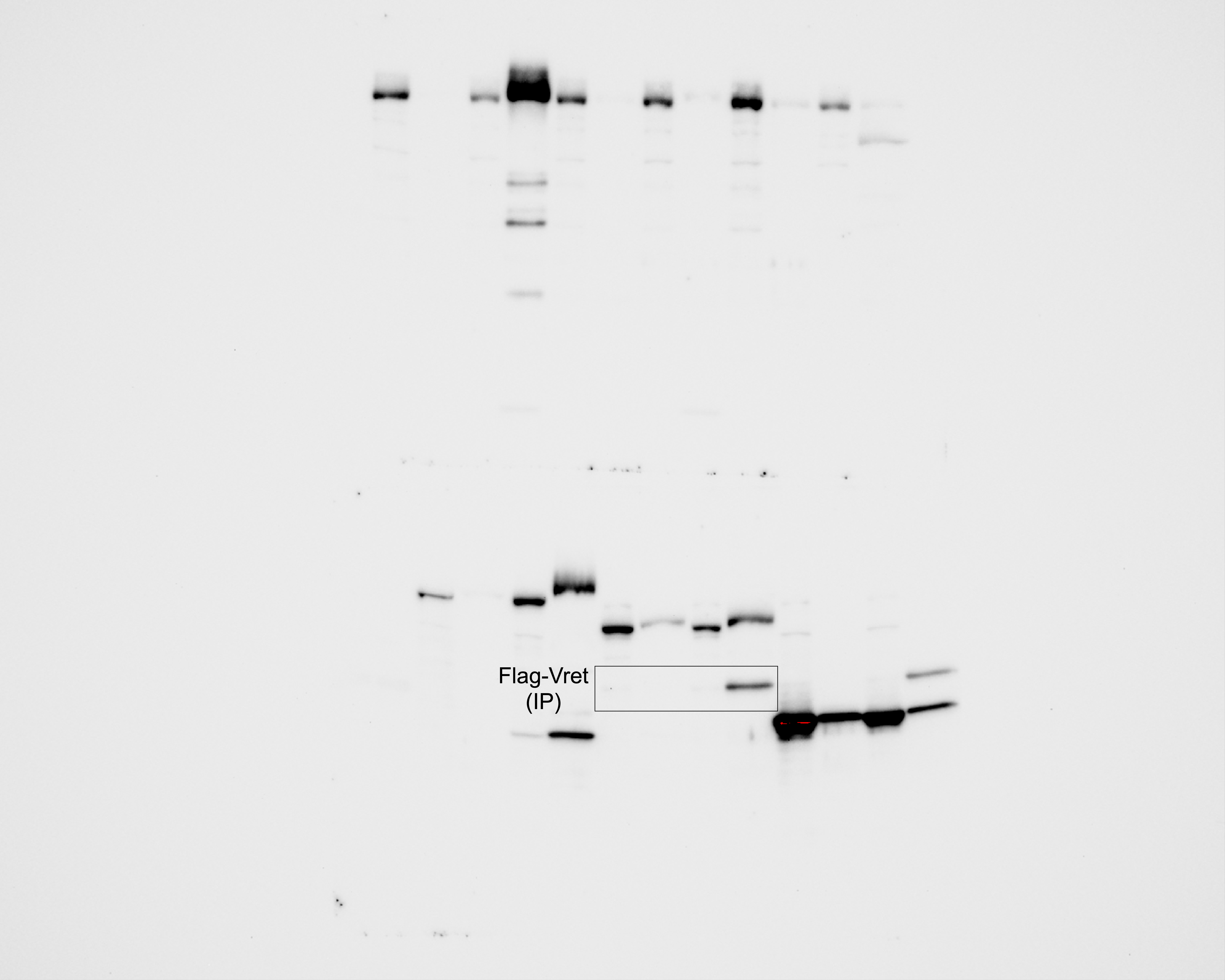

Supplement: Figure 1—source data 4. [file elife-101967-fig1-data4.zip › Figure 1-Source Data 4/Fig1D-ii_rep2_FLAG_label_2023-08-25.tiff]

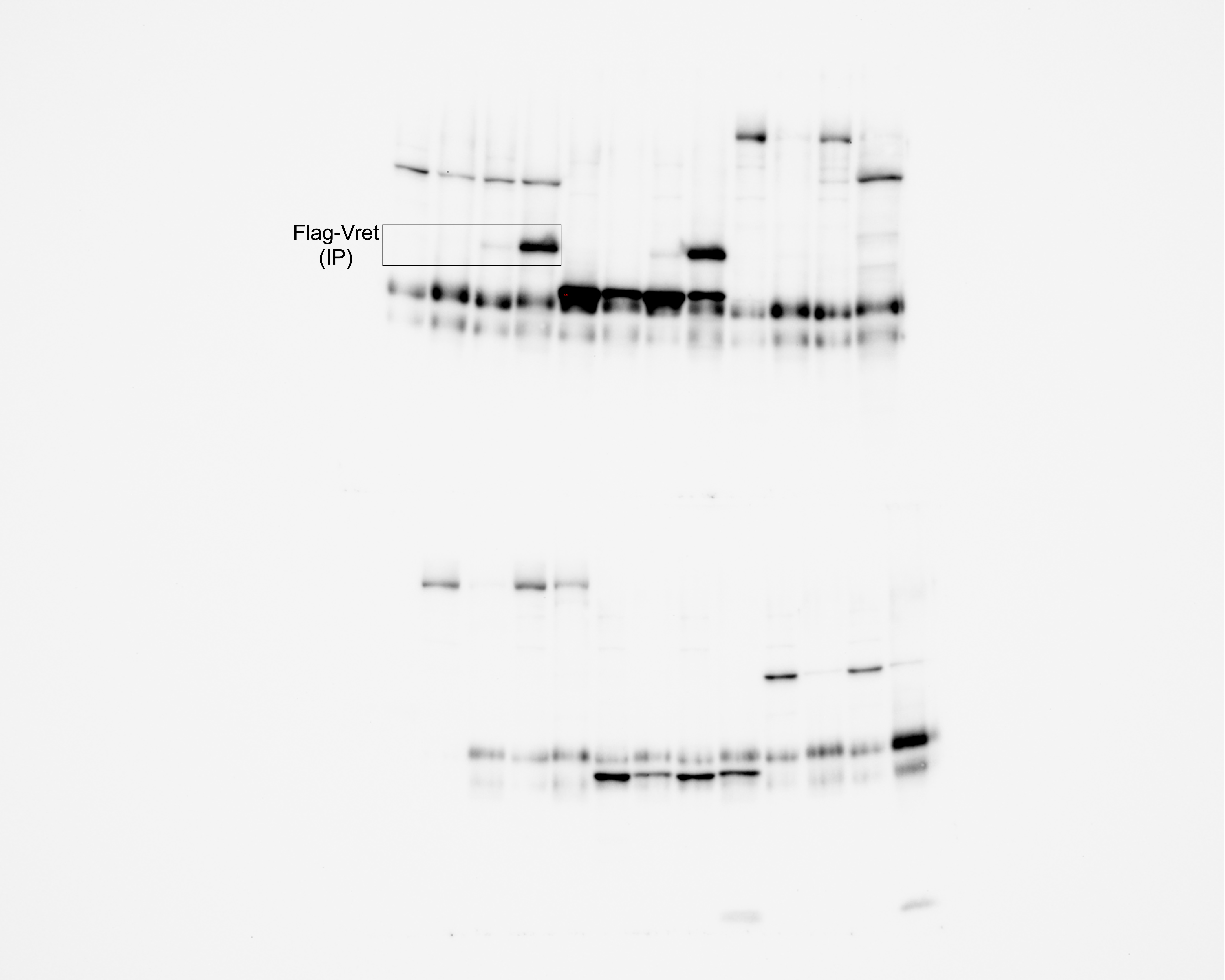

Supplement: Figure 1—source data 4. [file elife-101967-fig1-data4.zip › Figure 1-Source Data 4/Fig1D-ii_rep3_FLAG_label_2023-09-01.tiff]

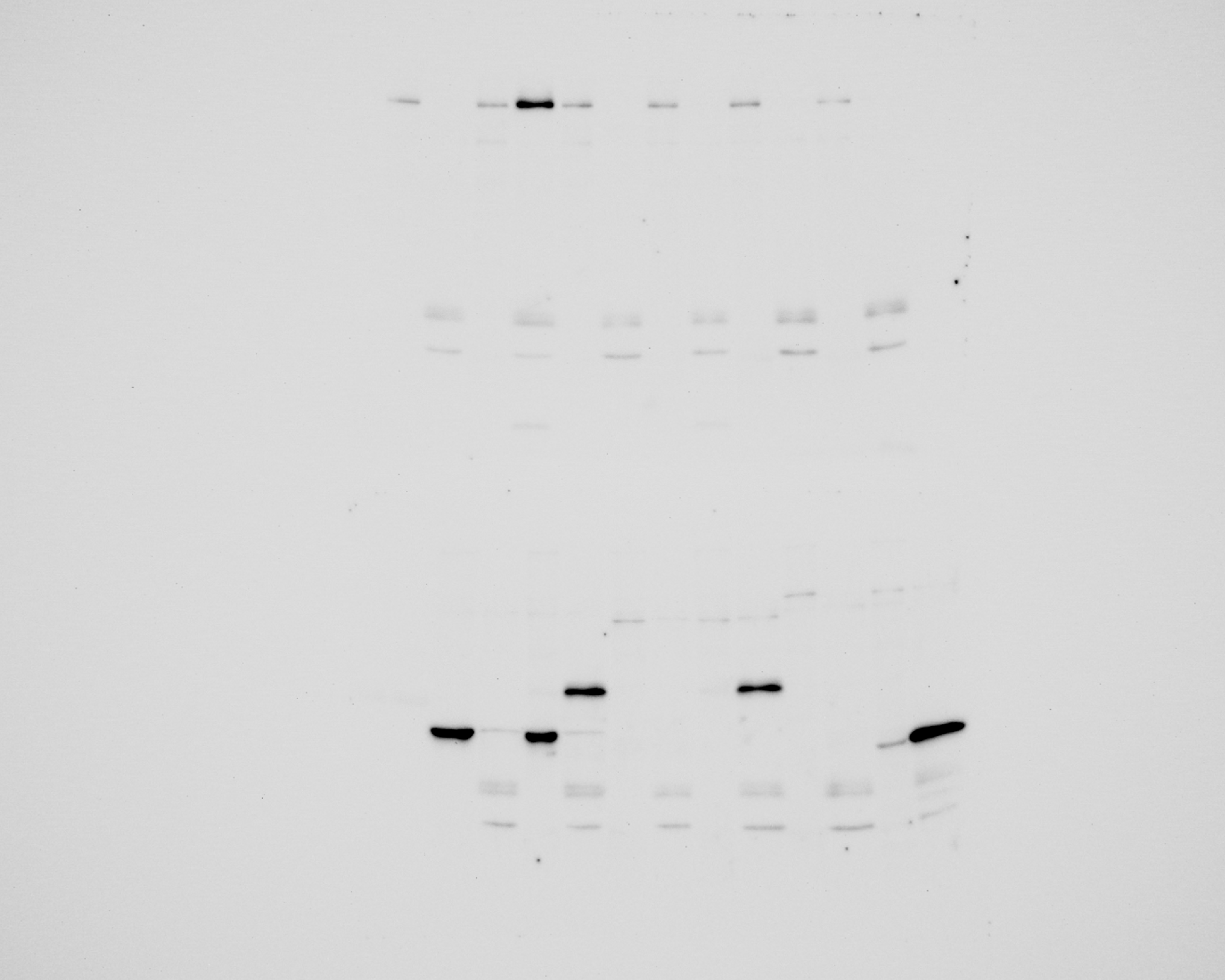

Supplement: Figure 1—source data 5. [file elife-101967-fig1-data5.zip › Figure 1-Source Data 5/Fig1D-ii_rep1_FLAG_original_2023-08-01.tif]

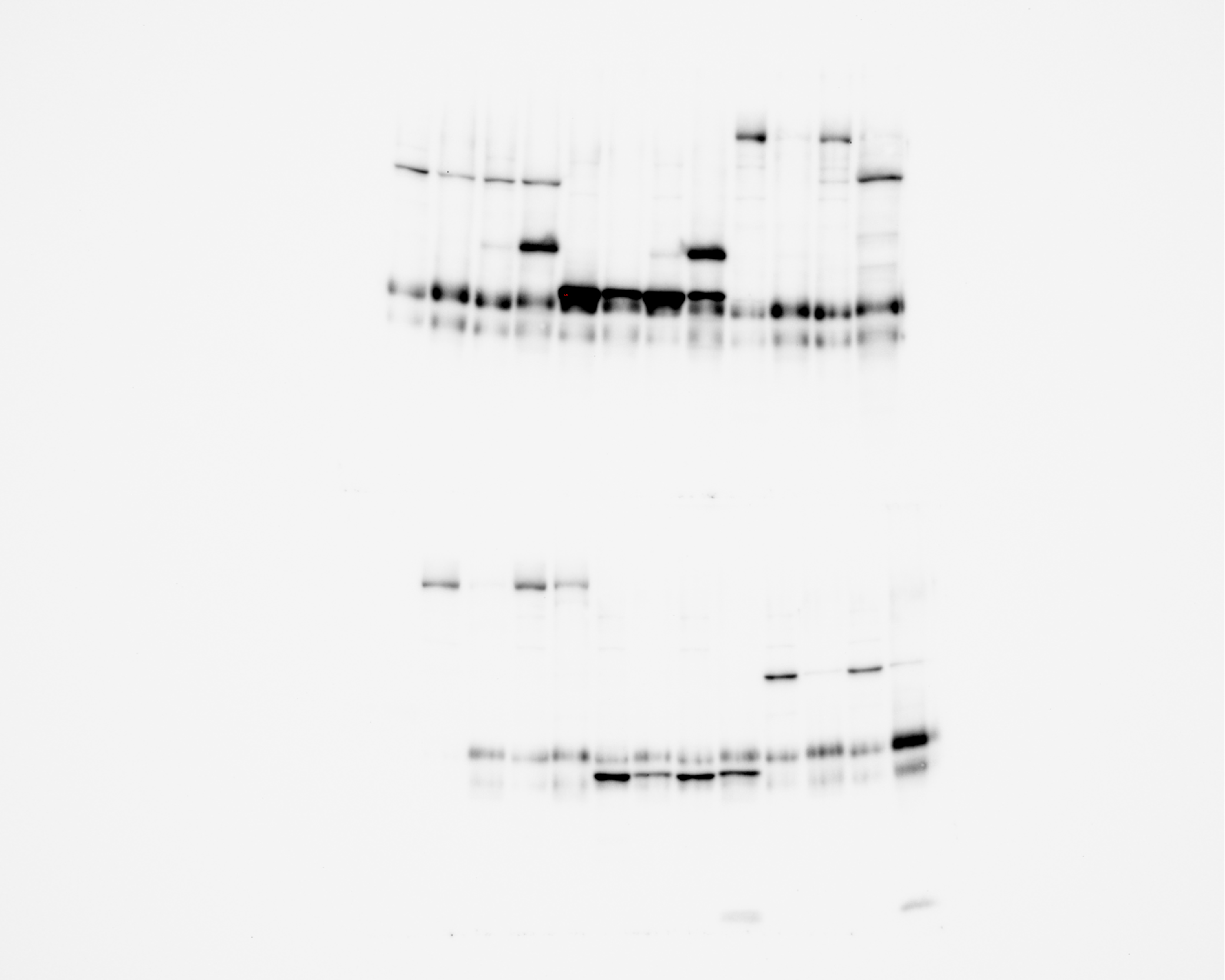

Supplement: Figure 1—source data 5. [file elife-101967-fig1-data5.zip › Figure 1-Source Data 5/Fig1D-ii_rep3_FLAG _original_2023-09-01.tif]

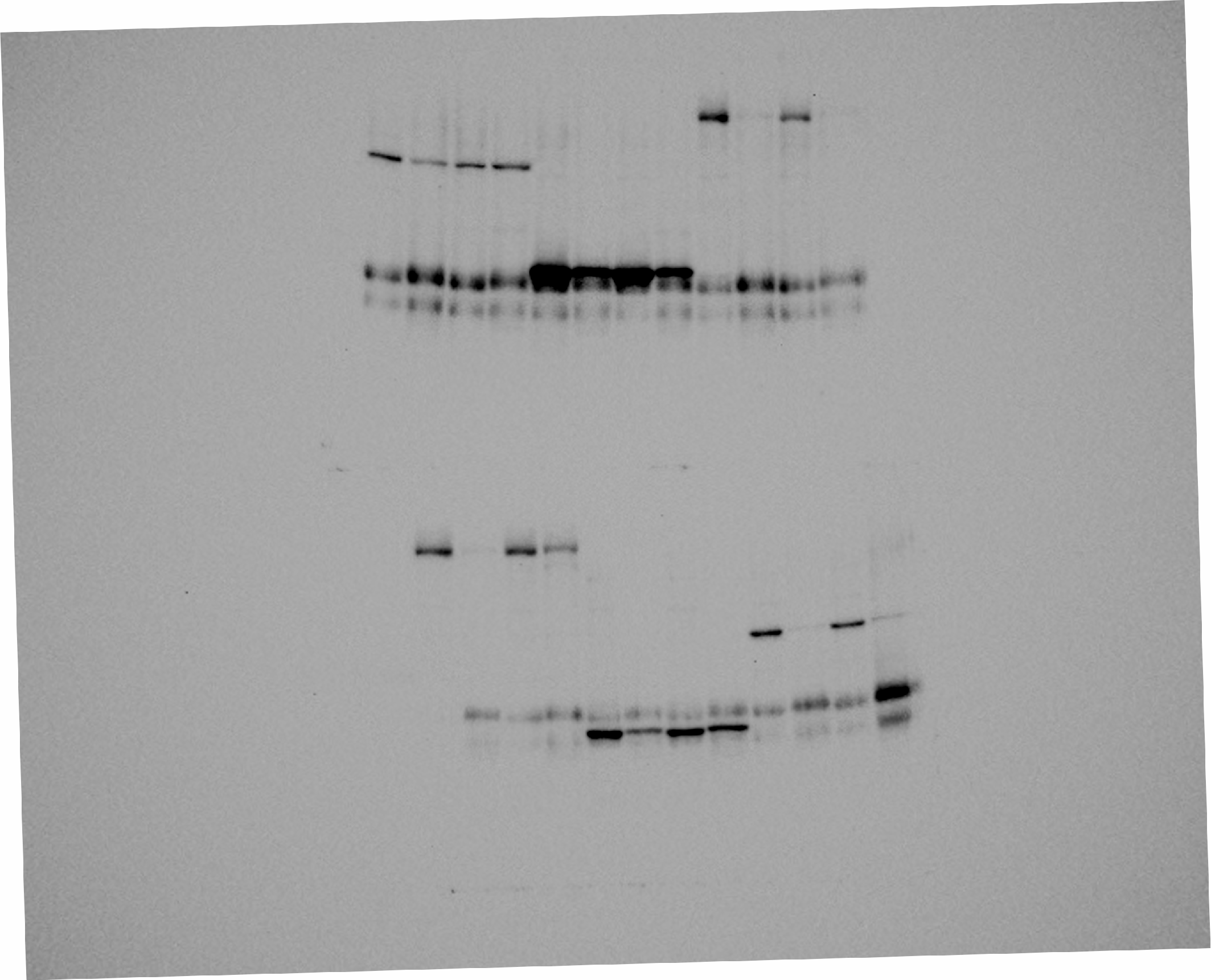

Supplement: Figure 1—source data 5. [file elife-101967-fig1-data5.zip › Figure 1-Source Data 5/Fig1D-ii_rep3_Myc_original_2023-09-01.tif]

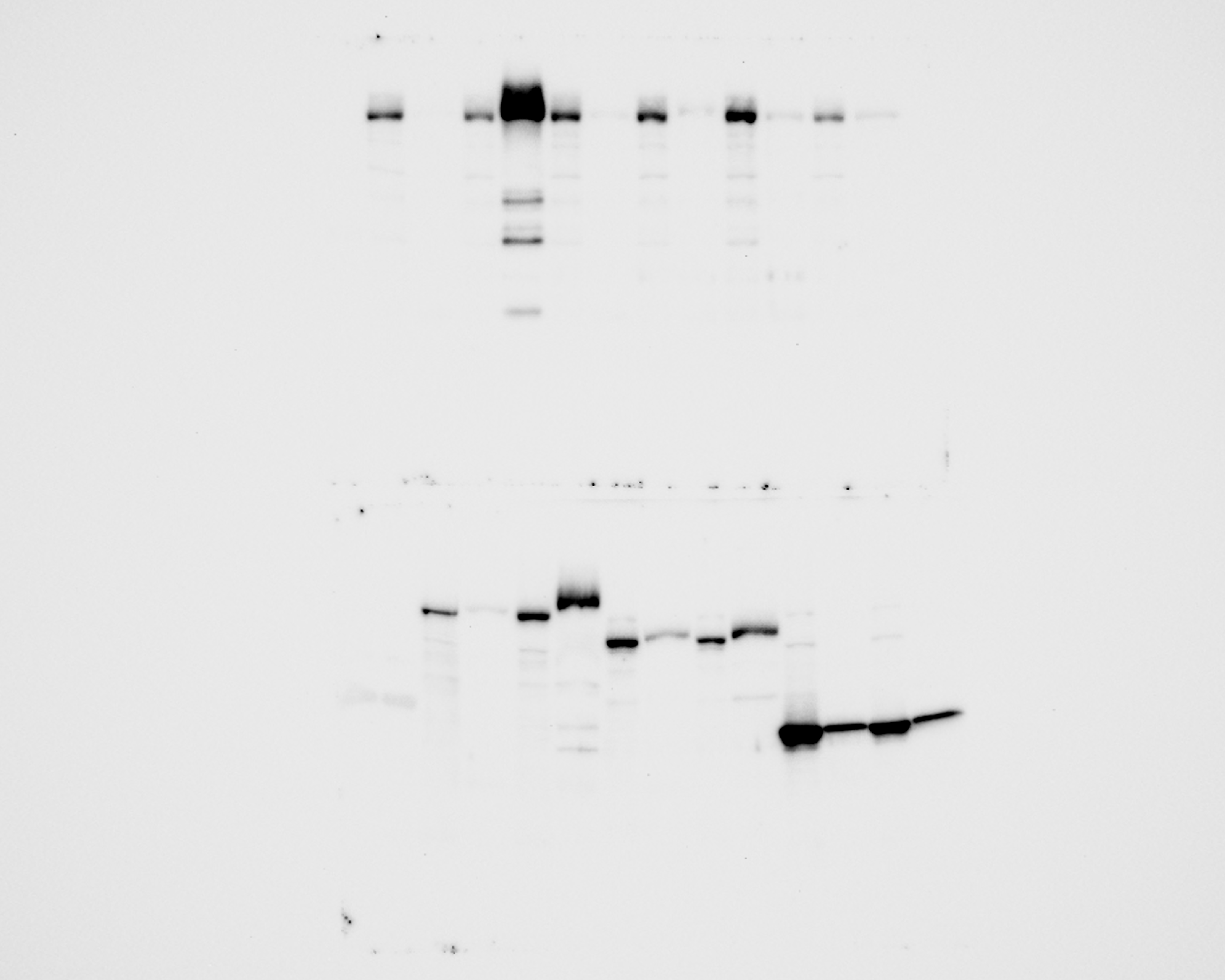

Supplement: Figure 1—source data 5. [file elife-101967-fig1-data5.zip › Figure 1-Source Data 5/Fig1D-ii_rep2_Myc_original_2023-08-25.tif]

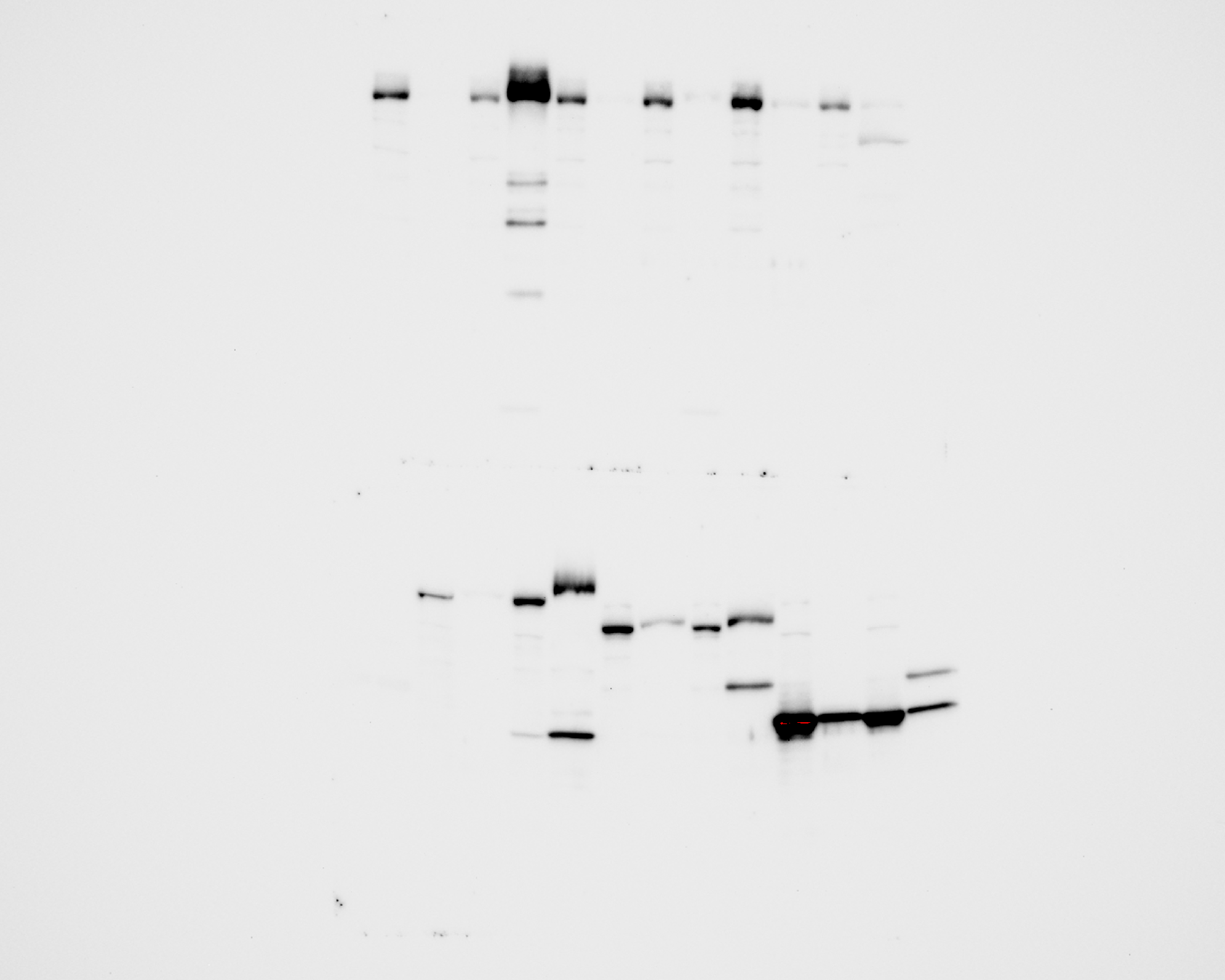

Supplement: Figure 1—source data 5. [file elife-101967-fig1-data5.zip › Figure 1-Source Data 5/Fig1D-ii_rep2_FLAG_original_2023-08-25.tif]

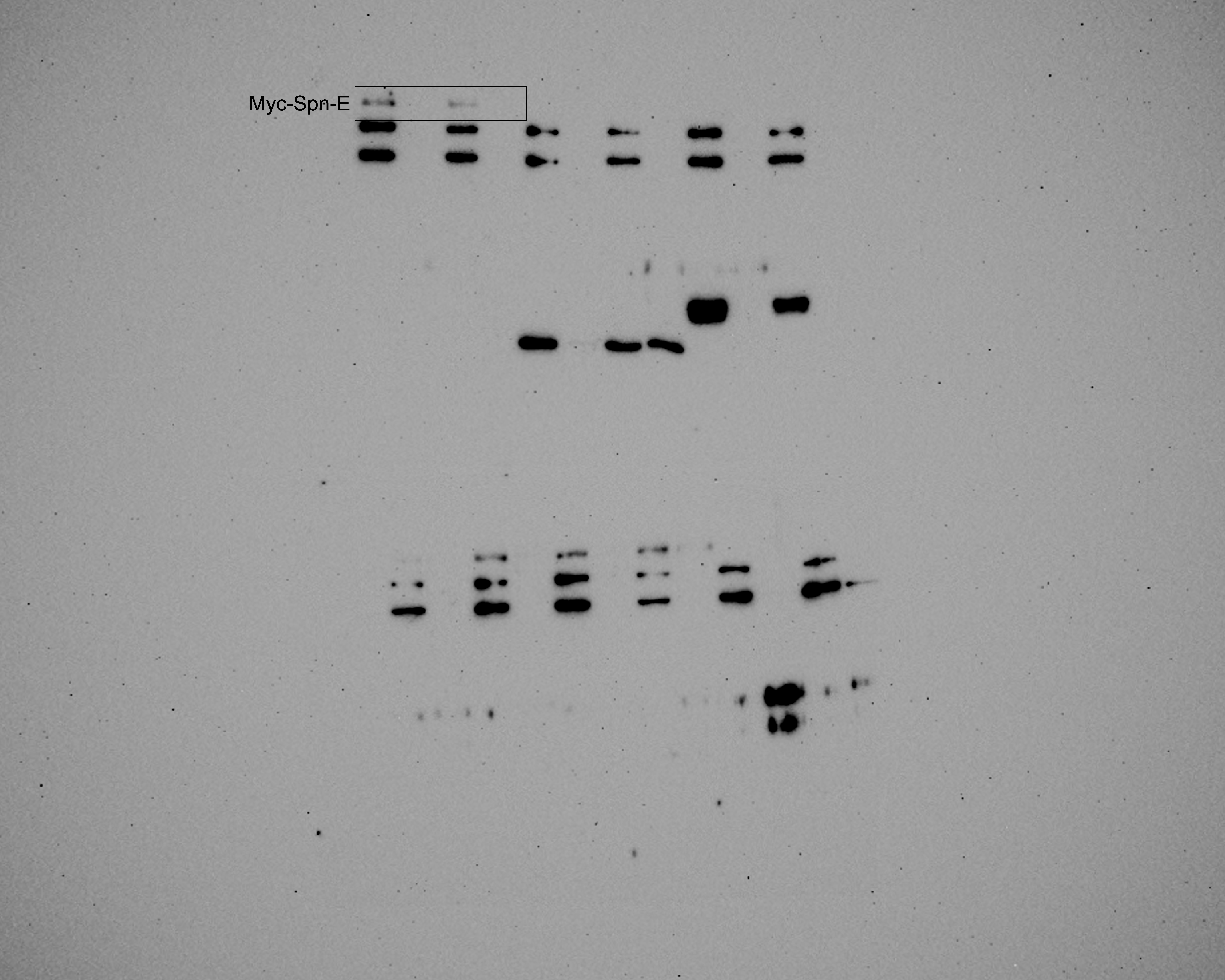

Supplement: Figure 1—source data 6. [file elife-101967-fig1-data6.zip › Figure 1-Source Data 6/Fig1D-iii_rep3_Myc_label_2024-06-01.tiff]

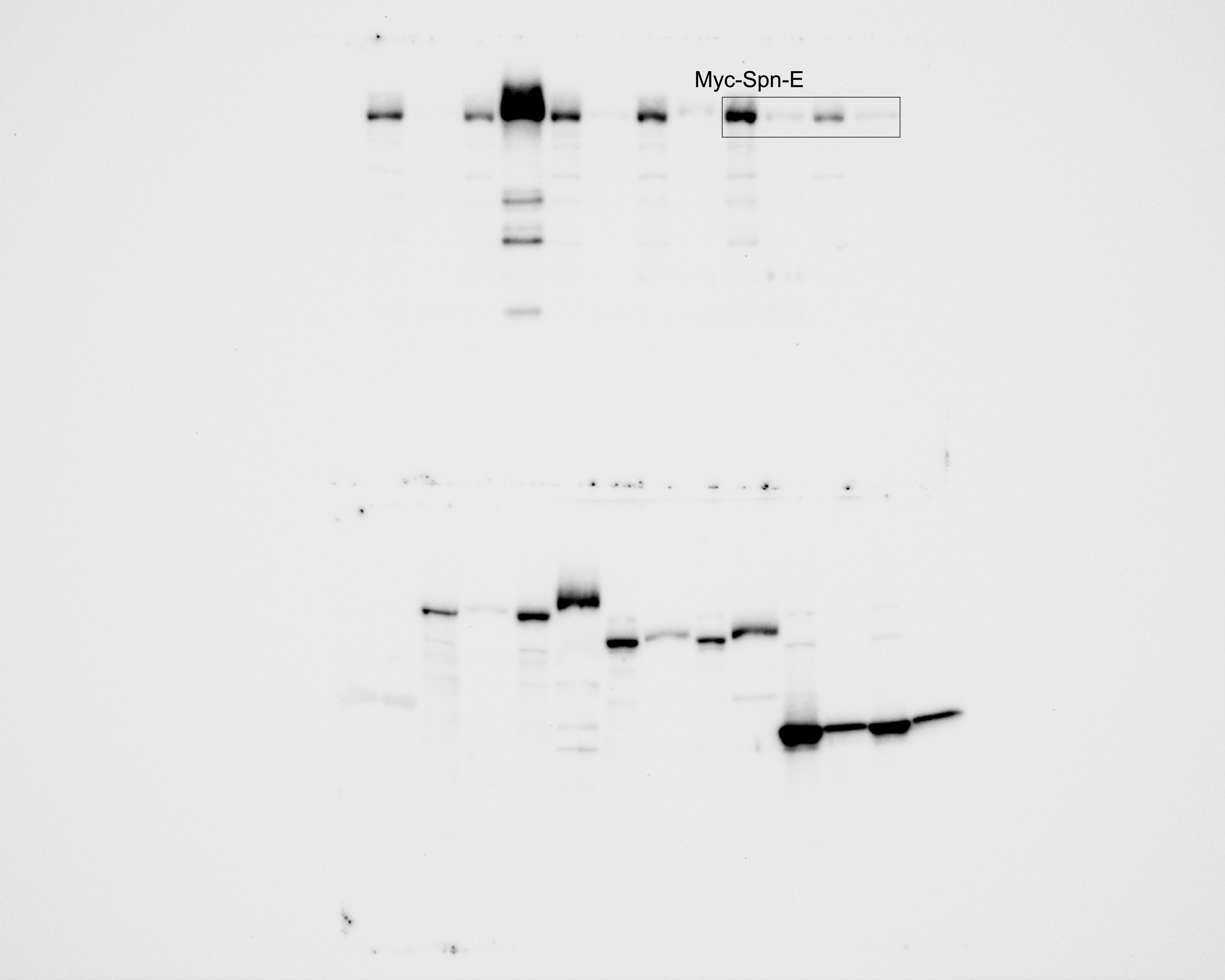

Supplement: Figure 1—source data 6. [file elife-101967-fig1-data6.zip › Figure 1-Source Data 6/Fig1D-iii_rep1_Myc_label_2023-08-25.tiff]

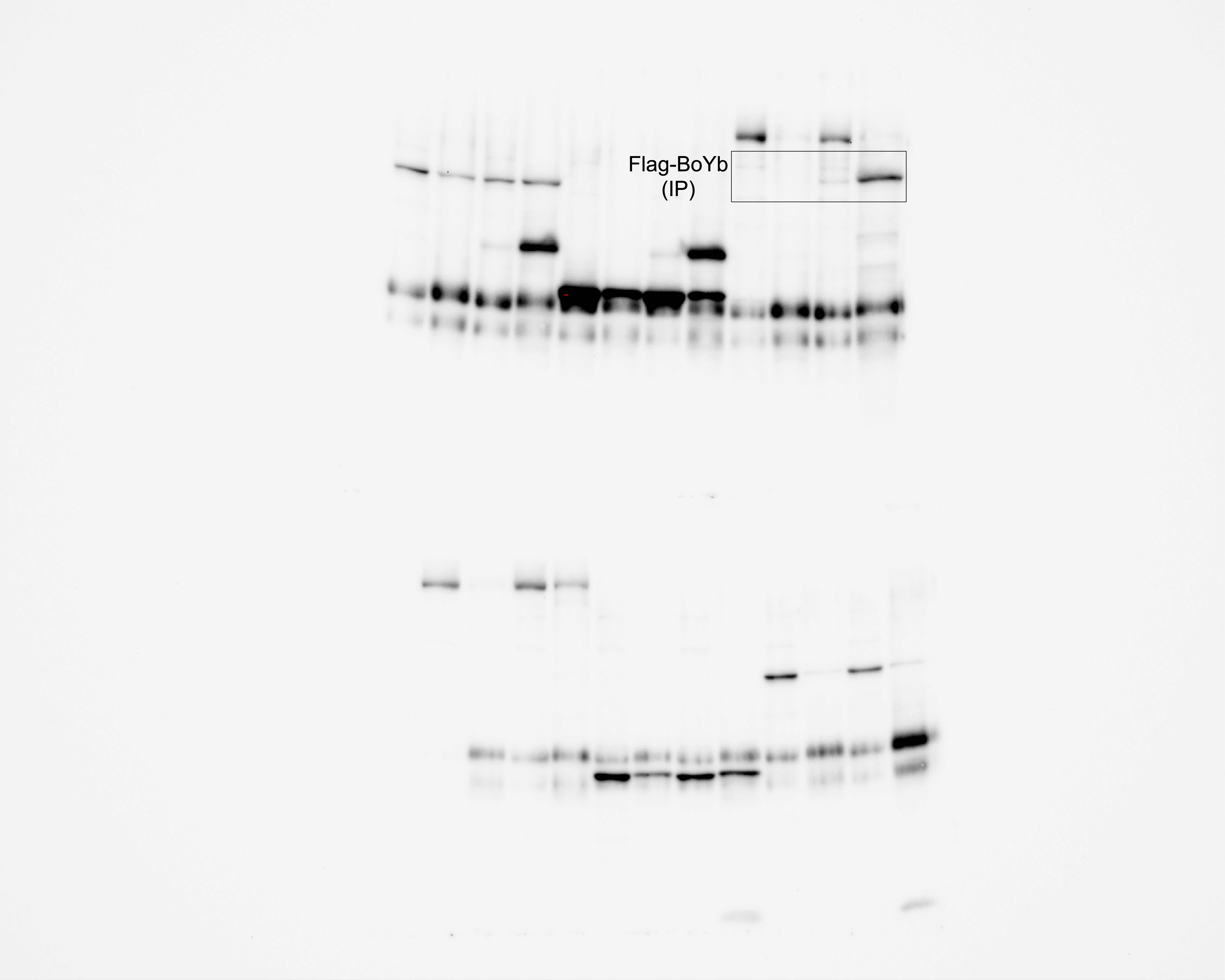

Supplement: Figure 1—source data 6. [file elife-101967-fig1-data6.zip › Figure 1-Source Data 6/Fig1D-iii_rep2_FLAG_label_2023-09-01.tiff]

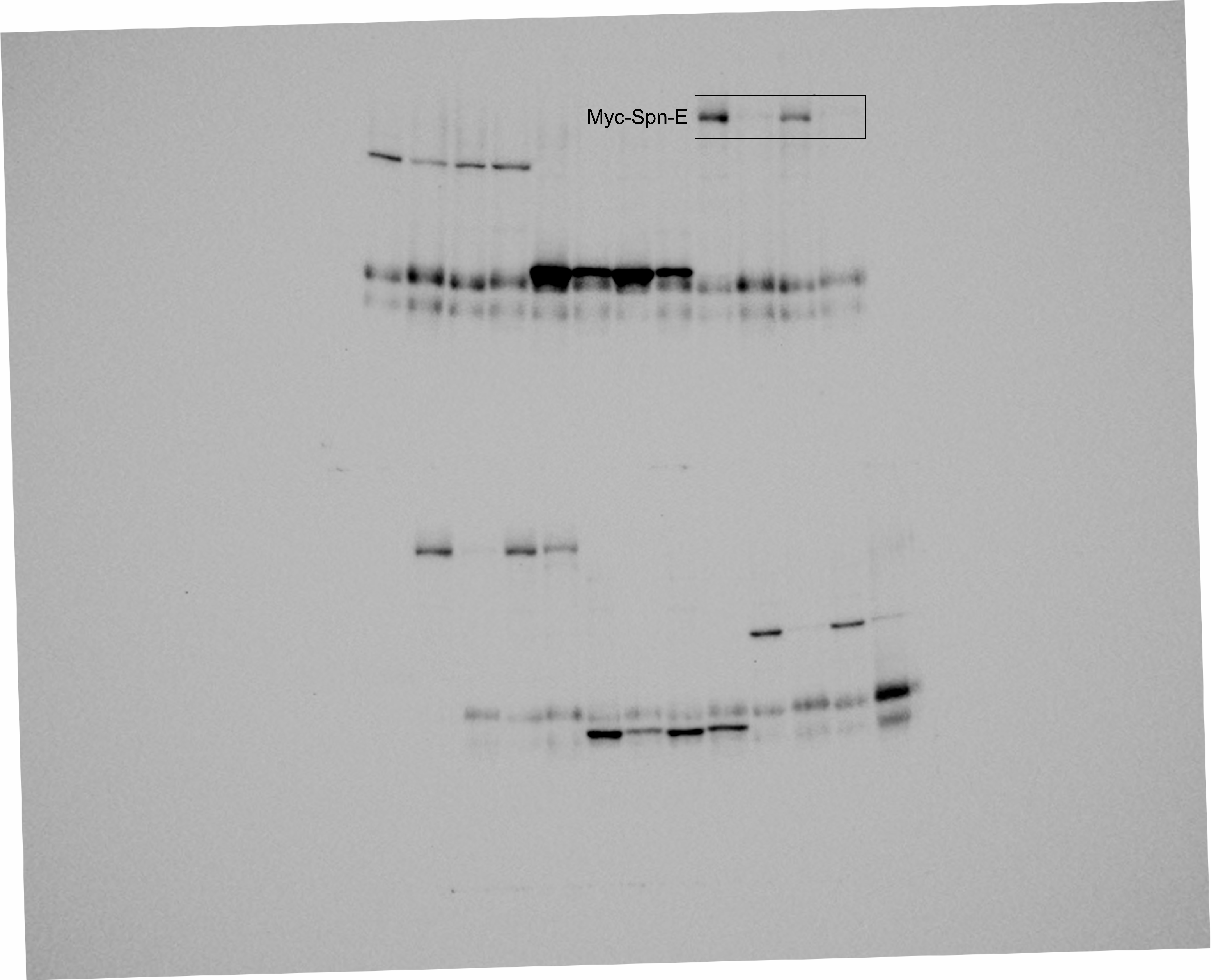

Supplement: Figure 1—source data 6. [file elife-101967-fig1-data6.zip › Figure 1-Source Data 6/Fig1D-iii_rep2_Myc_label_2023-09-01.tiff]

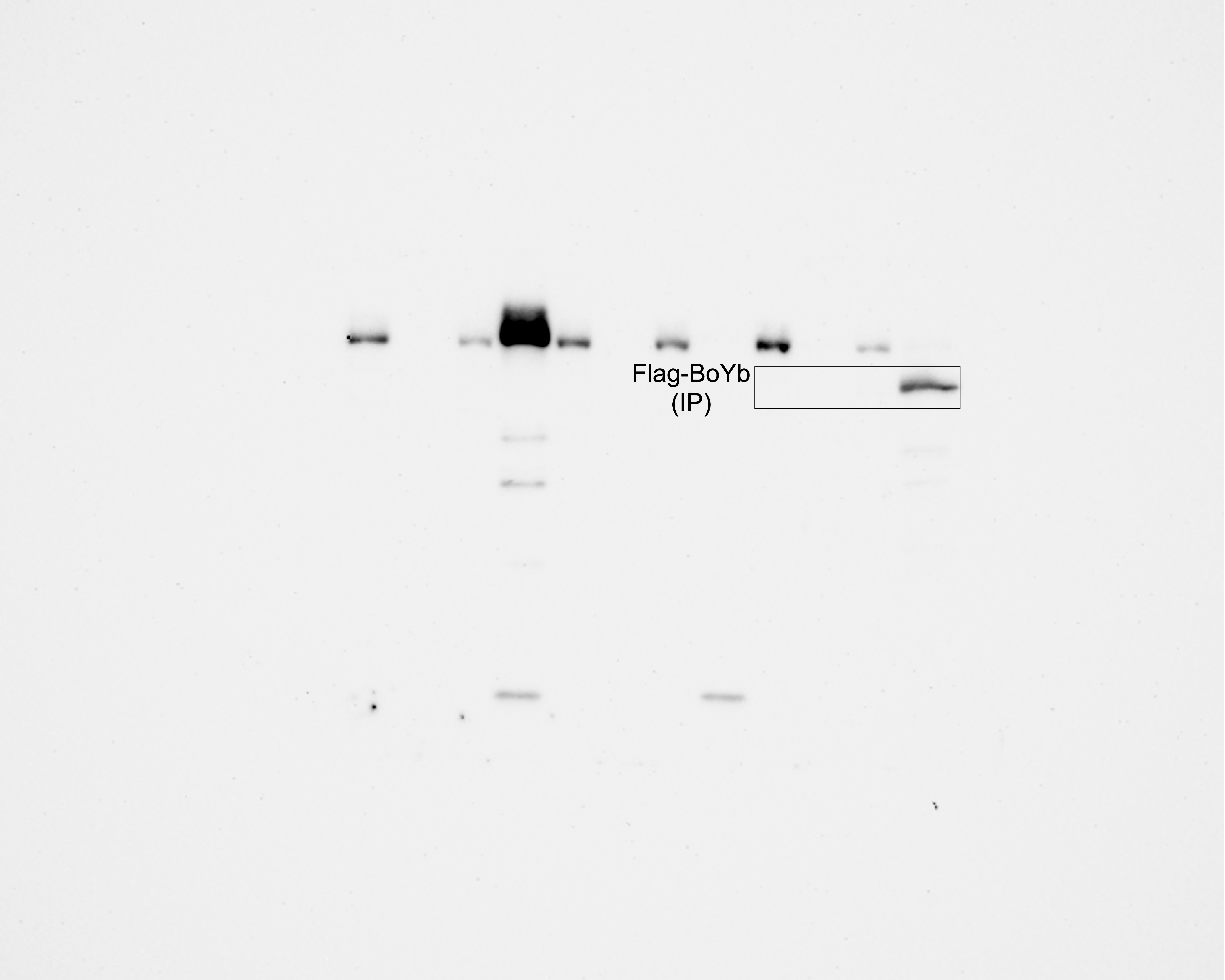

Supplement: Figure 1—source data 6. [file elife-101967-fig1-data6.zip › Figure 1-Source Data 6/Fig1D-iii_rep1_FLAG_label_2023-08-25.tiff]

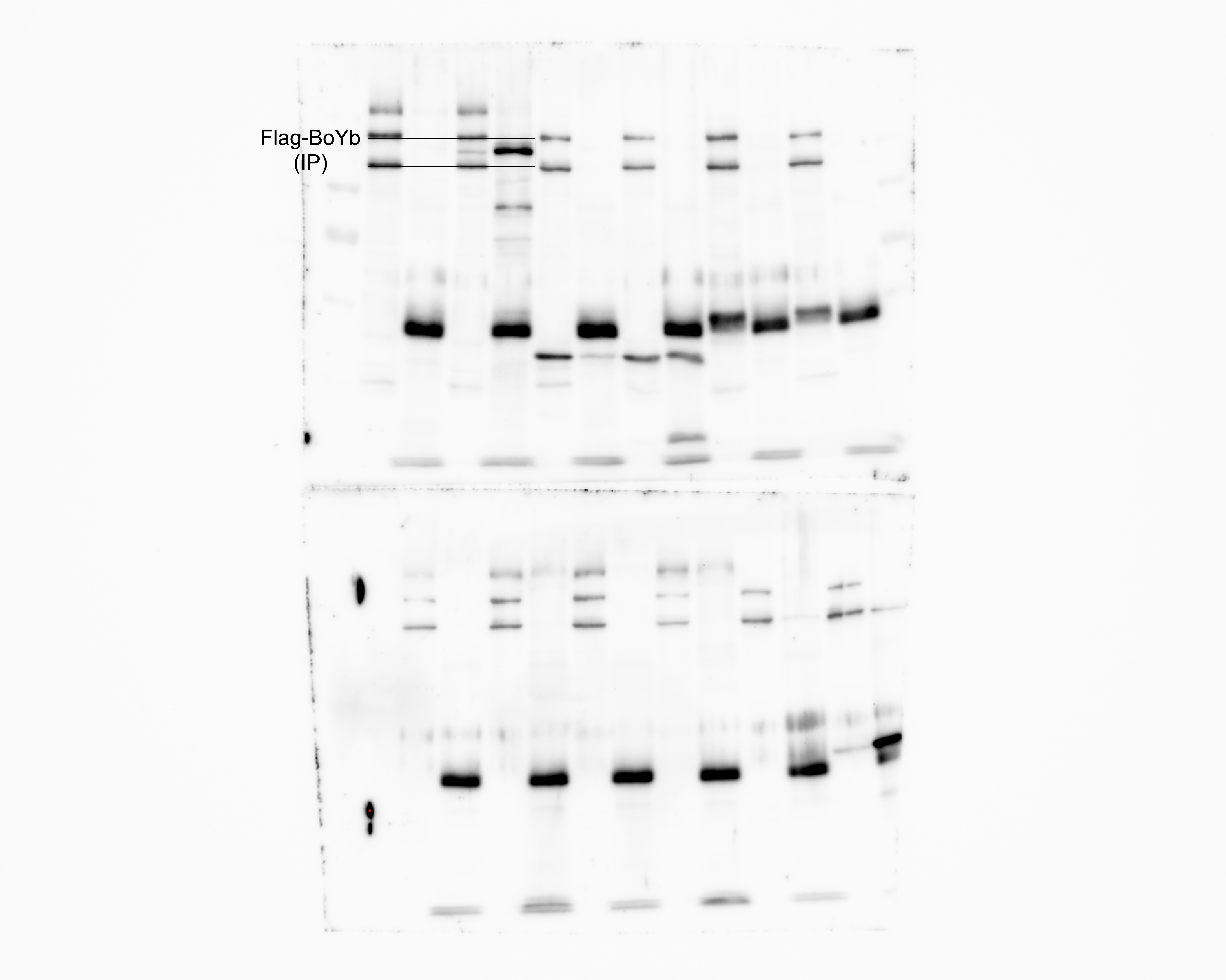

Supplement: Figure 1—source data 6. [file elife-101967-fig1-data6.zip › Figure 1-Source Data 6/Fig1D-iii_rep3_FLAG_label_2024-06-03.tiff]

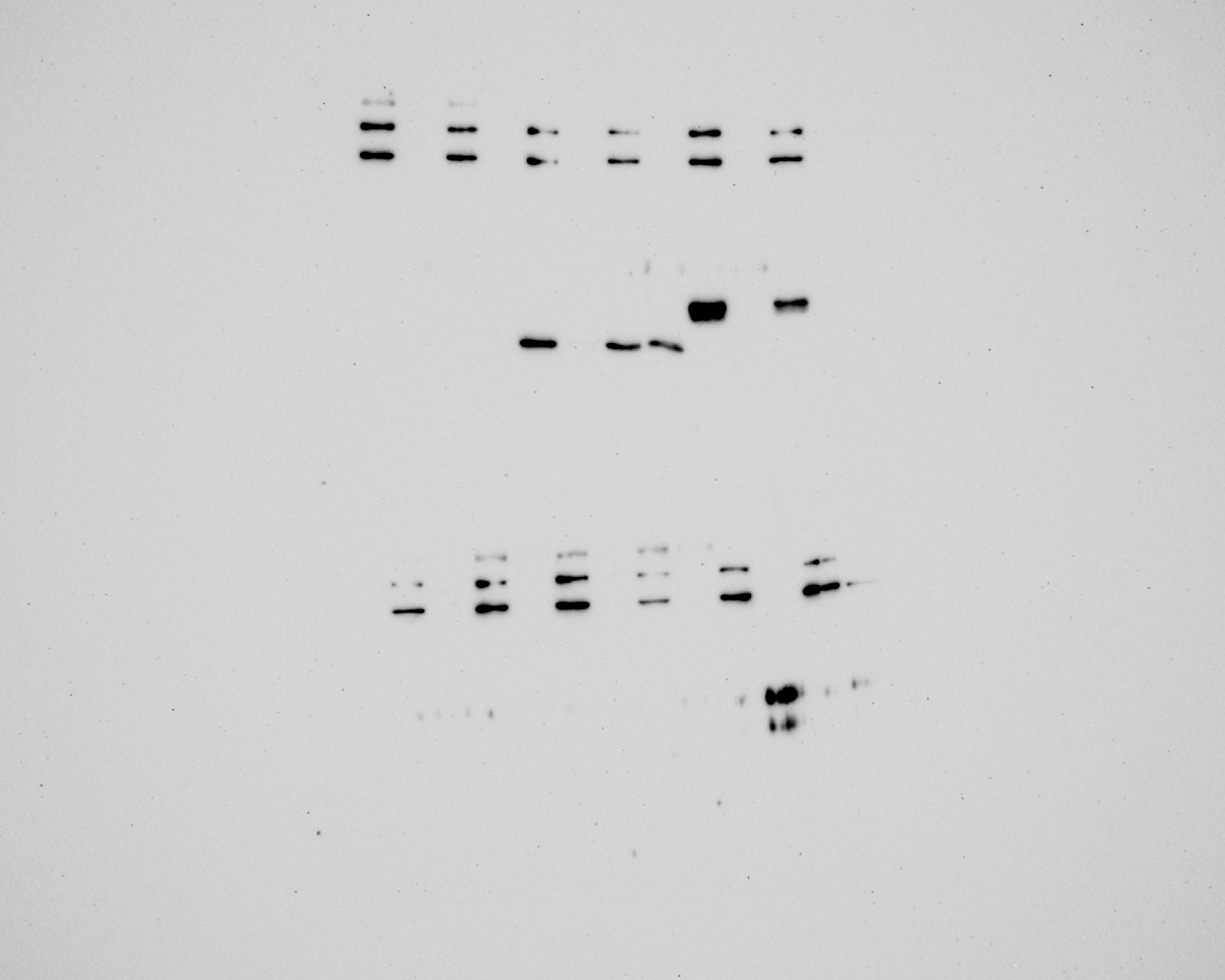

Supplement: Figure 1—source data 7. [file elife-101967-fig1-data7.zip › Figure 1-Source Data 7/Fig1D-iii_rep3_Myc_original_2024-06-01.tif]

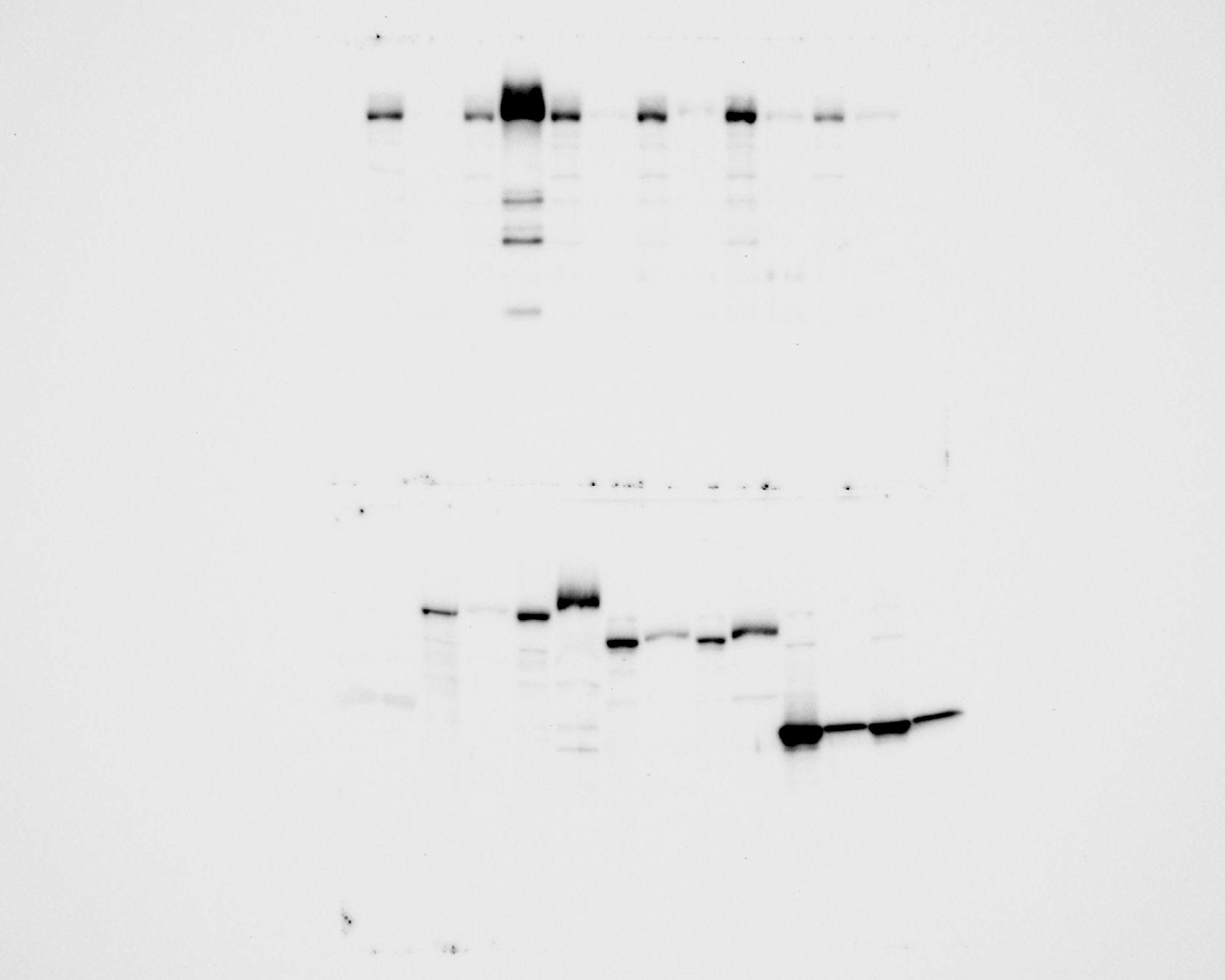

Supplement: Figure 1—source data 7. [file elife-101967-fig1-data7.zip › Figure 1-Source Data 7/Fig1D-iii_rep1_Myc_original_2023-08-25.tif]

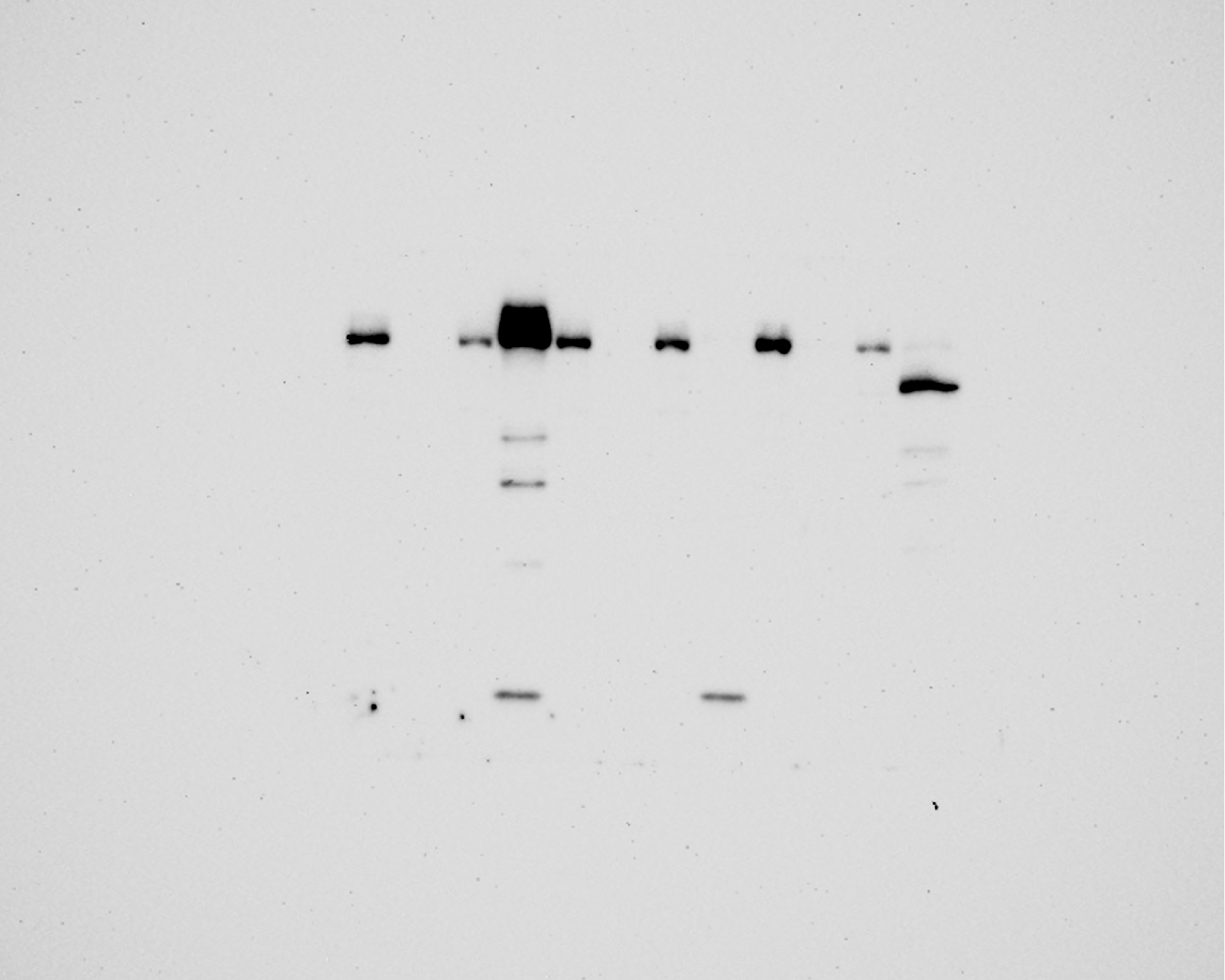

Supplement: Figure 1—source data 7. [file elife-101967-fig1-data7.zip › Figure 1-Source Data 7/Fig1D-iii_rep1_FLAG_original_2023-08-25.tif]

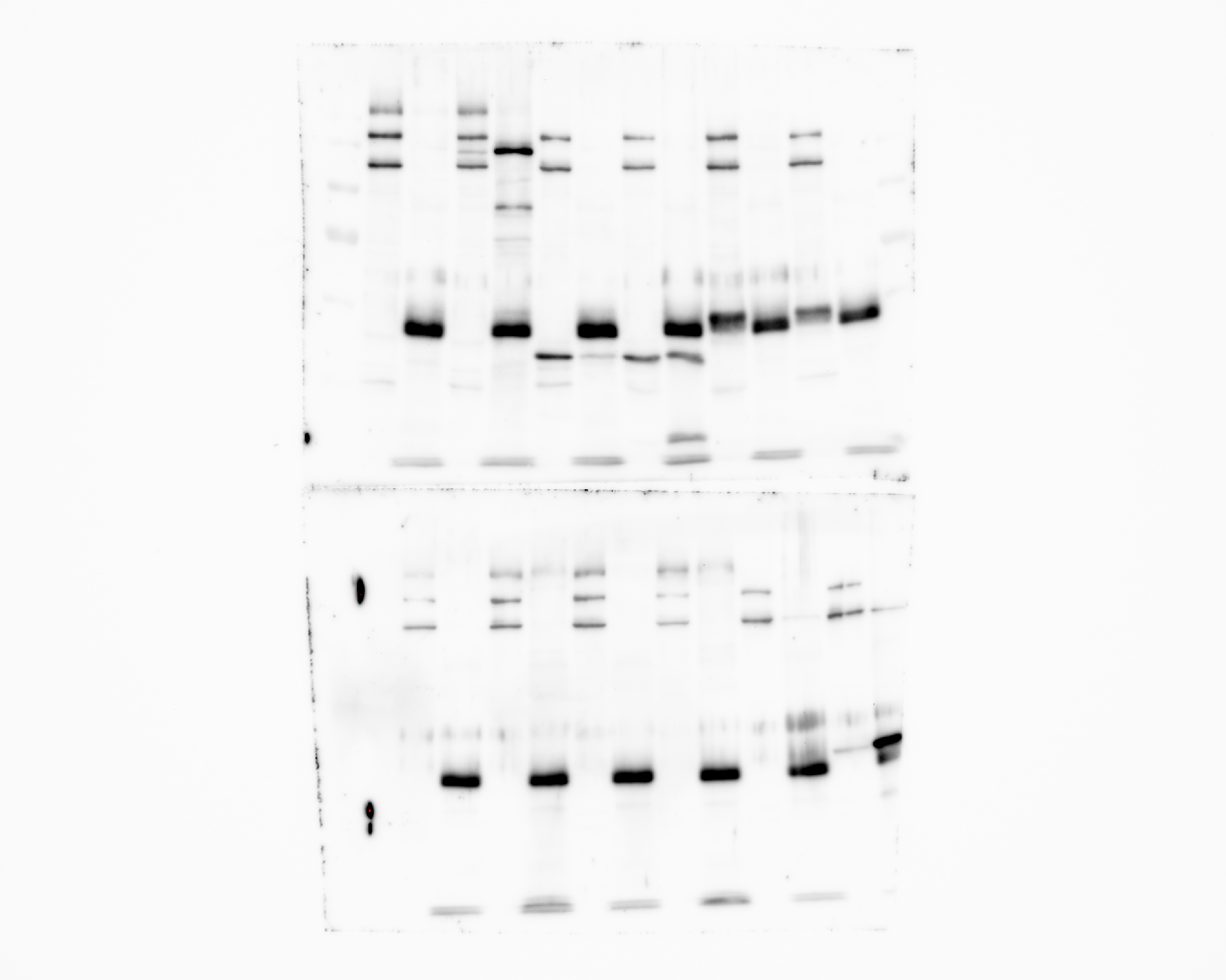

Supplement: Figure 1—source data 7. [file elife-101967-fig1-data7.zip › Figure 1-Source Data 7/Fig1D-iii_rep3_FLAG_original_2024-06-03.tif]

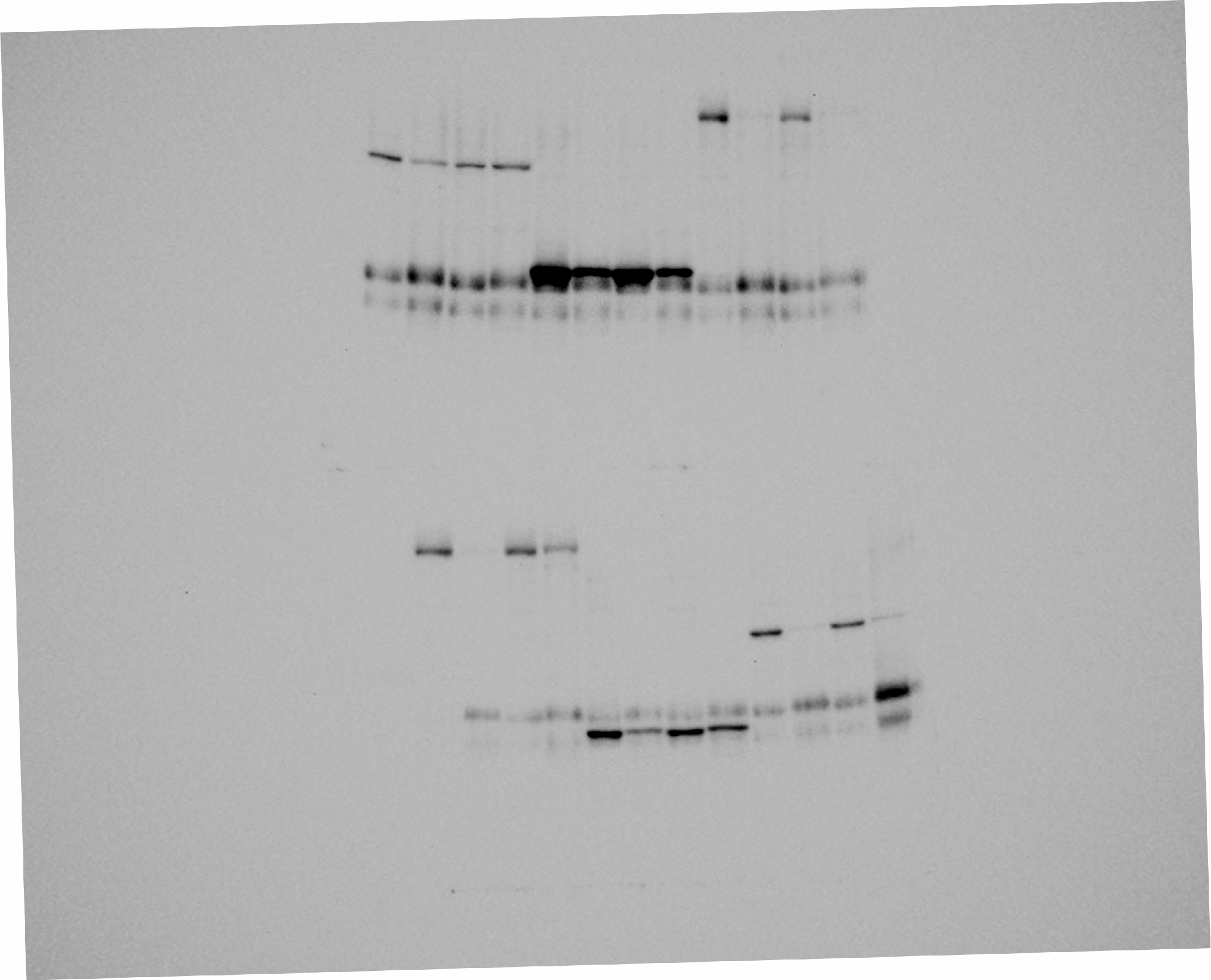

Supplement: Figure 1—source data 7. [file elife-101967-fig1-data7.zip › Figure 1-Source Data 7/Fig1D-iii_rep2_Myc1_original_2023-09-01.tif]

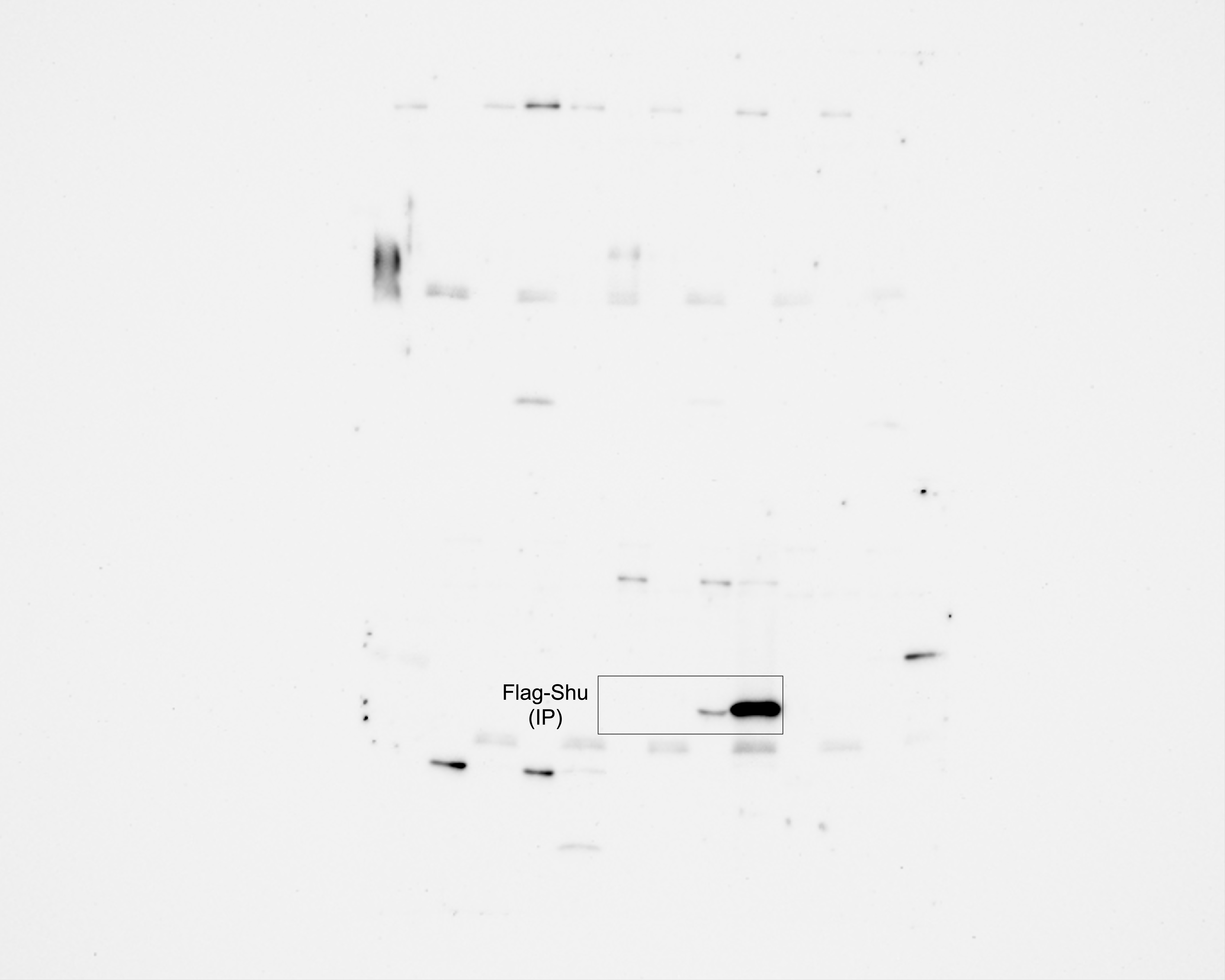

Supplement: Figure 1—source data 8. [file elife-101967-fig1-data8.zip › Figure 1-Source Data 8/Fig1D-iv_rep2_FLAG_label_2023-08-17.tiff]

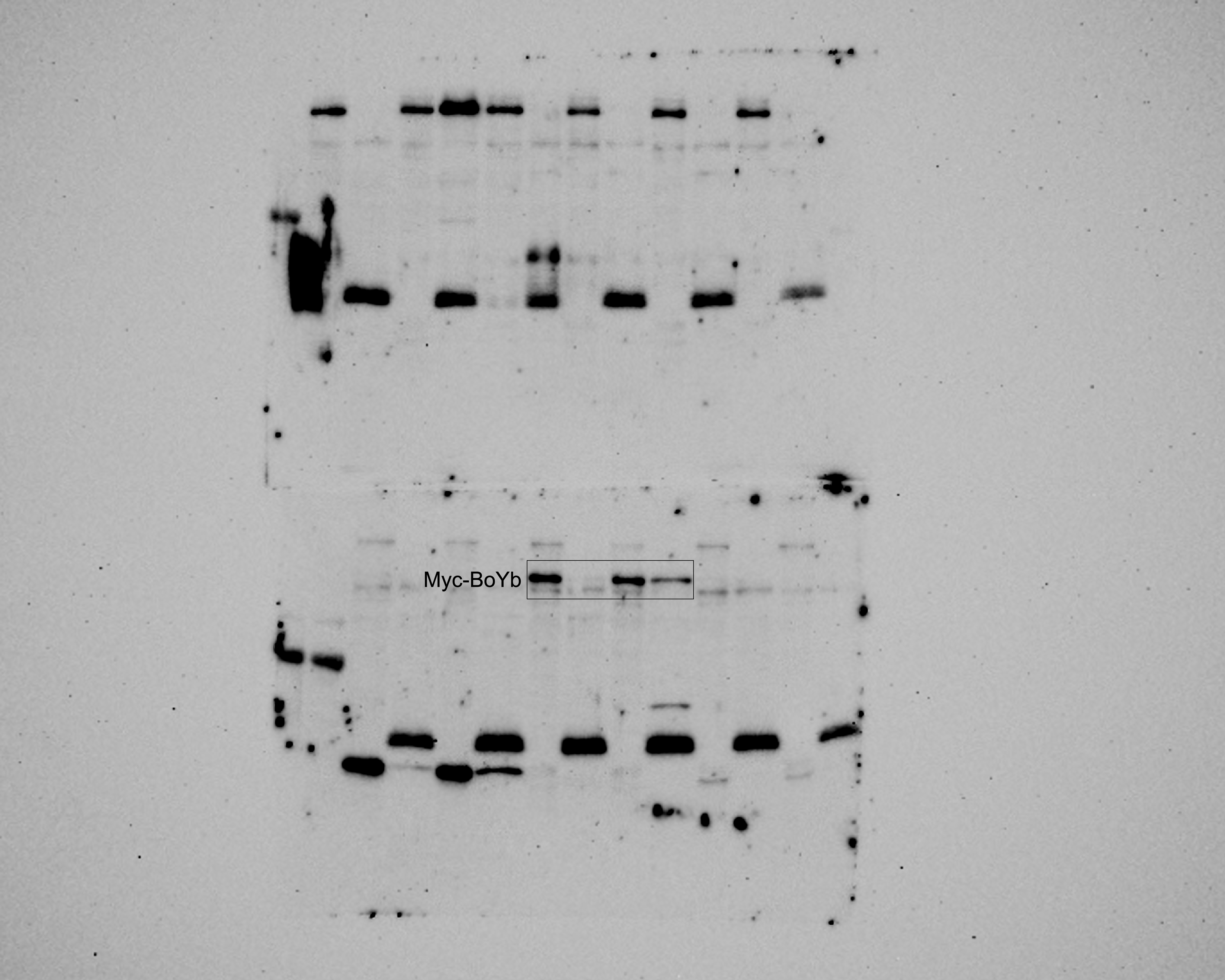

Supplement: Figure 1—source data 8. [file elife-101967-fig1-data8.zip › Figure 1-Source Data 8/Fig1D-iv_rep2_Myc_label_2023-08-17.tiff]

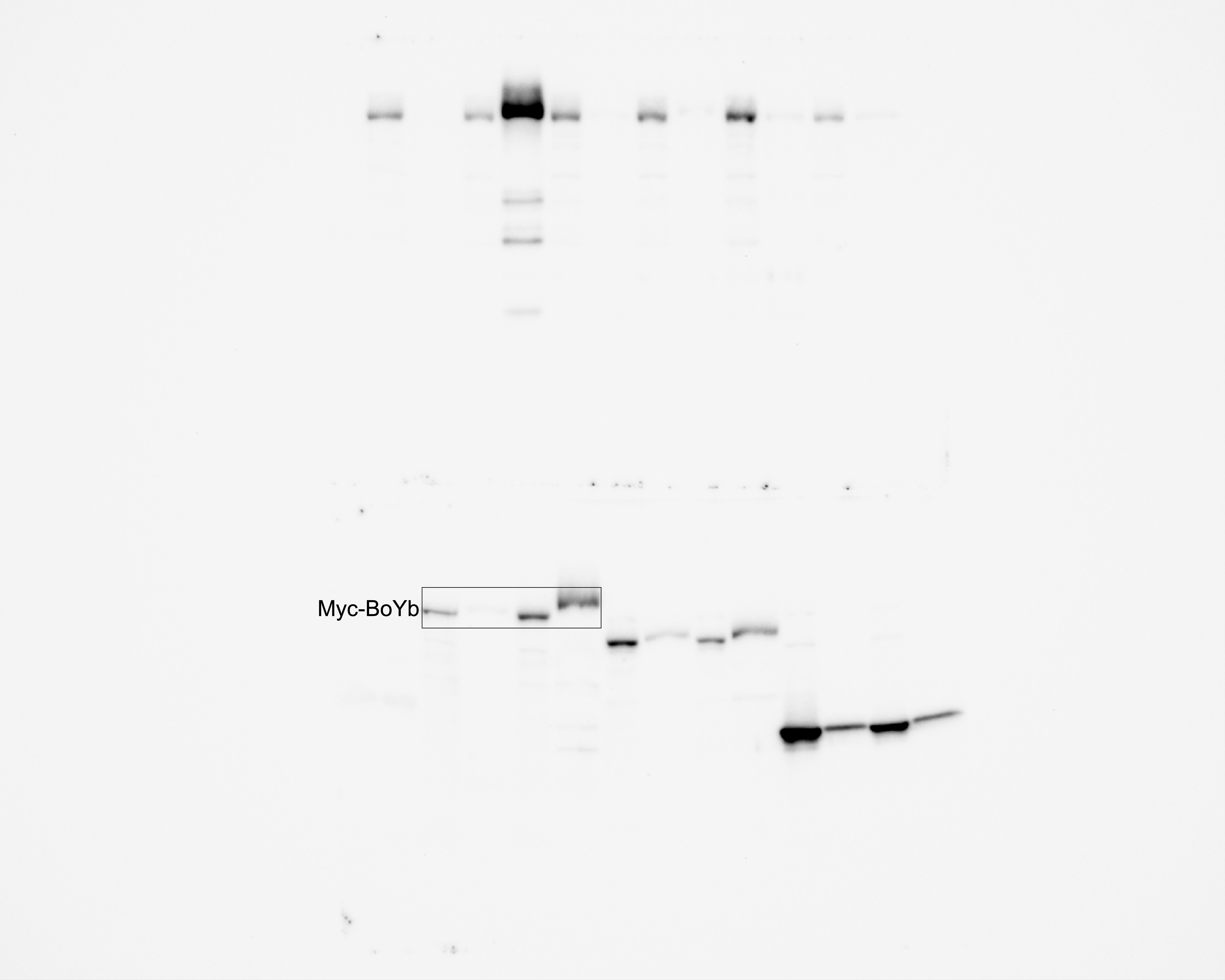

Supplement: Figure 1—source data 8. [file elife-101967-fig1-data8.zip › Figure 1-Source Data 8/Fig1D-iv_rep3_Myc_label_2023-08-25.tiff]

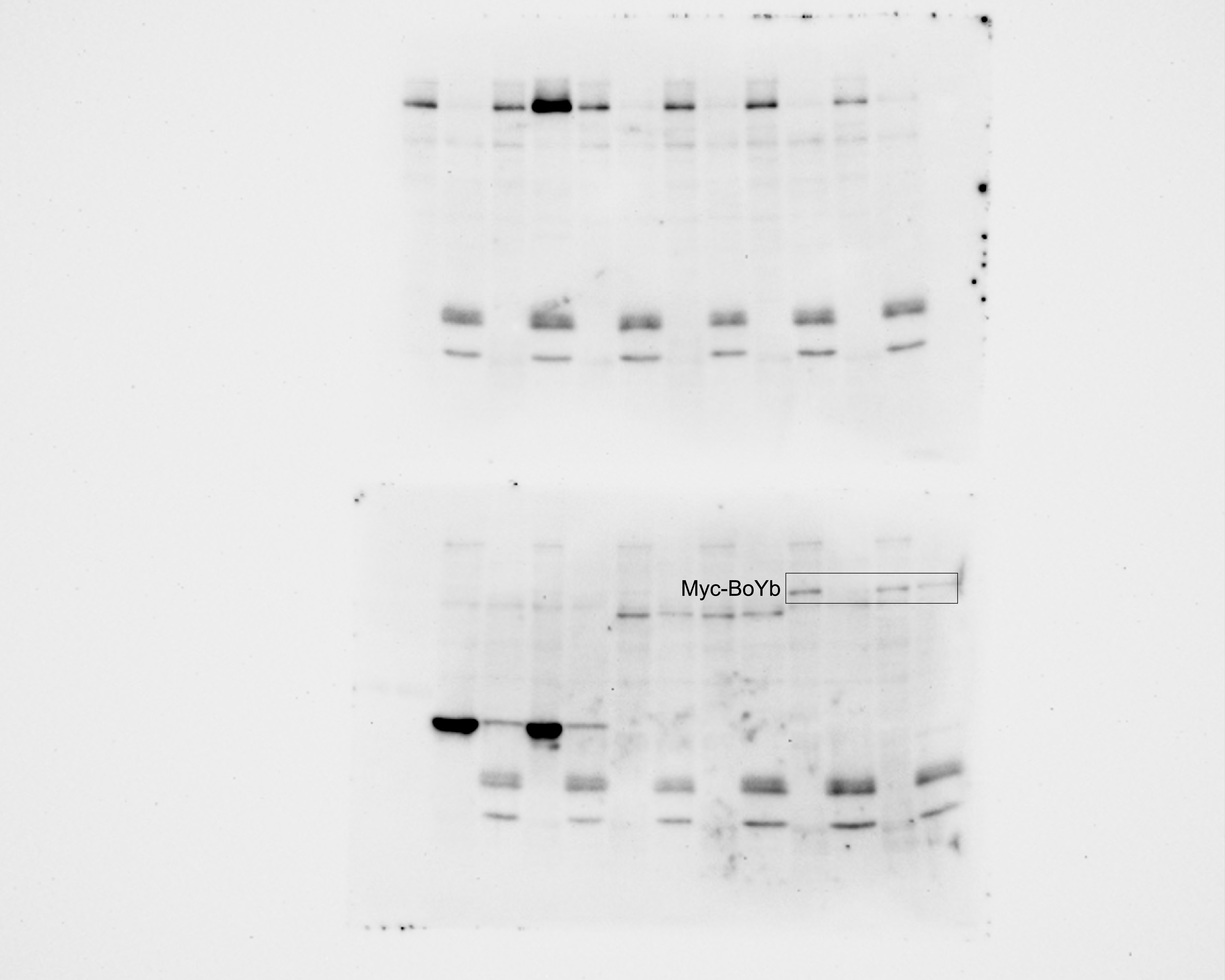

Supplement: Figure 1—source data 8. [file elife-101967-fig1-data8.zip › Figure 1-Source Data 8/Fig1D-iv_rep1_Myc_label_2023-08-01.tiff]

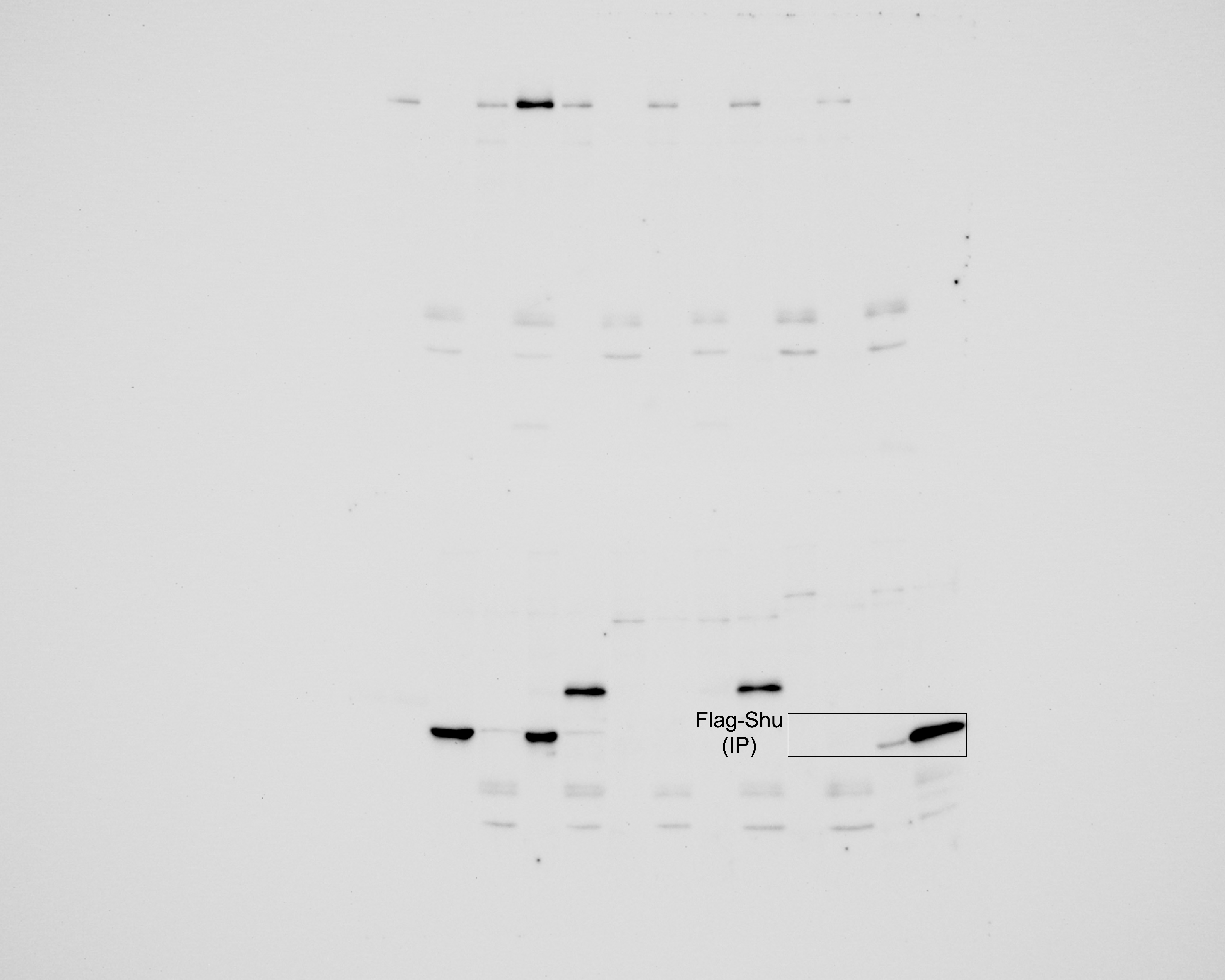

Supplement: Figure 1—source data 8. [file elife-101967-fig1-data8.zip › Figure 1-Source Data 8/Fig1D-iv_rep1_FLAG_label_2023-08-01.tiff]

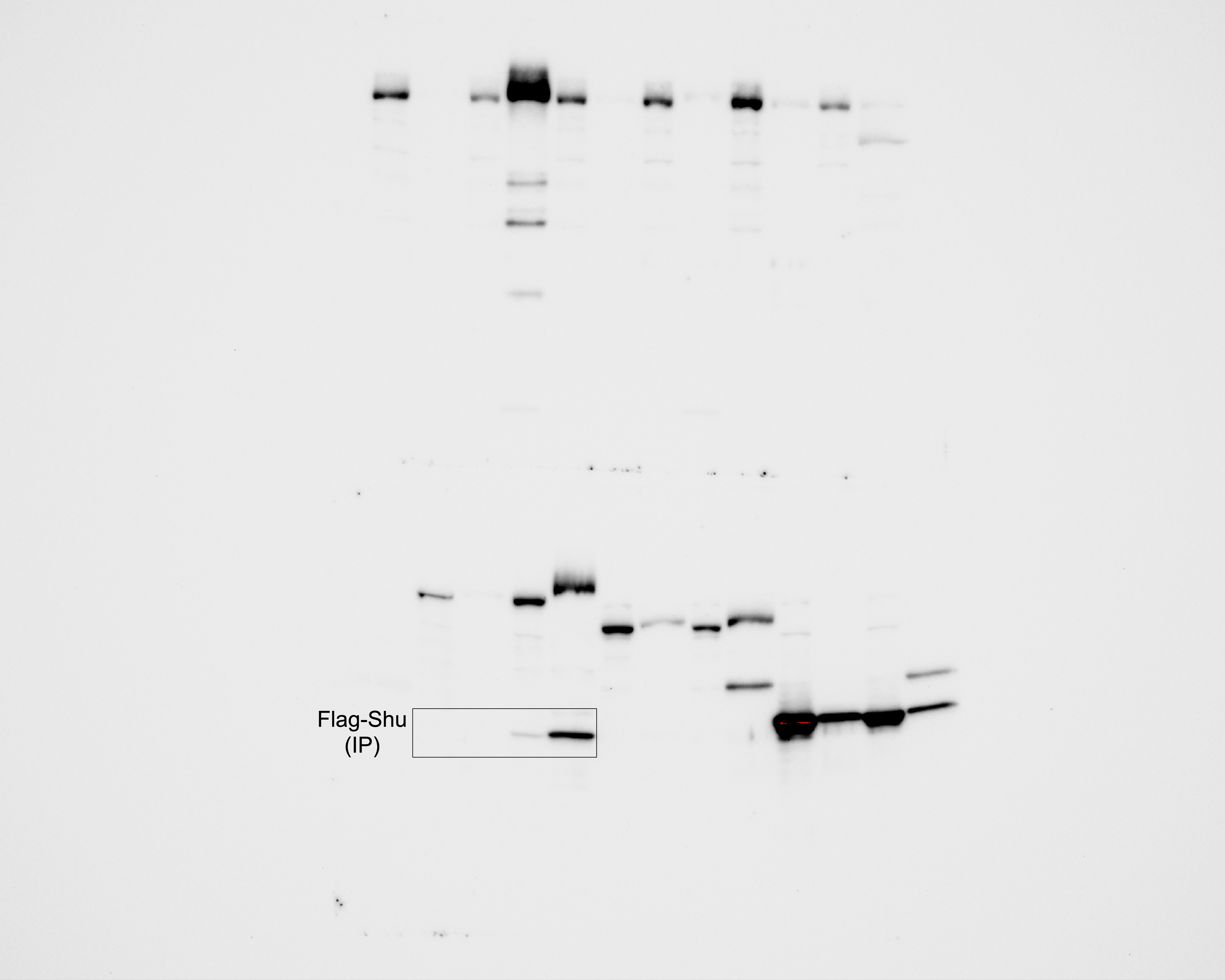

Supplement: Figure 1—source data 8. [file elife-101967-fig1-data8.zip › Figure 1-Source Data 8/Fig1D-iv_rep3_FLAG_label_2023-08-25.tiff]

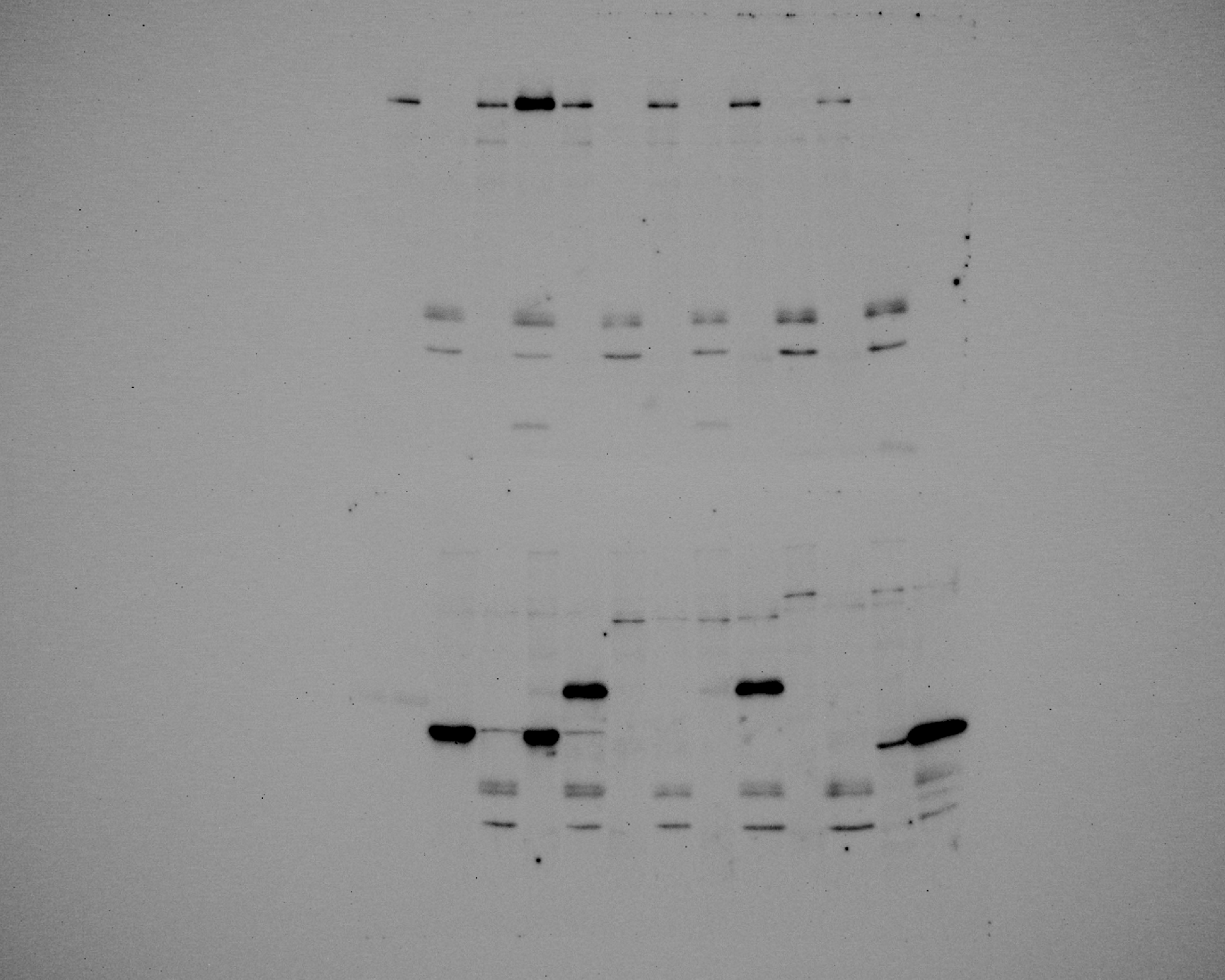

Supplement: Figure 1—source data 9. [file elife-101967-fig1-data9.zip › Figure 1-Source Data 9/Fig1D-iv_rep1_FLAG_original_2023-08-01.tif]

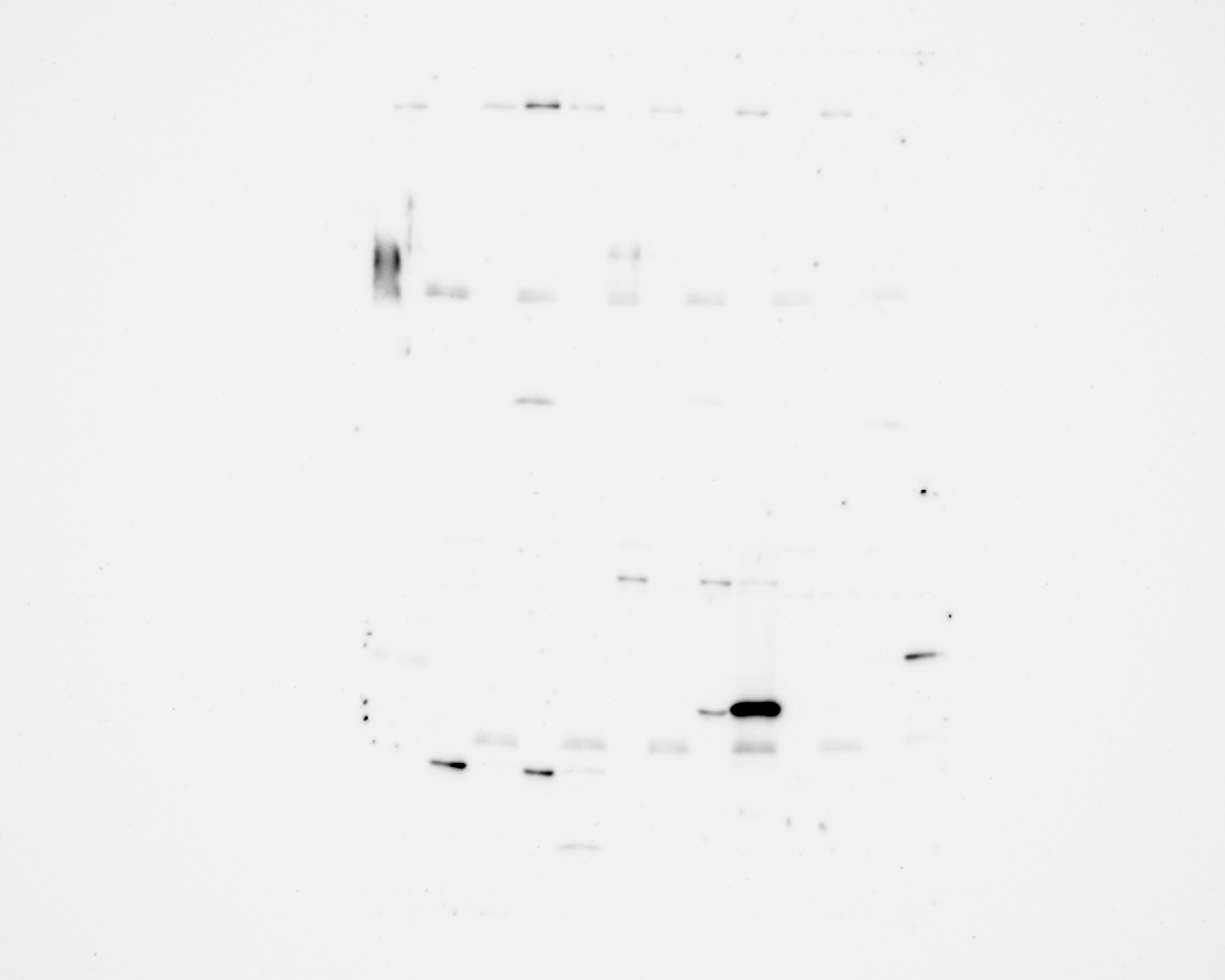

Supplement: Figure 1—source data 9. [file elife-101967-fig1-data9.zip › Figure 1-Source Data 9/Fig1D-iv_rep2_FLAG_original_2023-08-17.tif]

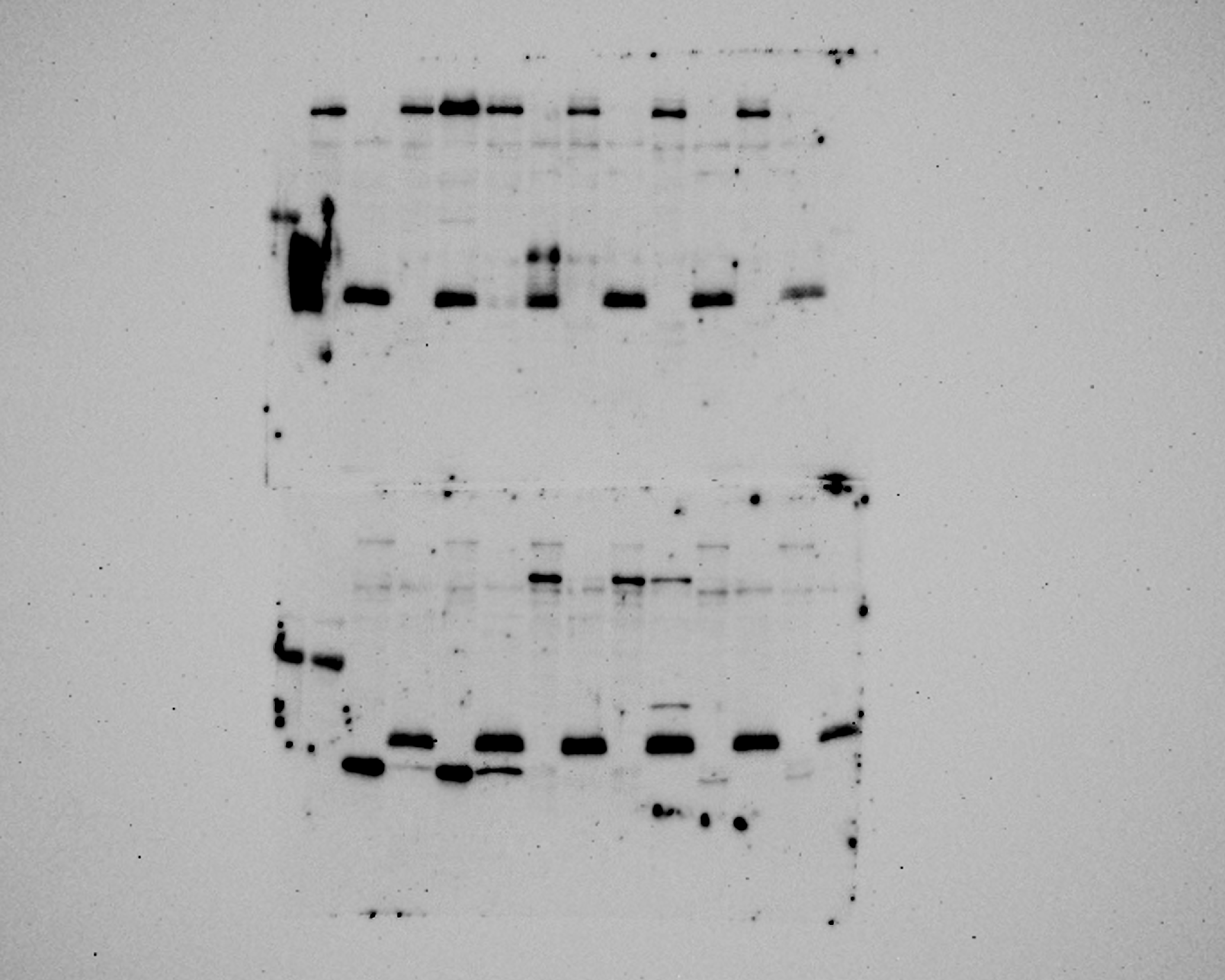

Supplement: Figure 1—source data 9. [file elife-101967-fig1-data9.zip › Figure 1-Source Data 9/Fig1D-iv_rep2_Myc_original_2023-08-17.tif]

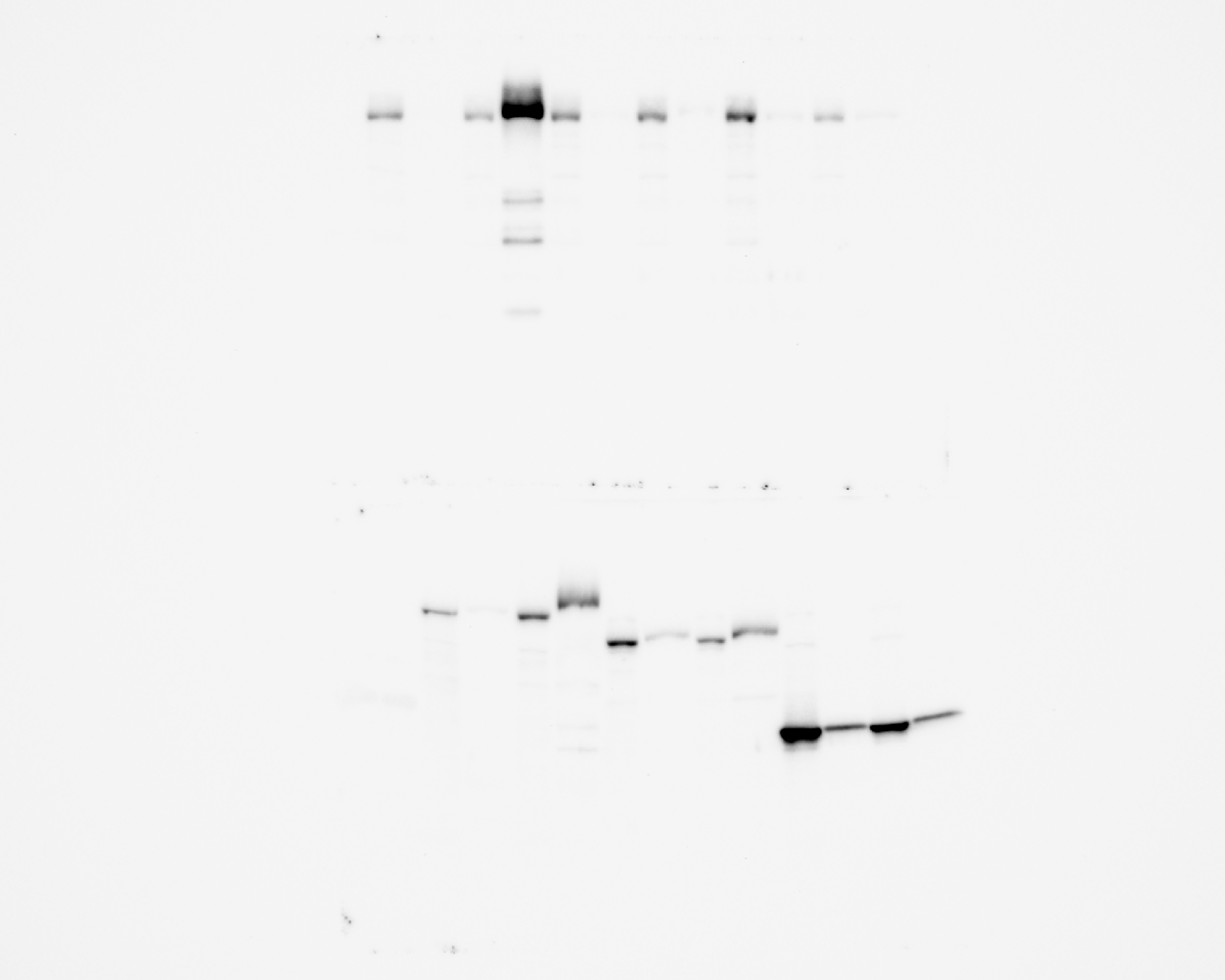

Supplement: Figure 1—source data 9. [file elife-101967-fig1-data9.zip › Figure 1-Source Data 9/Fig1D-iv_rep3_Myc_original_2023-08-25.tif]

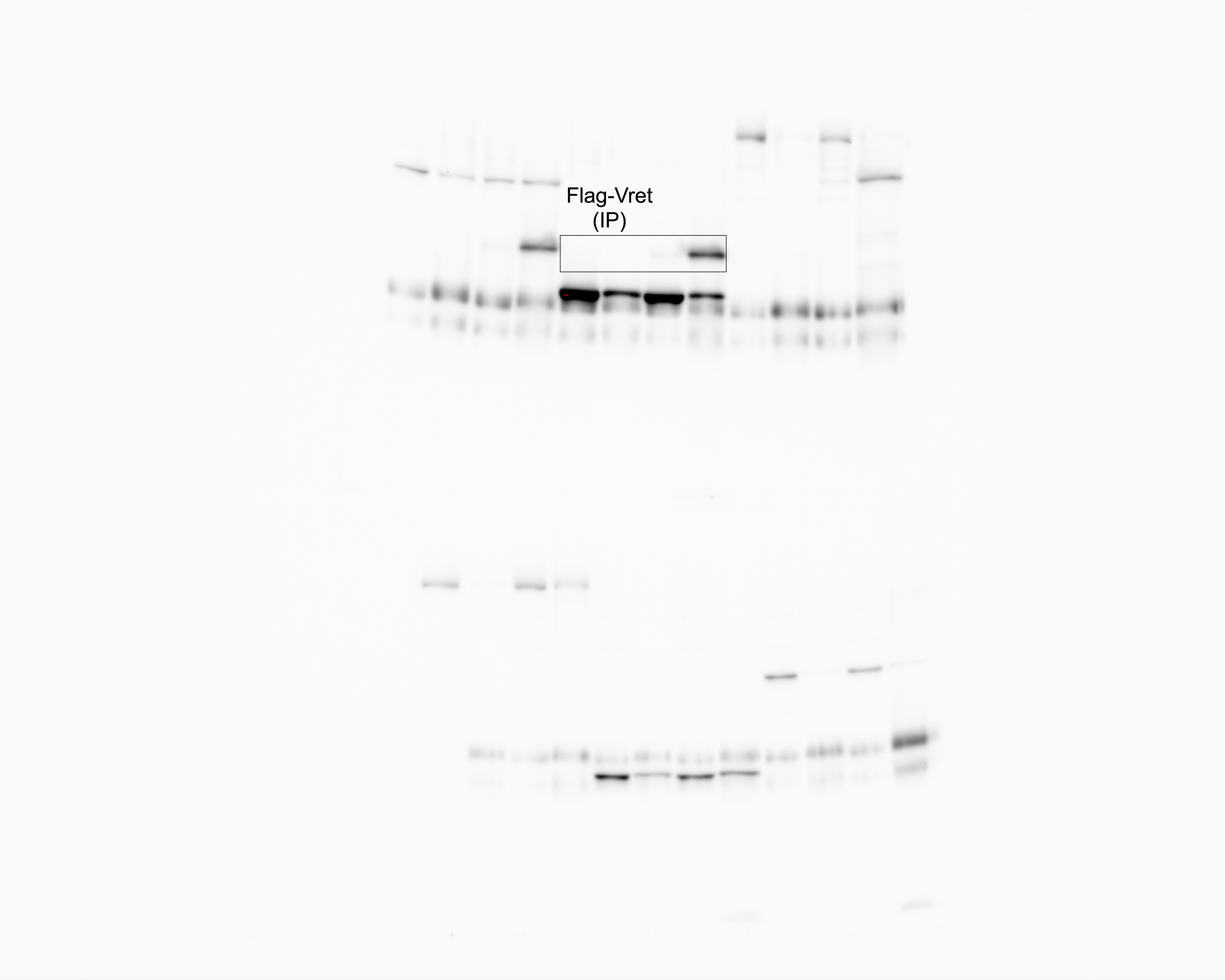

Supplement: Figure 1—source data 10. [file elife-101967-fig1-data10.zip › Figure 1-Source Data 10/Fig1D-v_rep3_FLAG_label_2023-09-01.tiff]

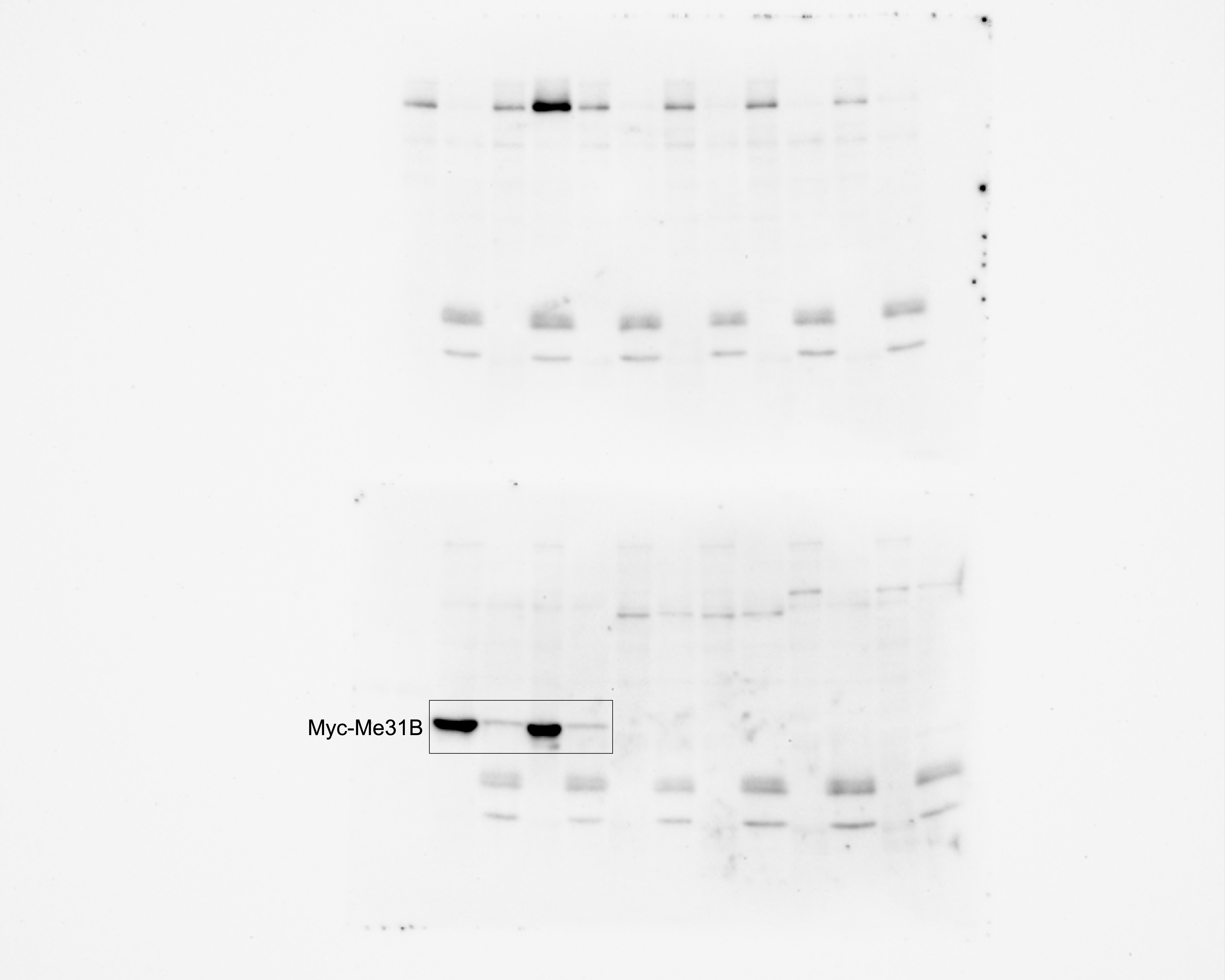

Supplement: Figure 1—source data 10. [file elife-101967-fig1-data10.zip › Figure 1-Source Data 10/Fig1D-v_rep1_Myc_label_2023-08-01.tiff]

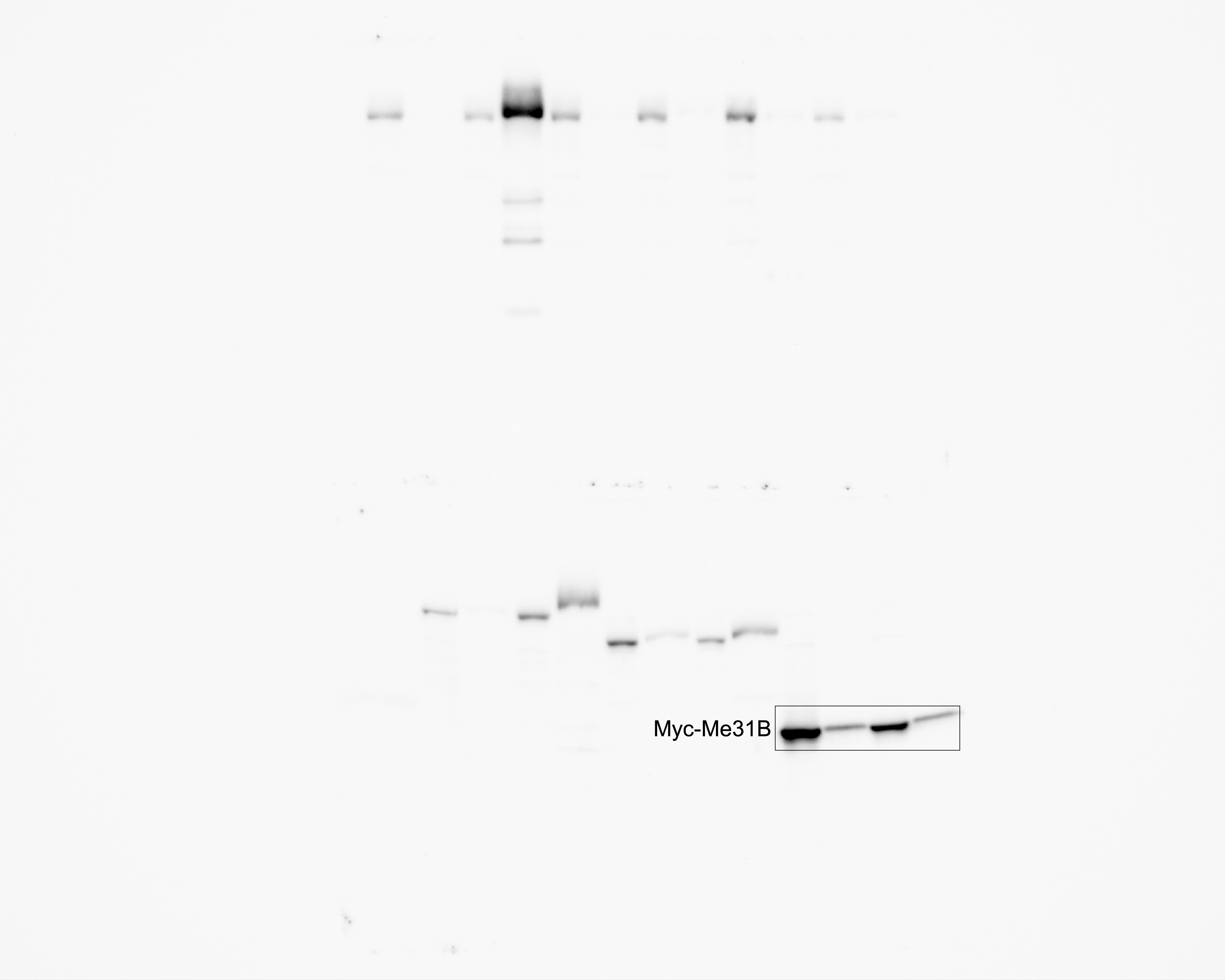

Supplement: Figure 1—source data 10. [file elife-101967-fig1-data10.zip › Figure 1-Source Data 10/Fig1D-v_rep2_Myc_label_2023-08-25.tiff]

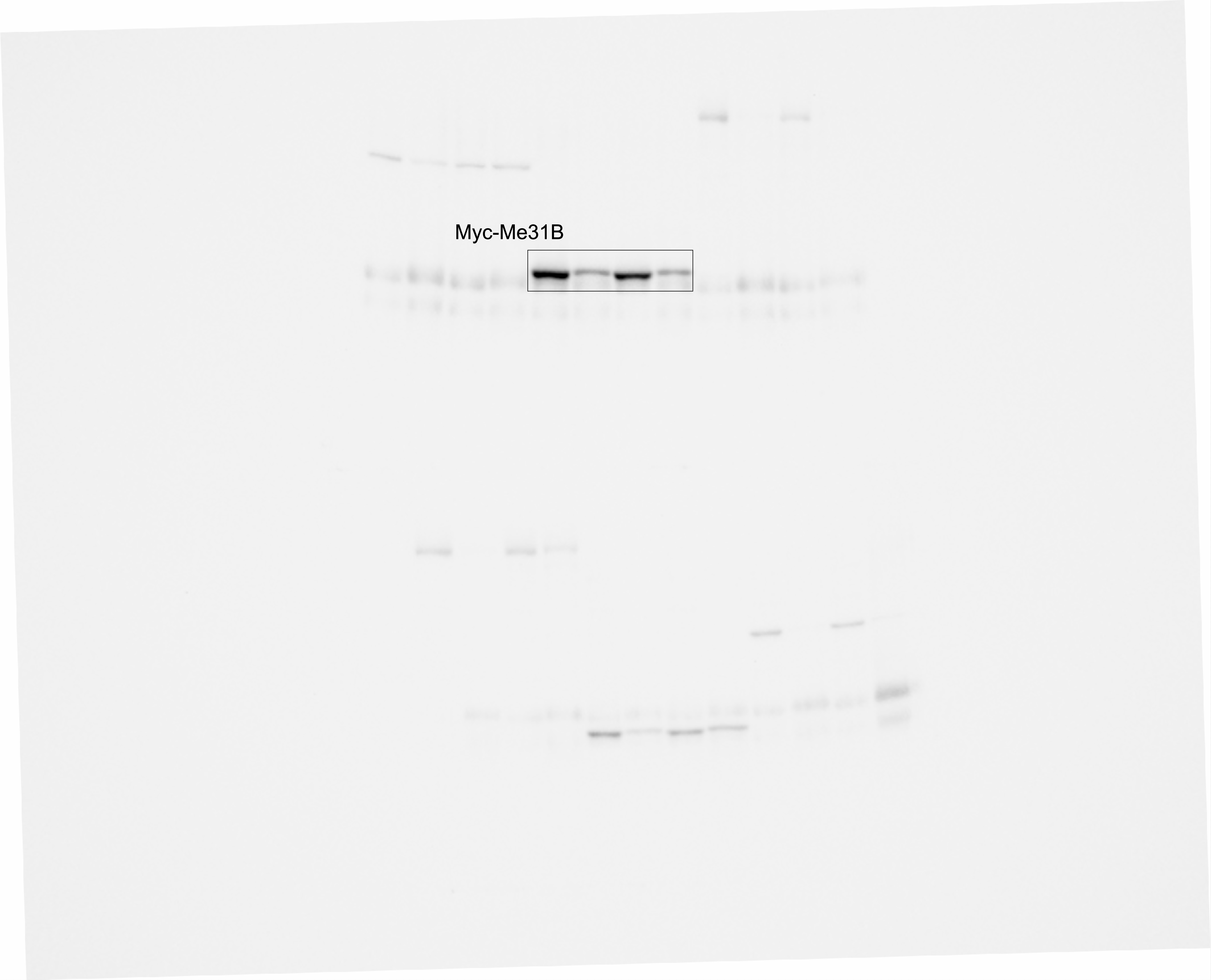

Supplement: Figure 1—source data 10. [file elife-101967-fig1-data10.zip › Figure 1-Source Data 10/Fig1D-v_rep3_Myc_label_2023-09-01.tiff]

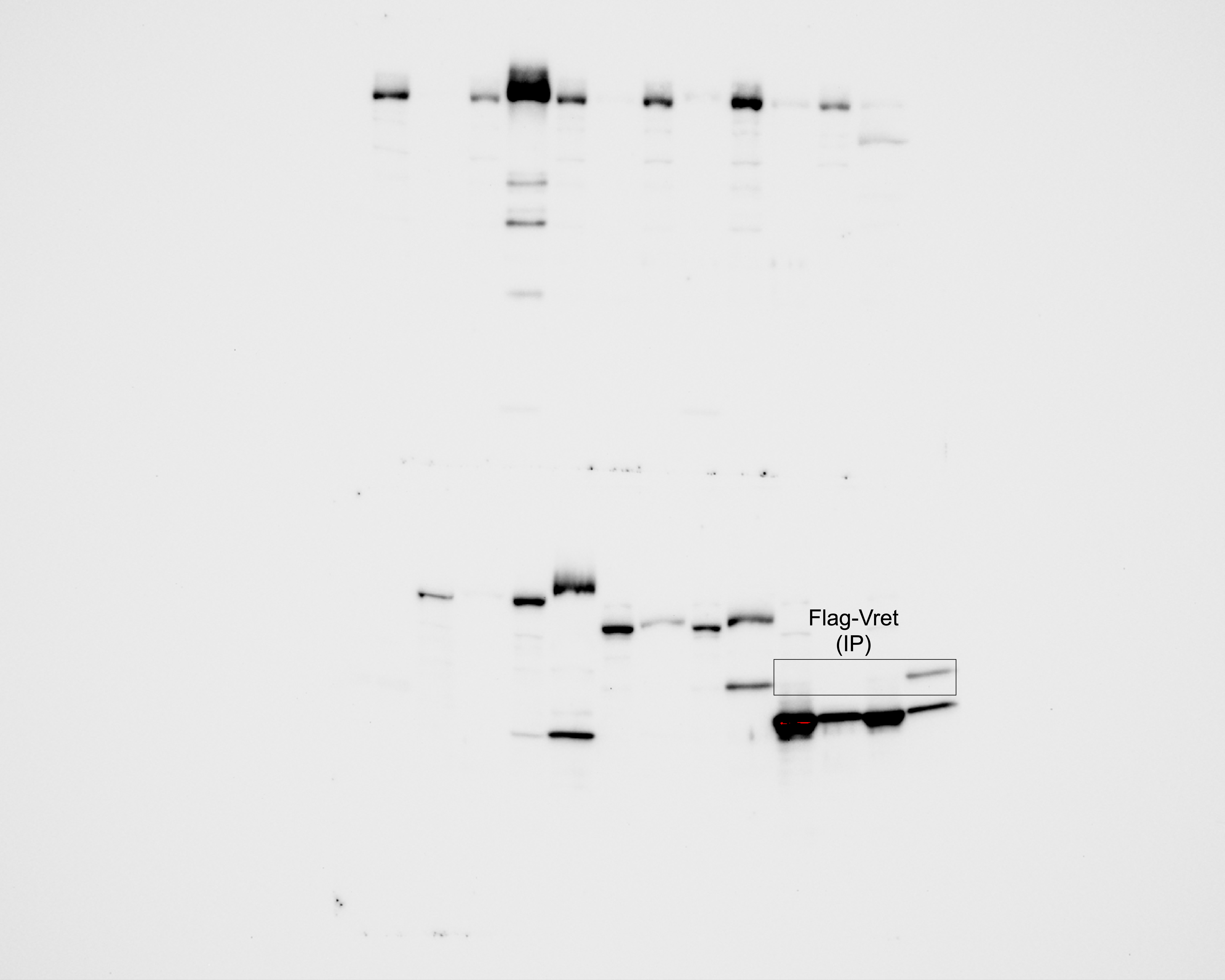

Supplement: Figure 1—source data 10. [file elife-101967-fig1-data10.zip › Figure 1-Source Data 10/Fig1D-v_rep2_FLAG_label_2023-08-25.tiff]

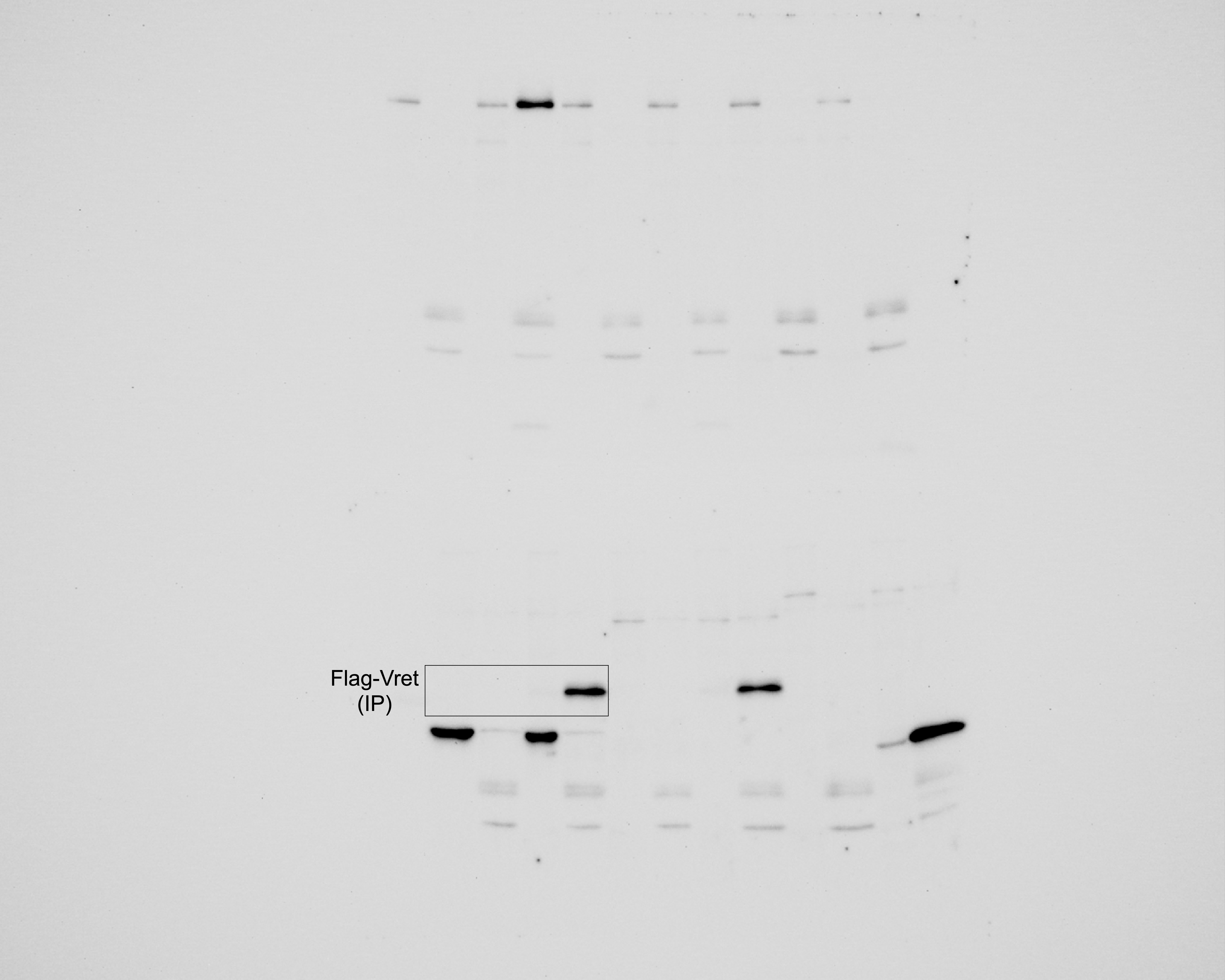

Supplement: Figure 1—source data 10. [file elife-101967-fig1-data10.zip › Figure 1-Source Data 10/Fig1D-v_rep1_FLAG_label_2023-08-01.tiff]

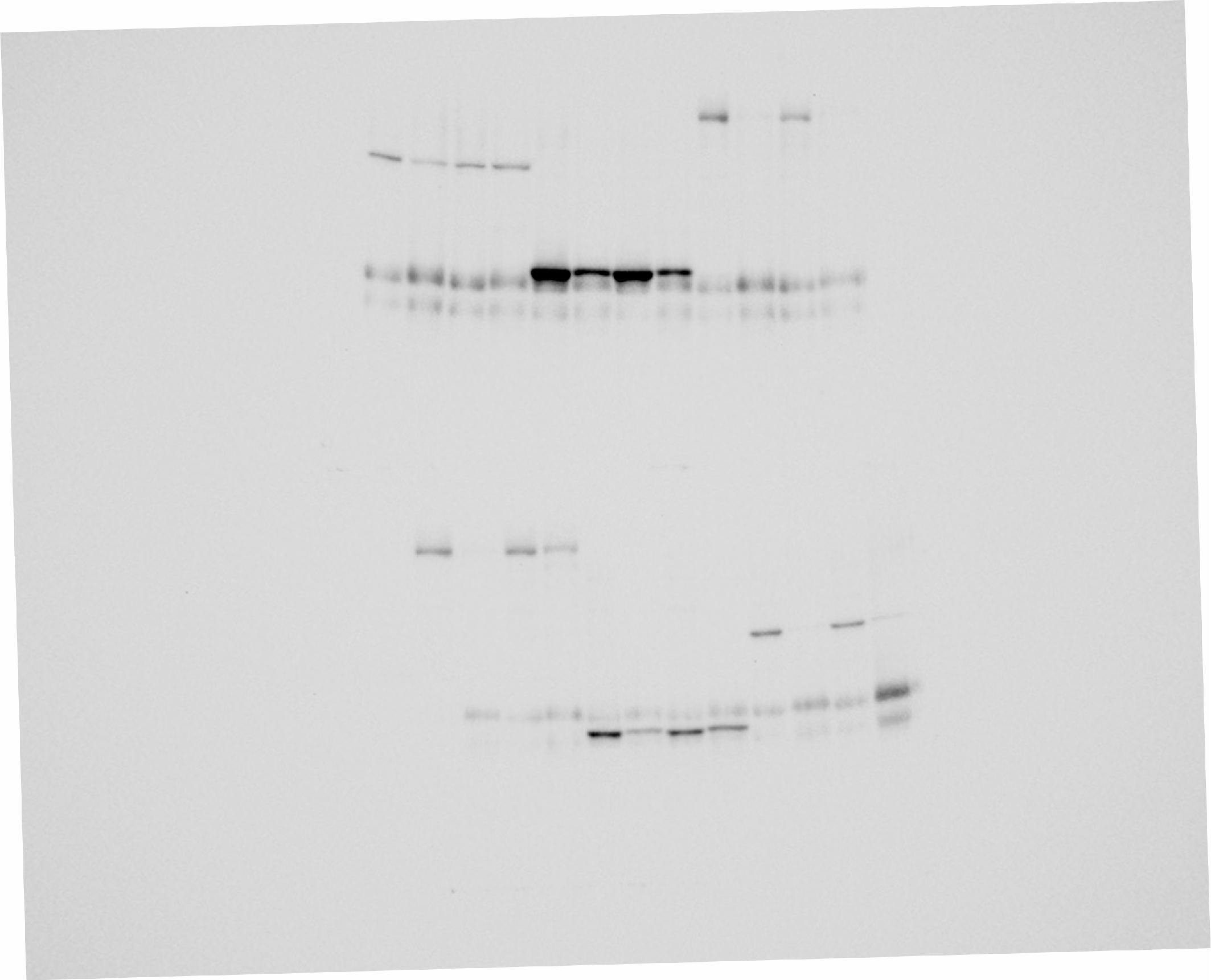

Supplement: Figure 1—source data 11. [file elife-101967-fig1-data11.zip › Figure 1-Source Data 11/Fig1D-v_rep3_Myc_original_2023-09-01.tif]

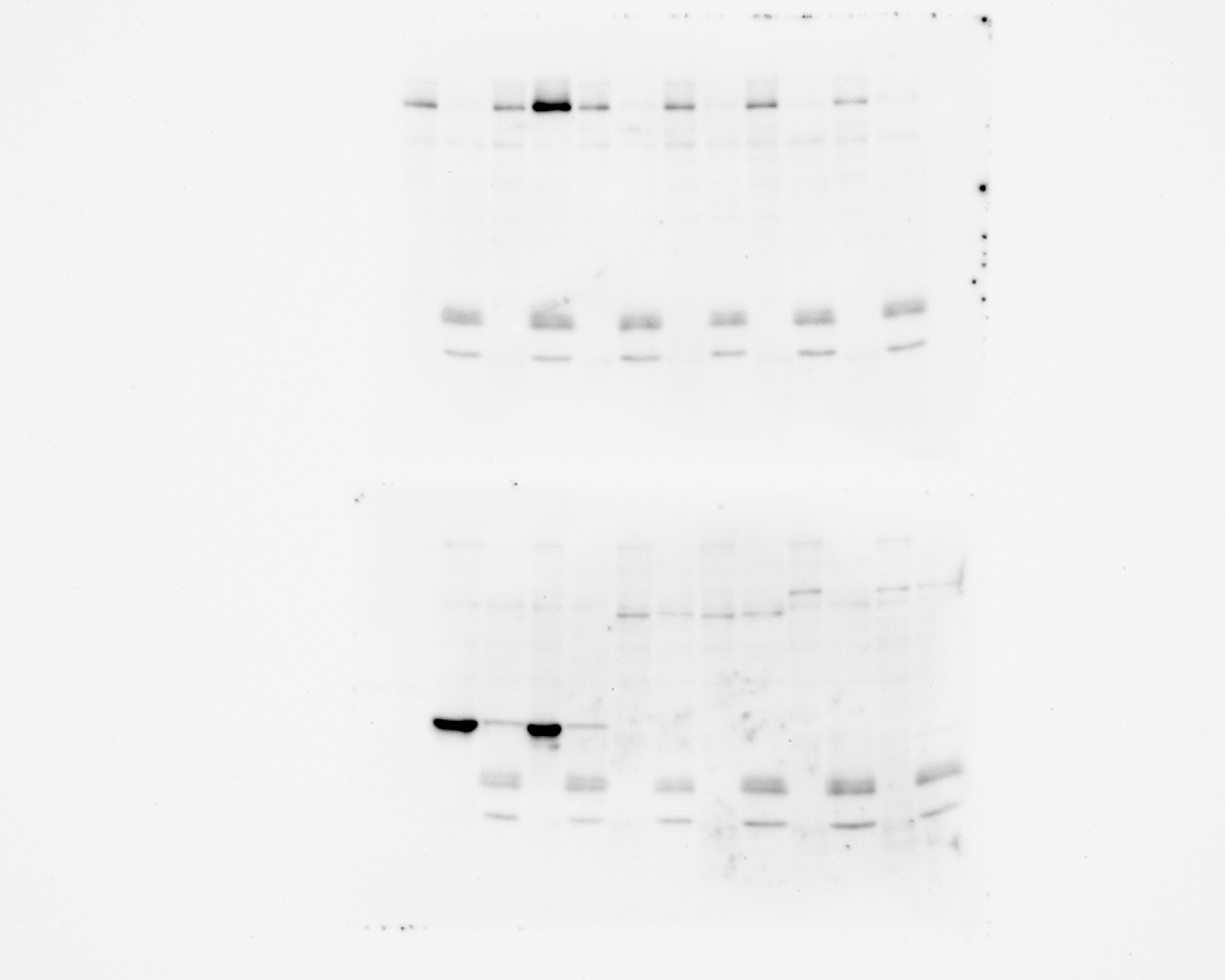

Supplement: Figure 1—source data 11. [file elife-101967-fig1-data11.zip › Figure 1-Source Data 11/Fig1D-v_rep1_Myc_original_2023-08-01.tif]

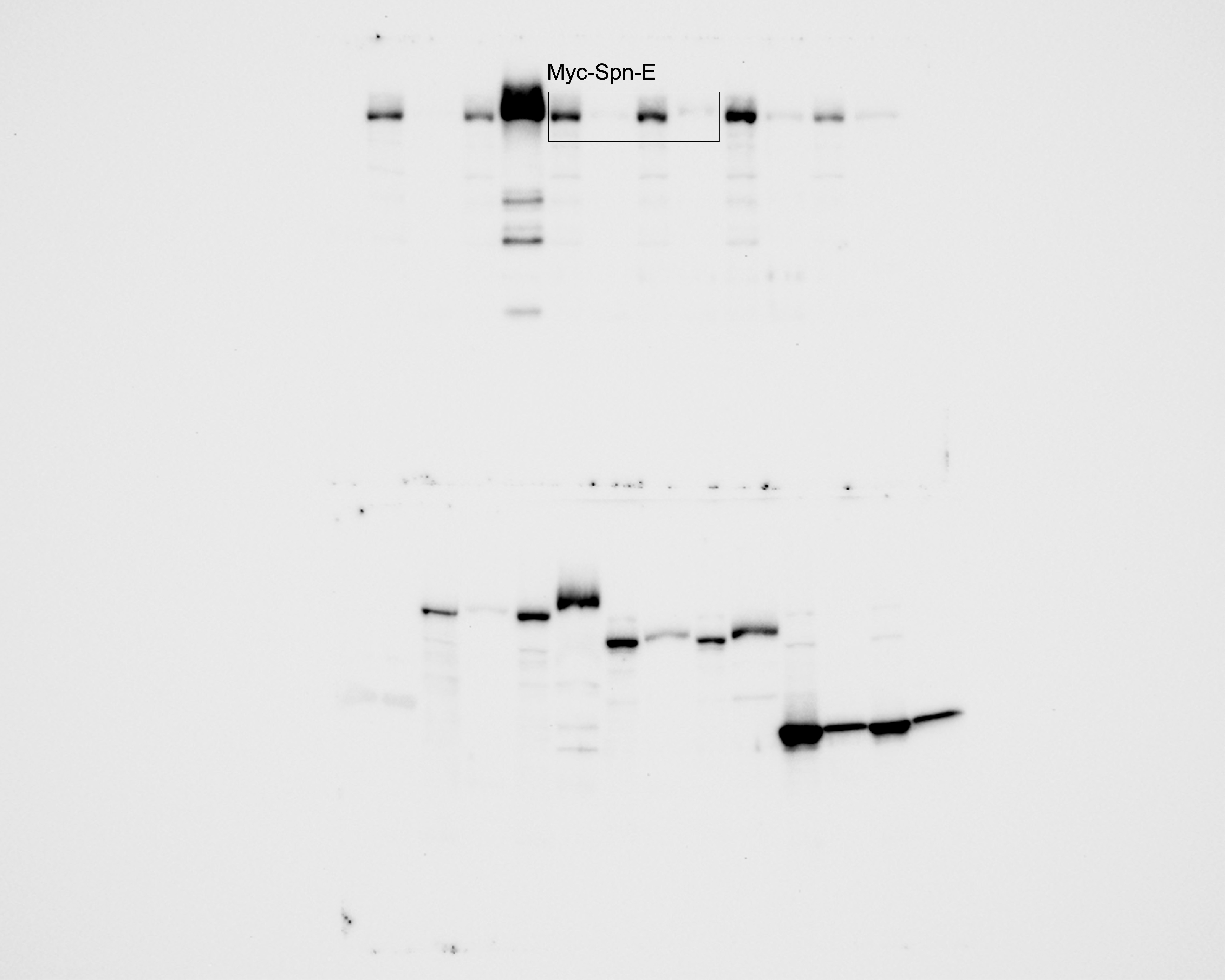

Supplement: Figure 2—source data 2. [file elife-101967-fig2-data2.zip › Figure 2-Source Data 2/Fig2E_rep3_Myc_label_2023-08-25.tiff]

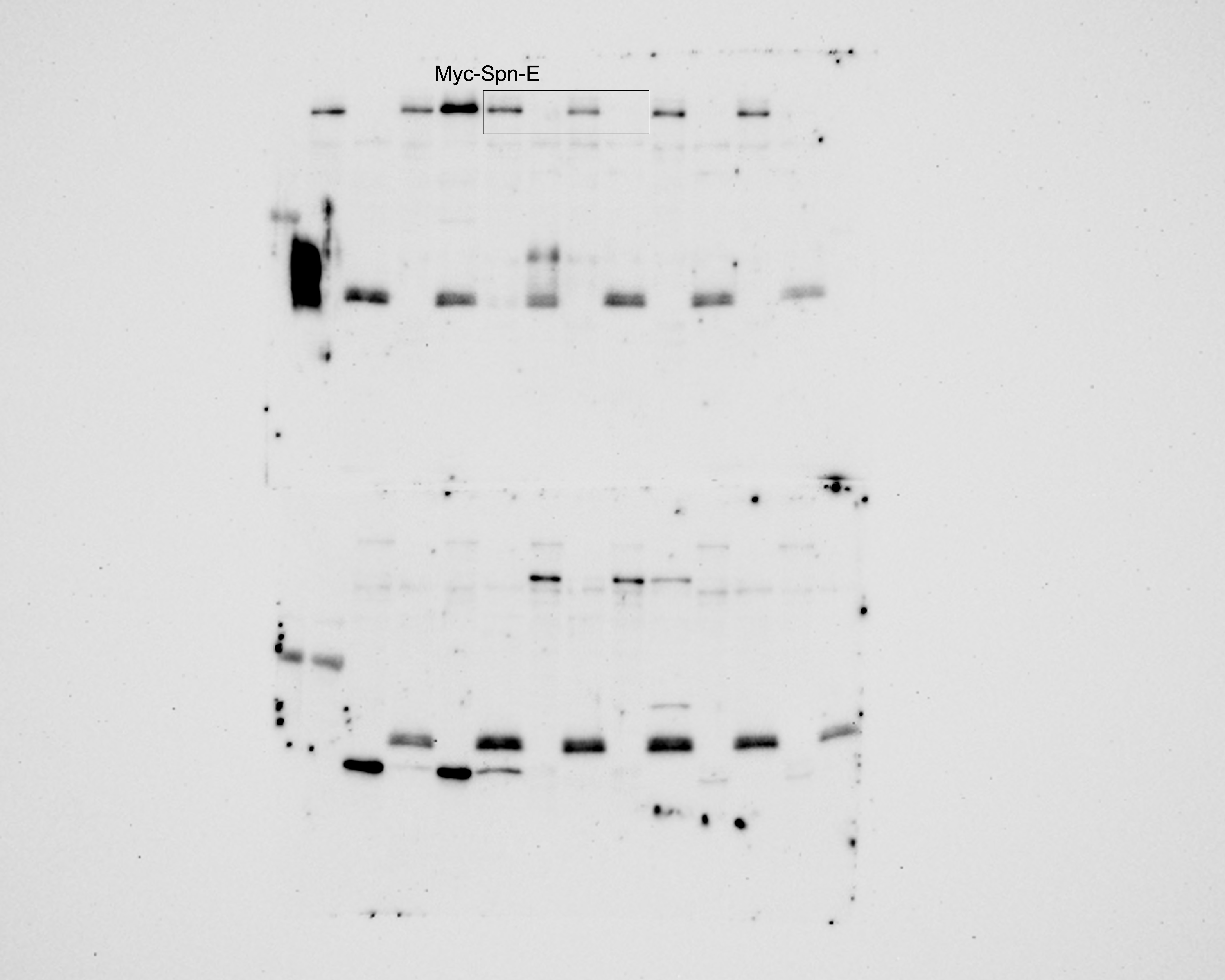

Supplement: Figure 2—source data 2. [file elife-101967-fig2-data2.zip › Figure 2-Source Data 2/Fig2E_rep2_Myc_label_2023-08-17.tiff]

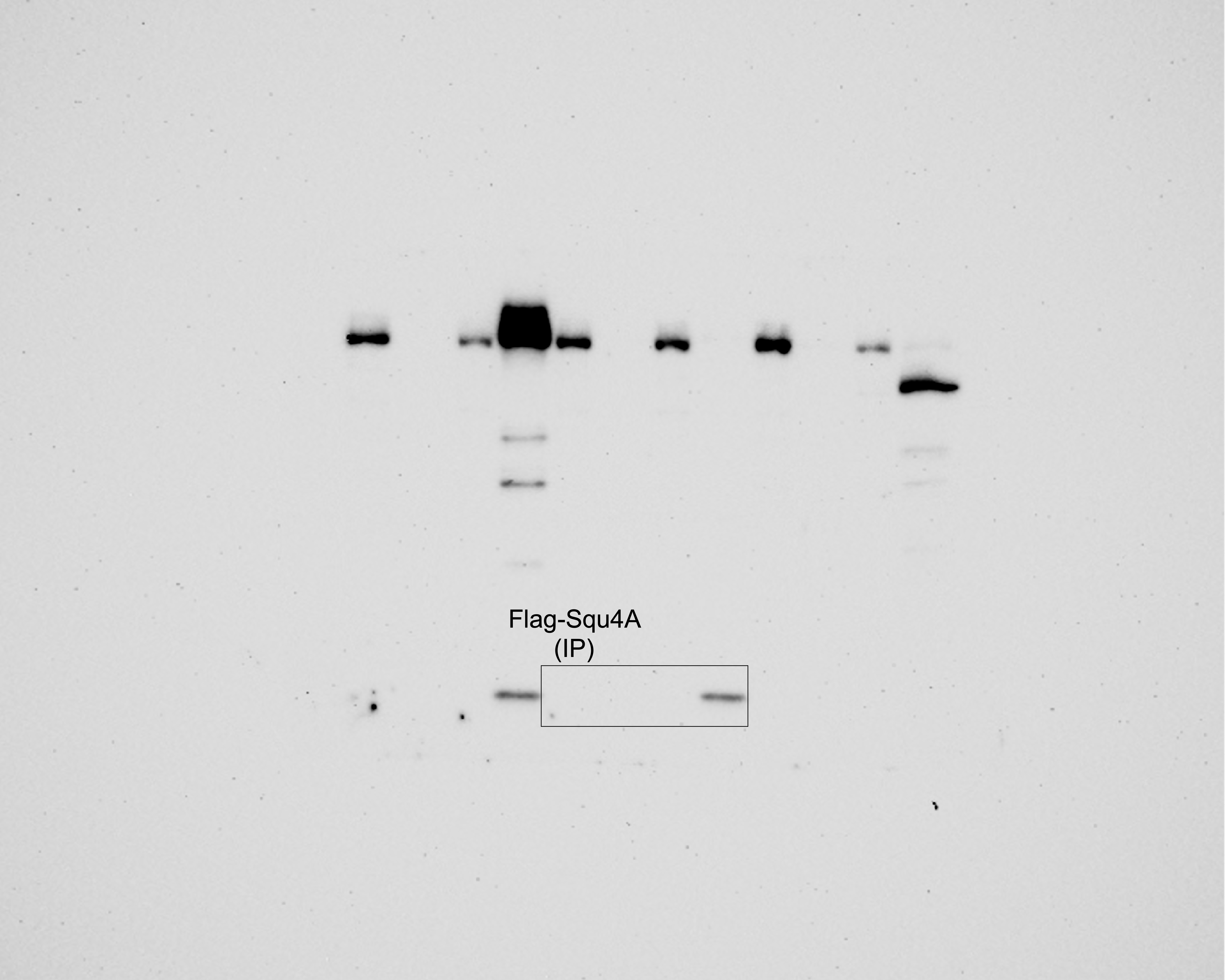

Supplement: Figure 2—source data 2. [file elife-101967-fig2-data2.zip › Figure 2-Source Data 2/Fig2E_rep3_FLAG_label_2023-08-25.tiff]

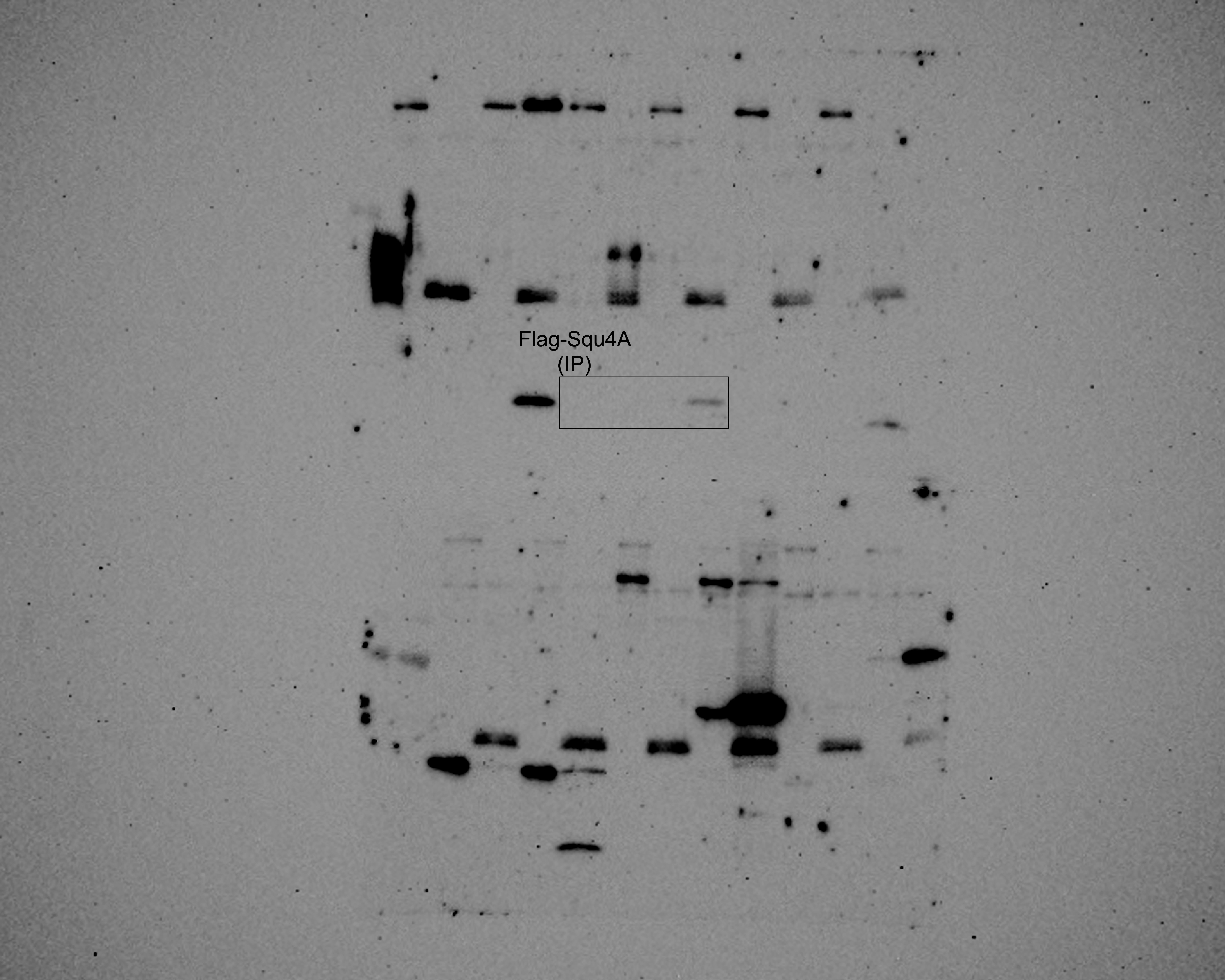

Supplement: Figure 2—source data 2. [file elife-101967-fig2-data2.zip › Figure 2-Source Data 2/Fig2E_rep2_FLAG_label_2023-08-17.tiff]

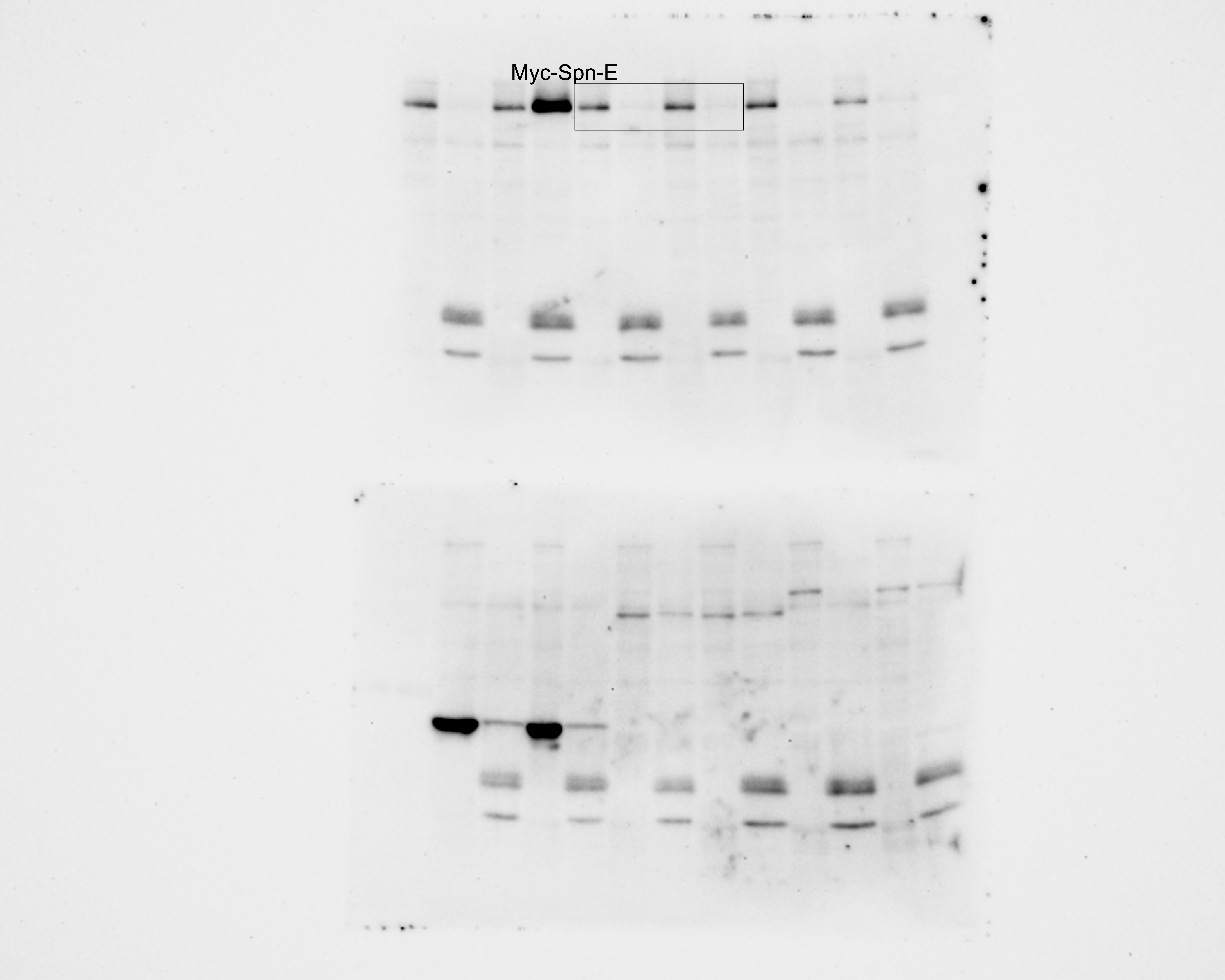

Supplement: Figure 2—source data 2. [file elife-101967-fig2-data2.zip › Figure 2-Source Data 2/Fig2E_rep1_Myc_label_2023-08-01.tiff]

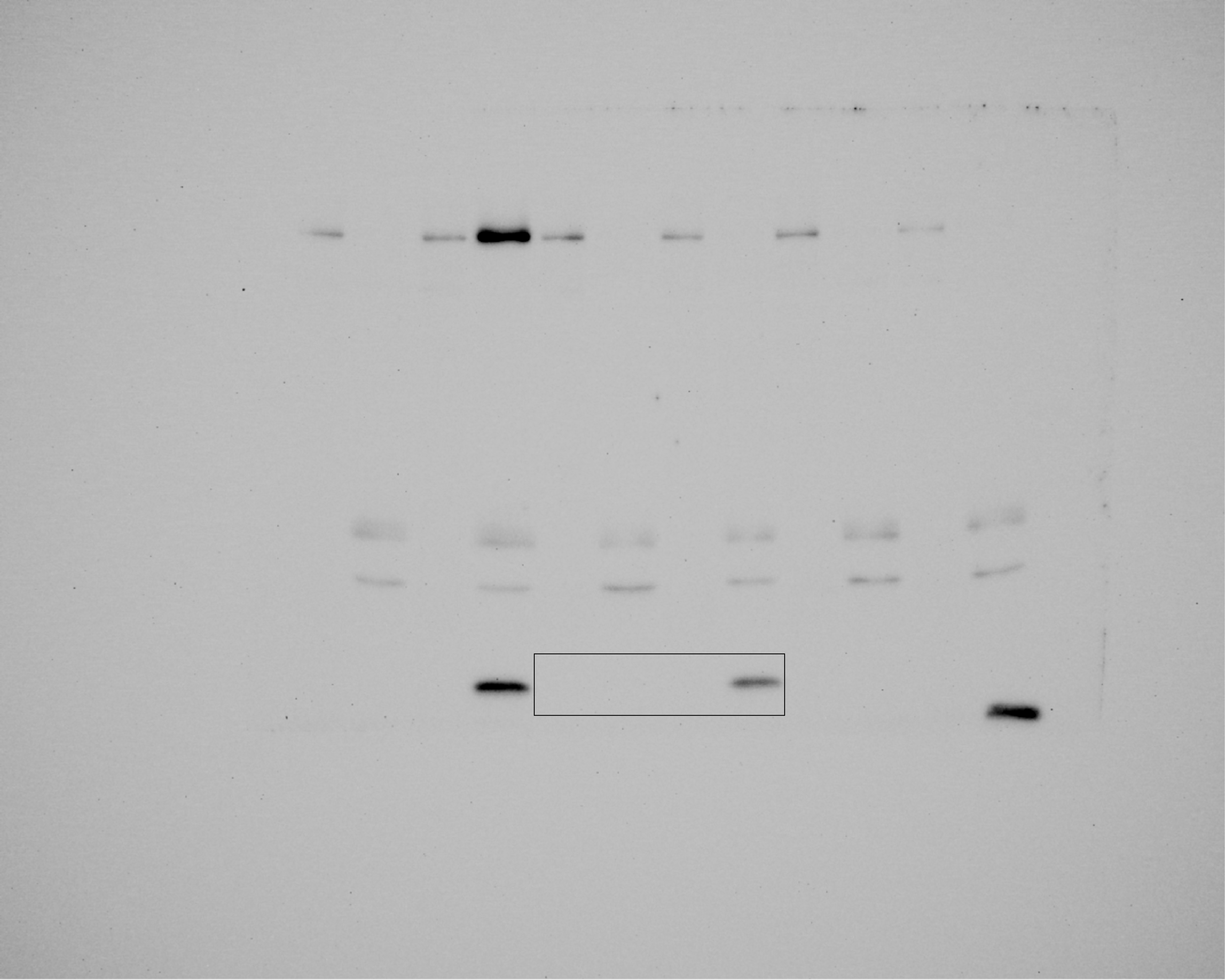

Supplement: Figure 2—source data 2. [file elife-101967-fig2-data2.zip › Figure 2-Source Data 2/Fig2E_rep1_FLAG_label_2023-08-02.tiff]

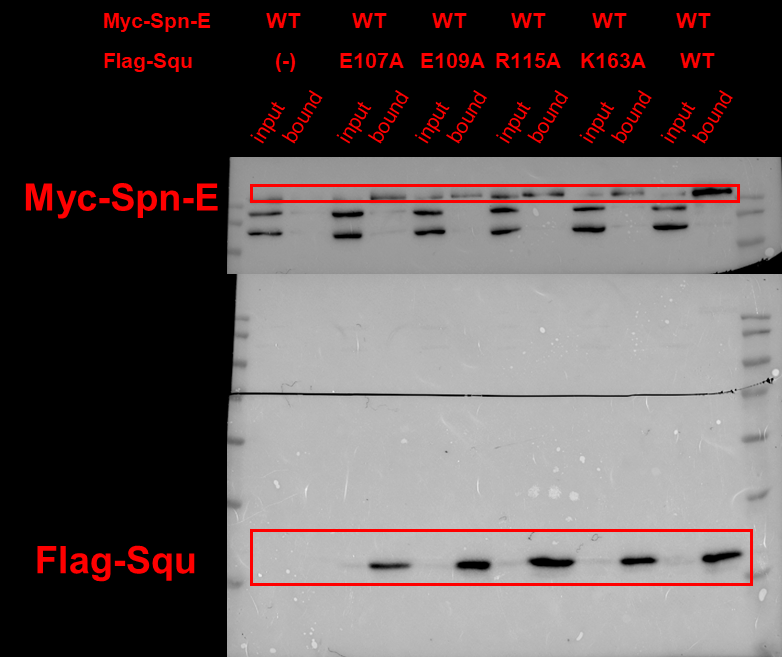

Supplement: Figure 2—figure supplement 2—source data 1. [file elife-101967-fig2-figsupp2-data1.zip › Figure 2-figure supplement 2-Source Data 1/Flag-Squ and Myc-SpnE rep3 with label in manuscript.tif]

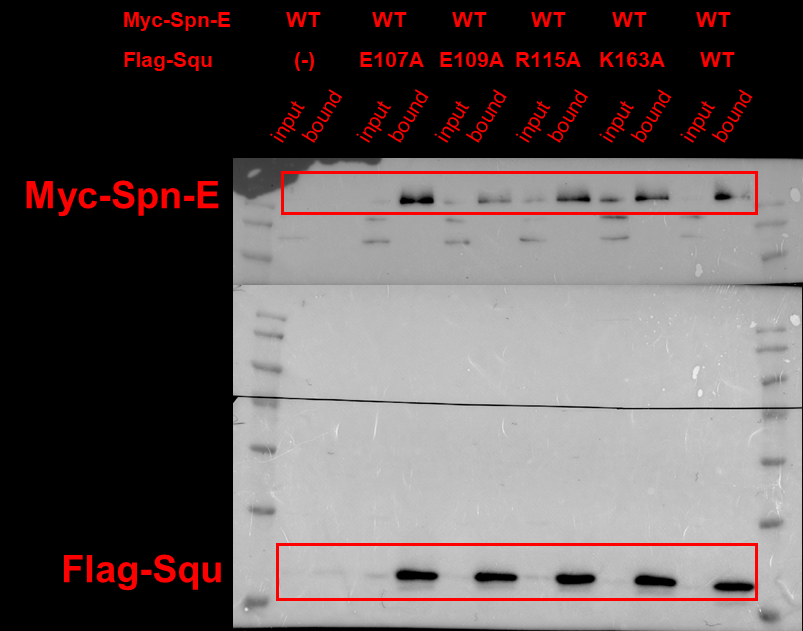

Supplement: Figure 2—figure supplement 2—source data 1. [file elife-101967-fig2-figsupp2-data1.zip › Figure 2-figure supplement 2-Source Data 1/Flag-Squ and Myc-SpnE rep2 with label.tif]

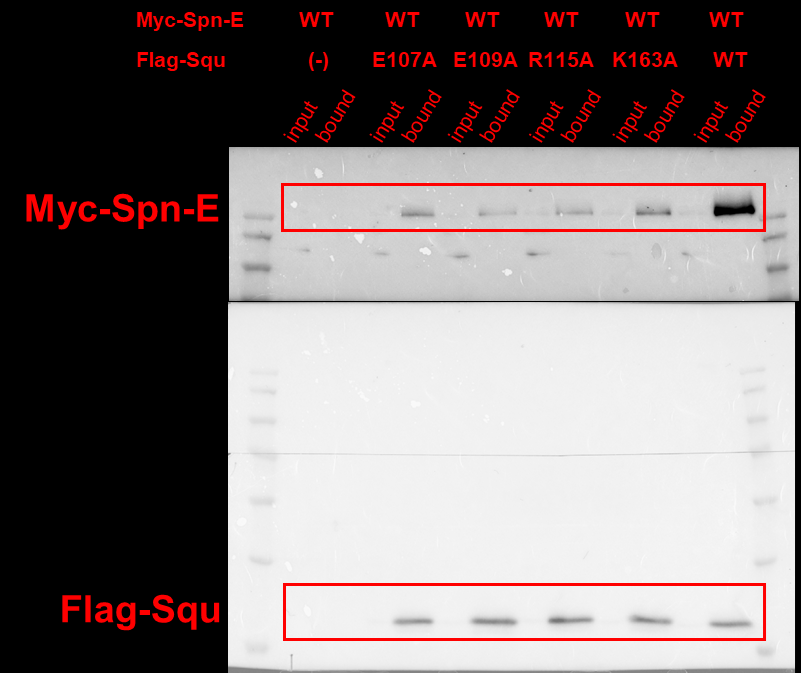

Supplement: Figure 2—figure supplement 2—source data 1. [file elife-101967-fig2-figsupp2-data1.zip › Figure 2-figure supplement 2-Source Data 1/Flag-Squ and Myc-SpnE rep1 with label.tif]

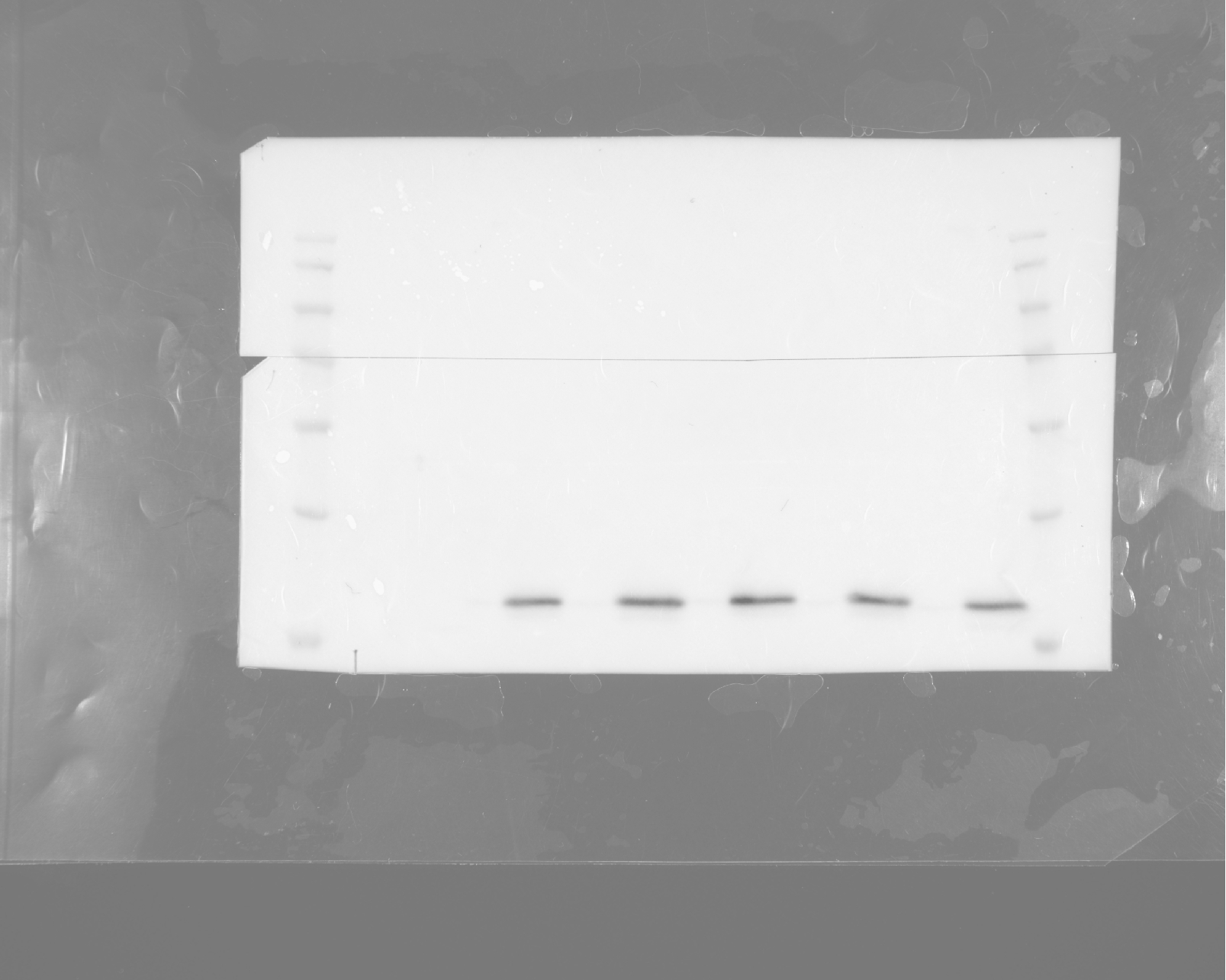

Supplement: Figure 2—figure supplement 2—source data 2. [file elife-101967-fig2-figsupp2-data2.zip › Figure 2-figure supplement 2-Source Data 2/Flag-Squ and Myc-SpnE anti-Flag rep1.tif]

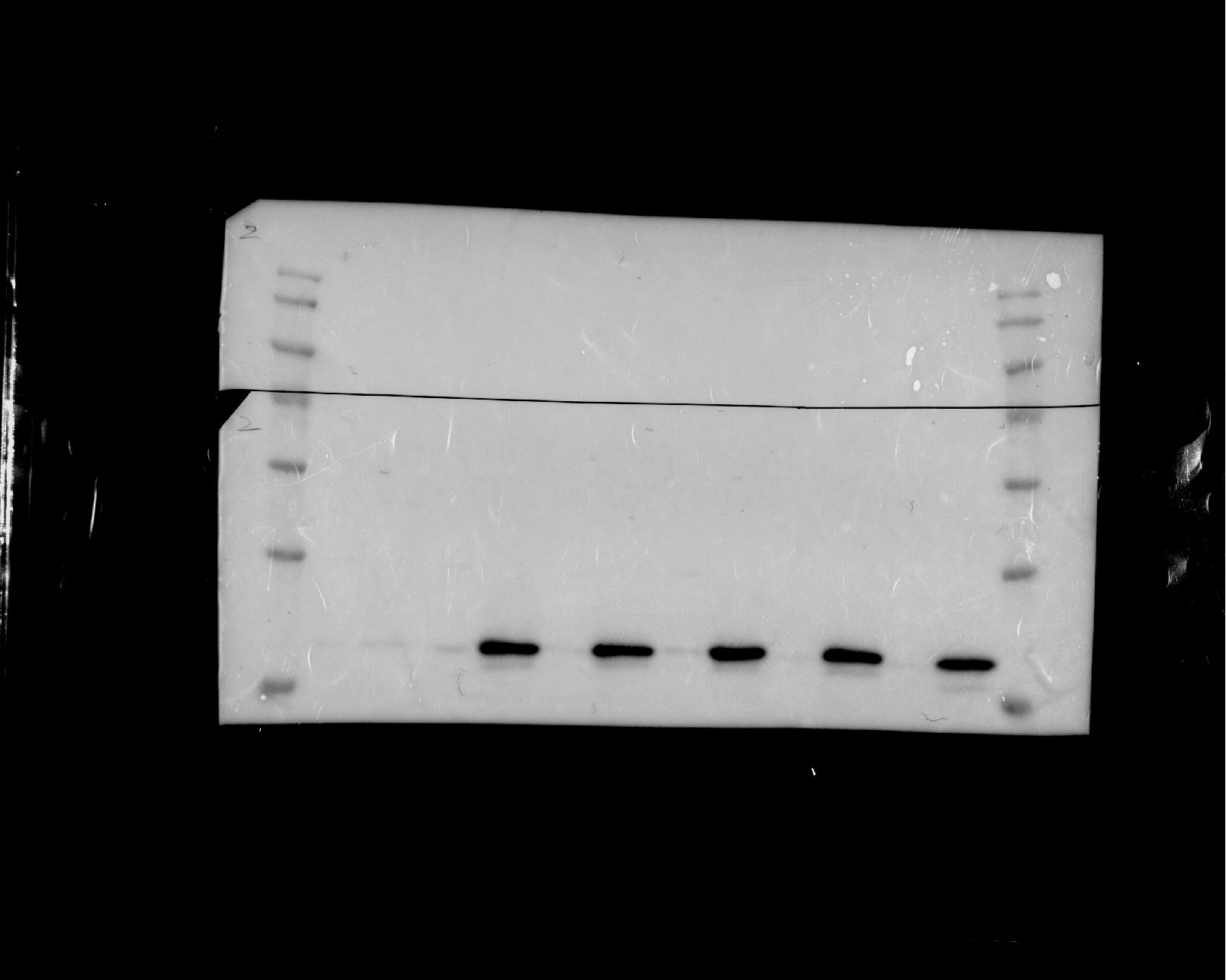

Supplement: Figure 2—figure supplement 2—source data 2. [file elife-101967-fig2-figsupp2-data2.zip › Figure 2-figure supplement 2-Source Data 2/Flag-Squ and Myc-SpnE anti-Flag rep2.tif]

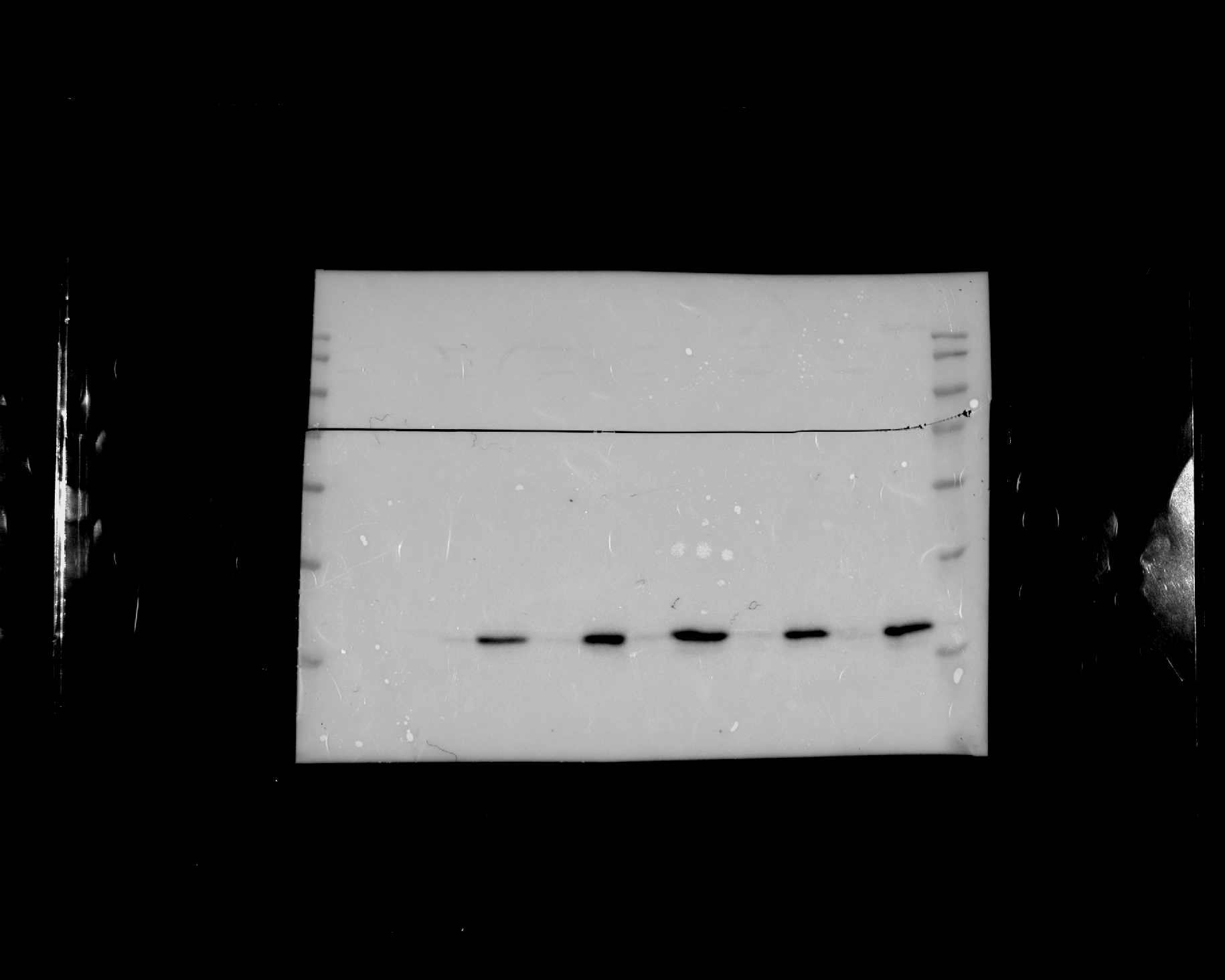

Supplement: Figure 2—figure supplement 2—source data 2. [file elife-101967-fig2-figsupp2-data2.zip › Figure 2-figure supplement 2-Source Data 2/Flag-Squ and Myc-SpnE anti-Flag rep3.tif]

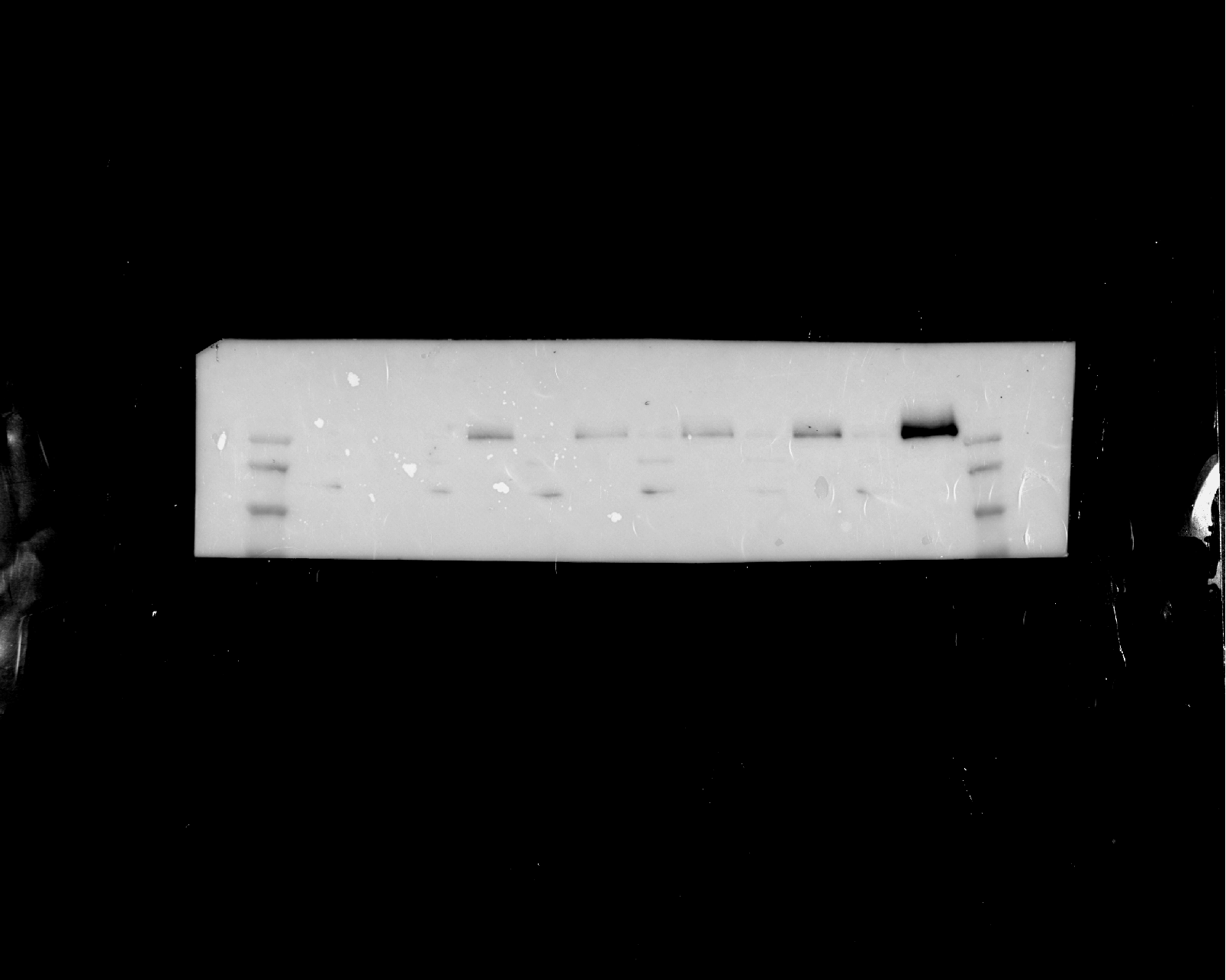

Supplement: Figure 2—figure supplement 2—source data 2. [file elife-101967-fig2-figsupp2-data2.zip › Figure 2-figure supplement 2-Source Data 2/Flag-Squ and Myc-SpnE anti-Myc rep1.tif]

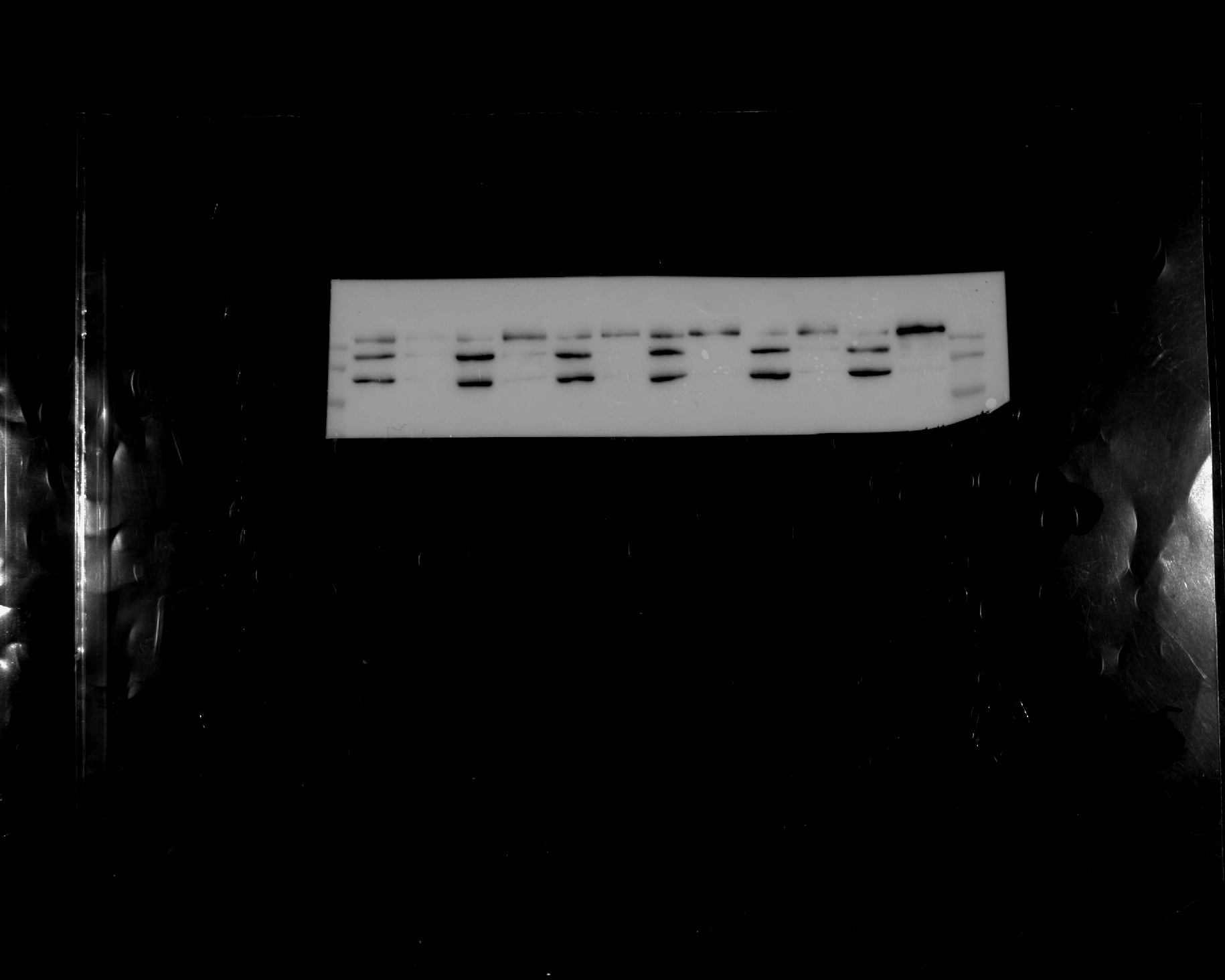

Supplement: Figure 2—figure supplement 2—source data 2. [file elife-101967-fig2-figsupp2-data2.zip › Figure 2-figure supplement 2-Source Data 2/Flag-Squ and Myc-SpnE anti-Myc rep3.tif]

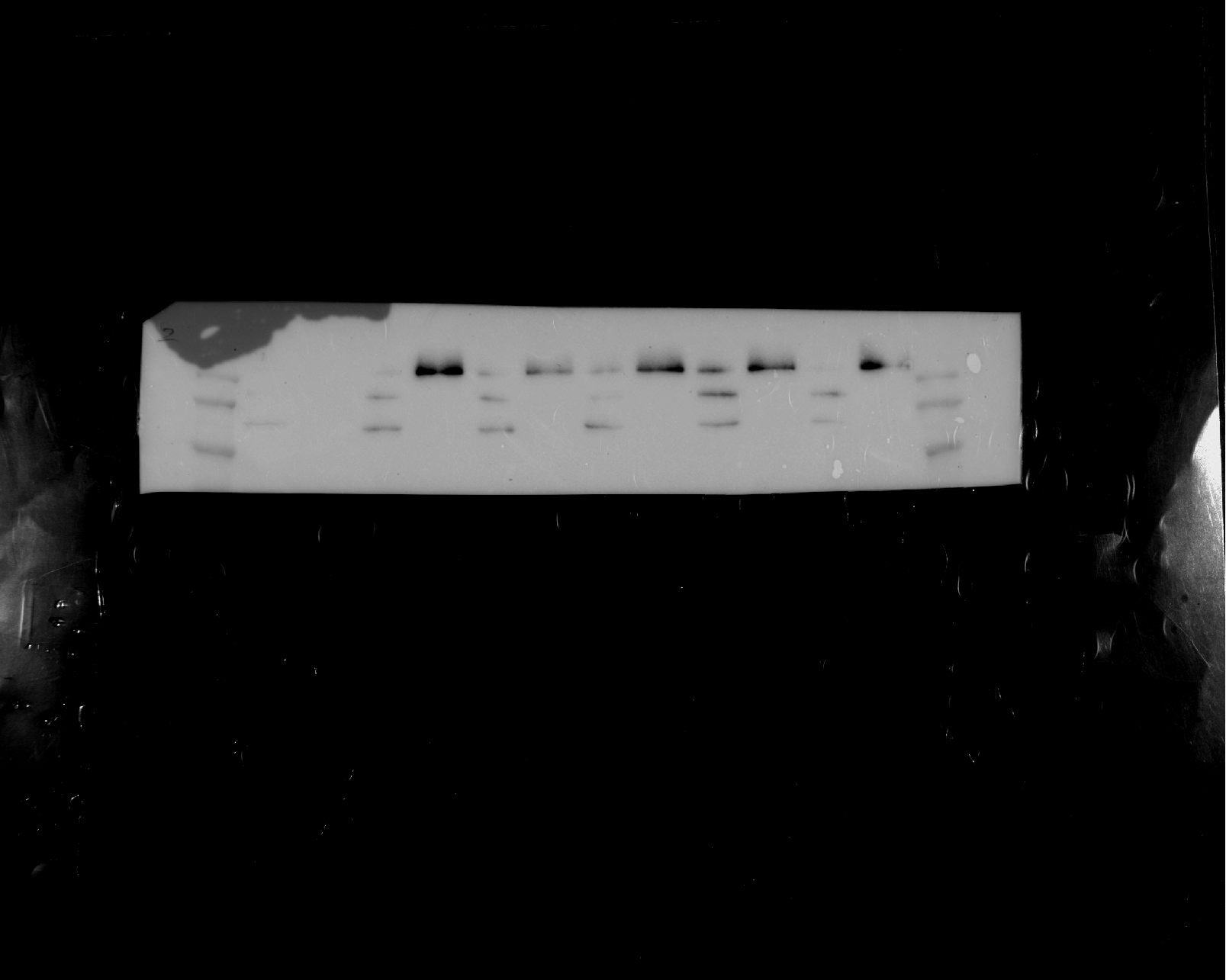

Supplement: Figure 2—figure supplement 2—source data 2. [file elife-101967-fig2-figsupp2-data2.zip › Figure 2-figure supplement 2-Source Data 2/Flag-Squ and Myc-SpnE anti-Myc rep2.tif]

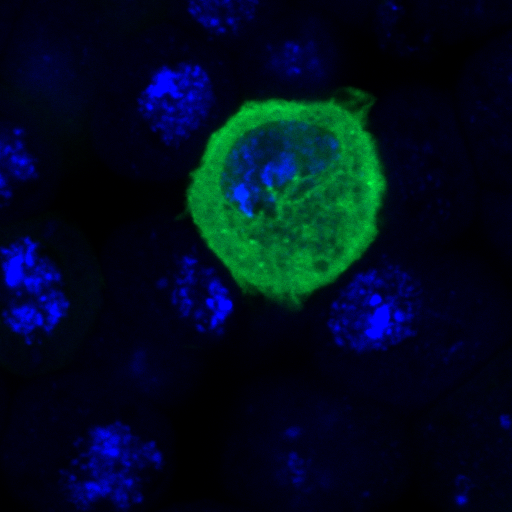

Supplement: Figure 2—figure supplement 2—source data 3. [file elife-101967-fig2-figsupp2-data3.zip › Figure 2-figure supplement 2-Source Data 3/Co-immunostaining in S2/GFP-Squ E107A/GFP-Squ E107A.tif]

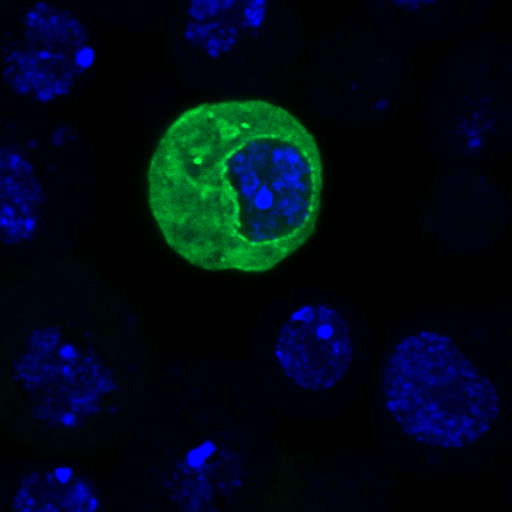

Supplement: Figure 2—figure supplement 2—source data 3. [file elife-101967-fig2-figsupp2-data3.zip › Figure 2-figure supplement 2-Source Data 3/Co-immunostaining in S2/GFP-Squ K163A/GFP-Squ K163A.tif]

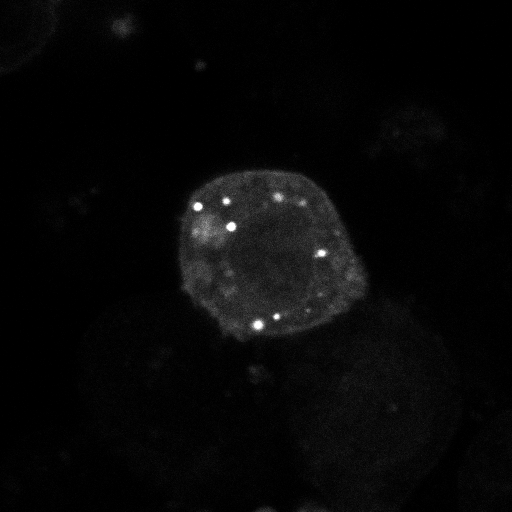

Supplement: Figure 2—figure supplement 2—source data 3. [file elife-101967-fig2-figsupp2-data3.zip › Figure 2-figure supplement 2-Source Data 3/Co-immunostaining in S2/GFP-Squ K163A + mk2-Spn-E WT/mk2-Spn-E WT.tif]

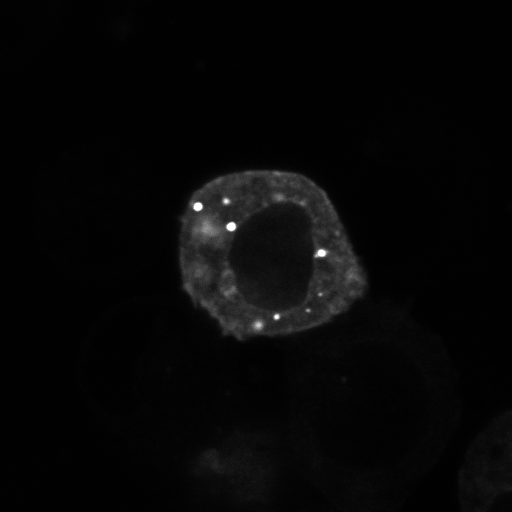

Supplement: Figure 2—figure supplement 2—source data 3. [file elife-101967-fig2-figsupp2-data3.zip › Figure 2-figure supplement 2-Source Data 3/Co-immunostaining in S2/GFP-Squ K163A + mk2-Spn-E WT/GFP-Squ K163A.tif]

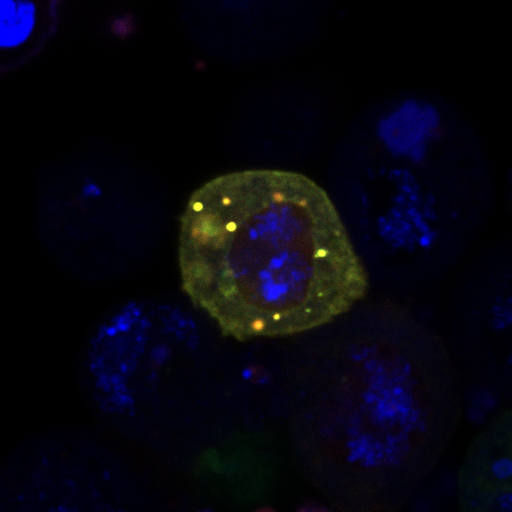

Supplement: Figure 2—figure supplement 2—source data 3. [file elife-101967-fig2-figsupp2-data3.zip › Figure 2-figure supplement 2-Source Data 3/Co-immunostaining in S2/GFP-Squ K163A + mk2-Spn-E WT/GFP-Squ K163A + mk2-Spn-E WT.tif]

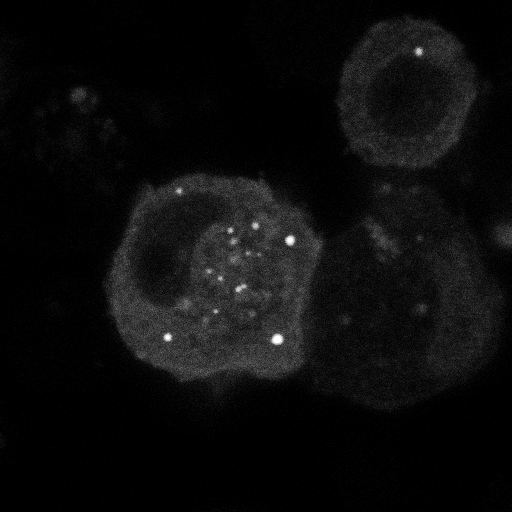

Supplement: Figure 2—figure supplement 2—source data 3. [file elife-101967-fig2-figsupp2-data3.zip › Figure 2-figure supplement 2-Source Data 3/Co-immunostaining in S2/GFP-Squ E107A + mk2-Spn-E WT/mk2-Spn-E WT.tif]

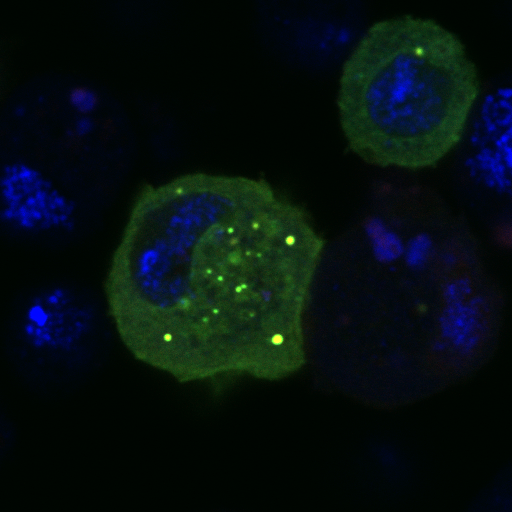

Supplement: Figure 2—figure supplement 2—source data 3. [file elife-101967-fig2-figsupp2-data3.zip › Figure 2-figure supplement 2-Source Data 3/Co-immunostaining in S2/GFP-Squ E107A + mk2-Spn-E WT/GFP-Squ E107A + mk2-Spn-E WT.tif]

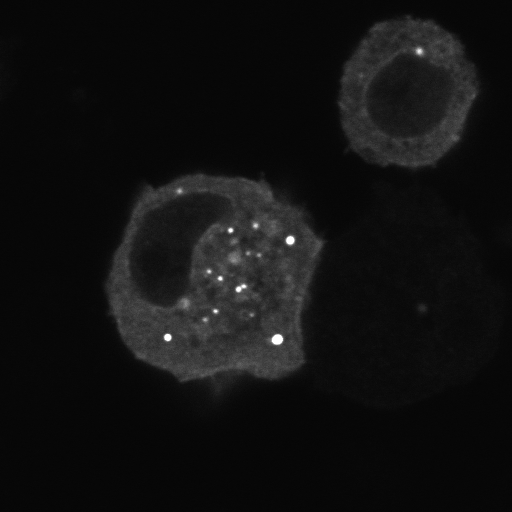

Supplement: Figure 2—figure supplement 2—source data 3. [file elife-101967-fig2-figsupp2-data3.zip › Figure 2-figure supplement 2-Source Data 3/Co-immunostaining in S2/GFP-Squ E107A + mk2-Spn-E WT/GFP-Squ E107A.tif]

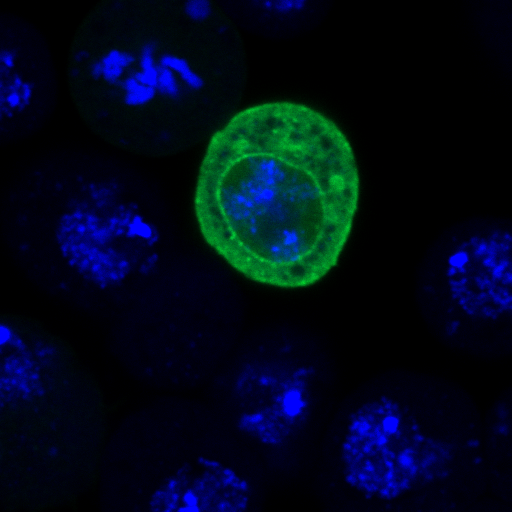

Supplement: Figure 2—figure supplement 2—source data 3. [file elife-101967-fig2-figsupp2-data3.zip › Figure 2-figure supplement 2-Source Data 3/Co-immunostaining in S2/GFP-Squ R115A/GFP-Squ R115A.tif]

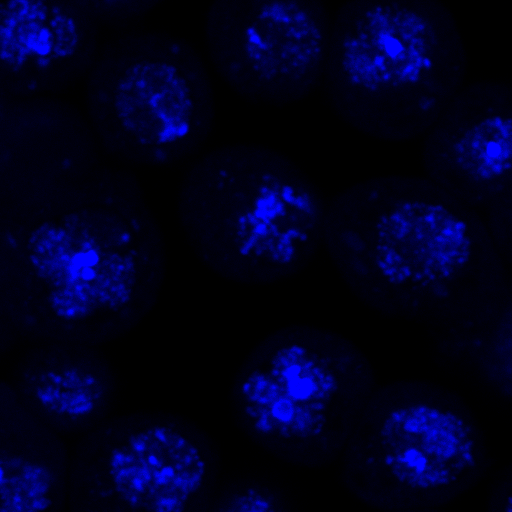

Supplement: Figure 2—figure supplement 2—source data 3. [file elife-101967-fig2-figsupp2-data3.zip › Figure 2-figure supplement 2-Source Data 3/Co-immunostaining in S2/GFP-Squ (-)/GFP-Squ (-).tif]

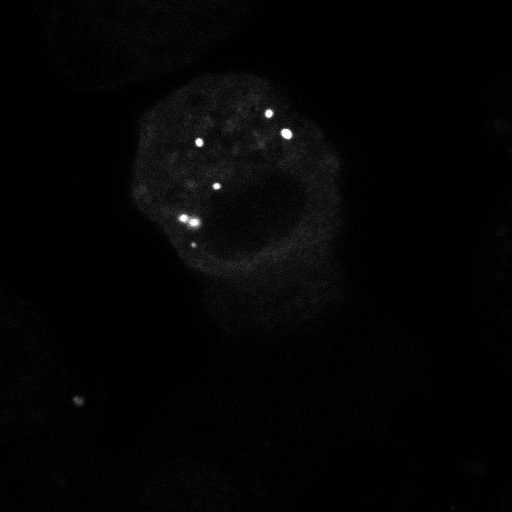

Supplement: Figure 2—figure supplement 2—source data 3. [file elife-101967-fig2-figsupp2-data3.zip › Figure 2-figure supplement 2-Source Data 3/Co-immunostaining in S2/GFP-Squ E109A + mk2-Spn-E WT/mk2-Spn-E WT.tif]

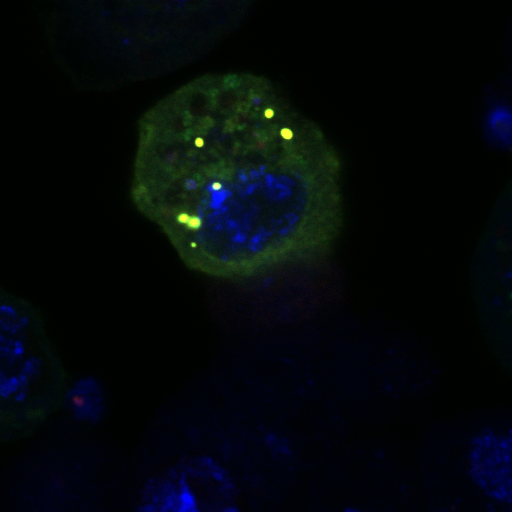

Supplement: Figure 2—figure supplement 2—source data 3. [file elife-101967-fig2-figsupp2-data3.zip › Figure 2-figure supplement 2-Source Data 3/Co-immunostaining in S2/GFP-Squ E109A + mk2-Spn-E WT/GFP-Squ E109A + mk2-Spn-E WT.tif]

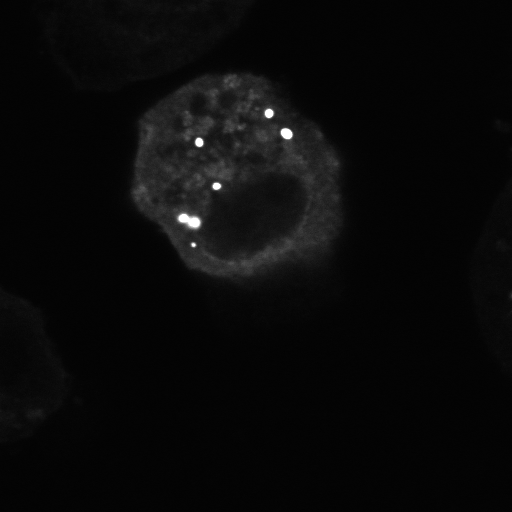

Supplement: Figure 2—figure supplement 2—source data 3. [file elife-101967-fig2-figsupp2-data3.zip › Figure 2-figure supplement 2-Source Data 3/Co-immunostaining in S2/GFP-Squ E109A + mk2-Spn-E WT/GFP-Squ E109A.tif]

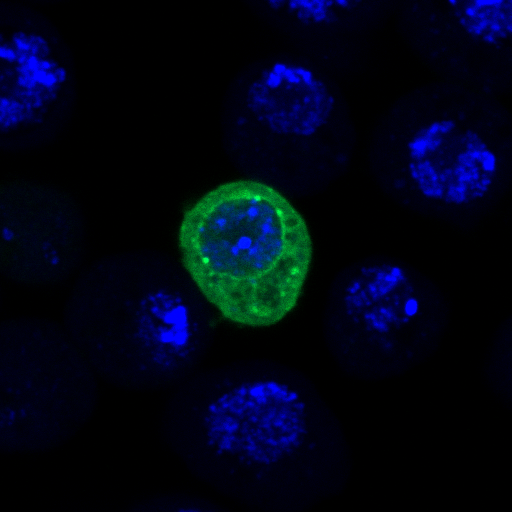

Supplement: Figure 2—figure supplement 2—source data 3. [file elife-101967-fig2-figsupp2-data3.zip › Figure 2-figure supplement 2-Source Data 3/Co-immunostaining in S2/GFP-Squ E109A/GFP-Squ E109A.tif]

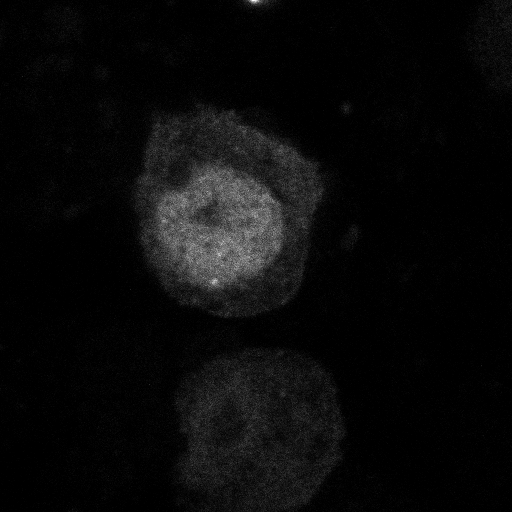

Supplement: Figure 2—figure supplement 2—source data 3. [file elife-101967-fig2-figsupp2-data3.zip › Figure 2-figure supplement 2-Source Data 3/Co-immunostaining in S2/GFP-Squ 4A + mk2-Spn-E WT/mk2-Spn-E WT.tif]

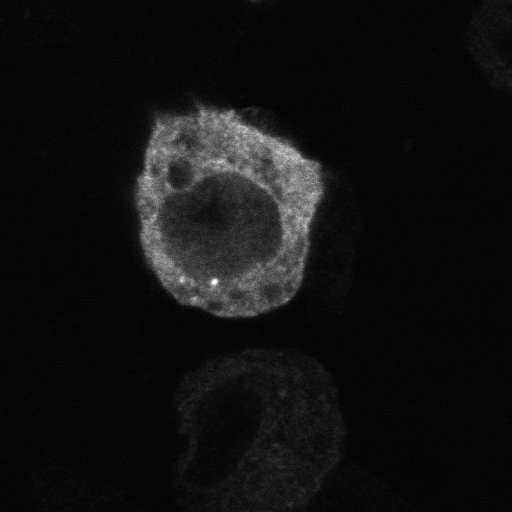

Supplement: Figure 2—figure supplement 2—source data 3. [file elife-101967-fig2-figsupp2-data3.zip › Figure 2-figure supplement 2-Source Data 3/Co-immunostaining in S2/GFP-Squ 4A + mk2-Spn-E WT/GFP-Squ 4A.tif]

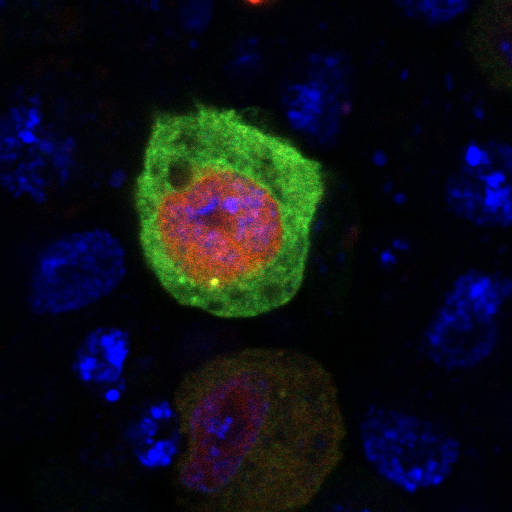

Supplement: Figure 2—figure supplement 2—source data 3. [file elife-101967-fig2-figsupp2-data3.zip › Figure 2-figure supplement 2-Source Data 3/Co-immunostaining in S2/GFP-Squ 4A + mk2-Spn-E WT/GFP-Squ 4A + mk2-Spn-E WT.tif]

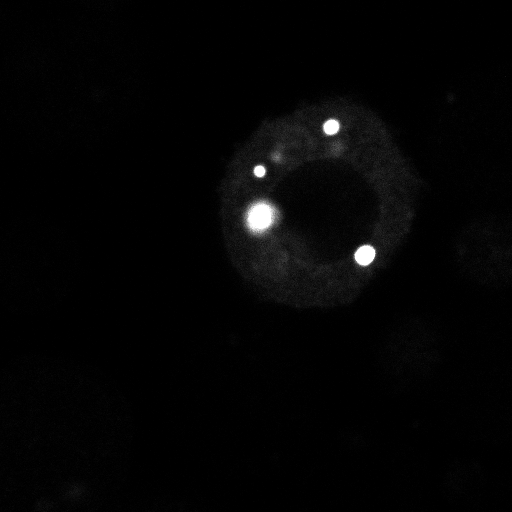

Supplement: Figure 2—figure supplement 2—source data 3. [file elife-101967-fig2-figsupp2-data3.zip › Figure 2-figure supplement 2-Source Data 3/Co-immunostaining in S2/GFP-Squ R115A + mk2-Spn-E WT/mk2-Spn-E WT.tif]

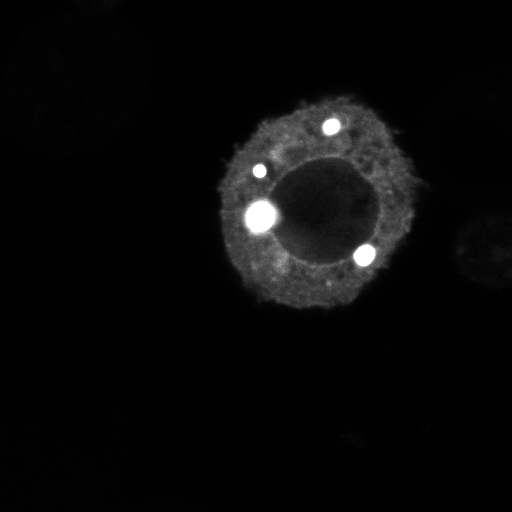

Supplement: Figure 2—figure supplement 2—source data 3. [file elife-101967-fig2-figsupp2-data3.zip › Figure 2-figure supplement 2-Source Data 3/Co-immunostaining in S2/GFP-Squ R115A + mk2-Spn-E WT/GFP-Squ R115A.tif]

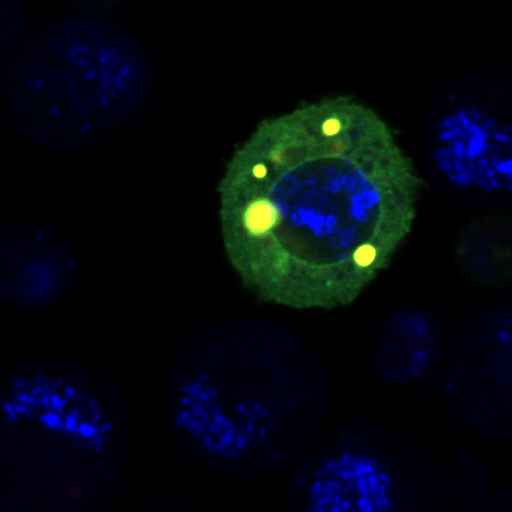

Supplement: Figure 2—figure supplement 2—source data 3. [file elife-101967-fig2-figsupp2-data3.zip › Figure 2-figure supplement 2-Source Data 3/Co-immunostaining in S2/GFP-Squ R115A + mk2-Spn-E WT/GFP-Squ R115A + mk2-Spn-E WT.tif]

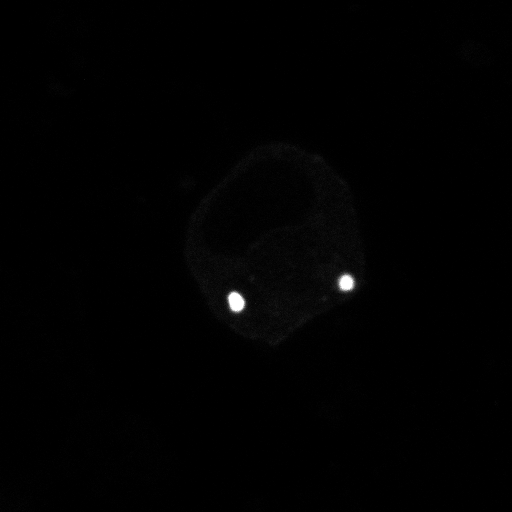

Supplement: Figure 2—figure supplement 2—source data 3. [file elife-101967-fig2-figsupp2-data3.zip › Figure 2-figure supplement 2-Source Data 3/Co-immunostaining in S2/GFP-Squ WT + mk2-Spn-E WT/mk2-Spn-E WT.tif]

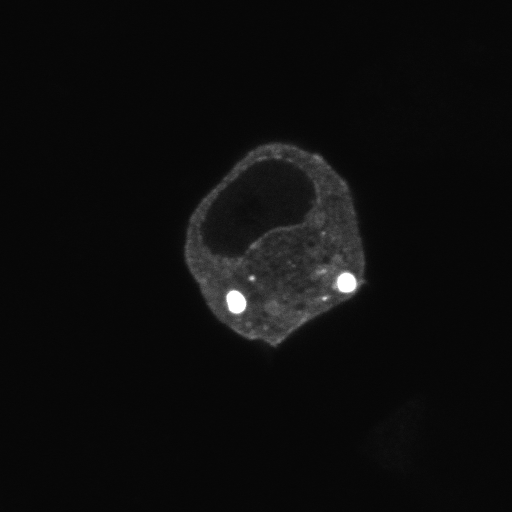

Supplement: Figure 2—figure supplement 2—source data 3. [file elife-101967-fig2-figsupp2-data3.zip › Figure 2-figure supplement 2-Source Data 3/Co-immunostaining in S2/GFP-Squ WT + mk2-Spn-E WT/GFP-Squ WT.tif]

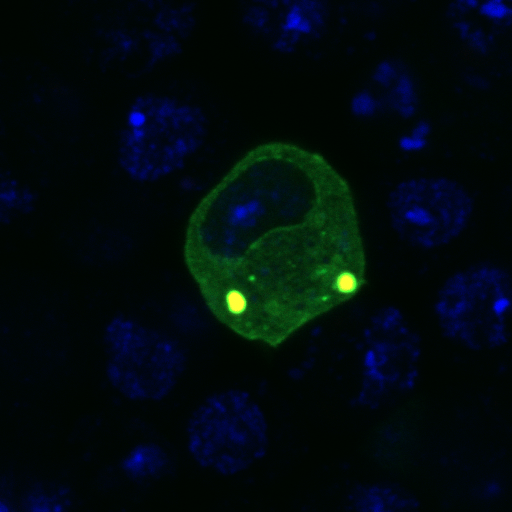

Supplement: Figure 2—figure supplement 2—source data 3. [file elife-101967-fig2-figsupp2-data3.zip › Figure 2-figure supplement 2-Source Data 3/Co-immunostaining in S2/GFP-Squ WT + mk2-Spn-E WT/GFP-Squ WT + mk2-Spn-E WT.tif]

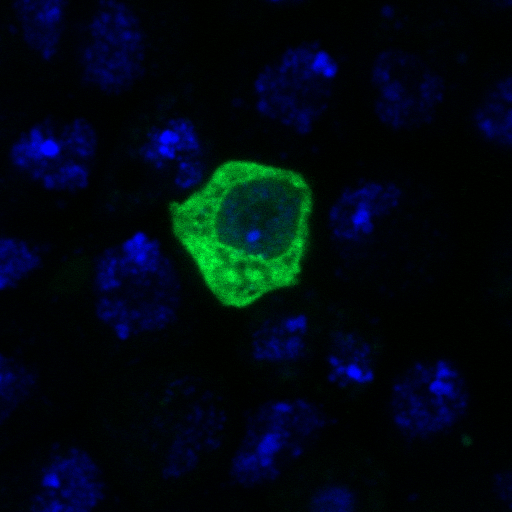

Supplement: Figure 2—figure supplement 2—source data 3. [file elife-101967-fig2-figsupp2-data3.zip › Figure 2-figure supplement 2-Source Data 3/Co-immunostaining in S2/GFP-Squ 4A/GFP-Squ 4A.tif]

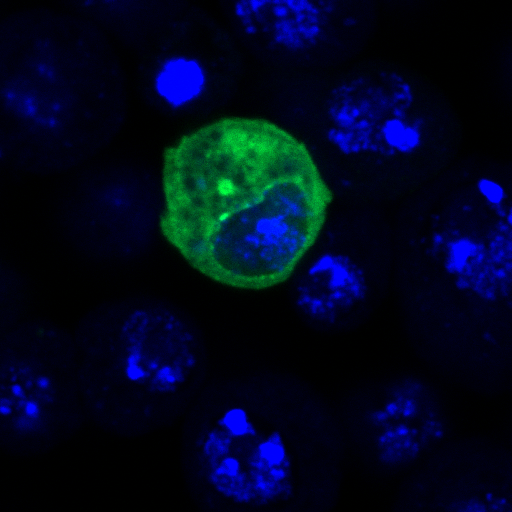

Supplement: Figure 2—figure supplement 2—source data 3. [file elife-101967-fig2-figsupp2-data3.zip › Figure 2-figure supplement 2-Source Data 3/Co-immunostaining in S2/GFP-Squ WT/GFP-Squ WT.tif]

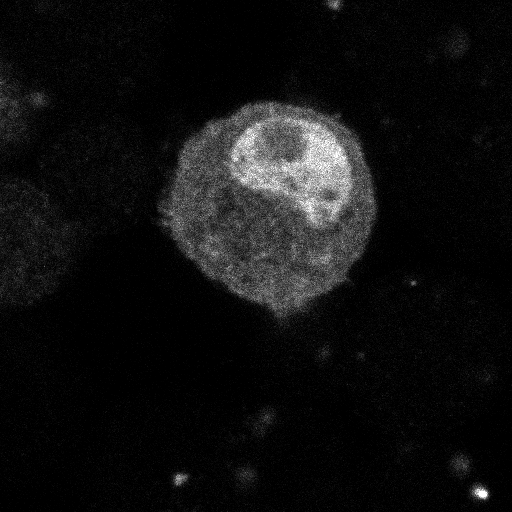

Supplement: Figure 2—figure supplement 2—source data 3. [file elife-101967-fig2-figsupp2-data3.zip › Figure 2-figure supplement 2-Source Data 3/Co-immunostaining in S2/GFP-Squ (-) + mk2-Spn-E WT/mk2-SpnE WT.tif]

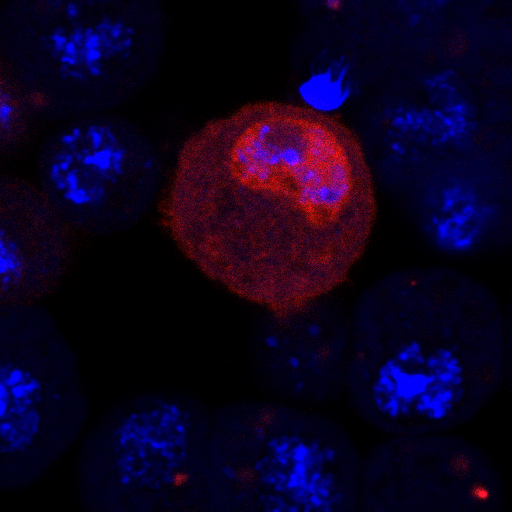

Supplement: Figure 2—figure supplement 2—source data 3. [file elife-101967-fig2-figsupp2-data3.zip › Figure 2-figure supplement 2-Source Data 3/Co-immunostaining in S2/GFP-Squ (-) + mk2-Spn-E WT/GFP-Squ (-) + mk2-Spn-E WT.tif]

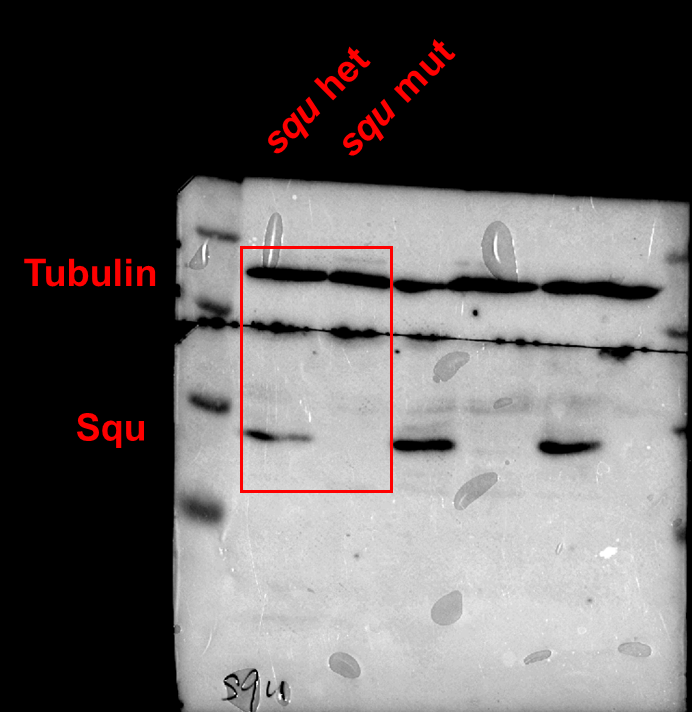

Supplement: Figure 3—source data 1. [file elife-101967-fig3-data1.zip › Figure 3-Source Data 1/Squ and Tubulin with label.tif]

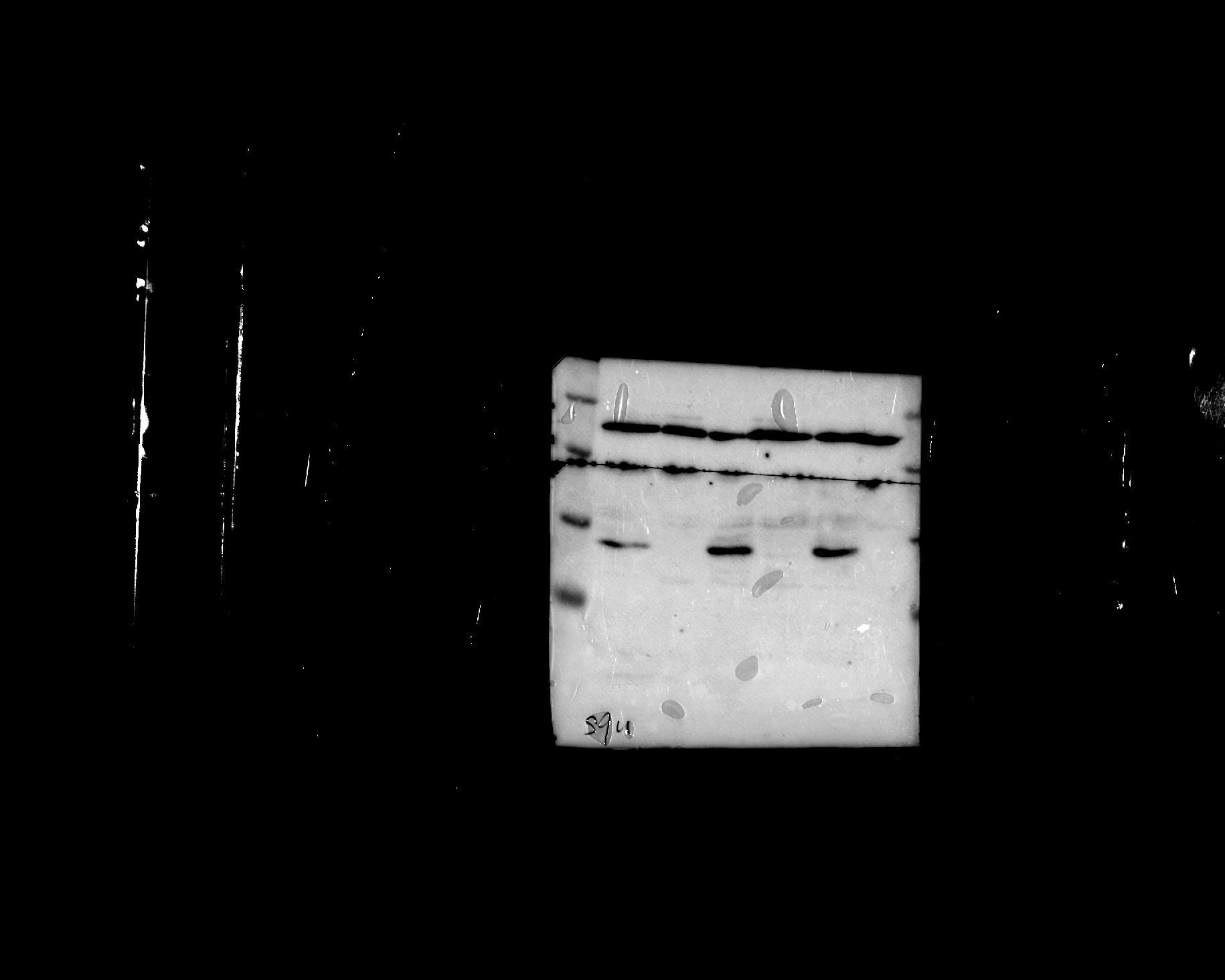

Supplement: Figure 3—source data 2. [file elife-101967-fig3-data2.zip › Figure 3-Source Data 2/Squ and Tubulin.tif]

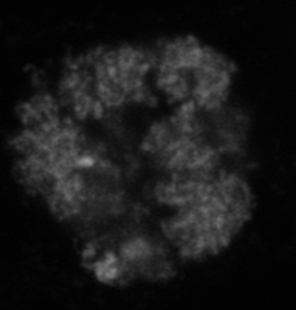

Supplement: Figure 3—source data 3. [file elife-101967-fig3-data3.zip › Figure 3-Source Data 3/Figure 3B confocol raw data/Fig3B_DAPI/Cropped image/dapi crop 2.tif]

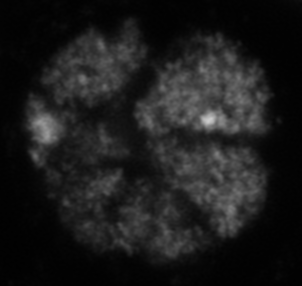

Supplement: Figure 3—source data 3. [file elife-101967-fig3-data3.zip › Figure 3-Source Data 3/Figure 3B confocol raw data/Fig3B_DAPI/Cropped image/dapi crop 3.tif]

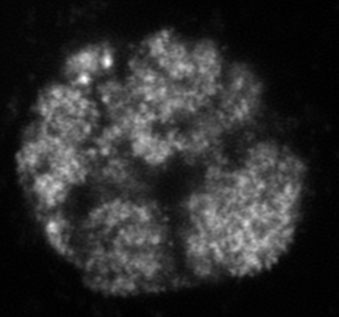

Supplement: Figure 3—source data 3. [file elife-101967-fig3-data3.zip › Figure 3-Source Data 3/Figure 3B confocol raw data/Fig3B_DAPI/Cropped image/dapi crop 1.tif]

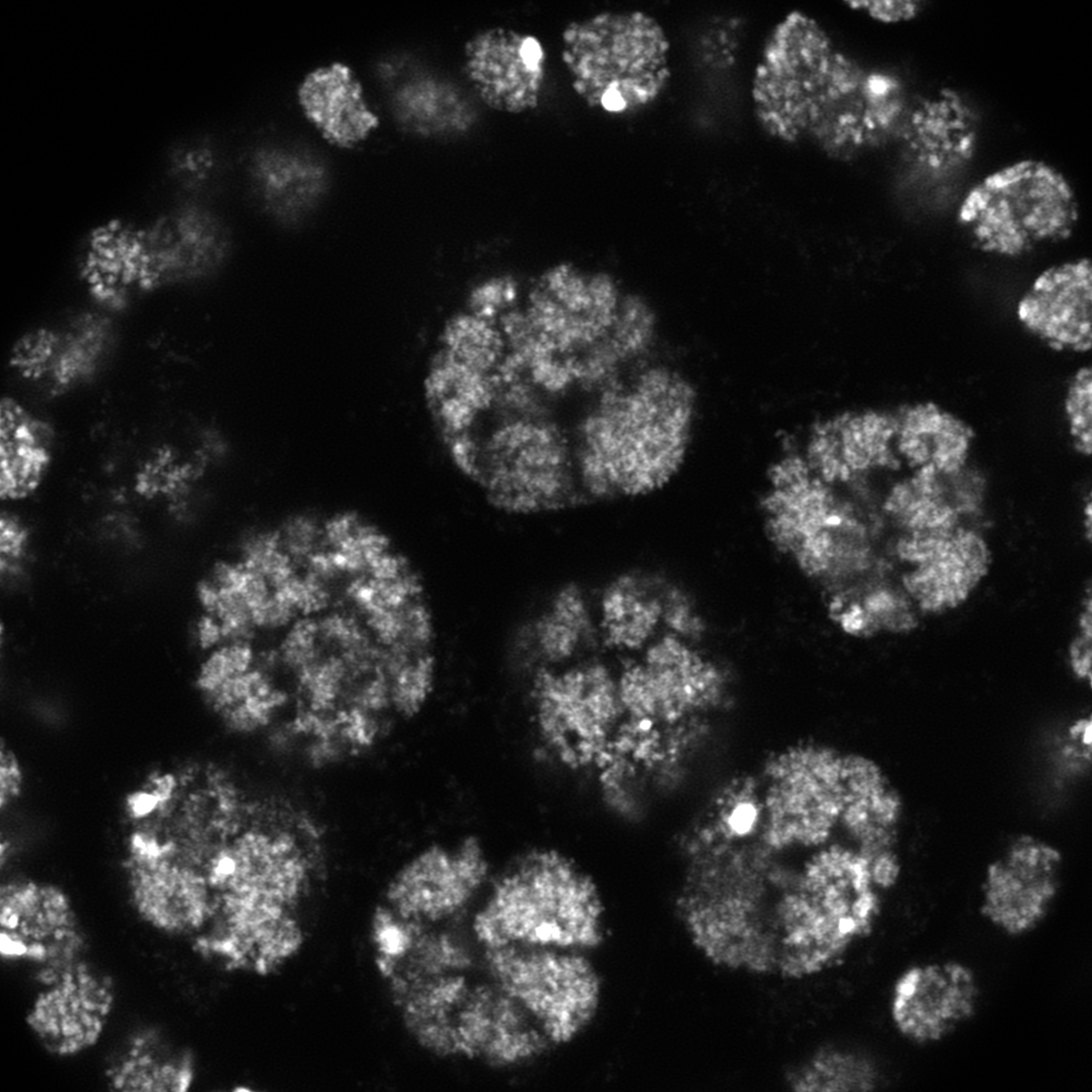

Supplement: Figure 3—source data 3. [file elife-101967-fig3-data3.zip › Figure 3-Source Data 3/Figure 3B confocol raw data/Fig3B_DAPI/Original files/DAPI.tif]
