# Supplementary figures and images for: In silico screening by AlphaFold2 program revealed the potential binding partners of nuage-localizing proteins and piRNA-related proteins (part 2 of 2)
Source: eLife. 2025 Apr 22;13:RP101967. doi: 10.7554/eLife.101967 (PMC12014135; doi:10.7554/eLife.101967)

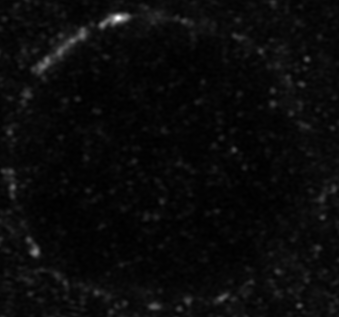

Supplement: Figure 3—source data 3. [file elife-101967-fig3-data3.zip › Figure 3-Source Data 3/Figure 3B confocol raw data/Fig3B_Squ/Cropped images/squ crop 1.tif]

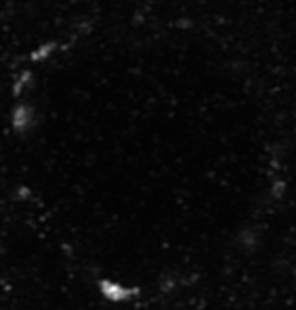

Supplement: Figure 3—source data 3. [file elife-101967-fig3-data3.zip › Figure 3-Source Data 3/Figure 3B confocol raw data/Fig3B_Squ/Cropped images/squ crop 2.tif]

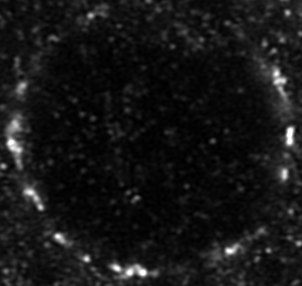

Supplement: Figure 3—source data 3. [file elife-101967-fig3-data3.zip › Figure 3-Source Data 3/Figure 3B confocol raw data/Fig3B_Squ/Cropped images/squ crop 3.tif]

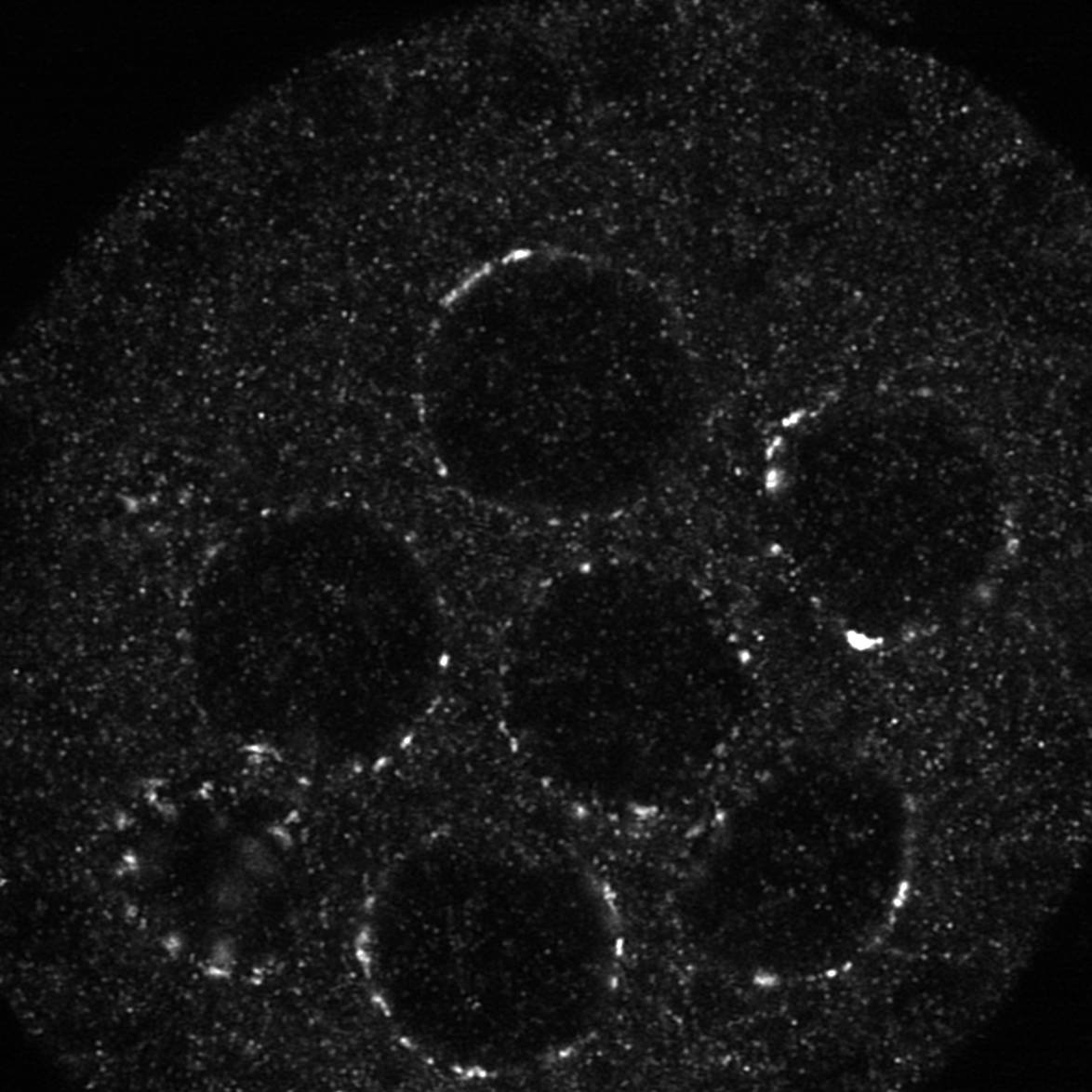

Supplement: Figure 3—source data 3. [file elife-101967-fig3-data3.zip › Figure 3-Source Data 3/Figure 3B confocol raw data/Fig3B_Squ/Original files/Squ.tif]

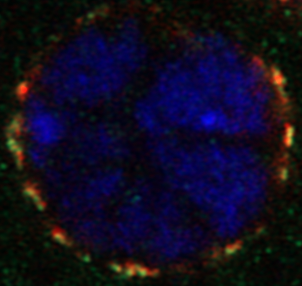

Supplement: Figure 3—source data 3. [file elife-101967-fig3-data3.zip › Figure 3-Source Data 3/Figure 3B confocol raw data/Fig3B_Merge/Cropped images/merge crop 3.tif]

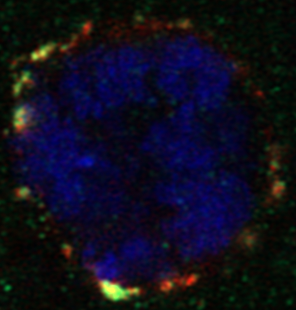

Supplement: Figure 3—source data 3. [file elife-101967-fig3-data3.zip › Figure 3-Source Data 3/Figure 3B confocol raw data/Fig3B_Merge/Cropped images/merge crop 2.tif]

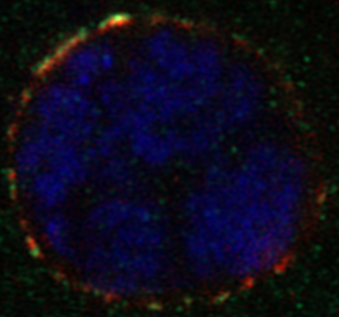

Supplement: Figure 3—source data 3. [file elife-101967-fig3-data3.zip › Figure 3-Source Data 3/Figure 3B confocol raw data/Fig3B_Merge/Cropped images/merge crop 1.tif]

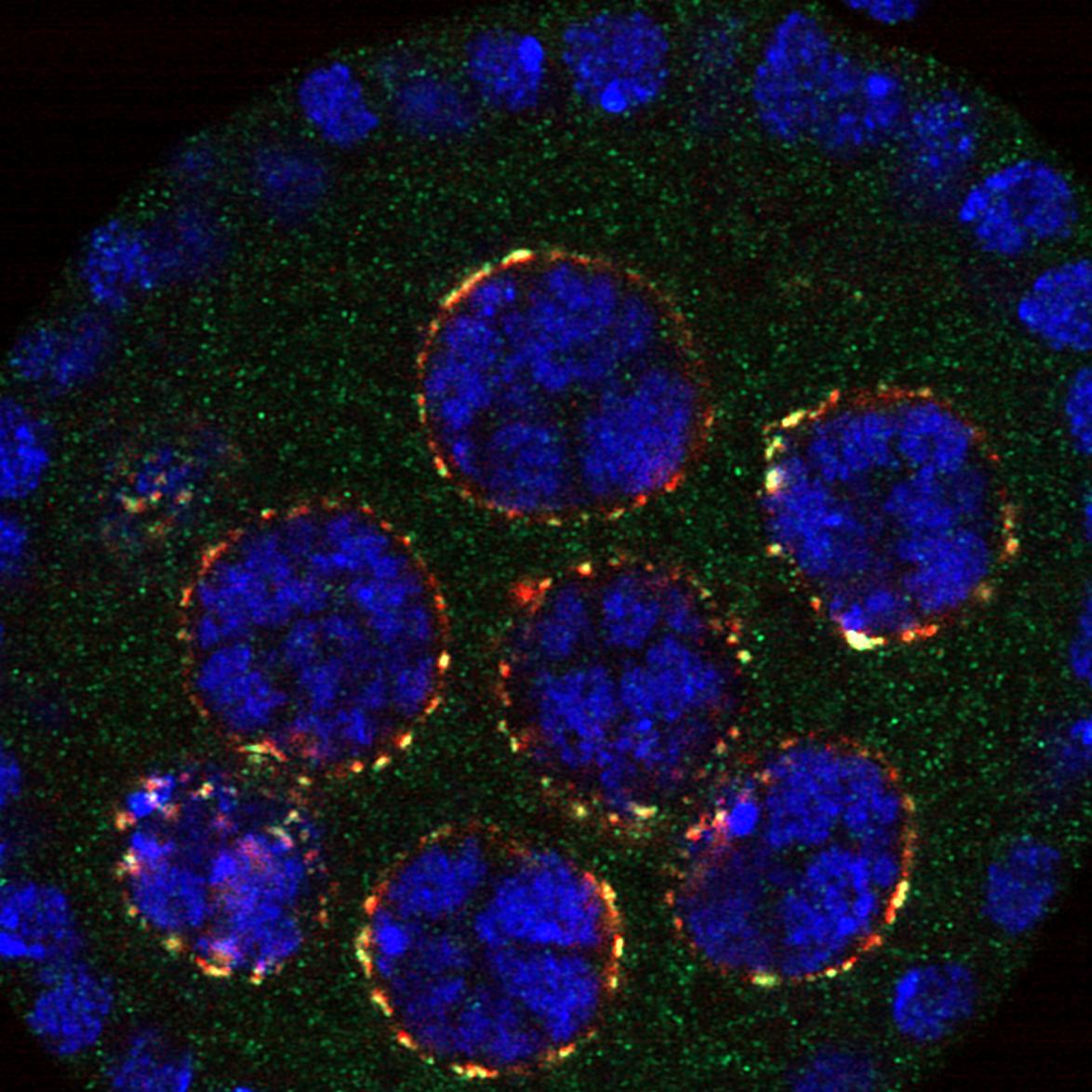

Supplement: Figure 3—source data 3. [file elife-101967-fig3-data3.zip › Figure 3-Source Data 3/Figure 3B confocol raw data/Fig3B_Merge/Original files/Merge.tif]

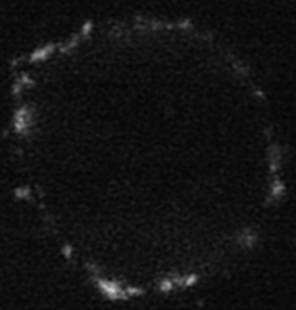

Supplement: Figure 3—source data 3. [file elife-101967-fig3-data3.zip › Figure 3-Source Data 3/Figure 3B confocol raw data/Fig3B_Spn-E_mk2/Cropped images/Spn-E_mk2 crop 2.tif]

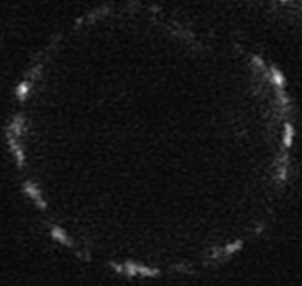

Supplement: Figure 3—source data 3. [file elife-101967-fig3-data3.zip › Figure 3-Source Data 3/Figure 3B confocol raw data/Fig3B_Spn-E_mk2/Cropped images/Spn-E_mk2 crop 3.tif]

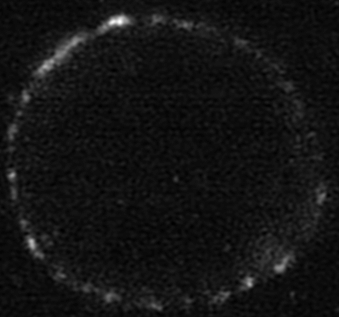

Supplement: Figure 3—source data 3. [file elife-101967-fig3-data3.zip › Figure 3-Source Data 3/Figure 3B confocol raw data/Fig3B_Spn-E_mk2/Cropped images/Spn-E_mk2 crop 1.tif]

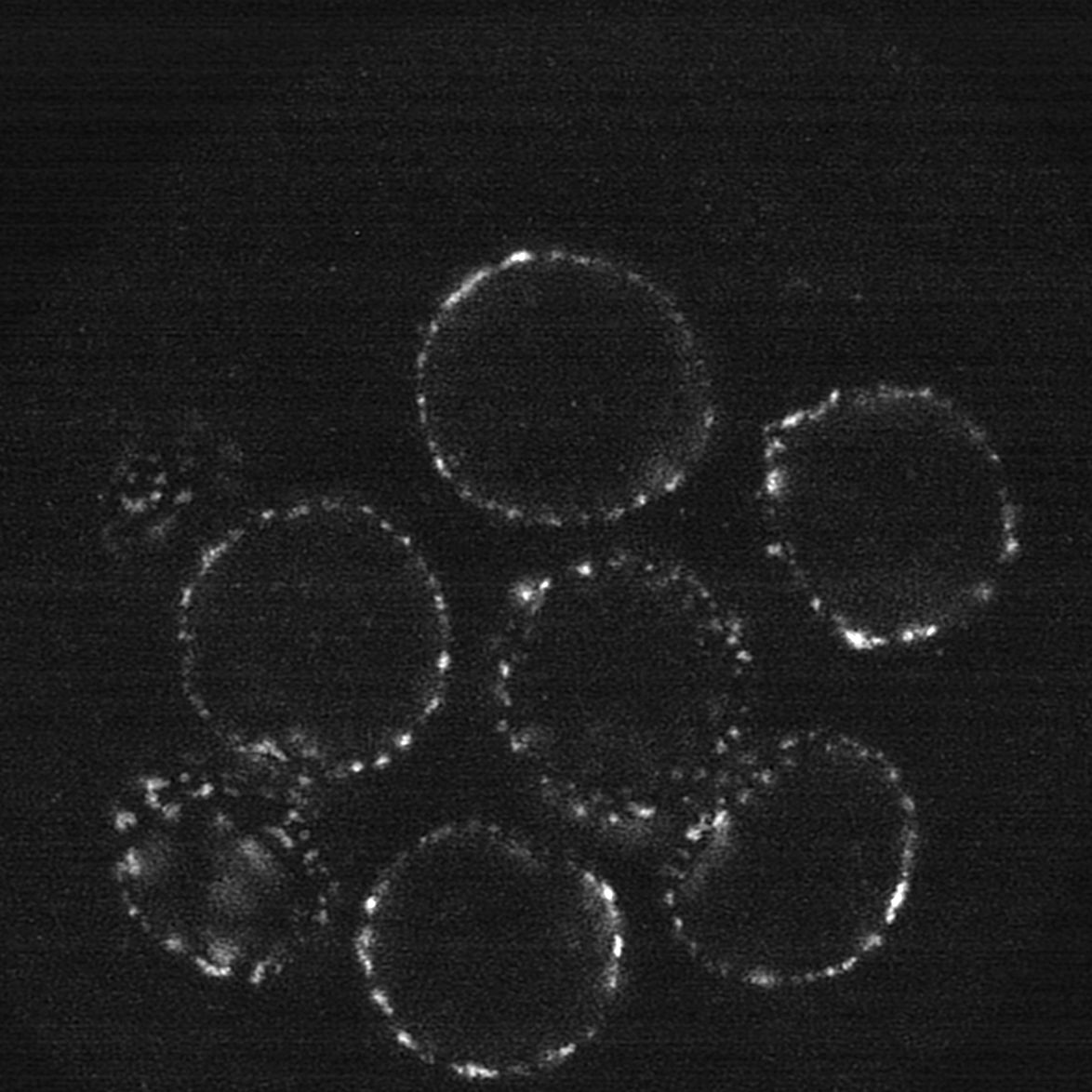

Supplement: Figure 3—source data 3. [file elife-101967-fig3-data3.zip › Figure 3-Source Data 3/Figure 3B confocol raw data/Fig3B_Spn-E_mk2/Original files/Spn-E_mk2.tif]

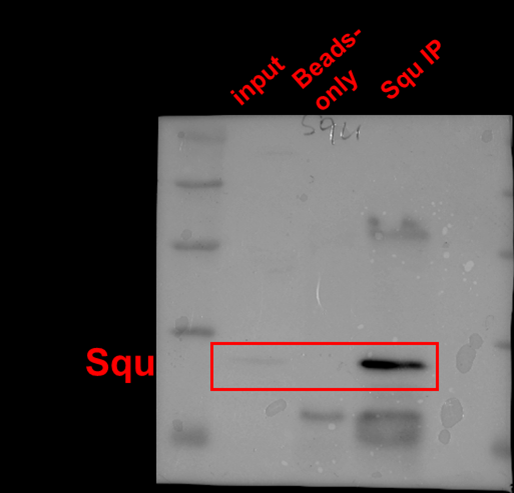

Supplement: Figure 3—source data 4. [file elife-101967-fig3-data4.zip › Figure 3-Source Data 4/Squ in Squ ip with label.tif]

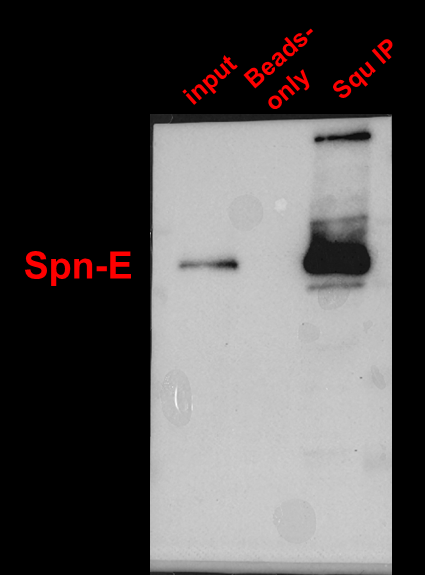

Supplement: Figure 3—source data 4. [file elife-101967-fig3-data4.zip › Figure 3-Source Data 4/Spn-E in Squ ip with label.tif]

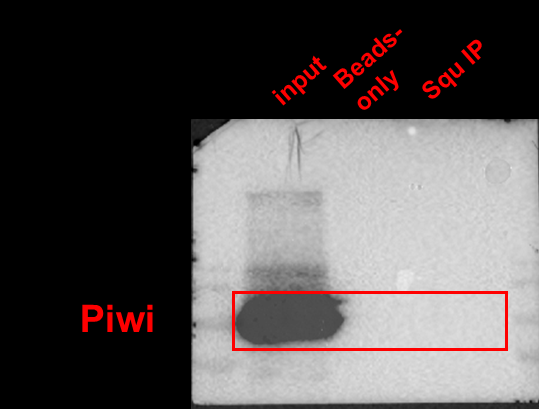

Supplement: Figure 3—source data 4. [file elife-101967-fig3-data4.zip › Figure 3-Source Data 4/Piwi in Squ ip with label.tif]

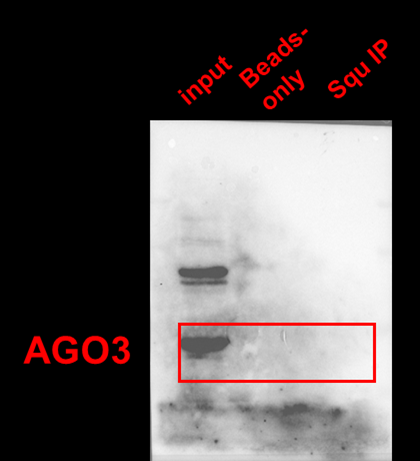

Supplement: Figure 3—source data 4. [file elife-101967-fig3-data4.zip › Figure 3-Source Data 4/AGO3 in Squ ip with label.tif]

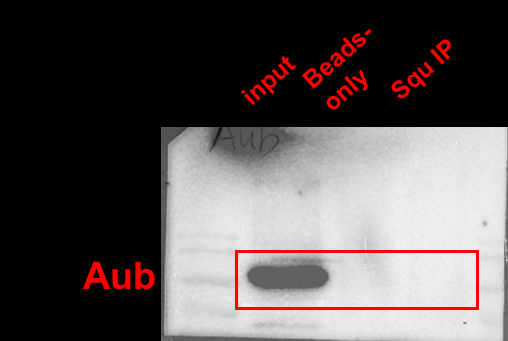

Supplement: Figure 3—source data 4. [file elife-101967-fig3-data4.zip › Figure 3-Source Data 4/Aub in Squ ip with label.tif]

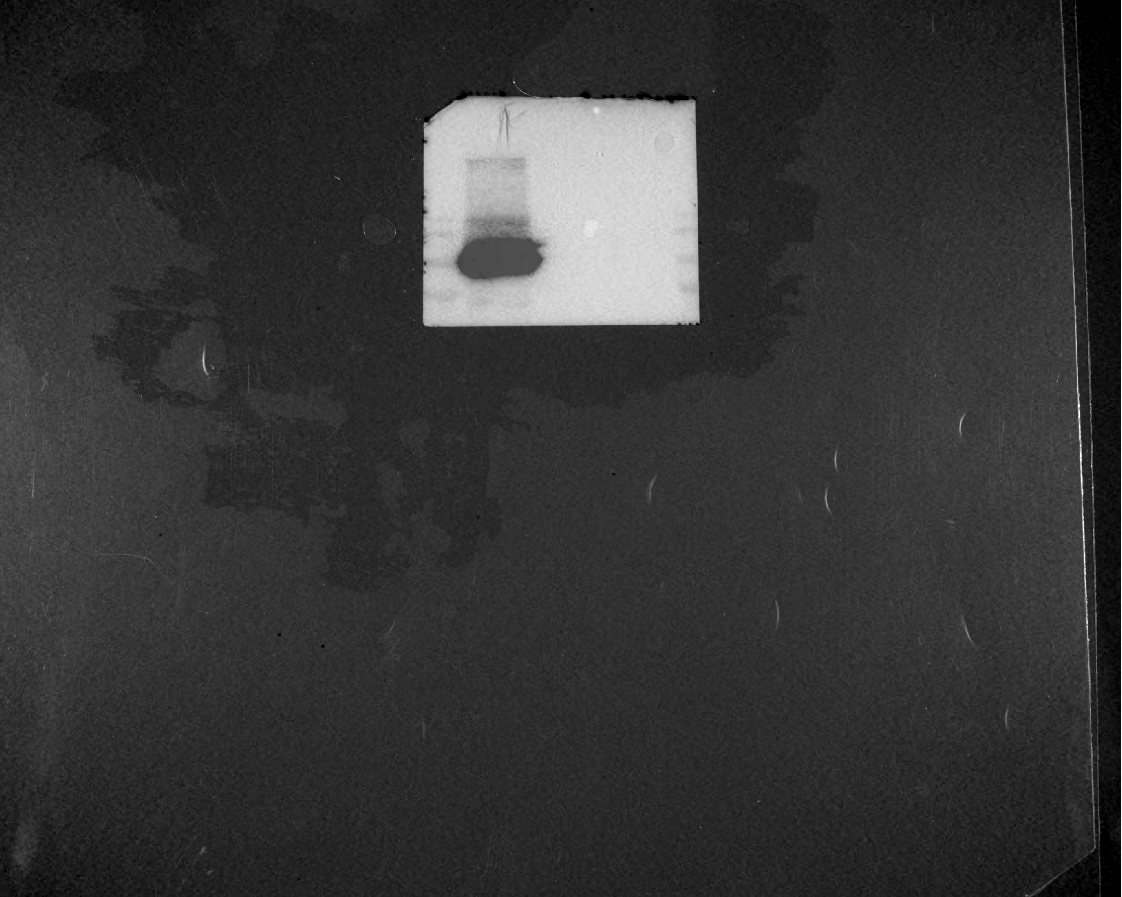

Supplement: Figure 3—source data 5. [file elife-101967-fig3-data5.zip › Figure 3-Source Data 5/Piwi in Squ ip.tif]

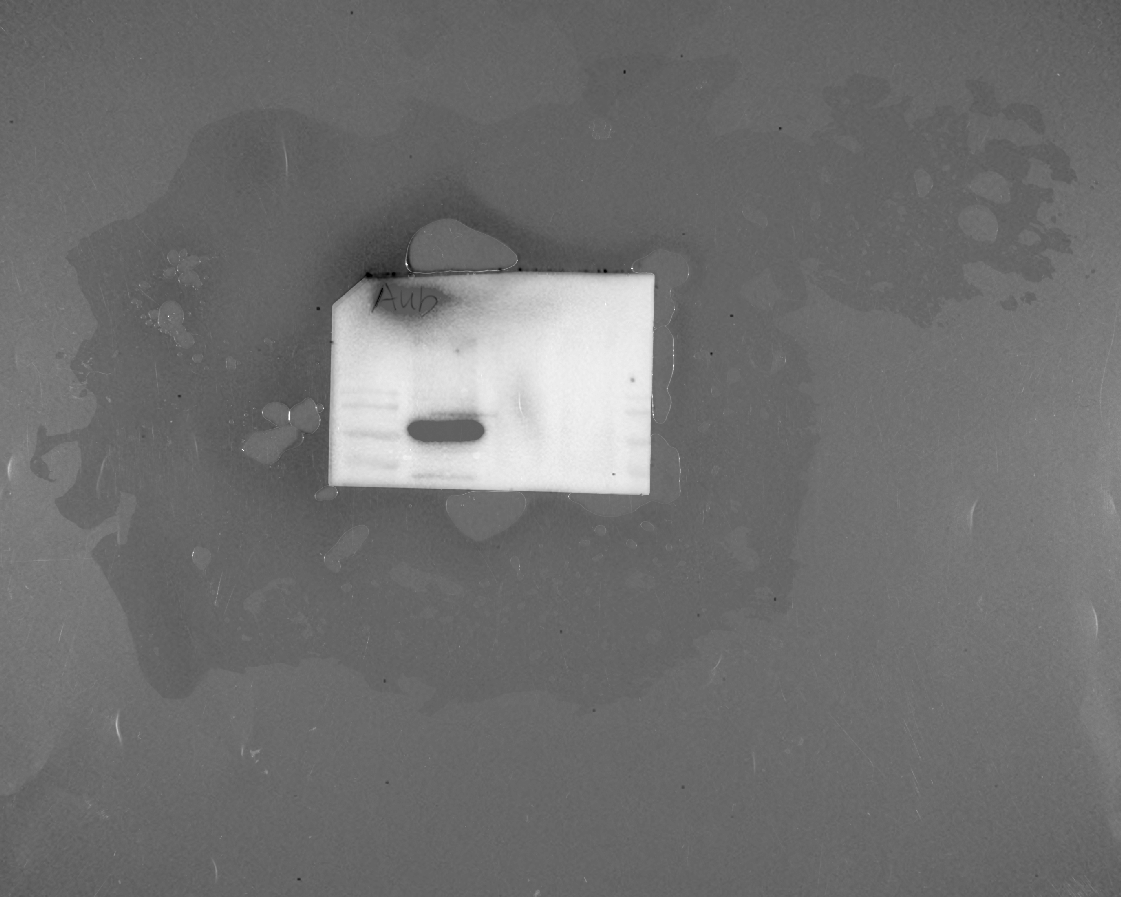

Supplement: Figure 3—source data 5. [file elife-101967-fig3-data5.zip › Figure 3-Source Data 5/Aub in Squ ip.tif]

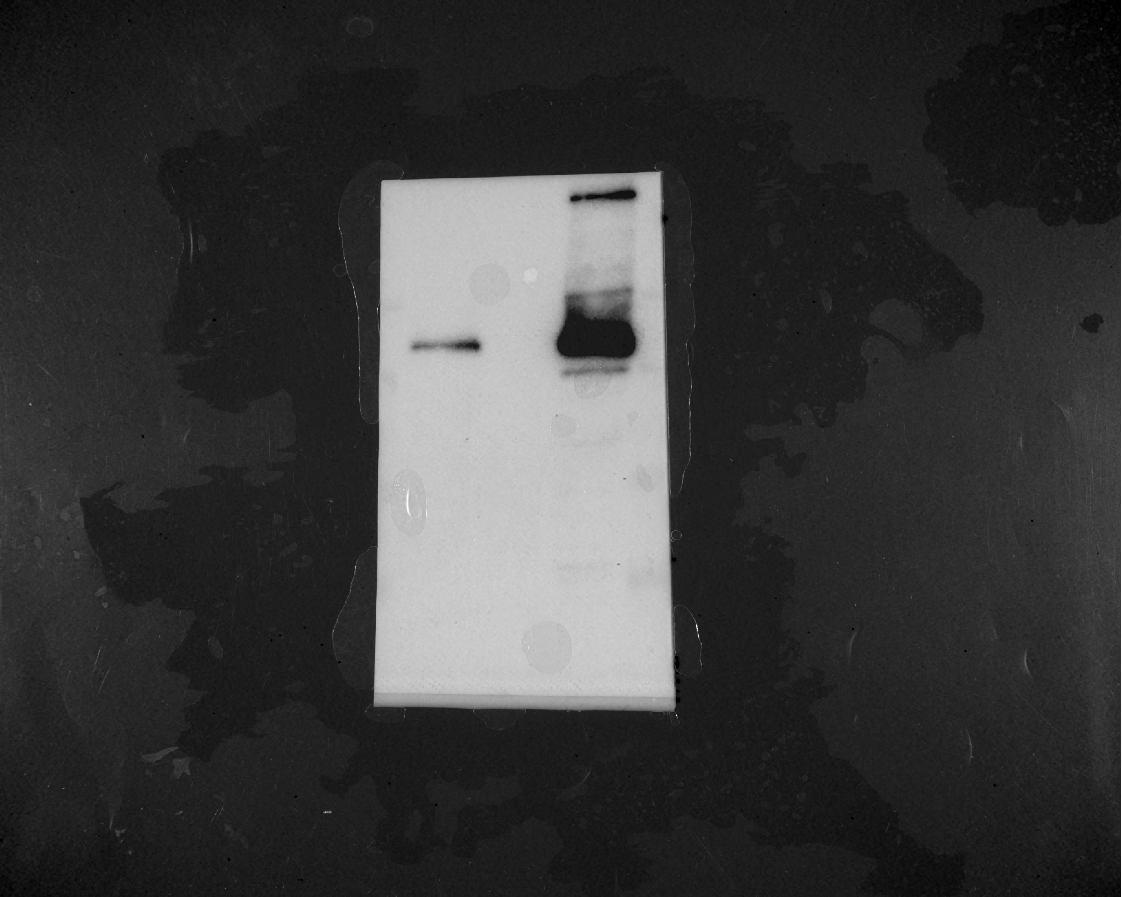

Supplement: Figure 3—source data 5. [file elife-101967-fig3-data5.zip › Figure 3-Source Data 5/Spn-E in Squ ip.tif]

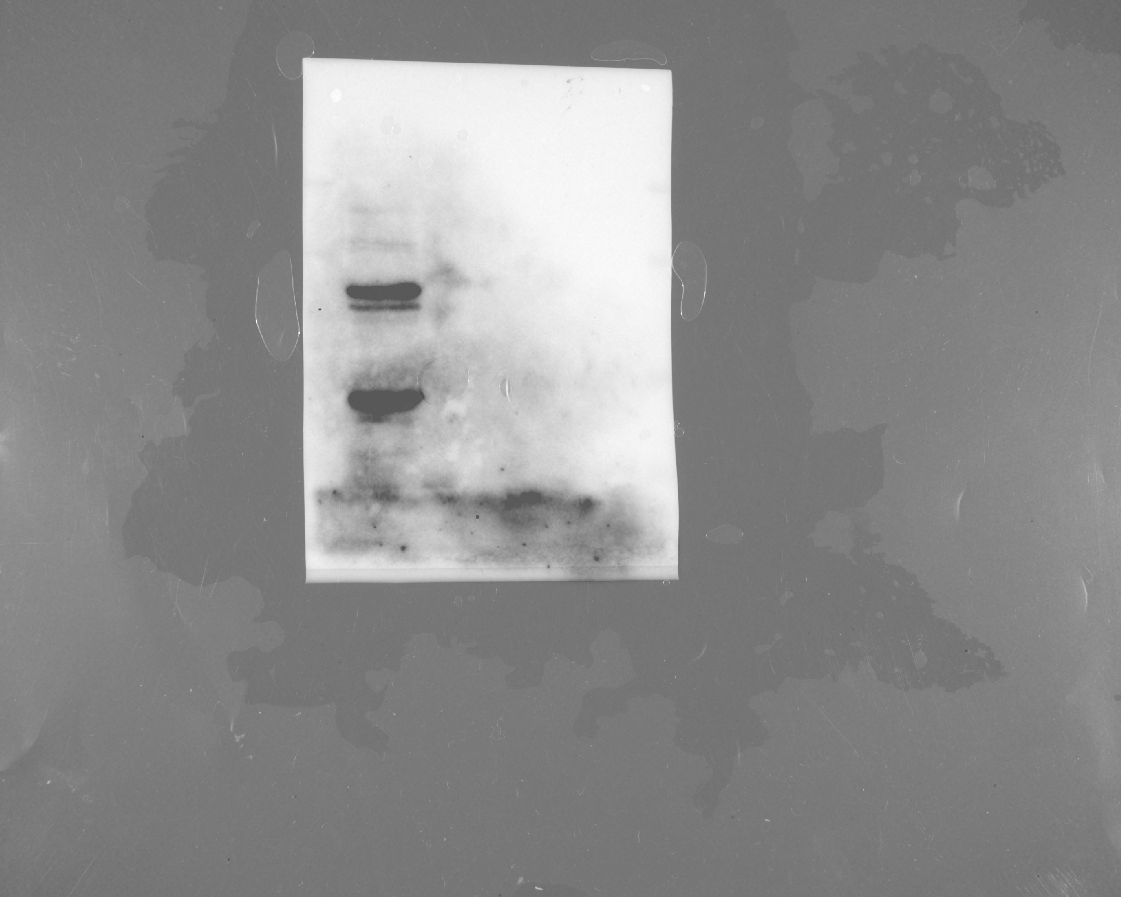

Supplement: Figure 3—source data 5. [file elife-101967-fig3-data5.zip › Figure 3-Source Data 5/AGO3 in Squ ip.tif]

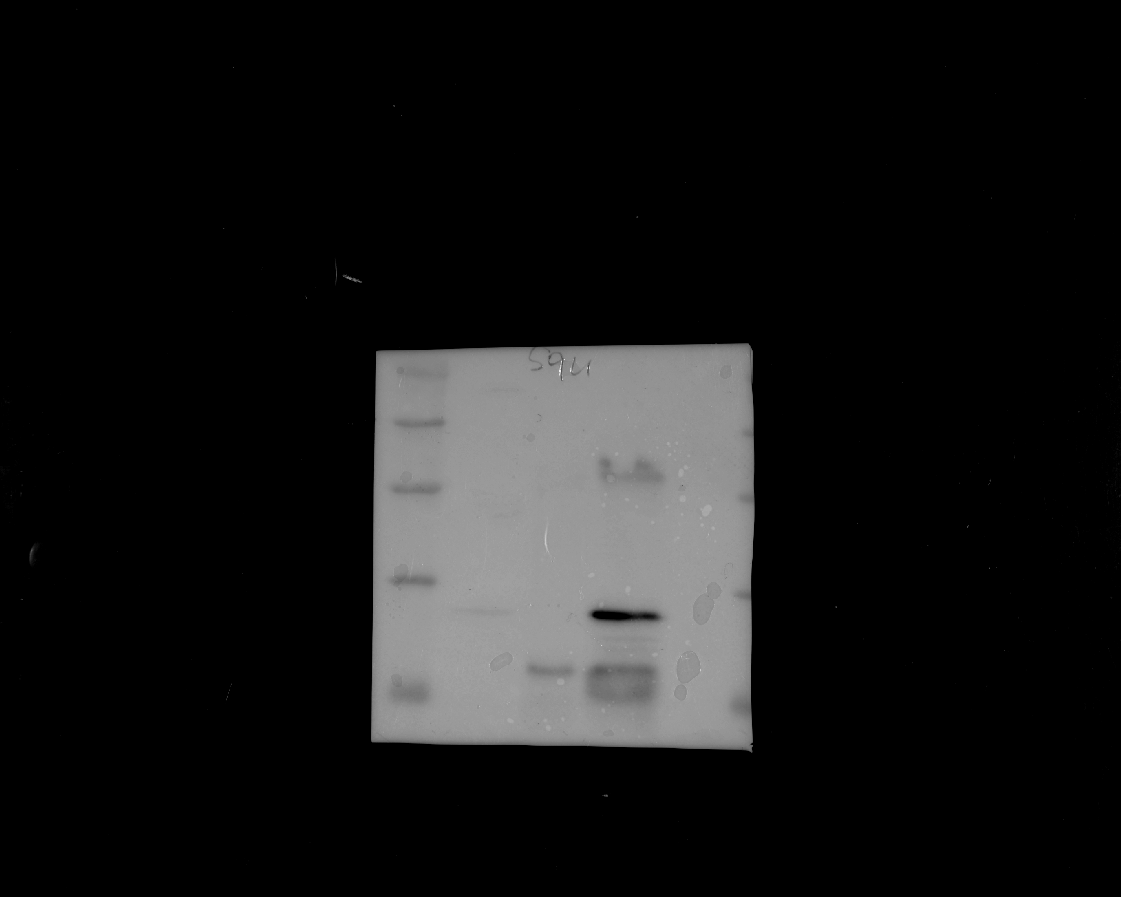

Supplement: Figure 3—source data 5. [file elife-101967-fig3-data5.zip › Figure 3-Source Data 5/Squ in Squ ip.tif]

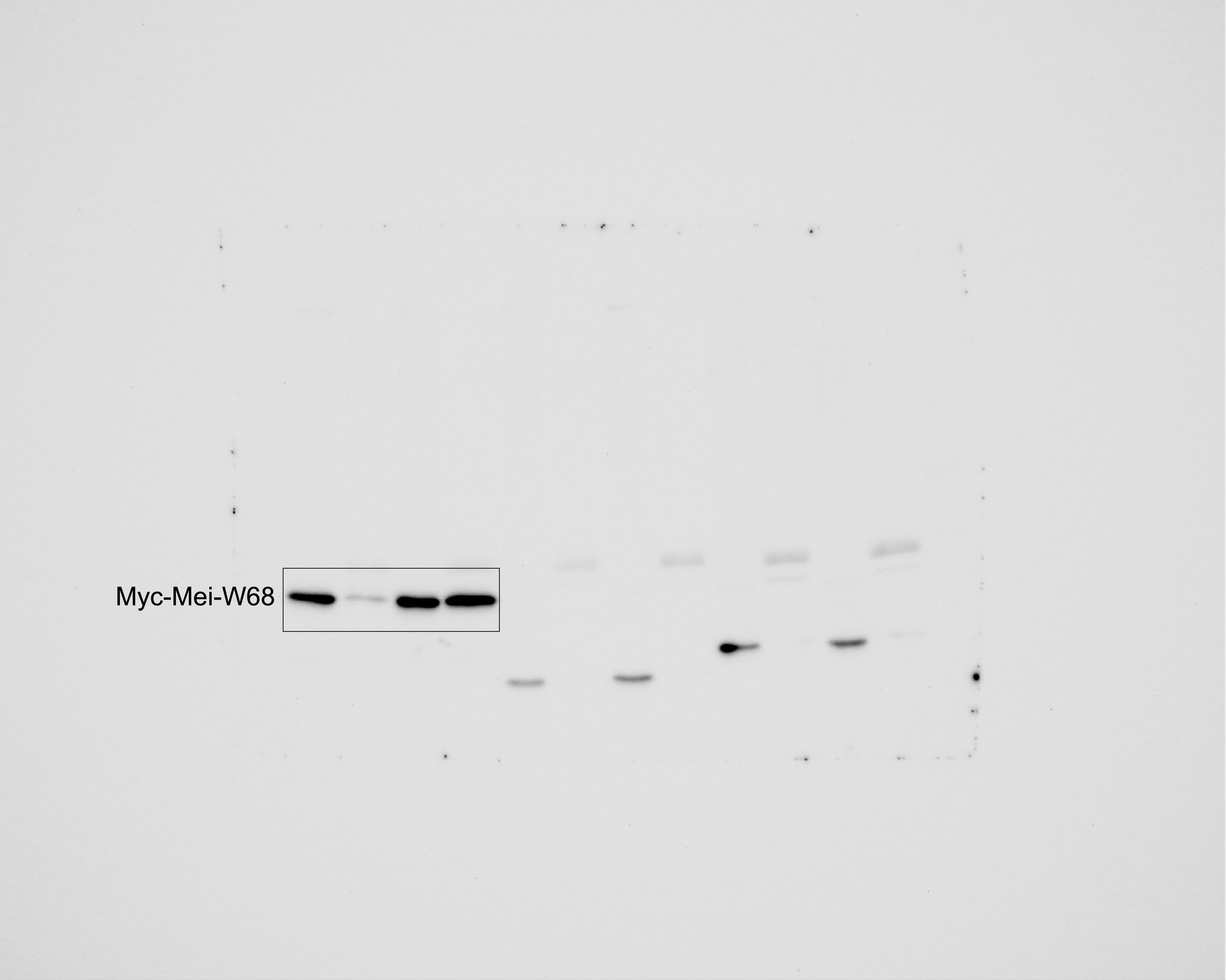

Supplement: Figure 4—source data 2. [file elife-101967-fig4-data2.zip › Figure 4-Source Data 2/Figure 4B-i_rep1_Myc_label_20230524.tiff]

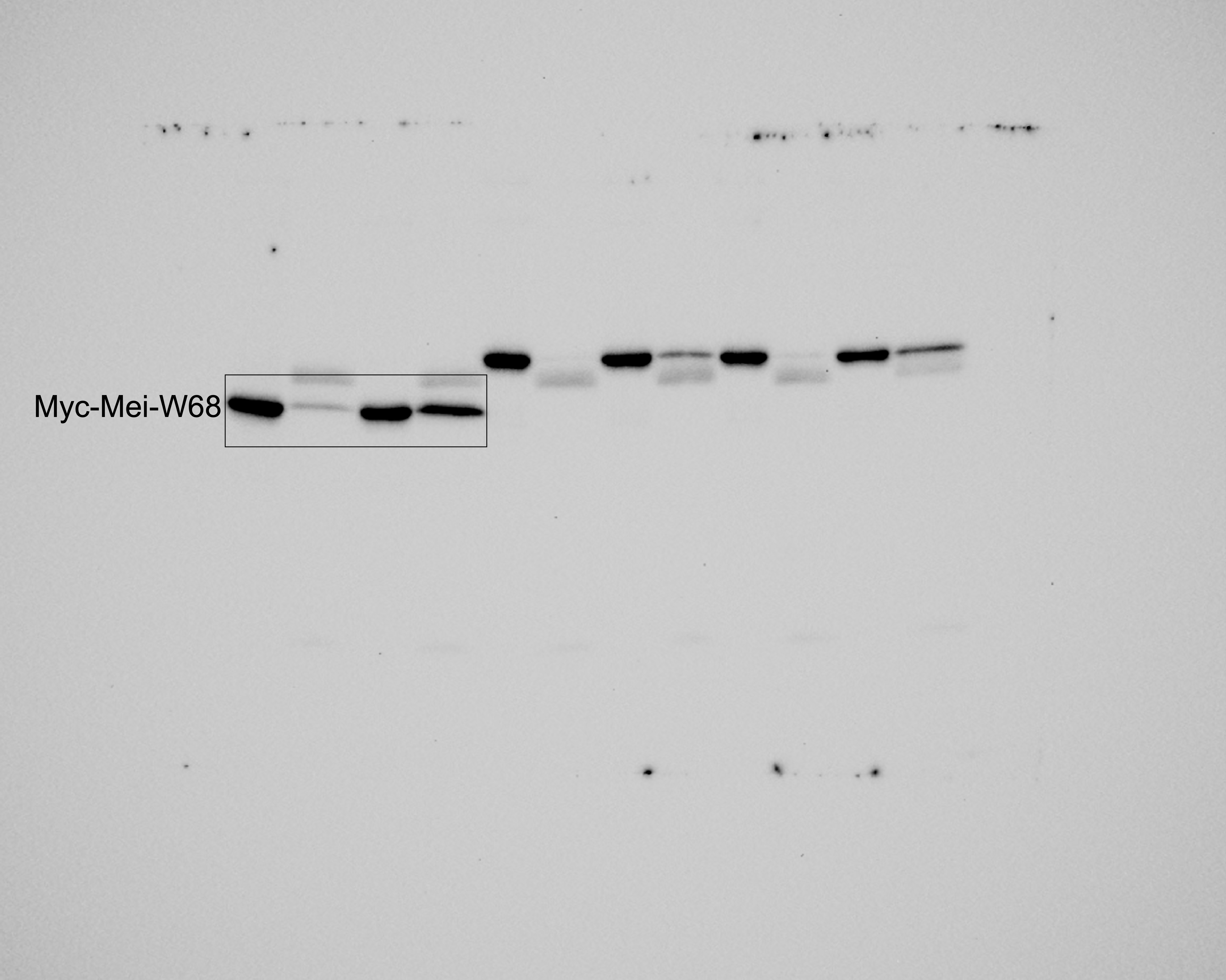

Supplement: Figure 4—source data 2. [file elife-101967-fig4-data2.zip › Figure 4-Source Data 2/Figure 4B-i_rep2_Myc_label_20230606.tiff]

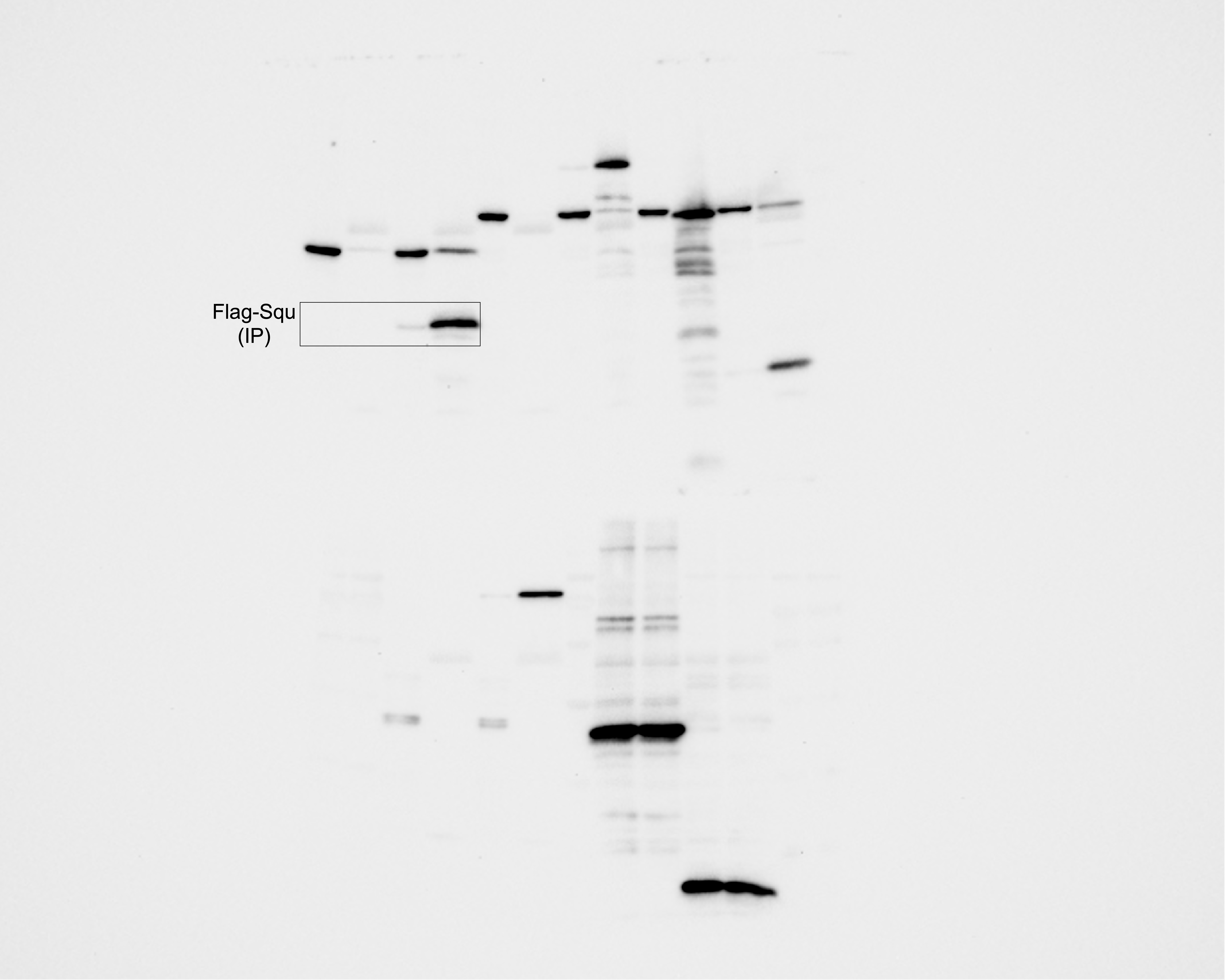

Supplement: Figure 4—source data 2. [file elife-101967-fig4-data2.zip › Figure 4-Source Data 2/Figure 4B-i_rep2_FLAG_label_20230606.tiff]

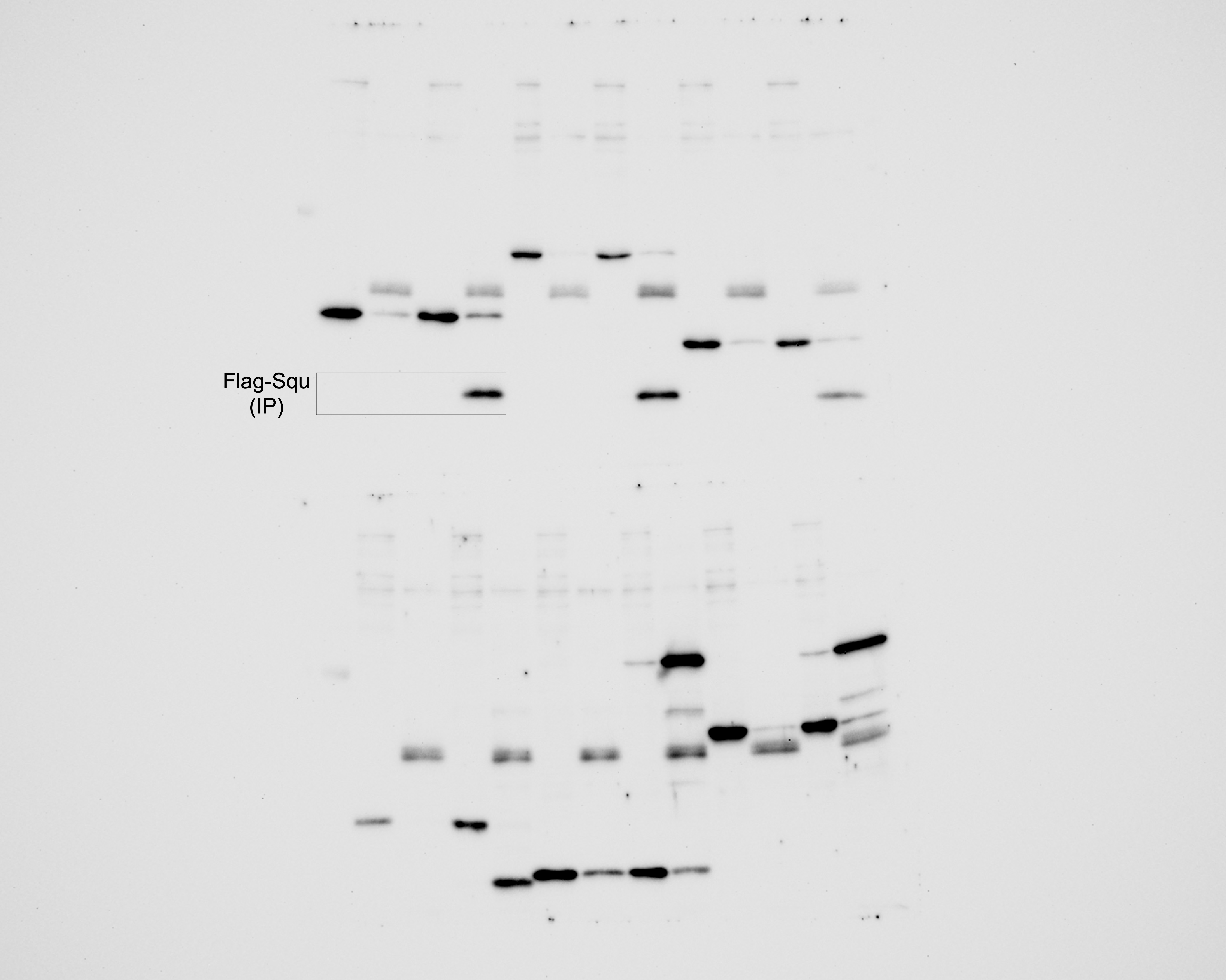

Supplement: Figure 4—source data 2. [file elife-101967-fig4-data2.zip › Figure 4-Source Data 2/Figure 4B-i_rep3_FLAG_label_20230714.tiff]

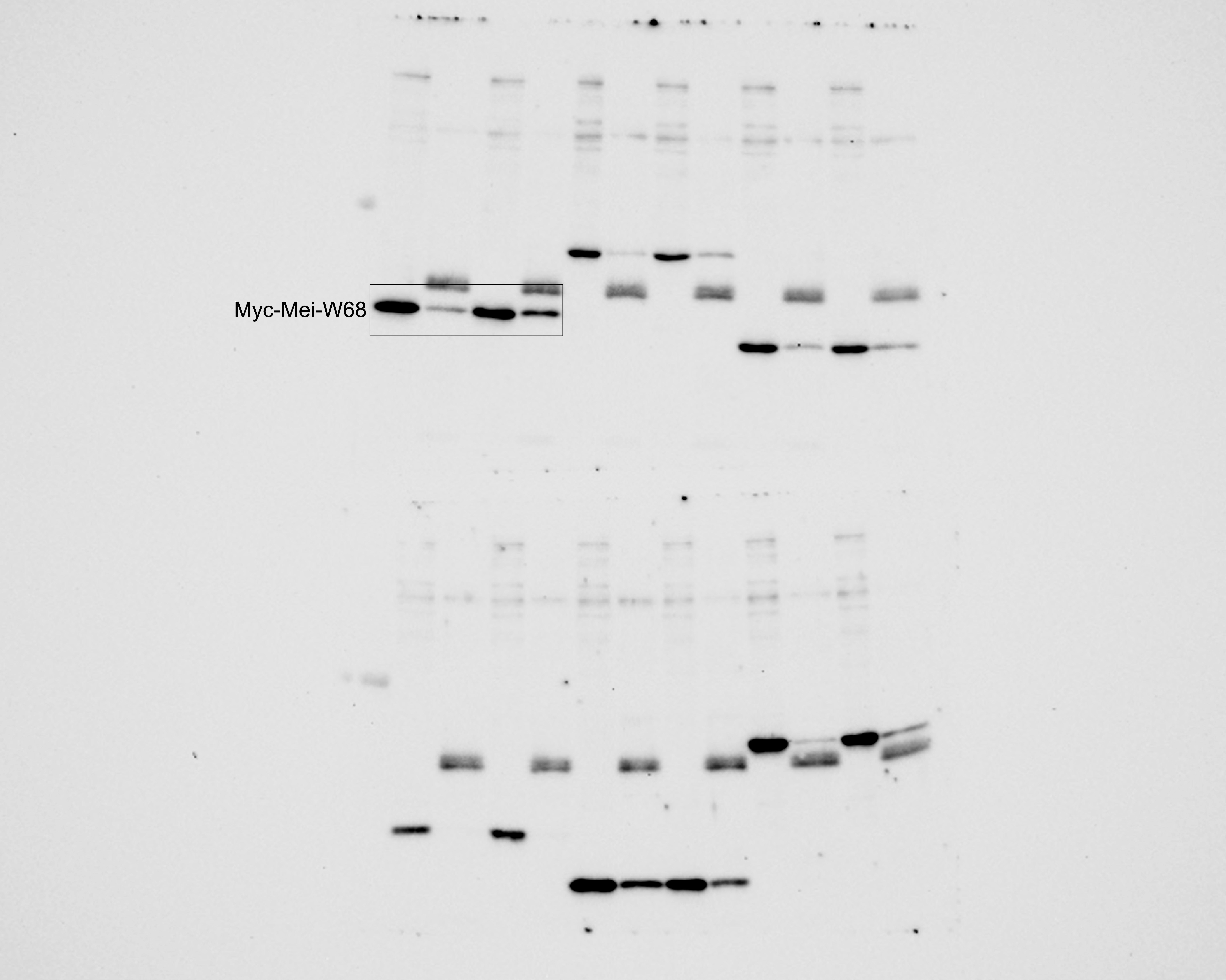

Supplement: Figure 4—source data 2. [file elife-101967-fig4-data2.zip › Figure 4-Source Data 2/Figure 4B-i_rep3_Myc_label_20230714.tiff]

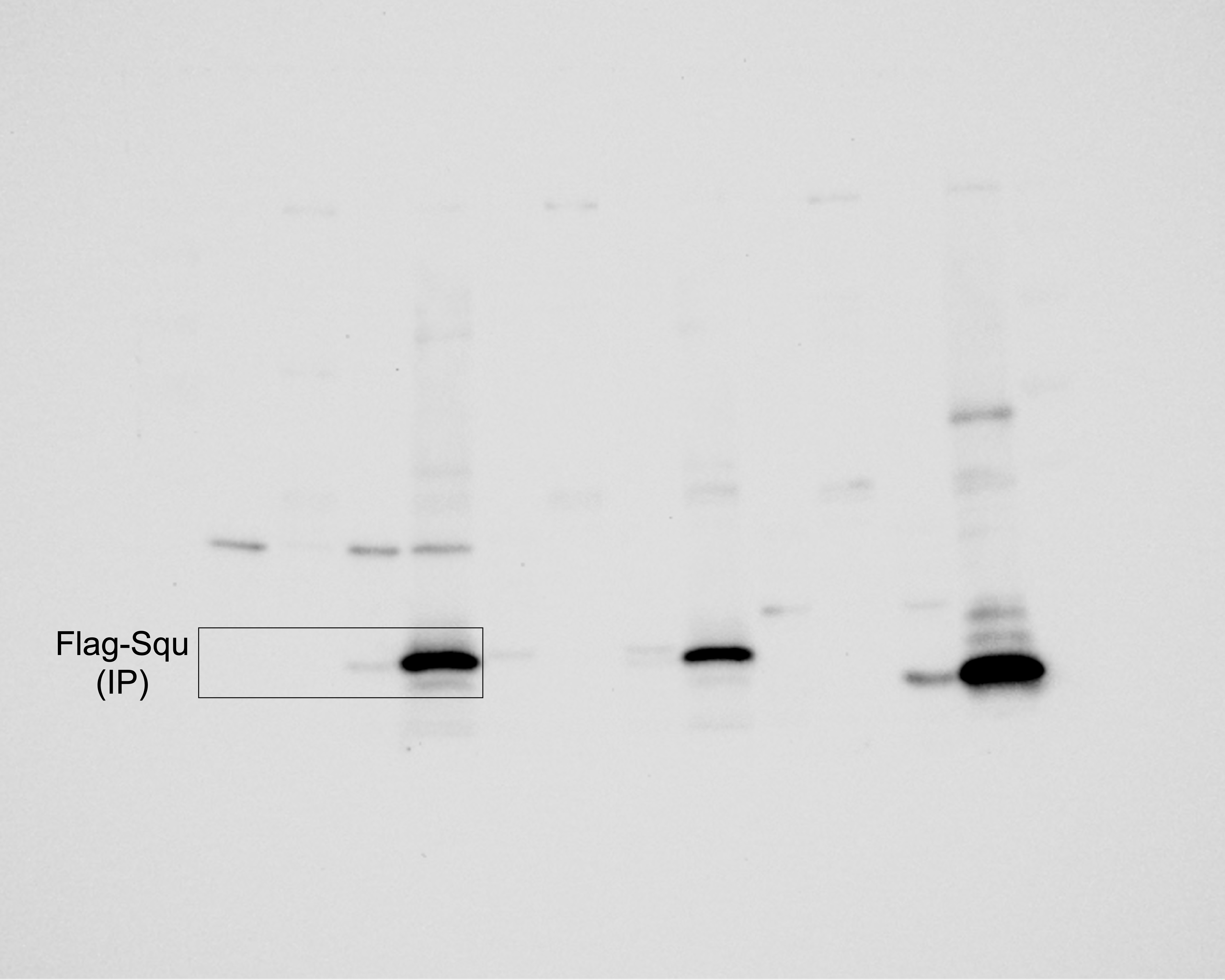

Supplement: Figure 4—source data 2. [file elife-101967-fig4-data2.zip › Figure 4-Source Data 2/Figure 4B-i_rep1_FLAG_label_20230524.tiff]

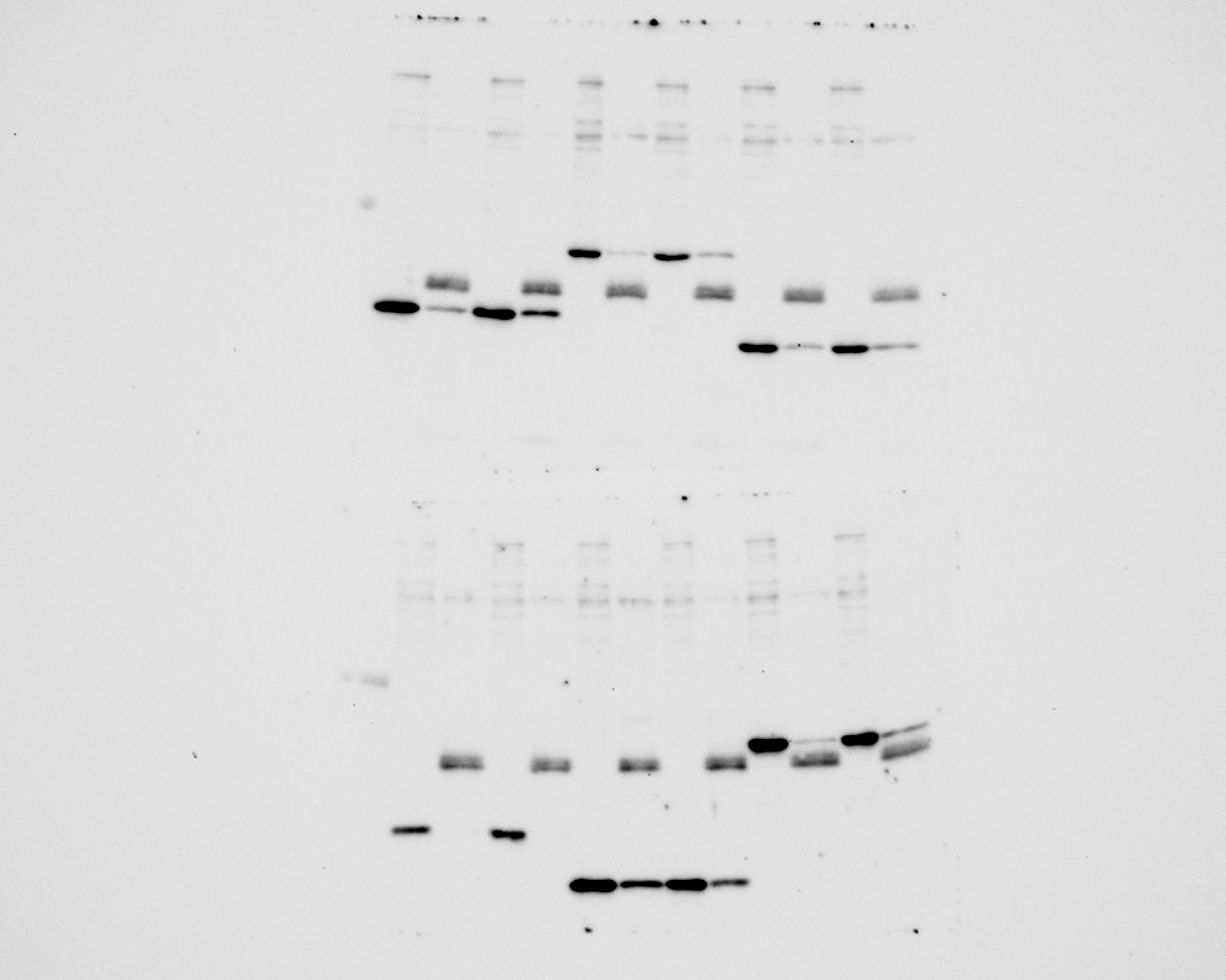

Supplement: Figure 4—source data 3. [file elife-101967-fig4-data3.zip › Figure 4-Source Data 3/Figure 4B-i_rep3_Myc_original_20230714.tif]

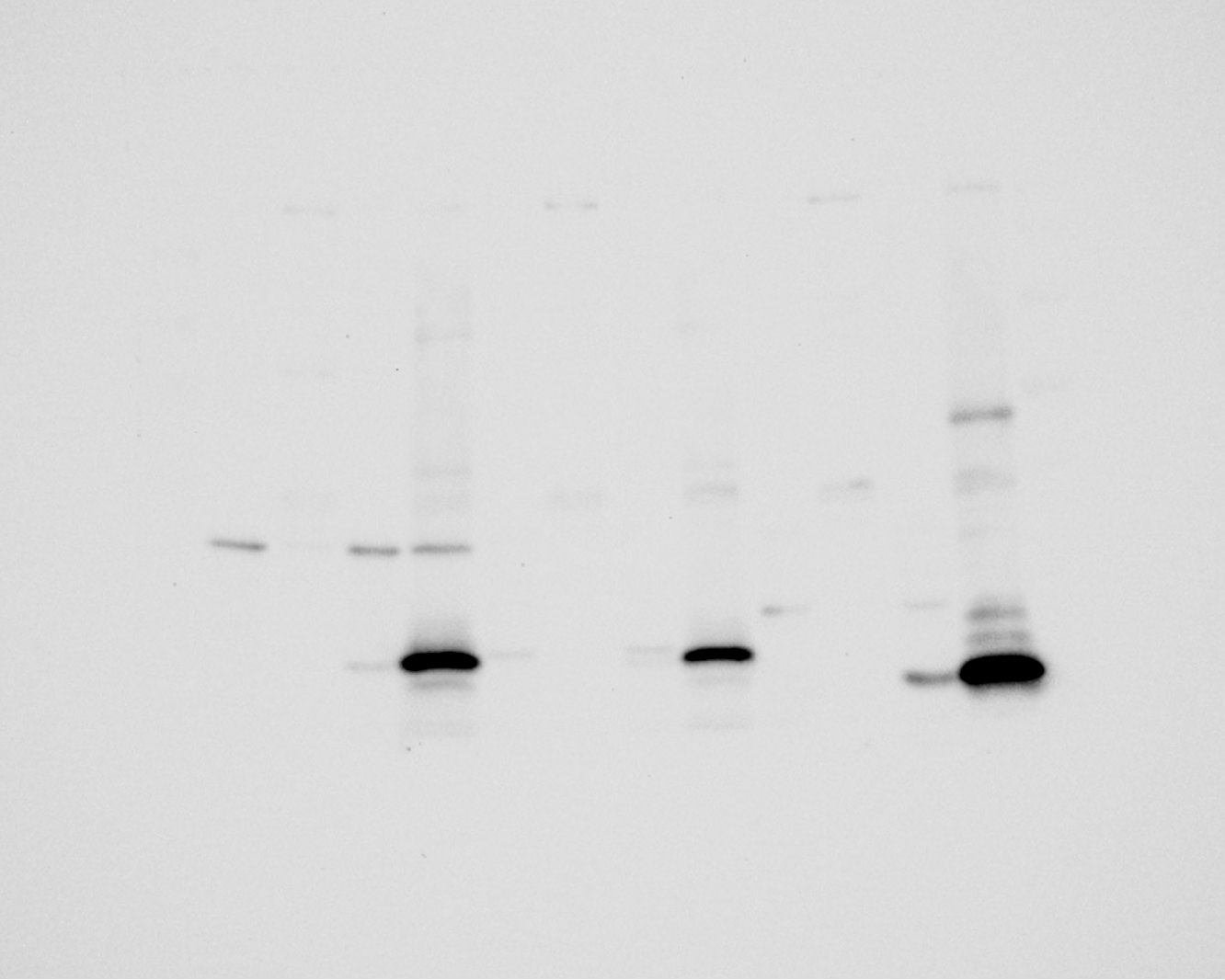

Supplement: Figure 4—source data 3. [file elife-101967-fig4-data3.zip › Figure 4-Source Data 3/Figure 4B-i_rep1_FLAG_original_20230524.tif]

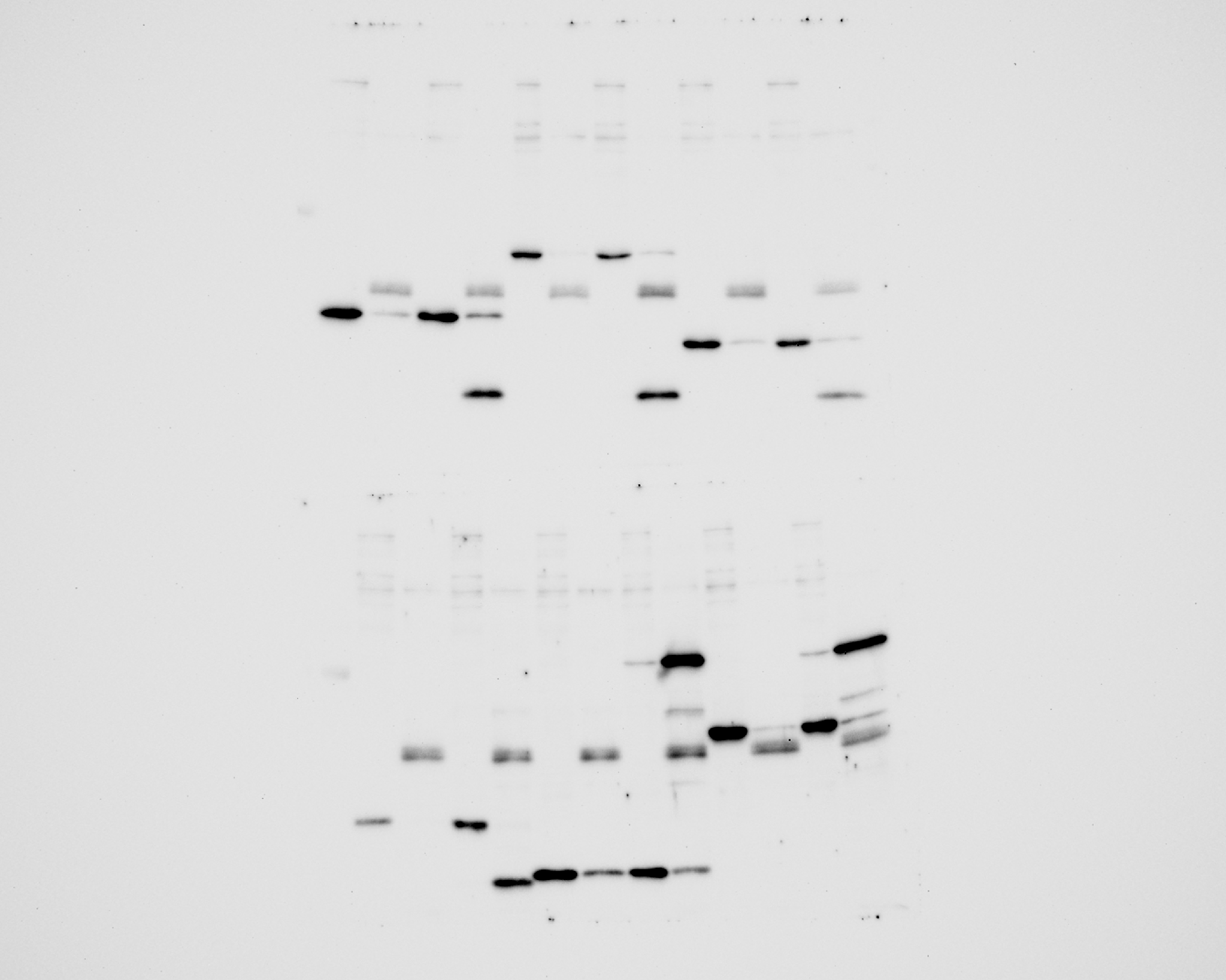

Supplement: Figure 4—source data 3. [file elife-101967-fig4-data3.zip › Figure 4-Source Data 3/Figure 4B-i_rep3_FLAG_original_20230714.tif]

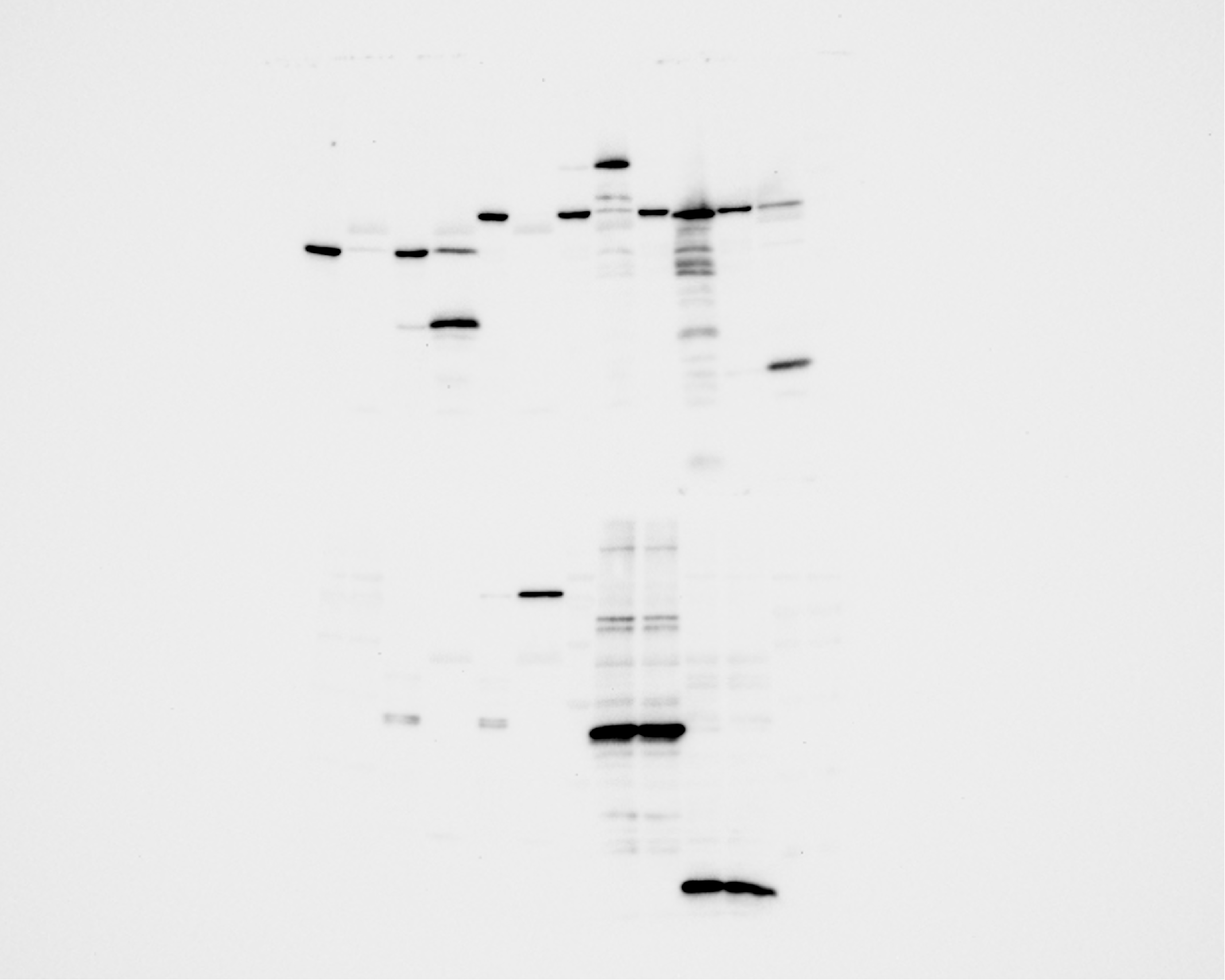

Supplement: Figure 4—source data 3. [file elife-101967-fig4-data3.zip › Figure 4-Source Data 3/Figure 4B-i_rep2_FLAG_original_20230606.tif]

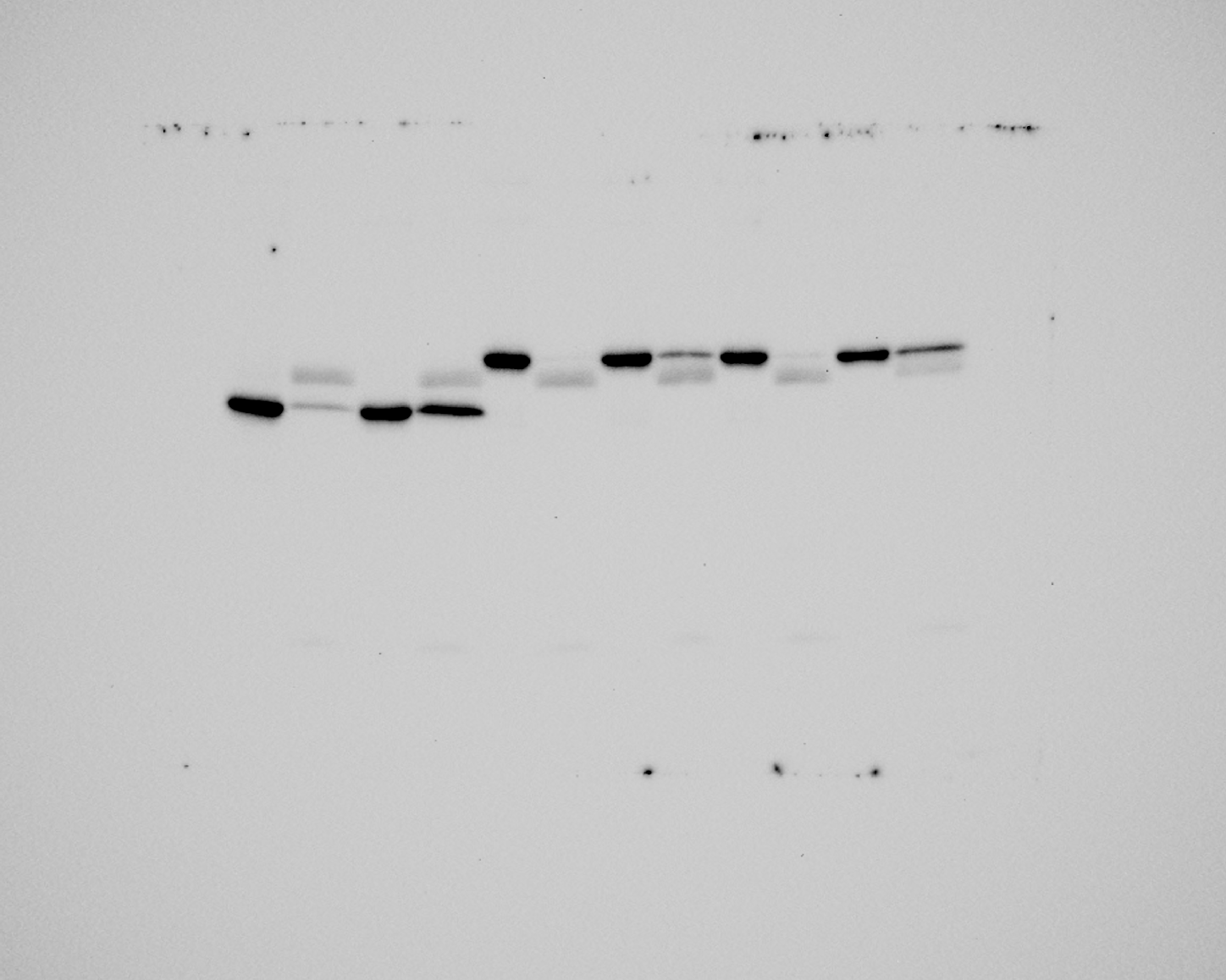

Supplement: Figure 4—source data 3. [file elife-101967-fig4-data3.zip › Figure 4-Source Data 3/Figure 4B-i_rep2_Myc_original_20230606.tif]

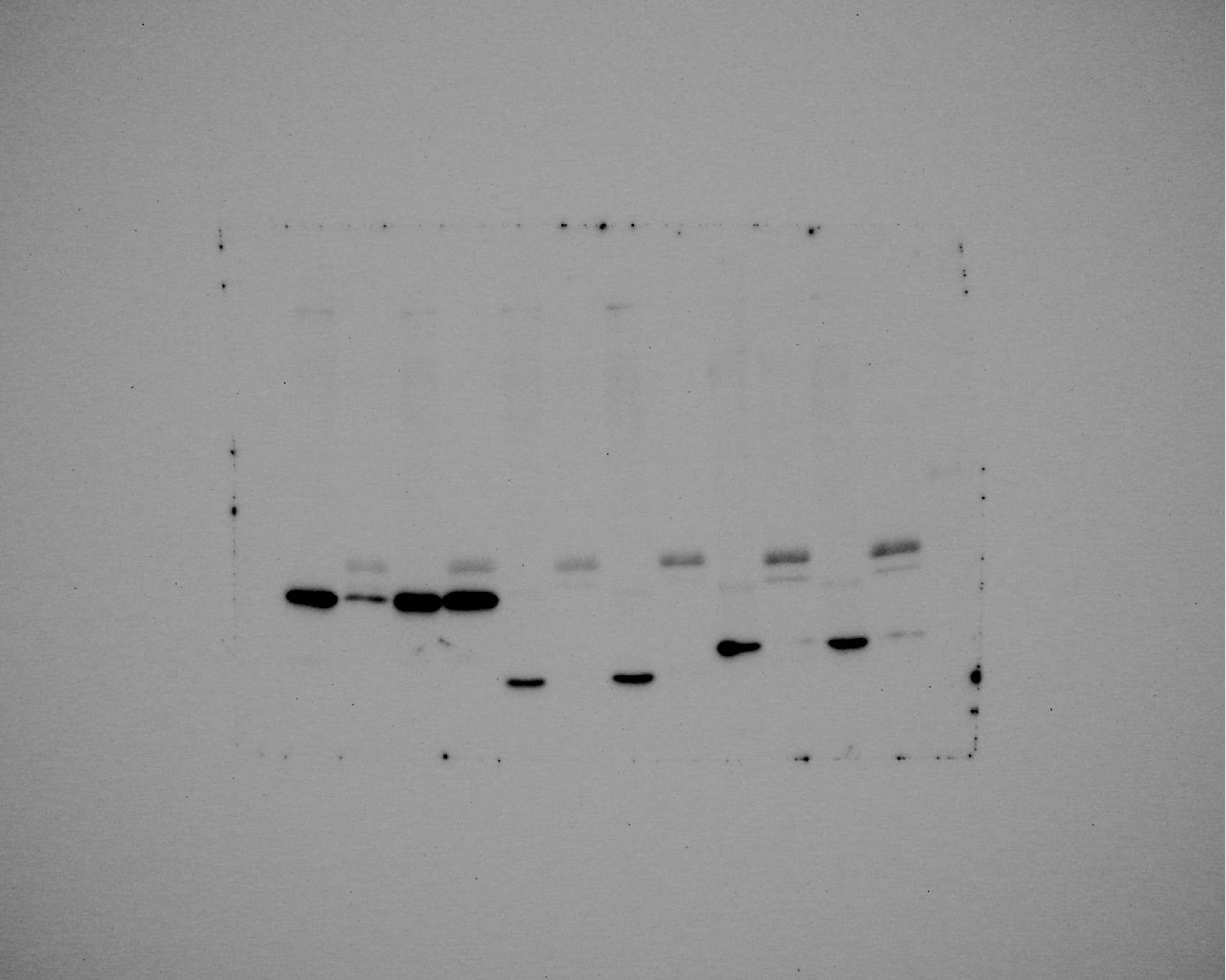

Supplement: Figure 4—source data 3. [file elife-101967-fig4-data3.zip › Figure 4-Source Data 3/Figure 4B-i_rep1_Myc_original_20230524.tif]

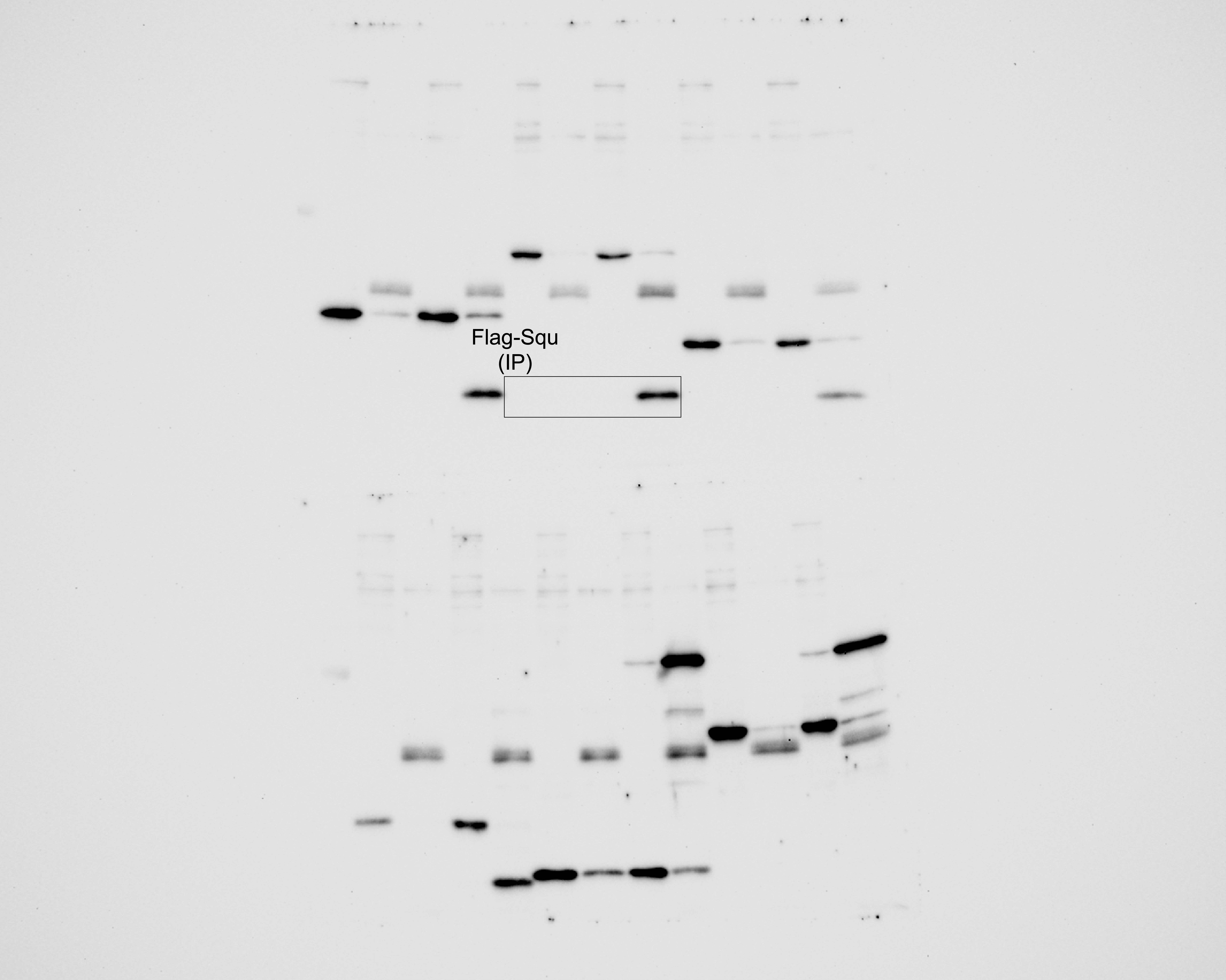

Supplement: Figure 4—source data 4. [file elife-101967-fig4-data4.zip › Figure 4-Source Data 4/Figure 4B-ii_rep3_FLAG_label_20230714.tiff]

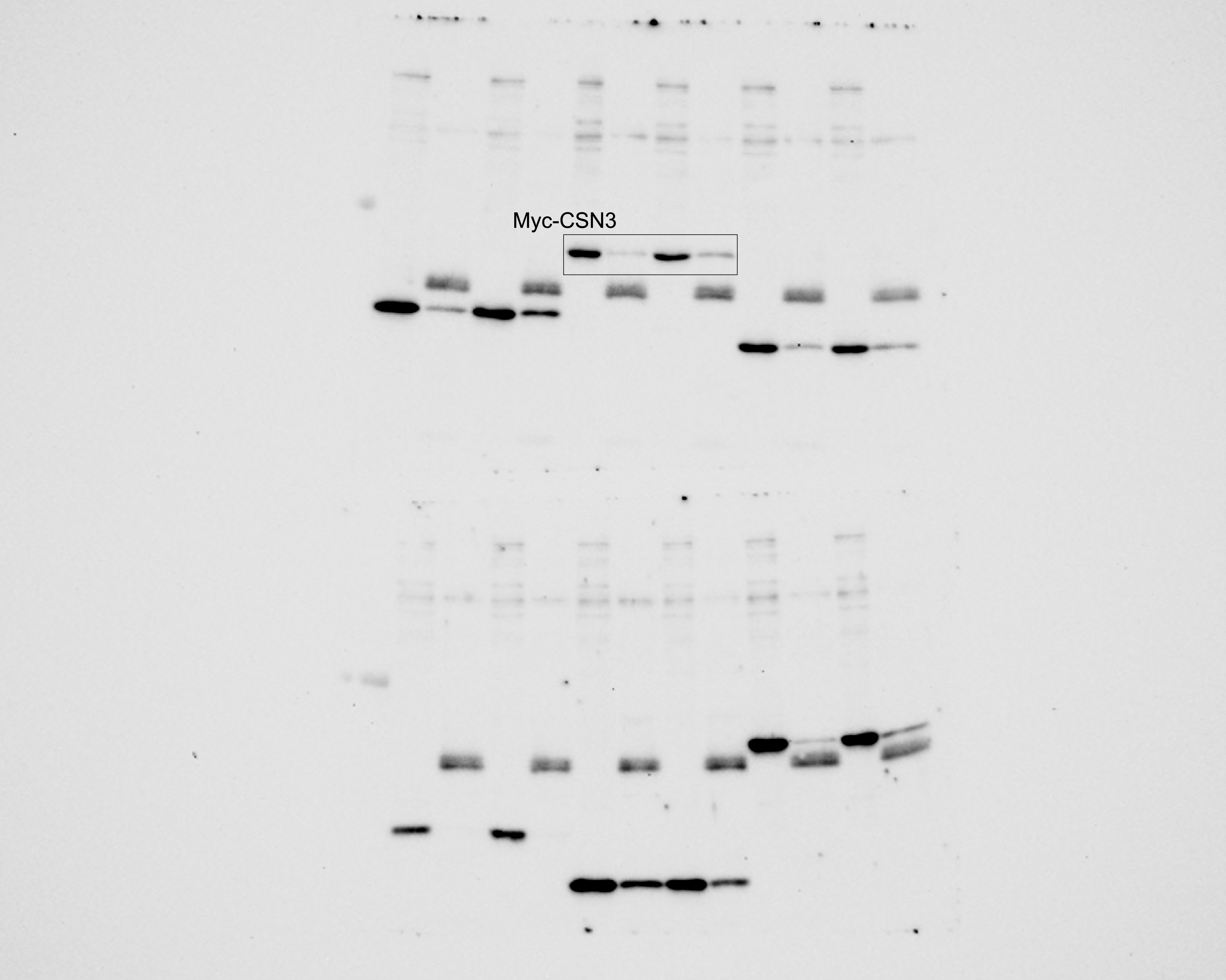

Supplement: Figure 4—source data 4. [file elife-101967-fig4-data4.zip › Figure 4-Source Data 4/Figure 4B-ii_rep3_Myc_label_20230714.tiff]

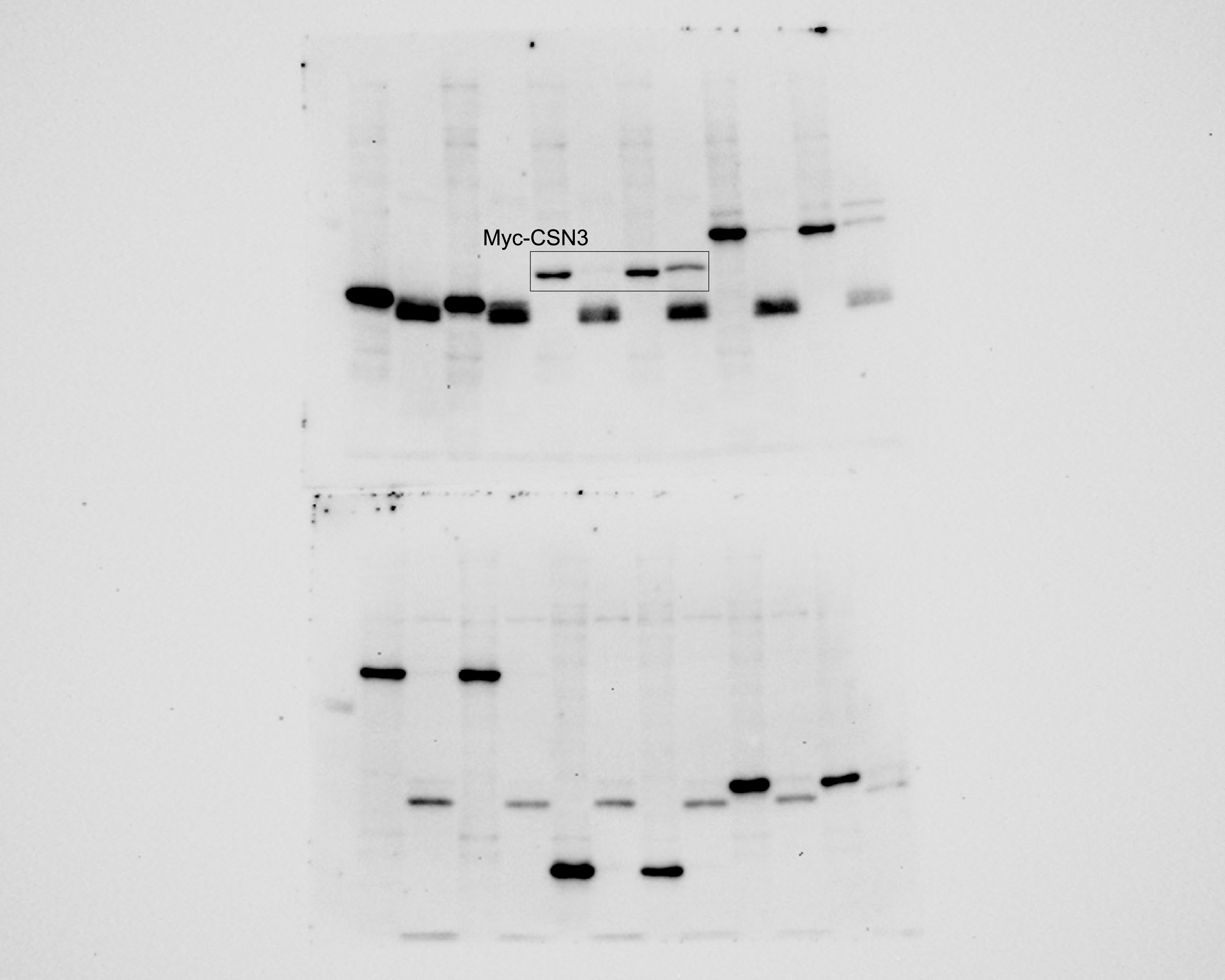

Supplement: Figure 4—source data 4. [file elife-101967-fig4-data4.zip › Figure 4-Source Data 4/Figure 4B-ii_rep2_Myc_label_20230623.tiff]

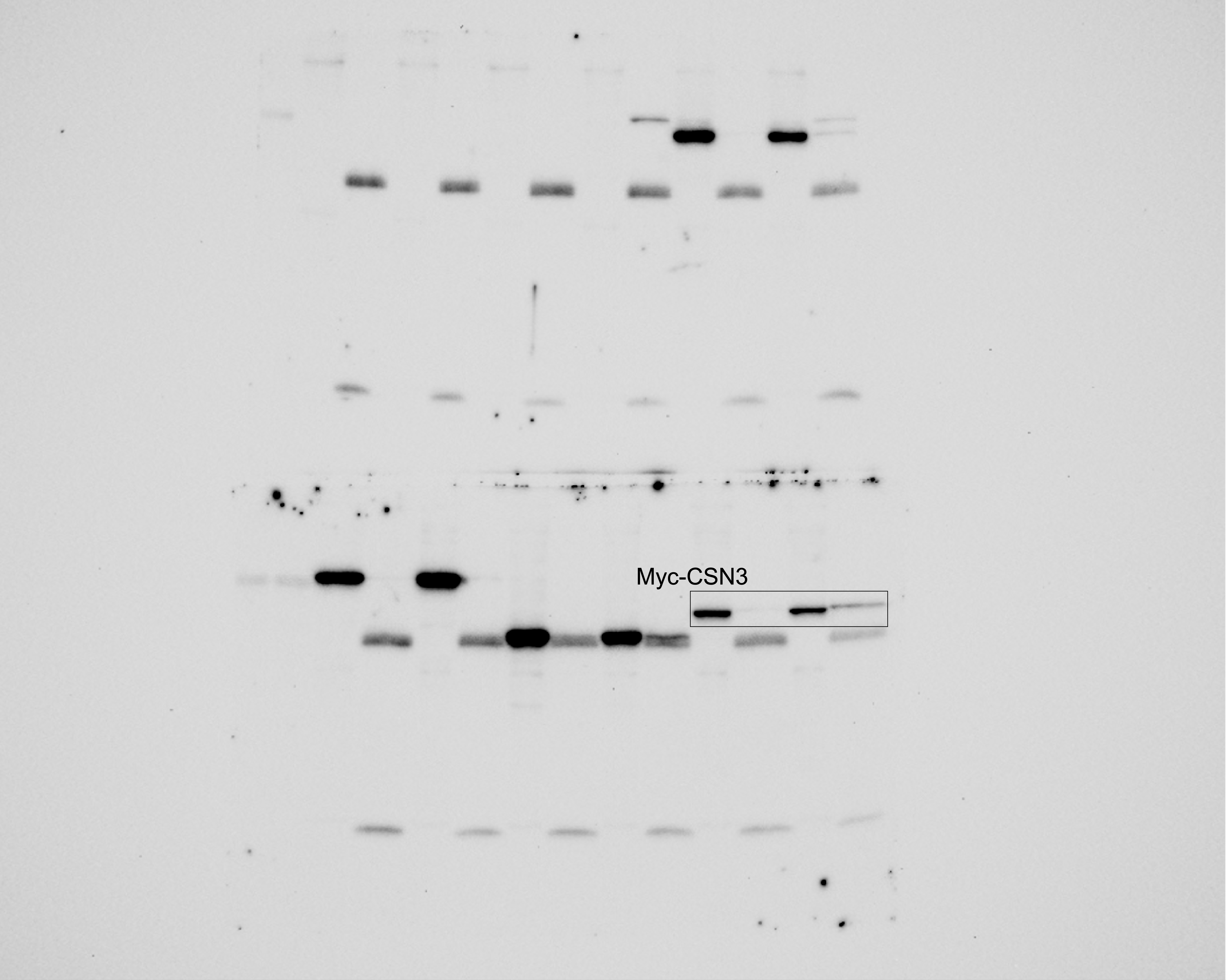

Supplement: Figure 4—source data 4. [file elife-101967-fig4-data4.zip › Figure 4-Source Data 4/Figure 4B-ii_rep1_Myc_label_20230613.tiff]

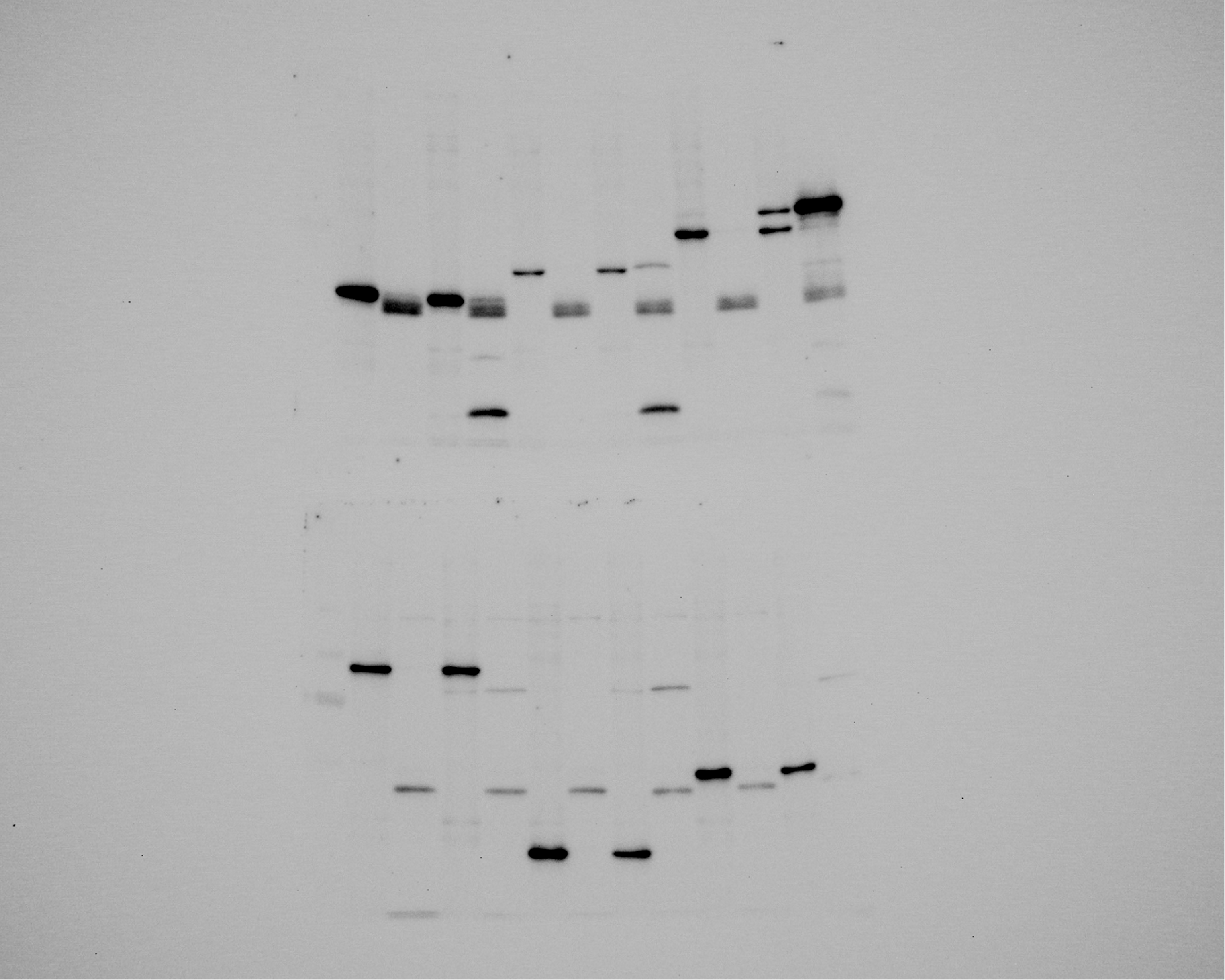

Supplement: Figure 4—source data 5. [file elife-101967-fig4-data5.zip › Figure 4-Source Data 5/Figure 4B-ii_rep2_FLAG_original_20230623.tif]

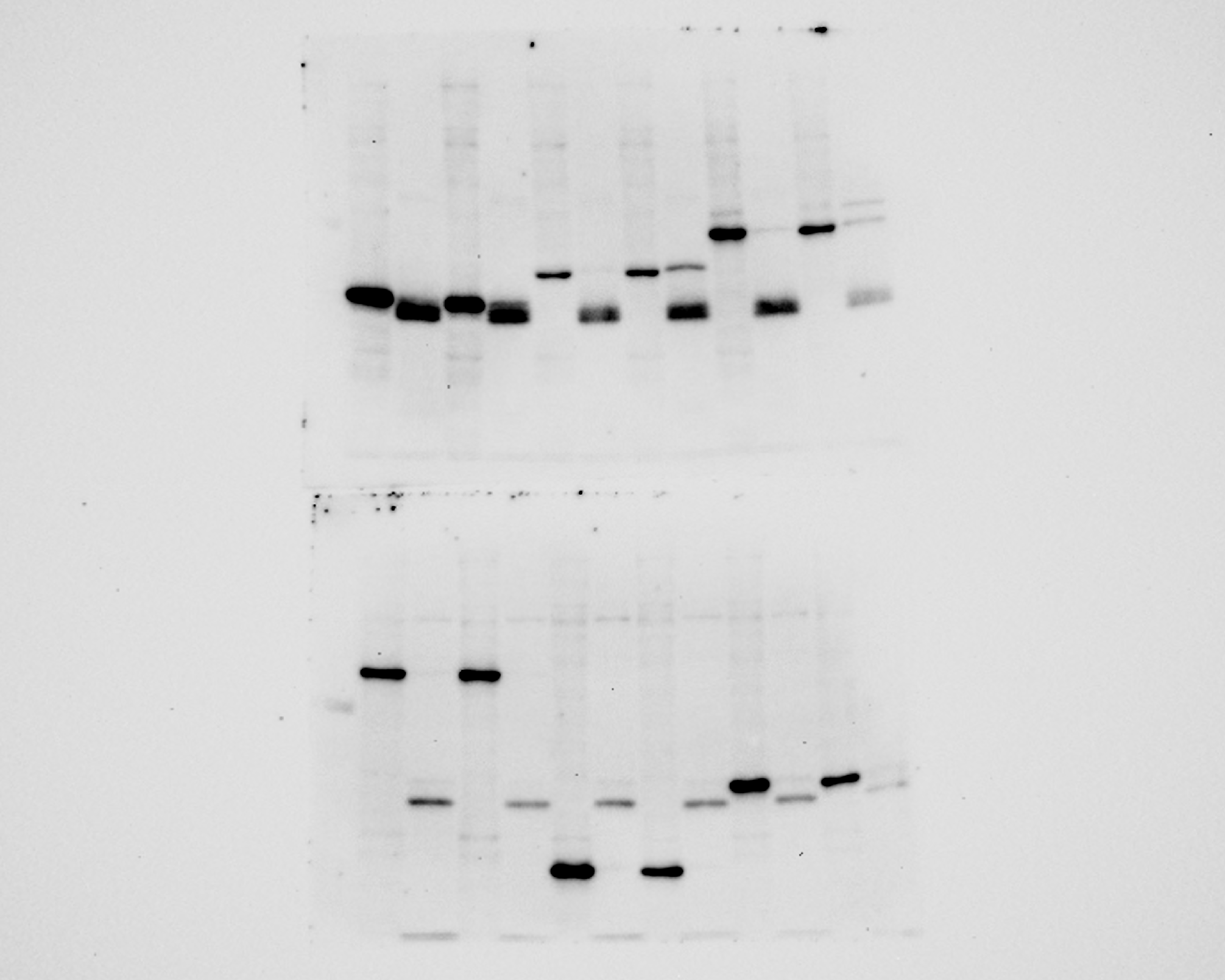

Supplement: Figure 4—source data 5. [file elife-101967-fig4-data5.zip › Figure 4-Source Data 5/Figure 4B-ii_rep2_Myc_original_20230623.tif]

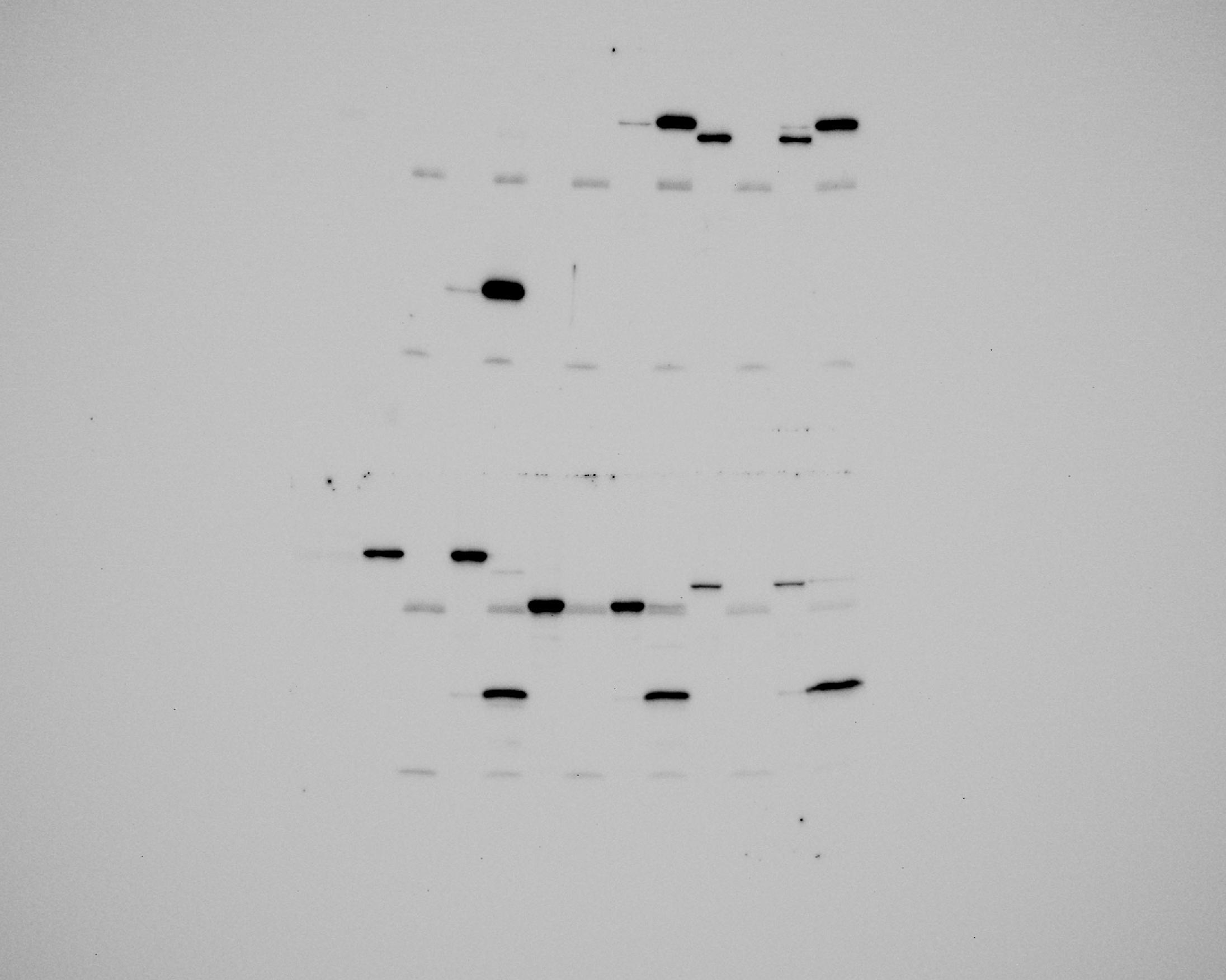

Supplement: Figure 4—source data 5. [file elife-101967-fig4-data5.zip › Figure 4-Source Data 5/Figure 4B-ii_rep1_FLAG_original_20230613.tif]

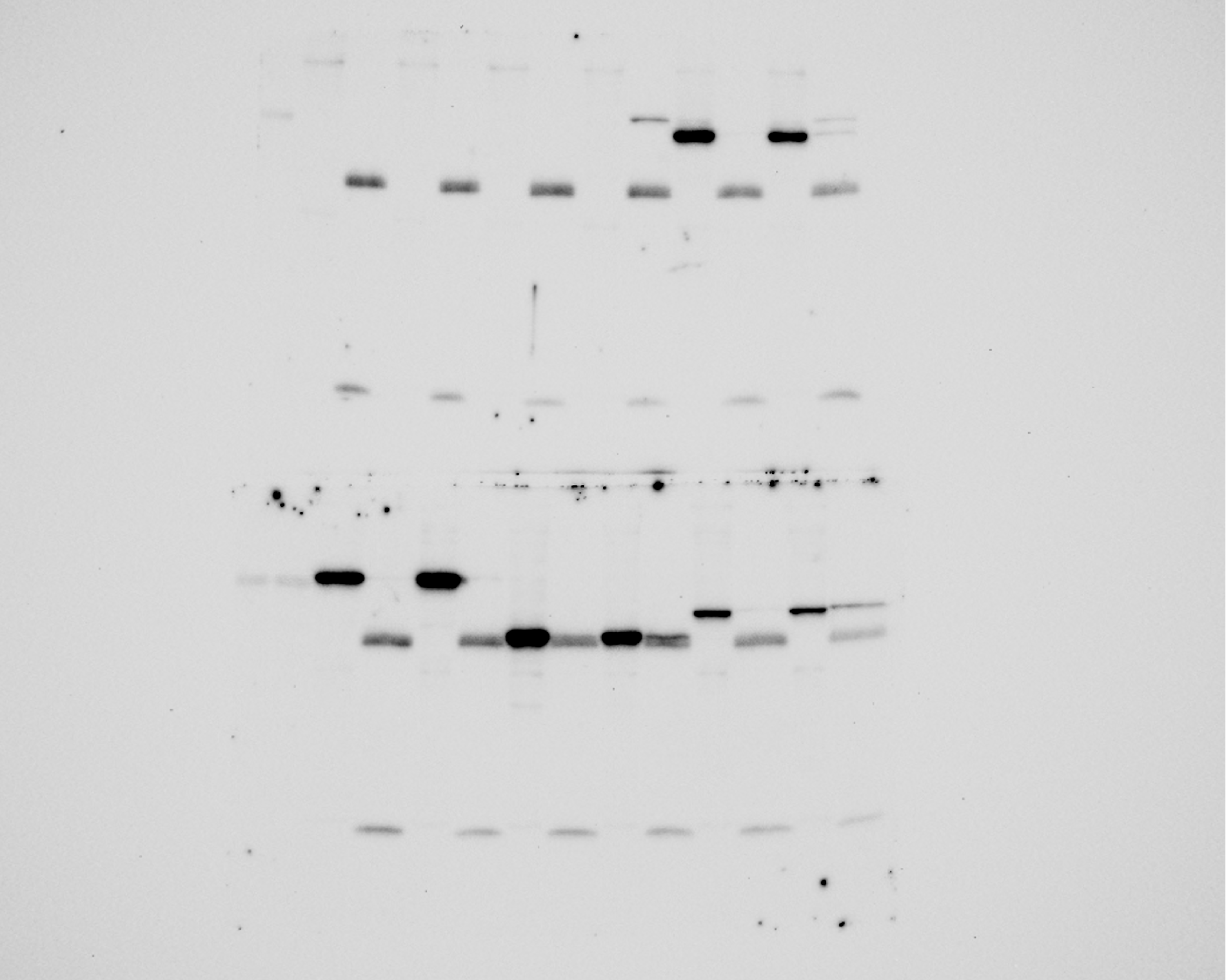

Supplement: Figure 4—source data 5. [file elife-101967-fig4-data5.zip › Figure 4-Source Data 5/Figure 4B-ii_rep1_Myc_original_20230613.tif]

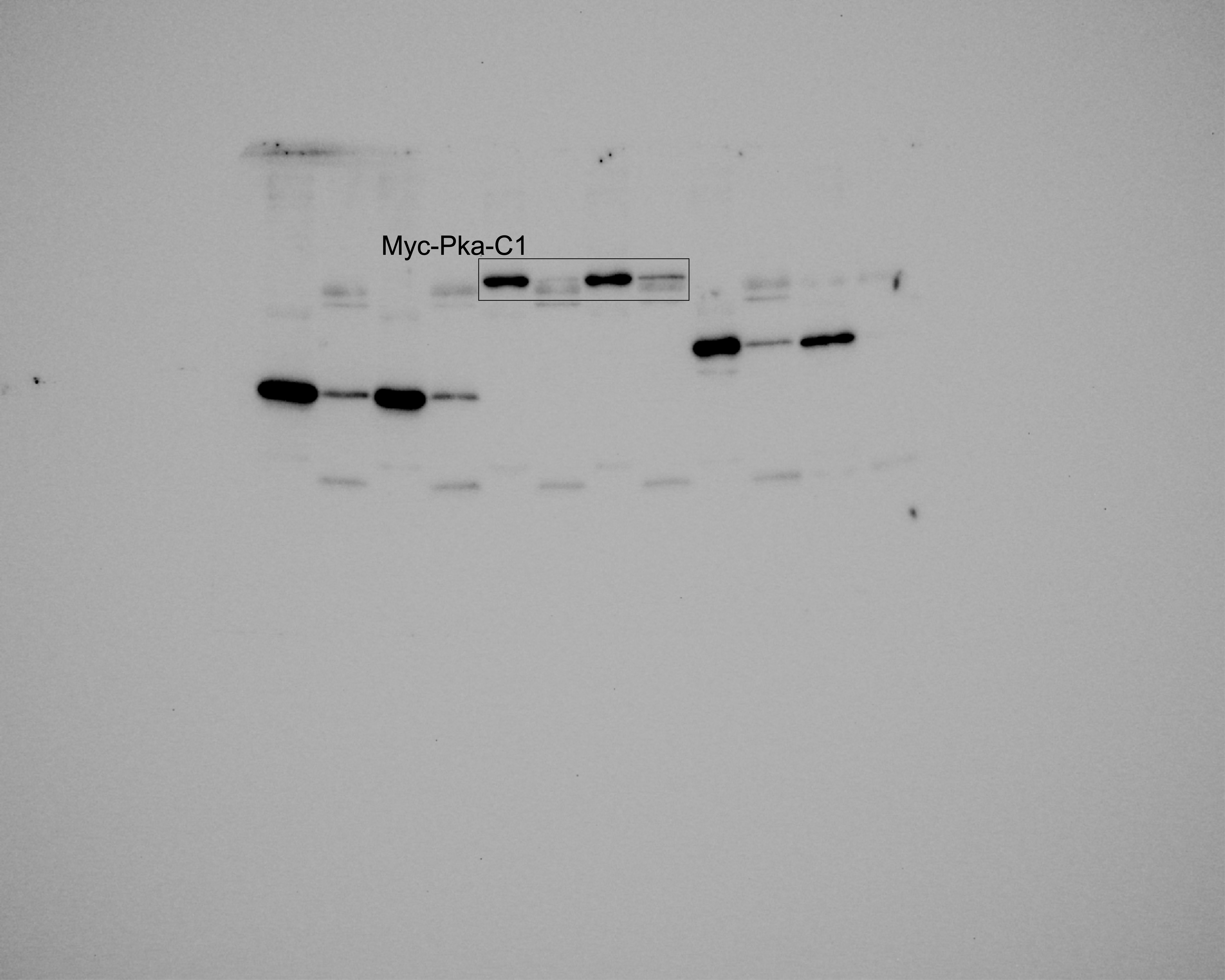

Supplement: Figure 4—source data 6. [file elife-101967-fig4-data6.zip › Figure 4-Source Data 6/Figure 4B-iii_rep1_Myc_label_20230519.tiff]

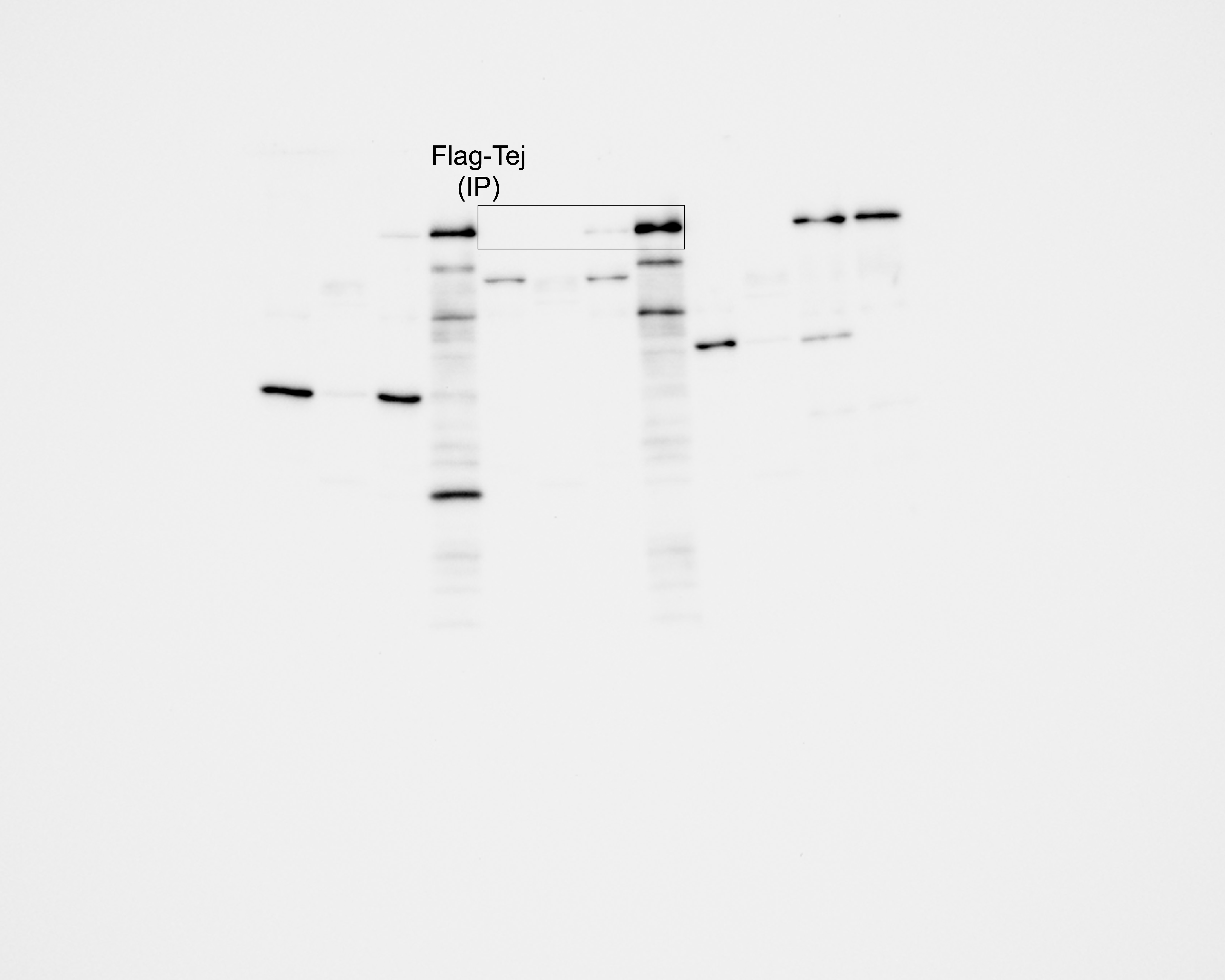

Supplement: Figure 4—source data 6. [file elife-101967-fig4-data6.zip › Figure 4-Source Data 6/Figure 4B-iii_rep1_FLAG_label_20230519.tiff]

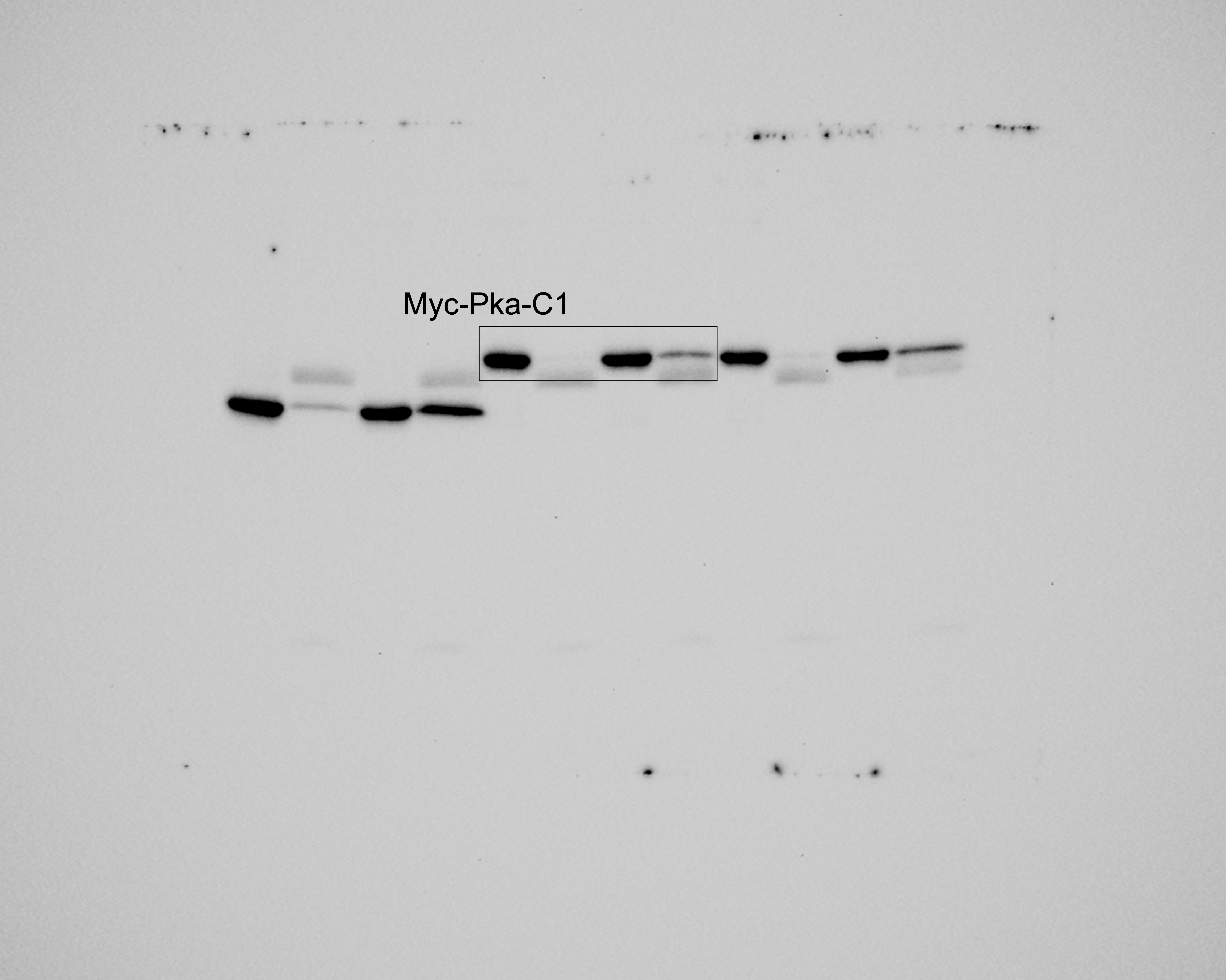

Supplement: Figure 4—source data 6. [file elife-101967-fig4-data6.zip › Figure 4-Source Data 6/Figure 4B-iii_rep2_Myc_label_20230606.tiff]

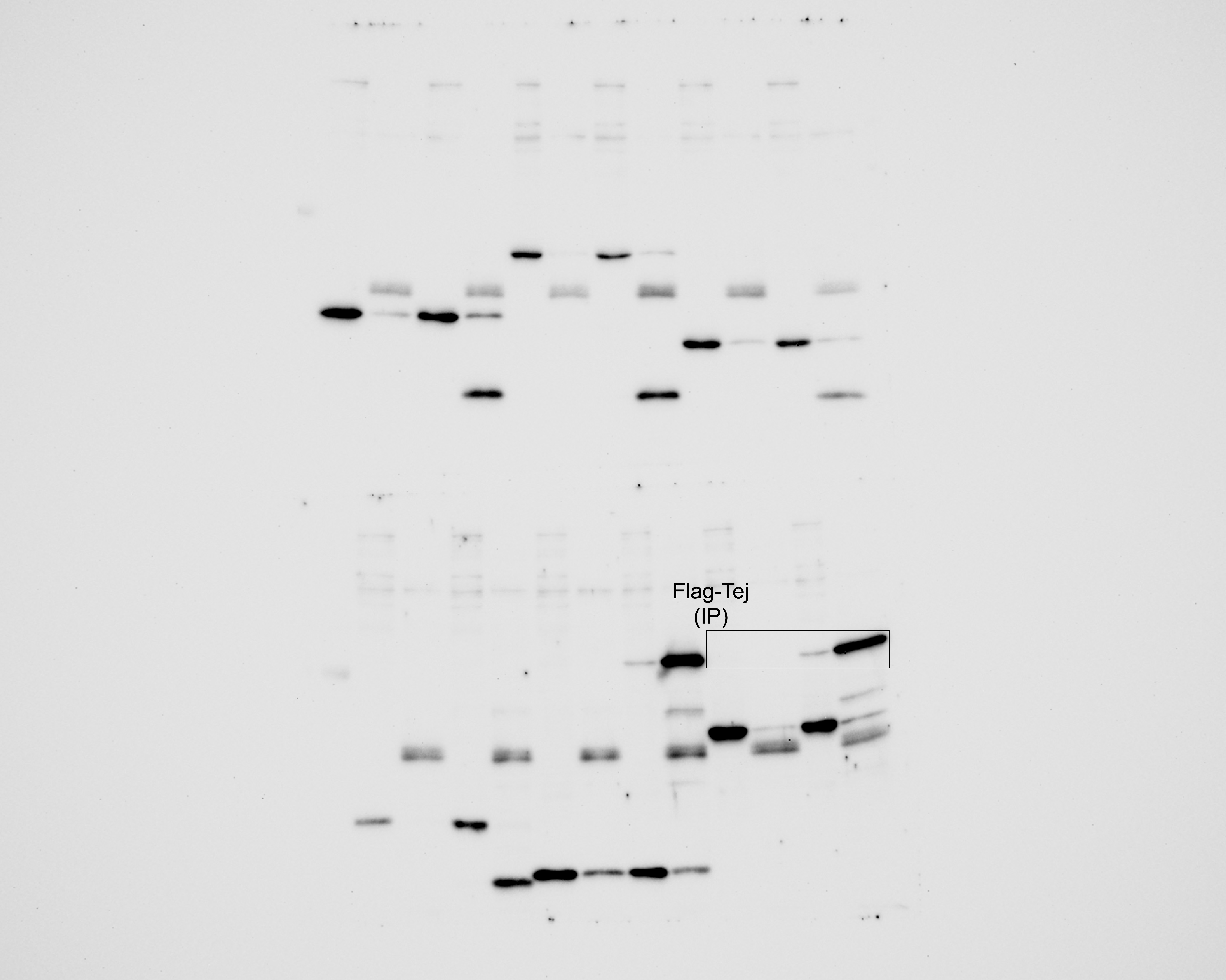

Supplement: Figure 4—source data 6. [file elife-101967-fig4-data6.zip › Figure 4-Source Data 6/Figure 4B-iii_rep3_FLAG_label_20230714.tiff]

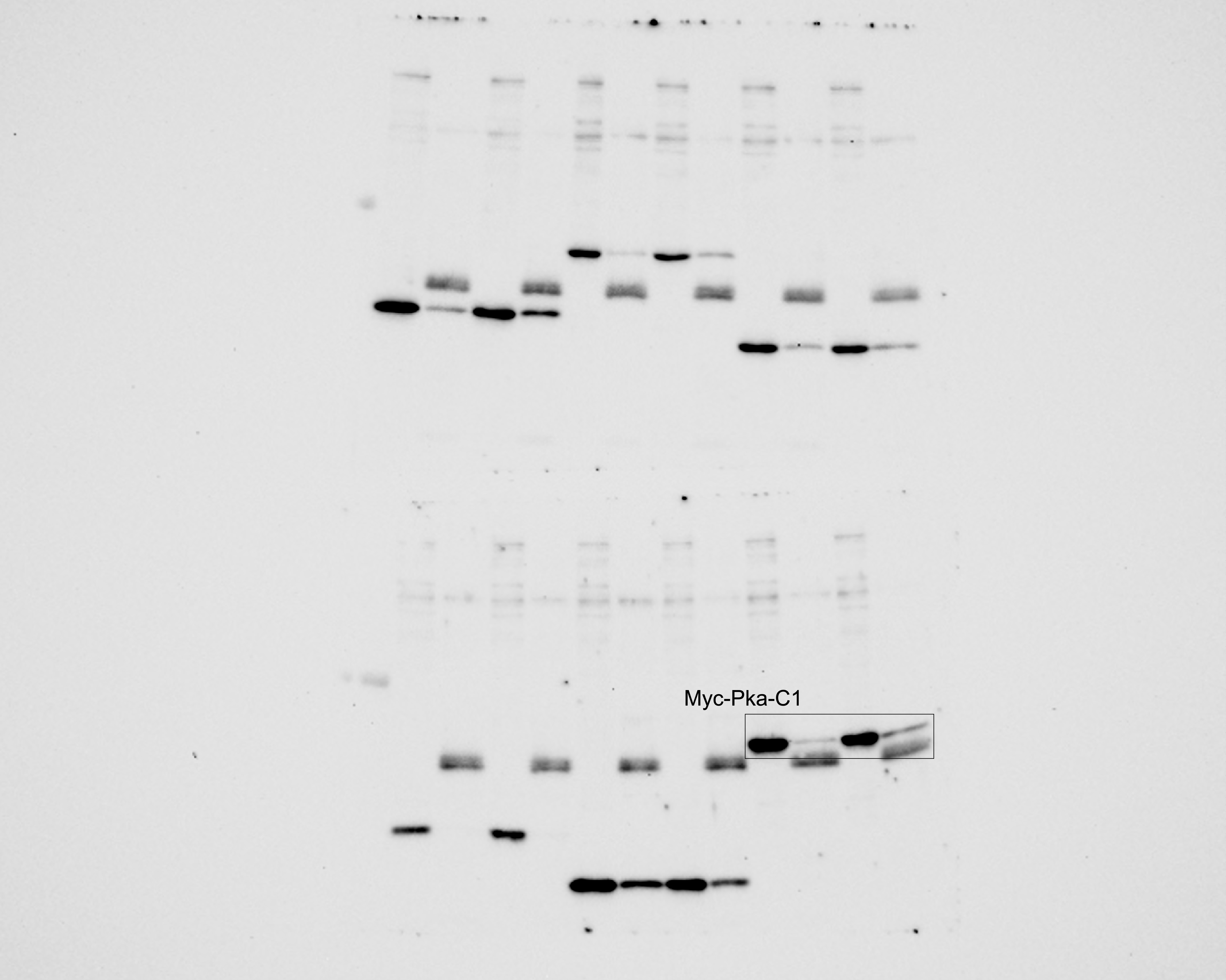

Supplement: Figure 4—source data 6. [file elife-101967-fig4-data6.zip › Figure 4-Source Data 6/Figure 4B-iii_rep3_Myc_label_20230714.tiff]

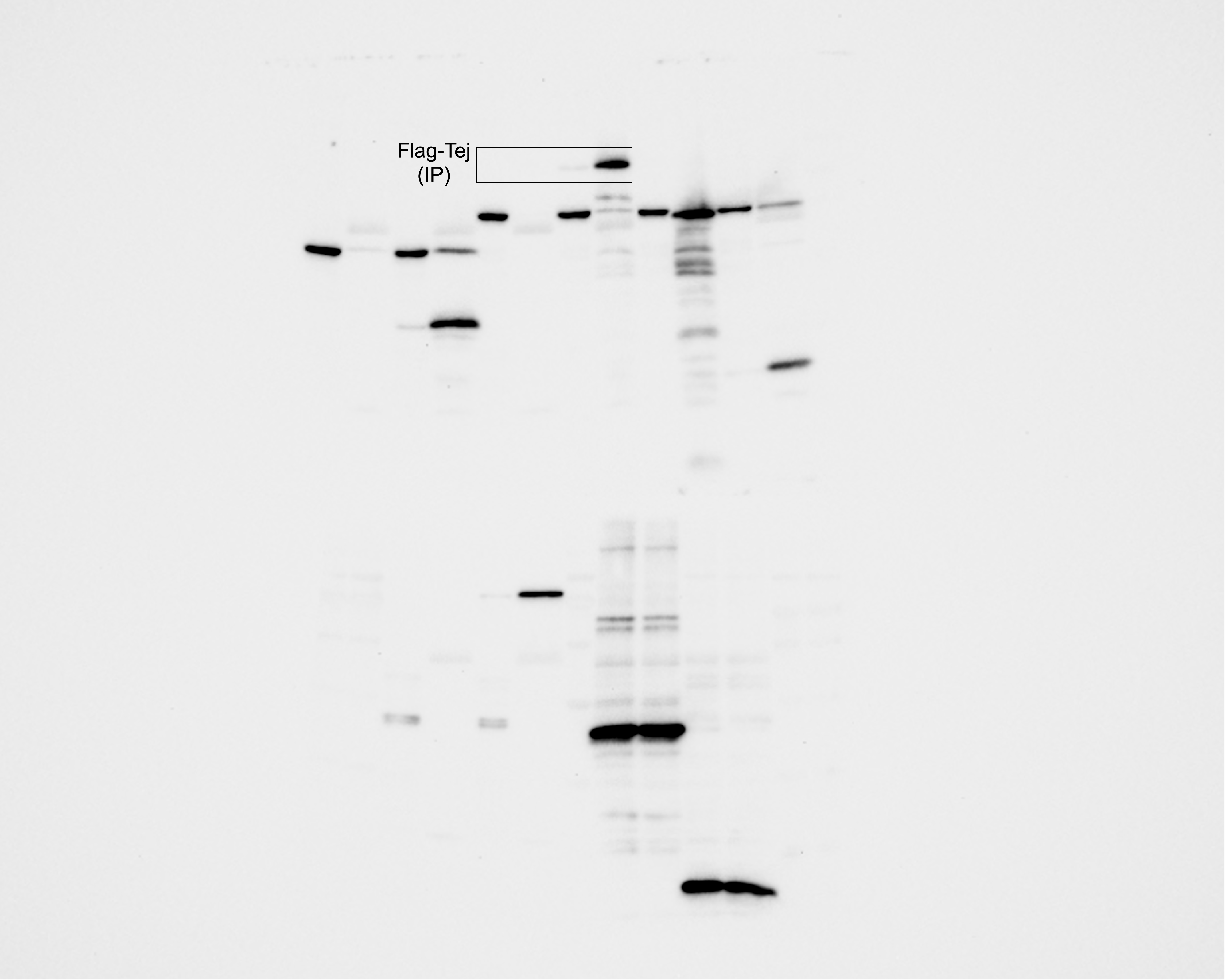

Supplement: Figure 4—source data 6. [file elife-101967-fig4-data6.zip › Figure 4-Source Data 6/Figure 4B-iii_rep2_FLAG_label_20230606.tiff]

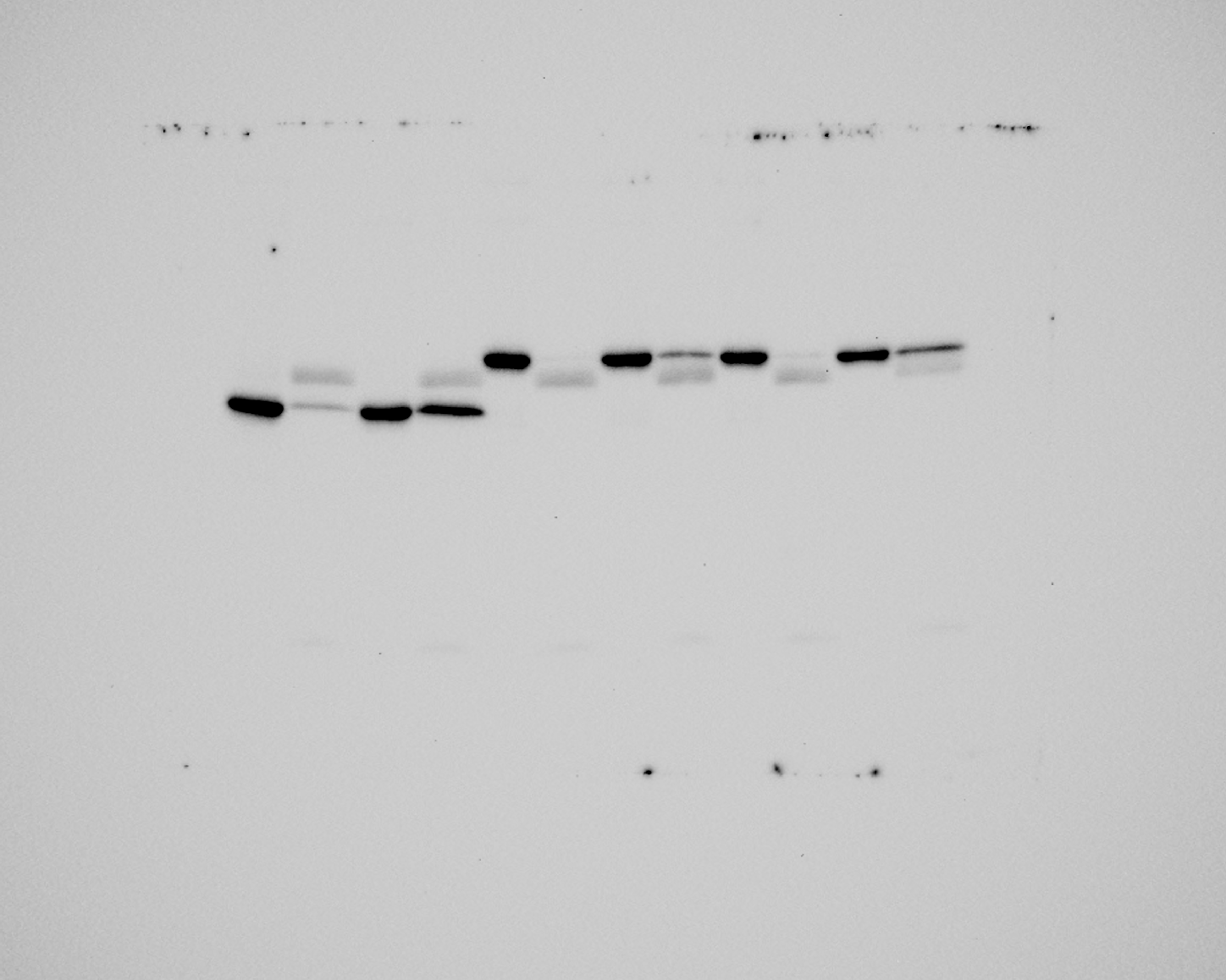

Supplement: Figure 4—source data 7. [file elife-101967-fig4-data7.zip › Figure 4-Source Data 7/Figure 4B-iii_rep2_Myc_original_20230606.tif]

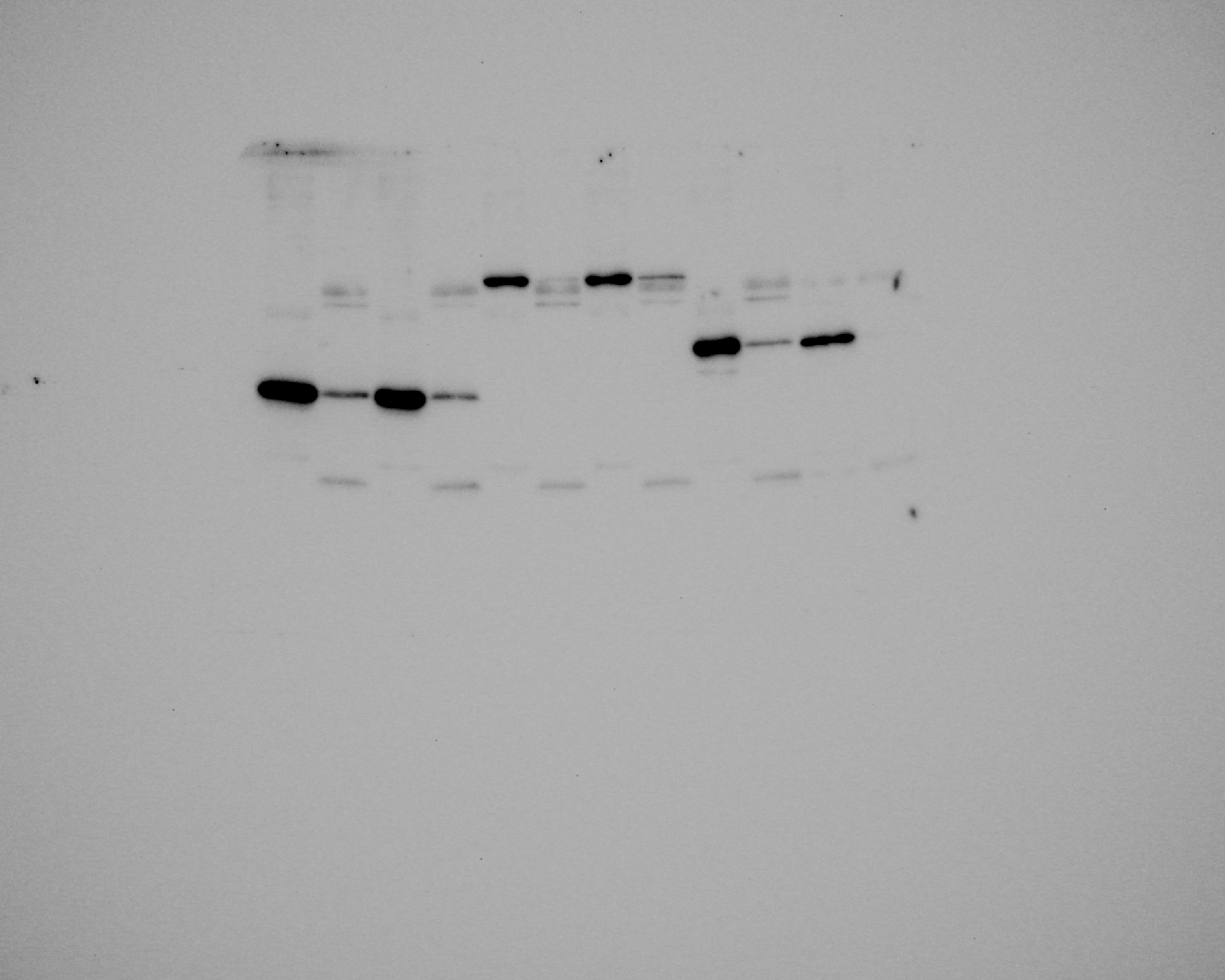

Supplement: Figure 4—source data 7. [file elife-101967-fig4-data7.zip › Figure 4-Source Data 7/Figure 4B-iii_rep1_Myc_original_20230519.tif]

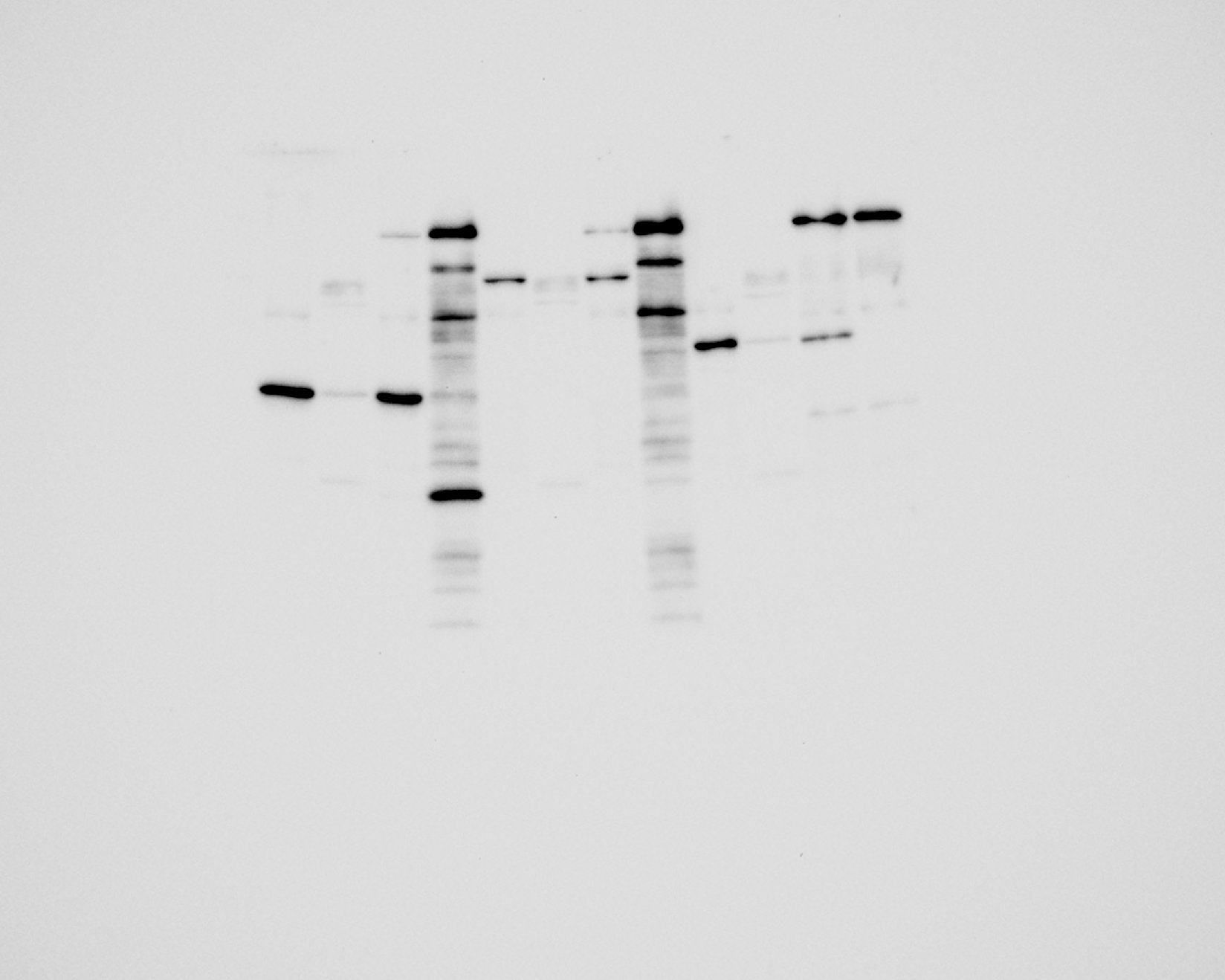

Supplement: Figure 4—source data 7. [file elife-101967-fig4-data7.zip › Figure 4-Source Data 7/Figure 4B-iii_rep1_FLAG_original_20230519.tif]

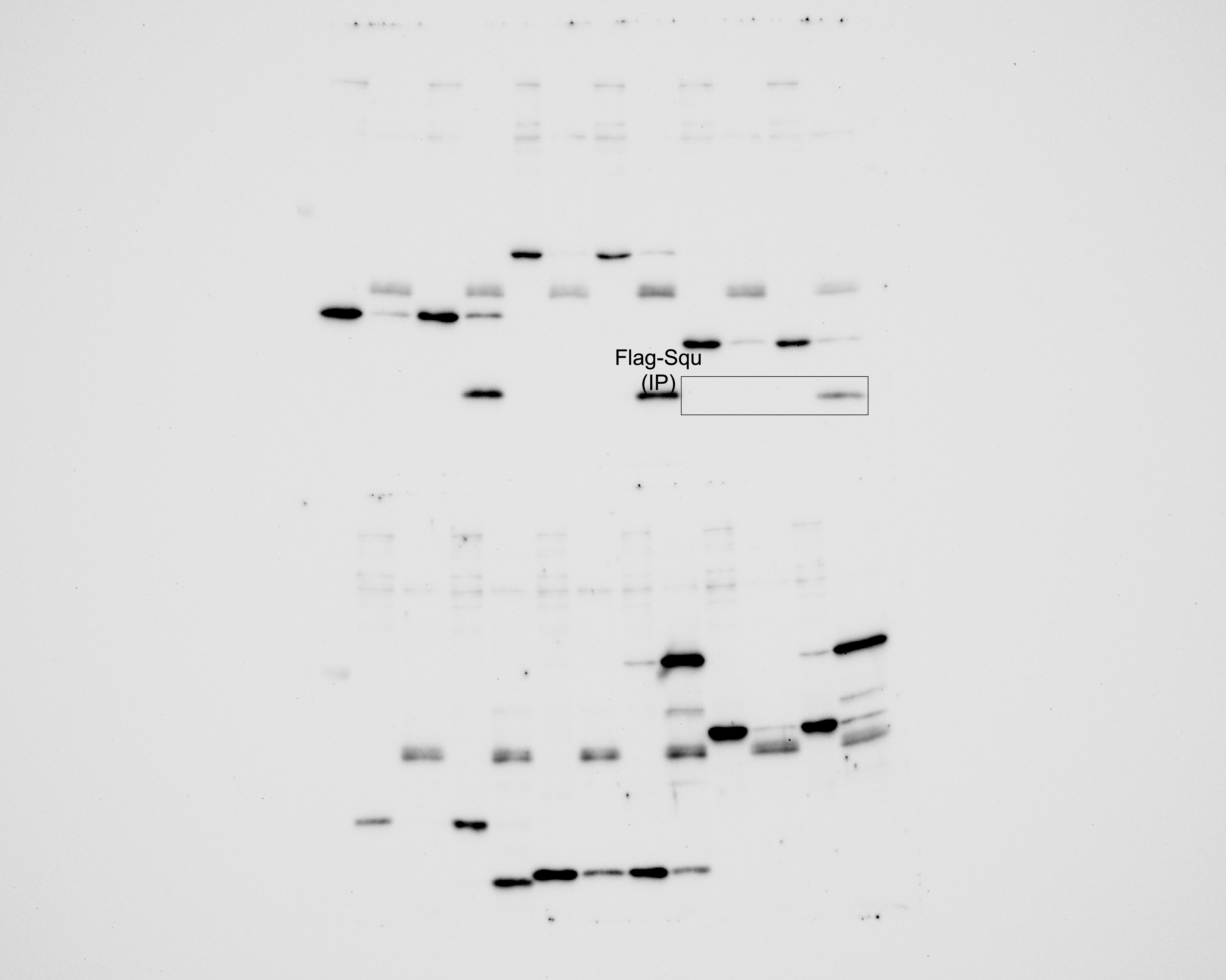

Supplement: Figure 4—figure supplement 1—source data 1. [file elife-101967-fig4-figsupp1-data1.zip › Figure 4-Figure Supplement 1-Source Data 1/Spn-D_Squ_FLAG_label_20230714.tiff]

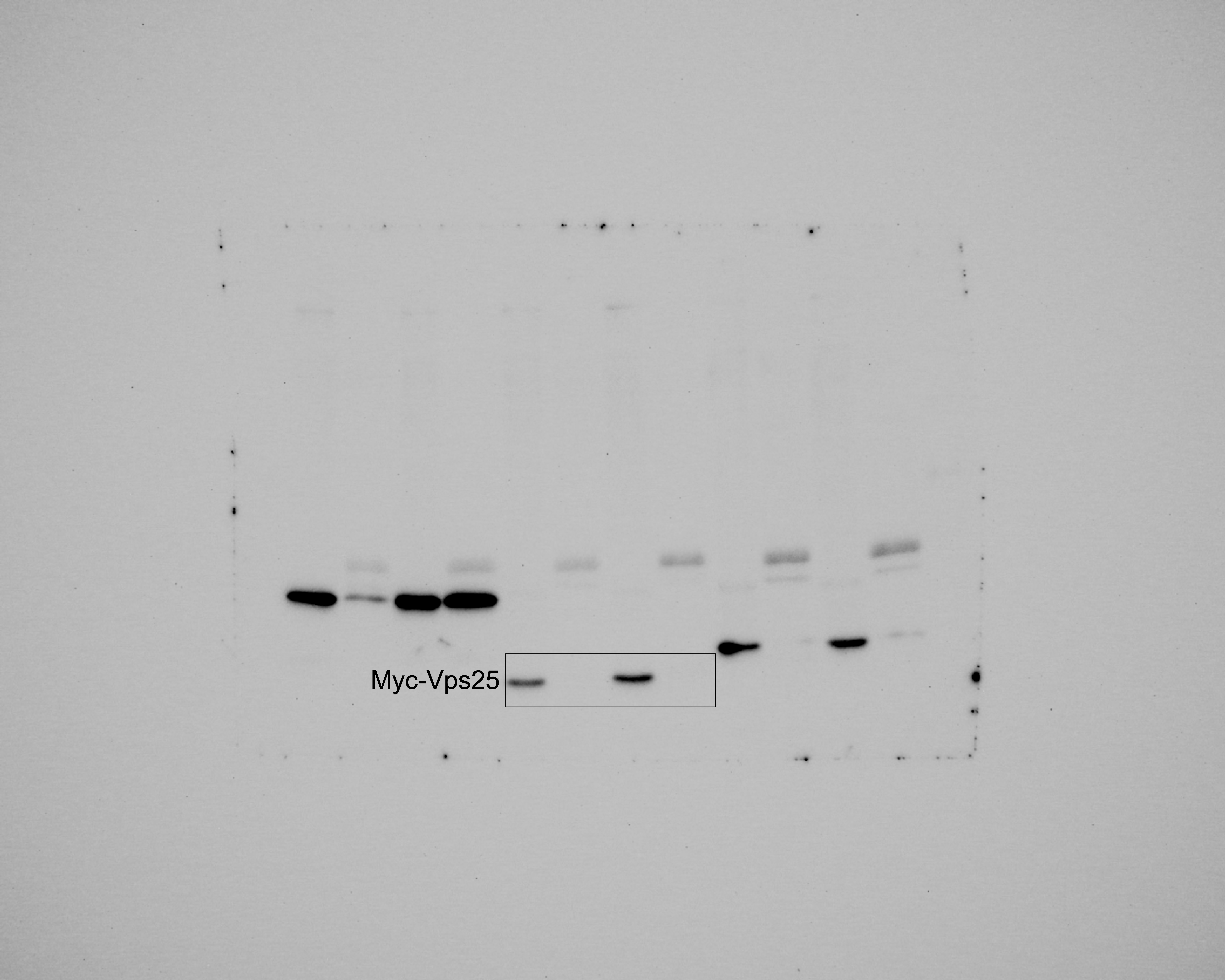

Supplement: Figure 4—figure supplement 1—source data 1. [file elife-101967-fig4-figsupp1-data1.zip › Figure 4-Figure Supplement 1-Source Data 1/Vps25_Squ_Myc_label_2023-05-24.tiff]

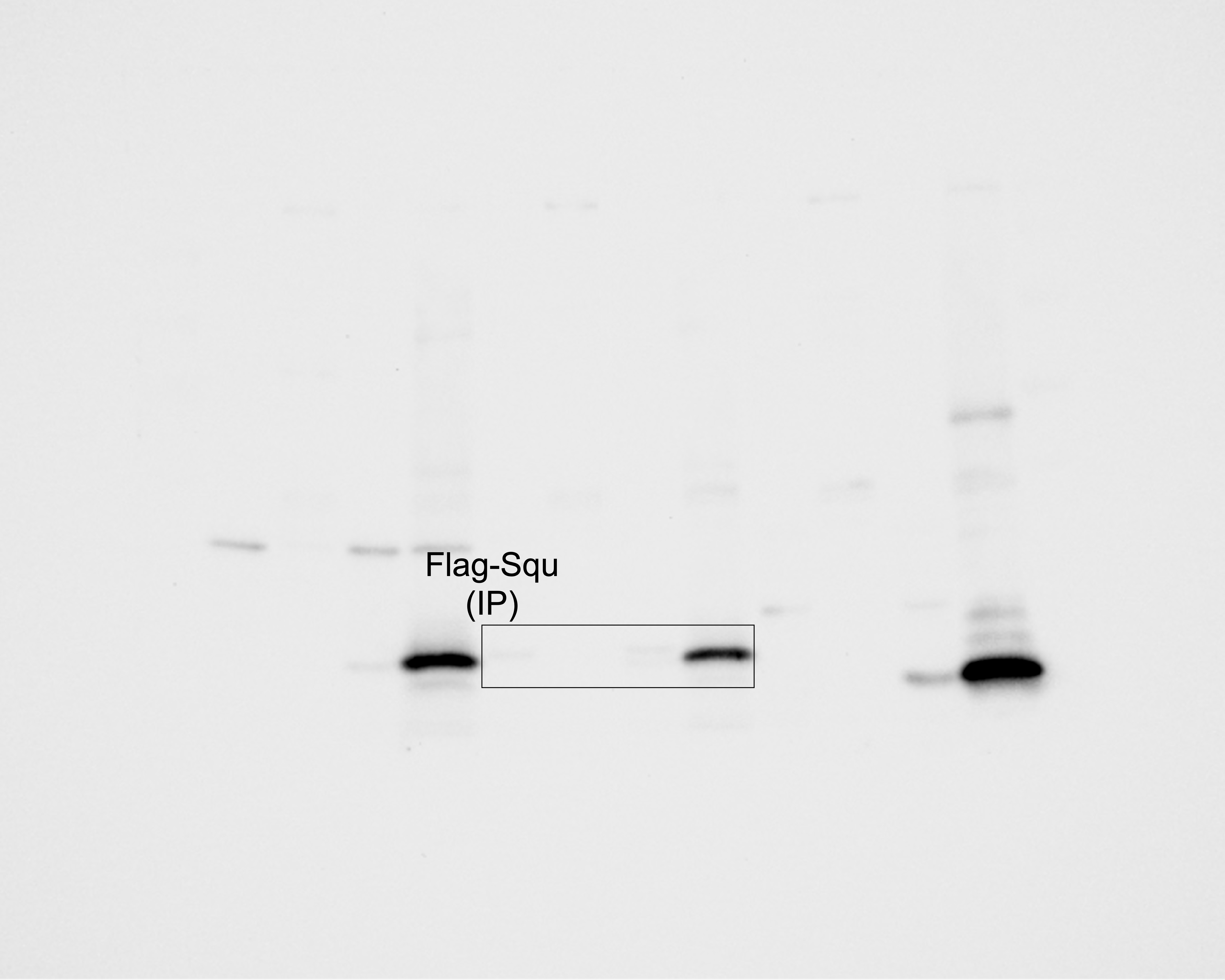

Supplement: Figure 4—figure supplement 1—source data 1. [file elife-101967-fig4-figsupp1-data1.zip › Figure 4-Figure Supplement 1-Source Data 1/Vps25_Squ_Flag_label_2023-05-24.tiff]

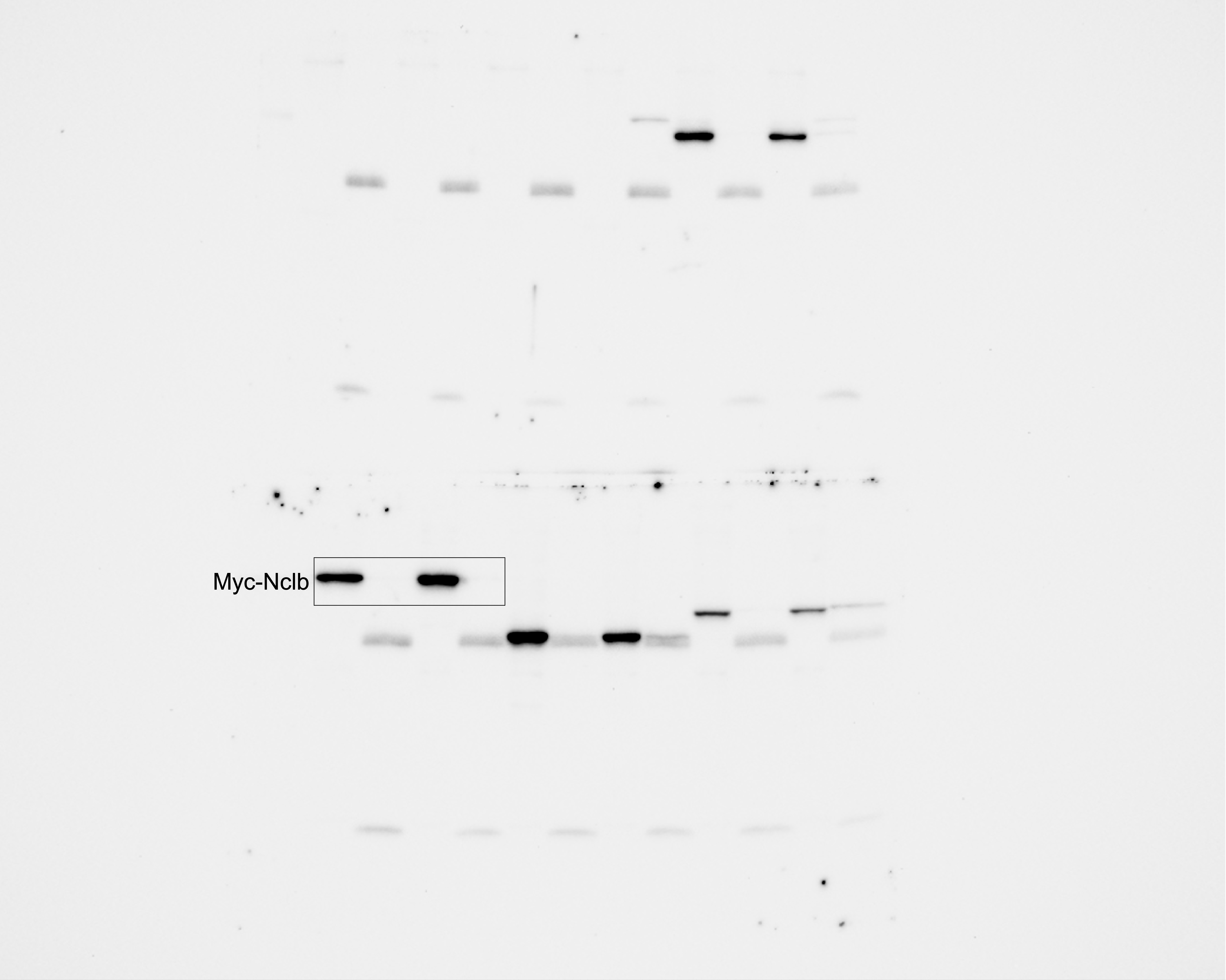

Supplement: Figure 4—figure supplement 1—source data 1. [file elife-101967-fig4-figsupp1-data1.zip › Figure 4-Figure Supplement 1-Source Data 1/Nclb_Squ_Myc_label_20230613.tiff]

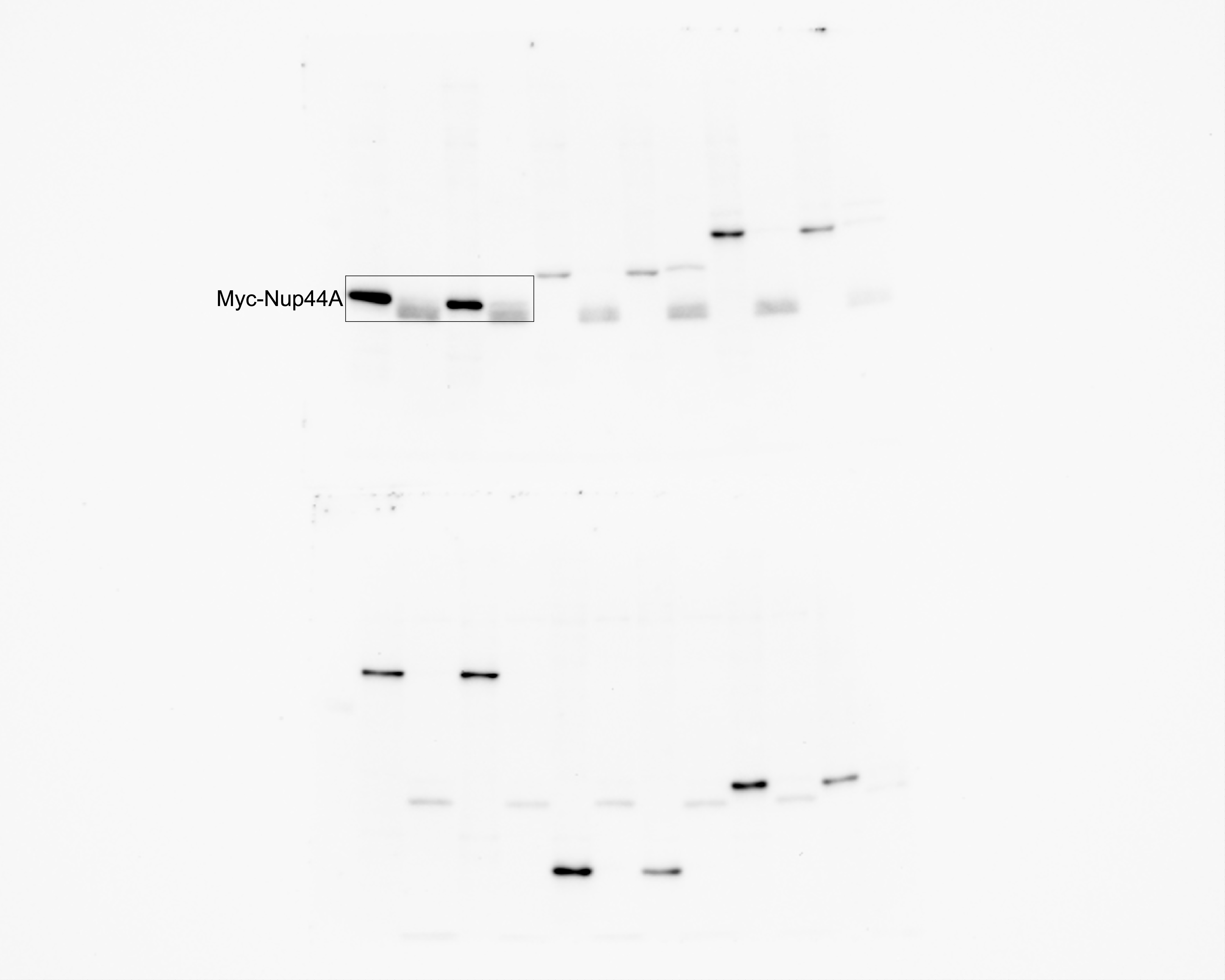

Supplement: Figure 4—figure supplement 1—source data 1. [file elife-101967-fig4-figsupp1-data1.zip › Figure 4-Figure Supplement 1-Source Data 1/Nup44A_Squ_Myc_label_20230623.tiff]

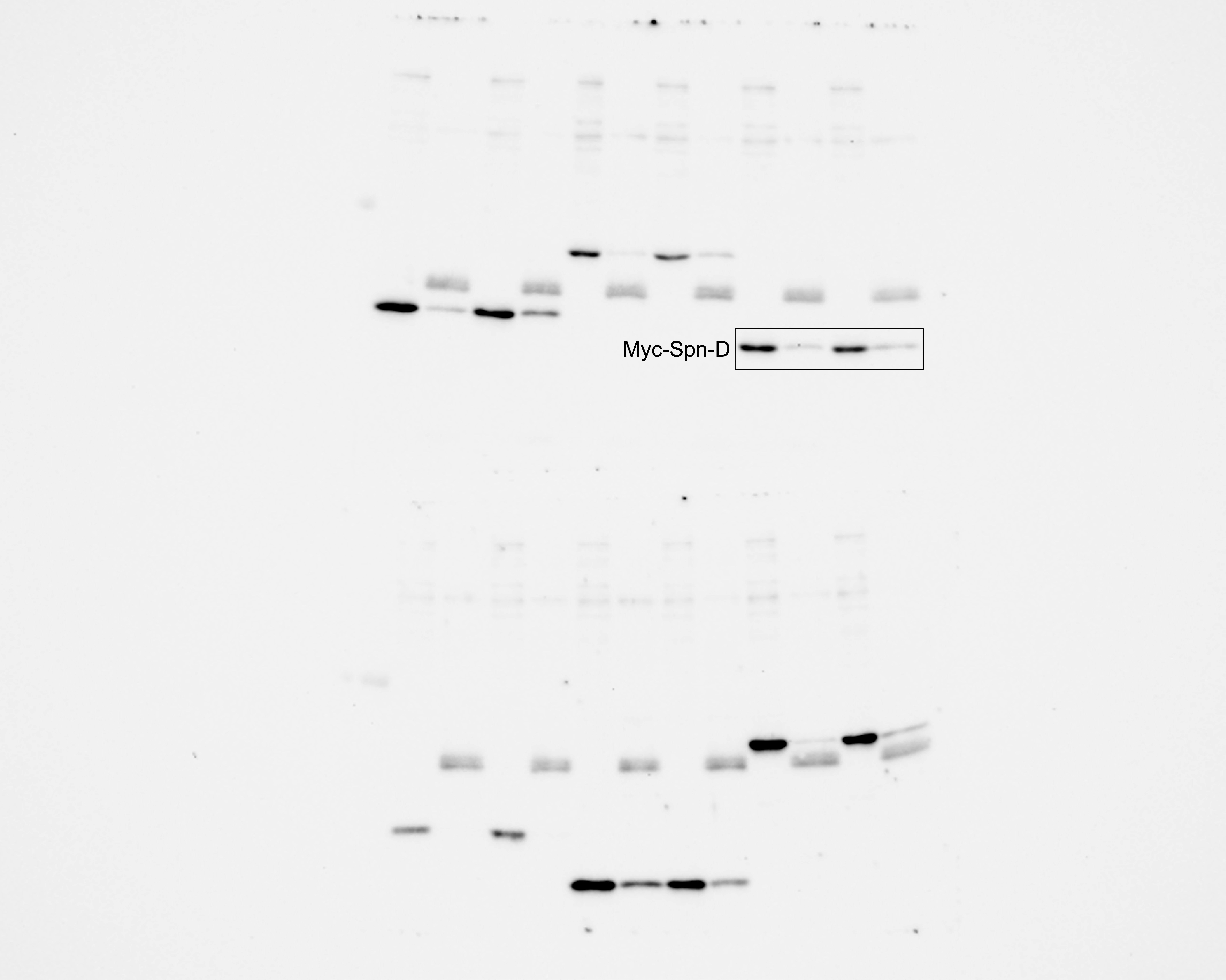

Supplement: Figure 4—figure supplement 1—source data 1. [file elife-101967-fig4-figsupp1-data1.zip › Figure 4-Figure Supplement 1-Source Data 1/Spn-D_Squ_Myc_label_20230714.tiff]

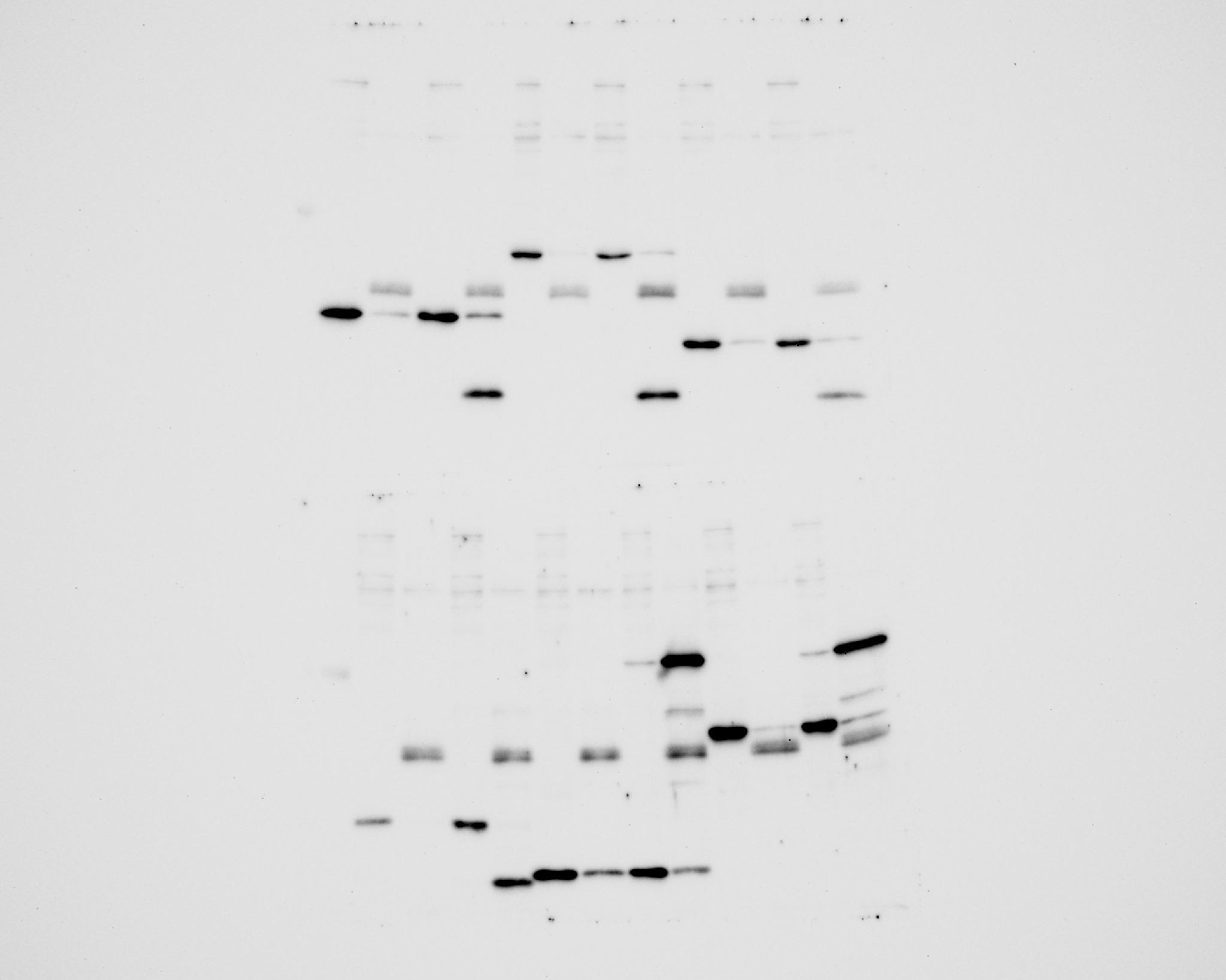

Supplement: Figure 4—figure supplement 1—source data 2. [file elife-101967-fig4-figsupp1-data2.zip › Figure 4-Figure Supplement 1-Source Data 2/Spn-D_Squ_FLAG_original_20230714.tif]

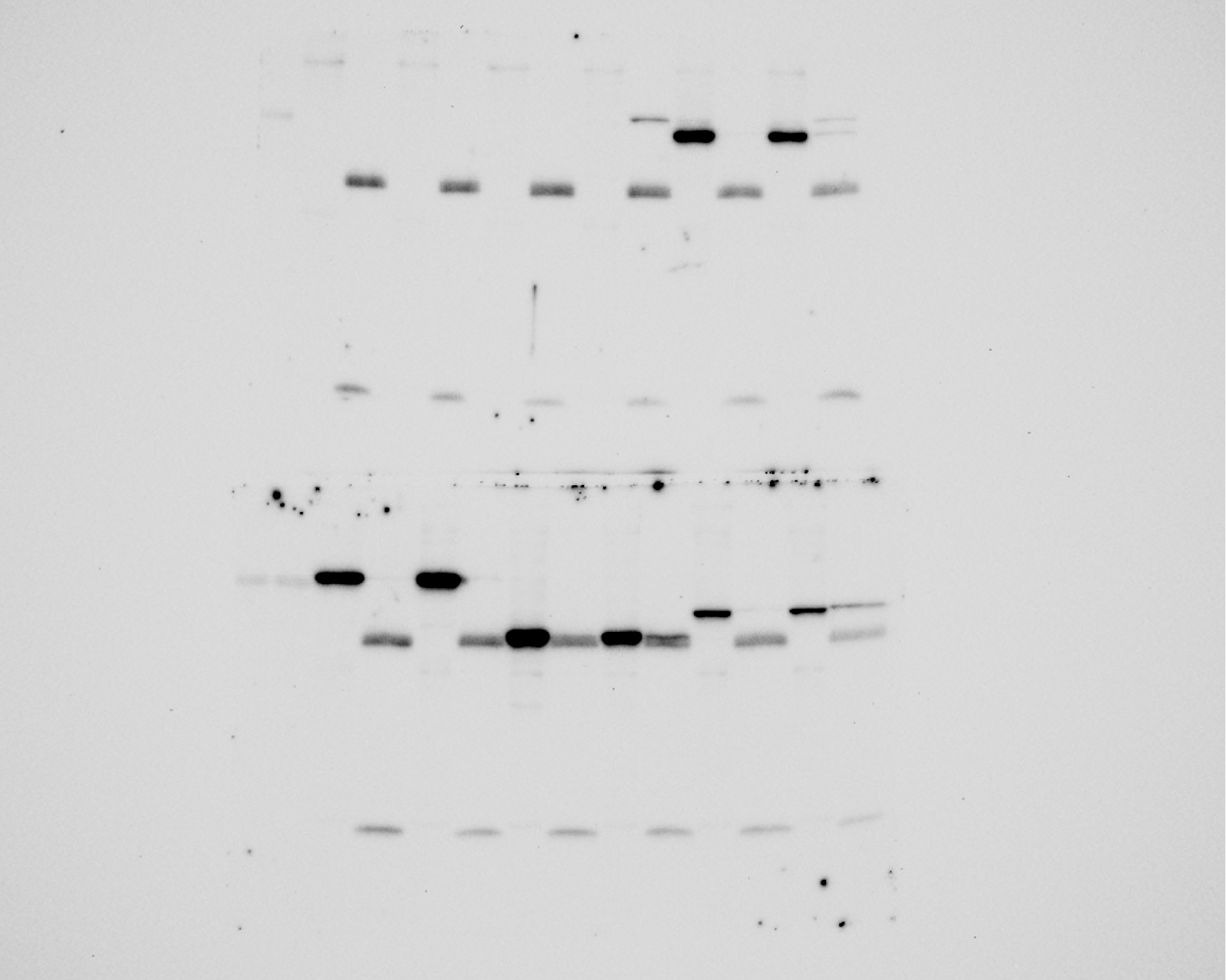

Supplement: Figure 4—figure supplement 1—source data 2. [file elife-101967-fig4-figsupp1-data2.zip › Figure 4-Figure Supplement 1-Source Data 2/Nclb_Squ_Myc_original_20230613.tif]

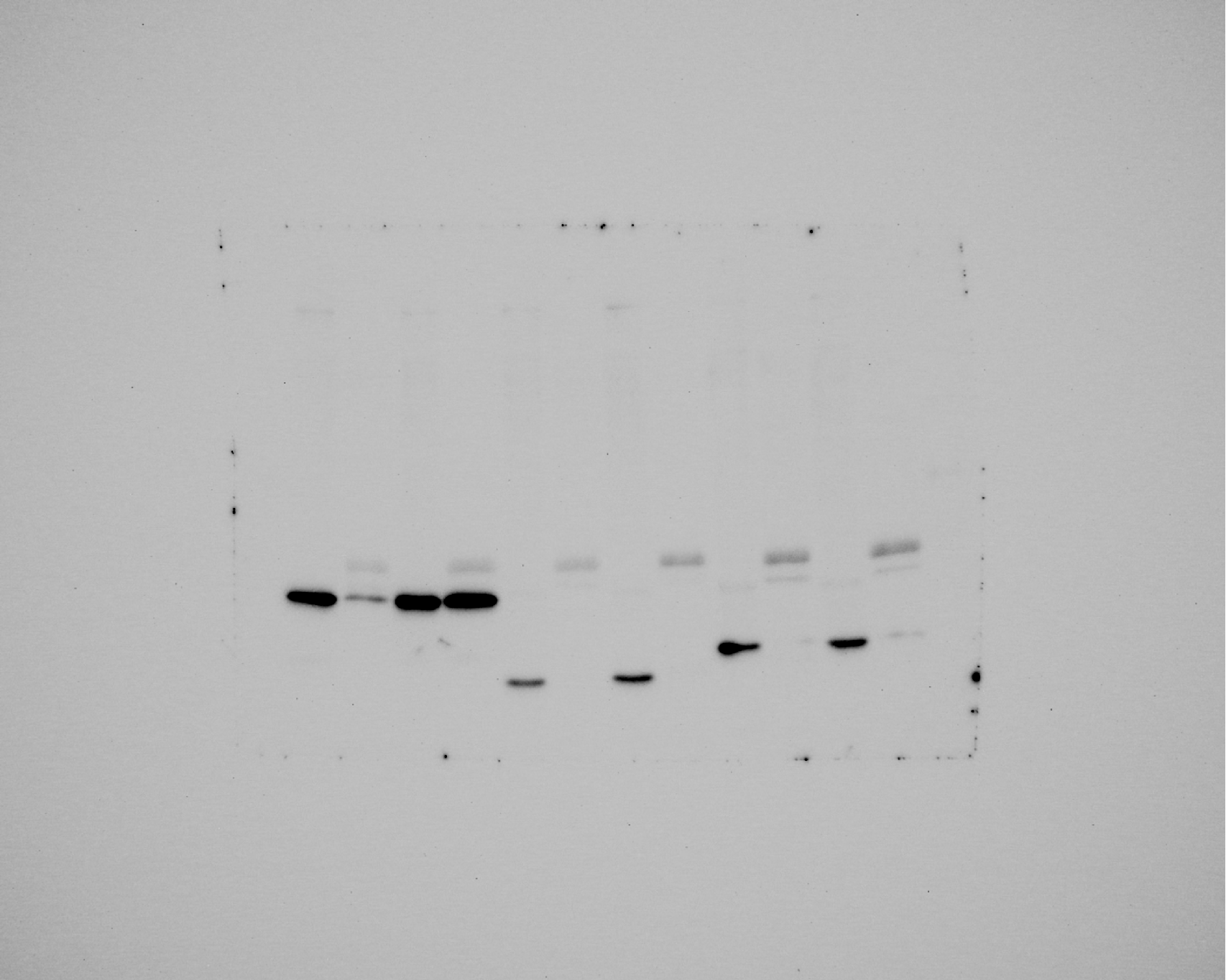

Supplement: Figure 4—figure supplement 1—source data 2. [file elife-101967-fig4-figsupp1-data2.zip › Figure 4-Figure Supplement 1-Source Data 2/Vps25_Squ_Myc_original_2023-05-24.tif]

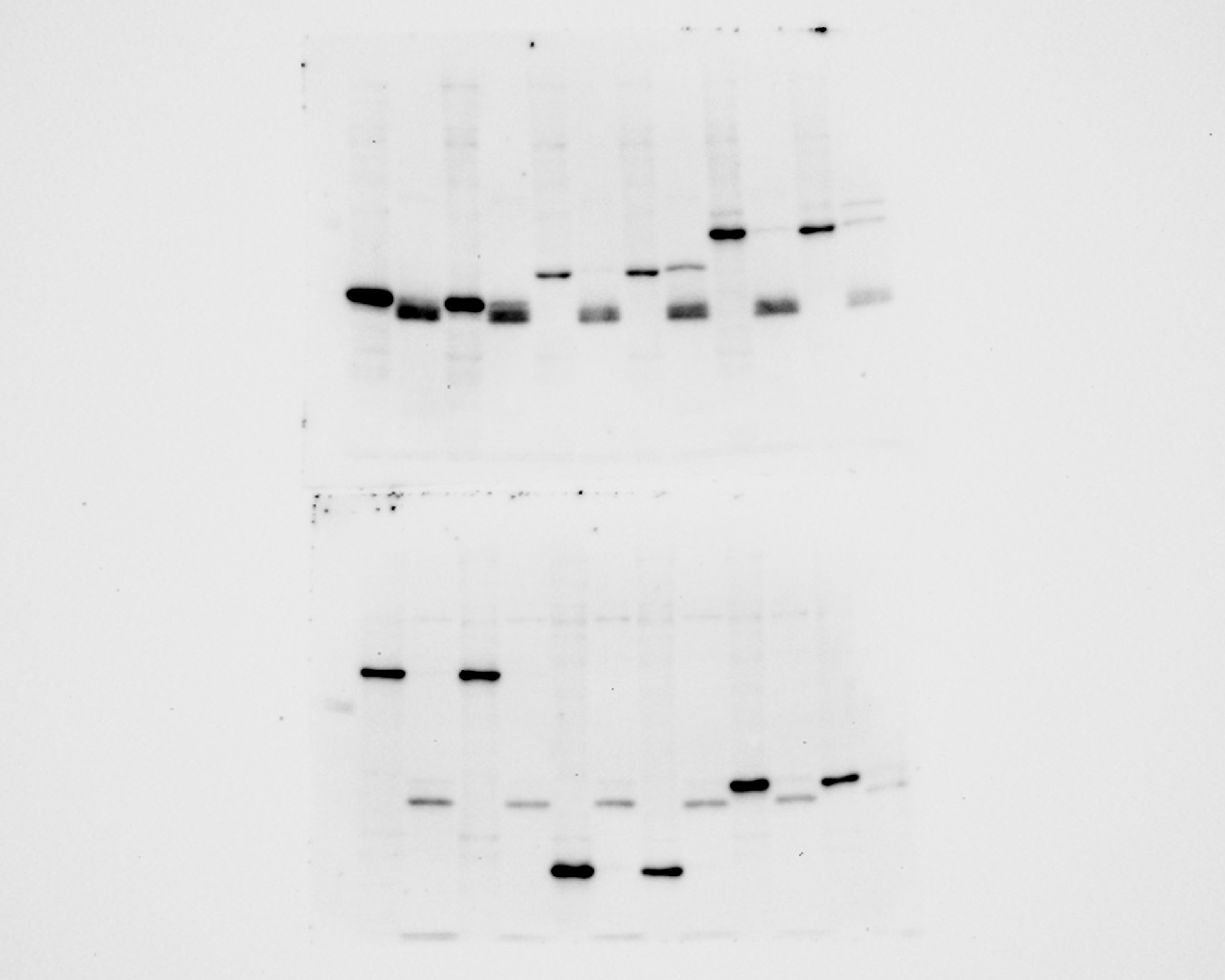

Supplement: Figure 4—figure supplement 1—source data 2. [file elife-101967-fig4-figsupp1-data2.zip › Figure 4-Figure Supplement 1-Source Data 2/Nup44A_Squ_Myc_original_20230623.tif]

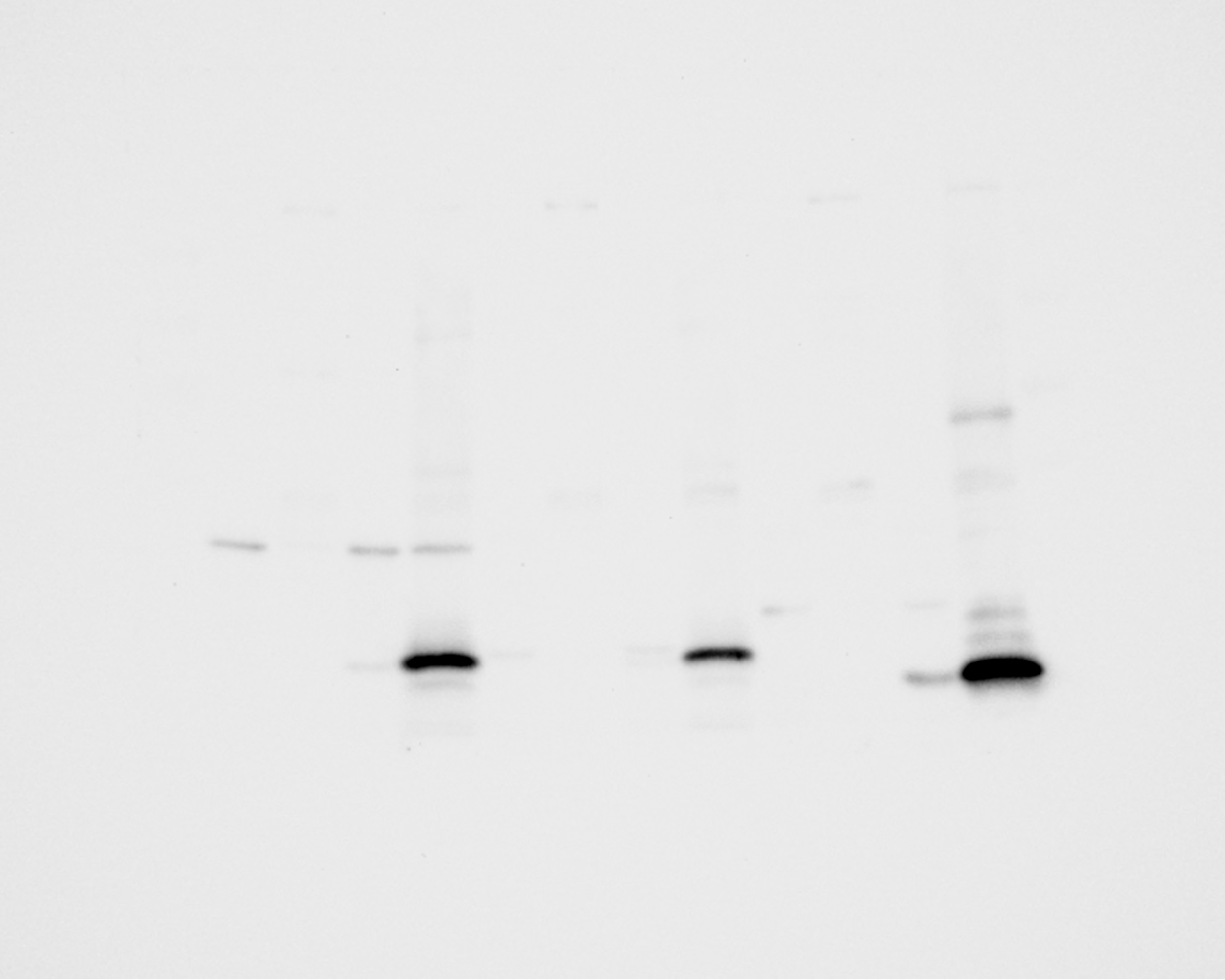

Supplement: Figure 4—figure supplement 1—source data 2. [file elife-101967-fig4-figsupp1-data2.zip › Figure 4-Figure Supplement 1-Source Data 2/Vps25_Squ_Flag_original_2023-05-24.tif]

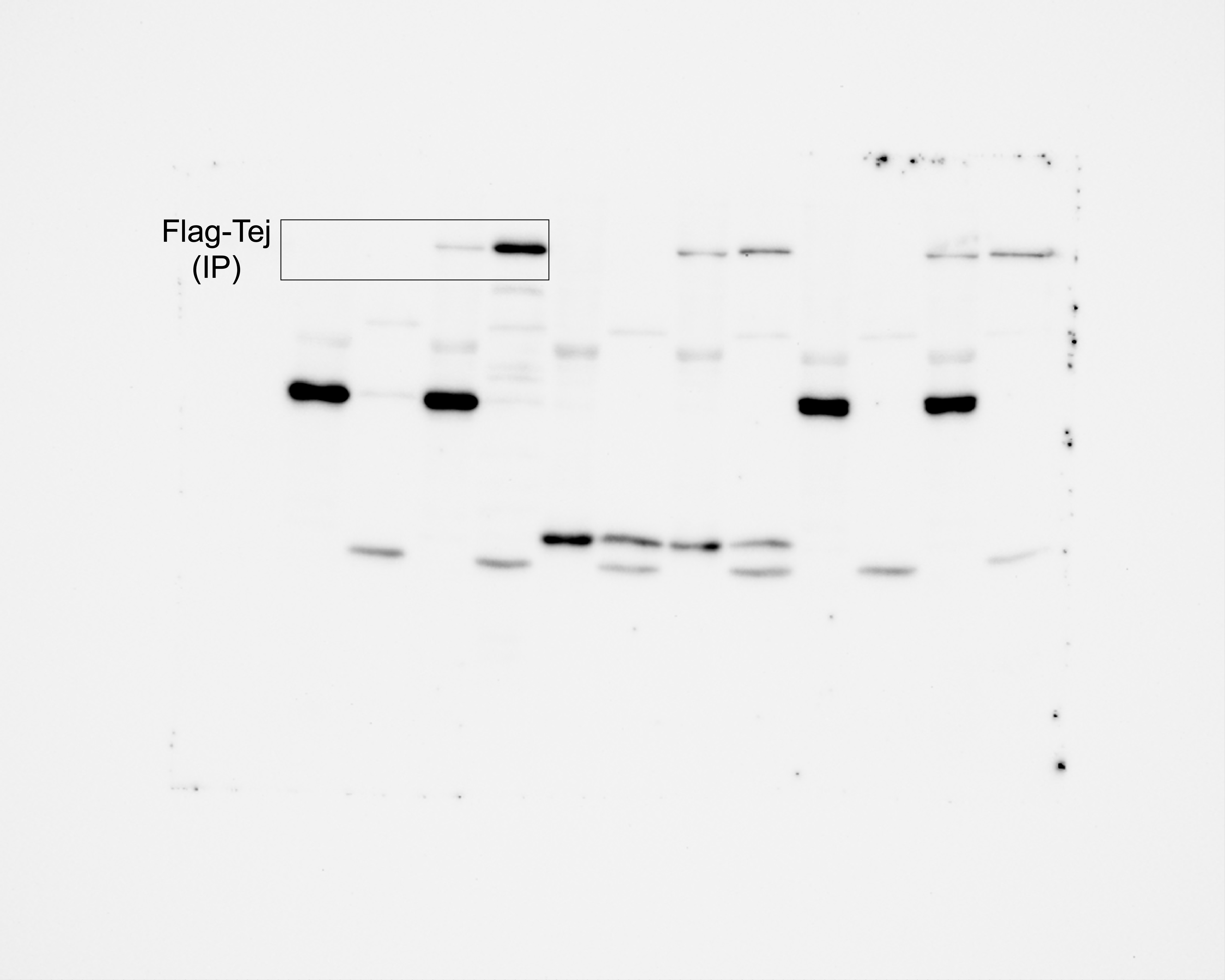

Supplement: Figure 4—figure supplement 1—source data 3. [file elife-101967-fig4-figsupp1-data3.zip › Figure 4-Figure Supplement 1-Source Data 3/Rab7_Tej_Flag_label_2023-06-16.tiff]

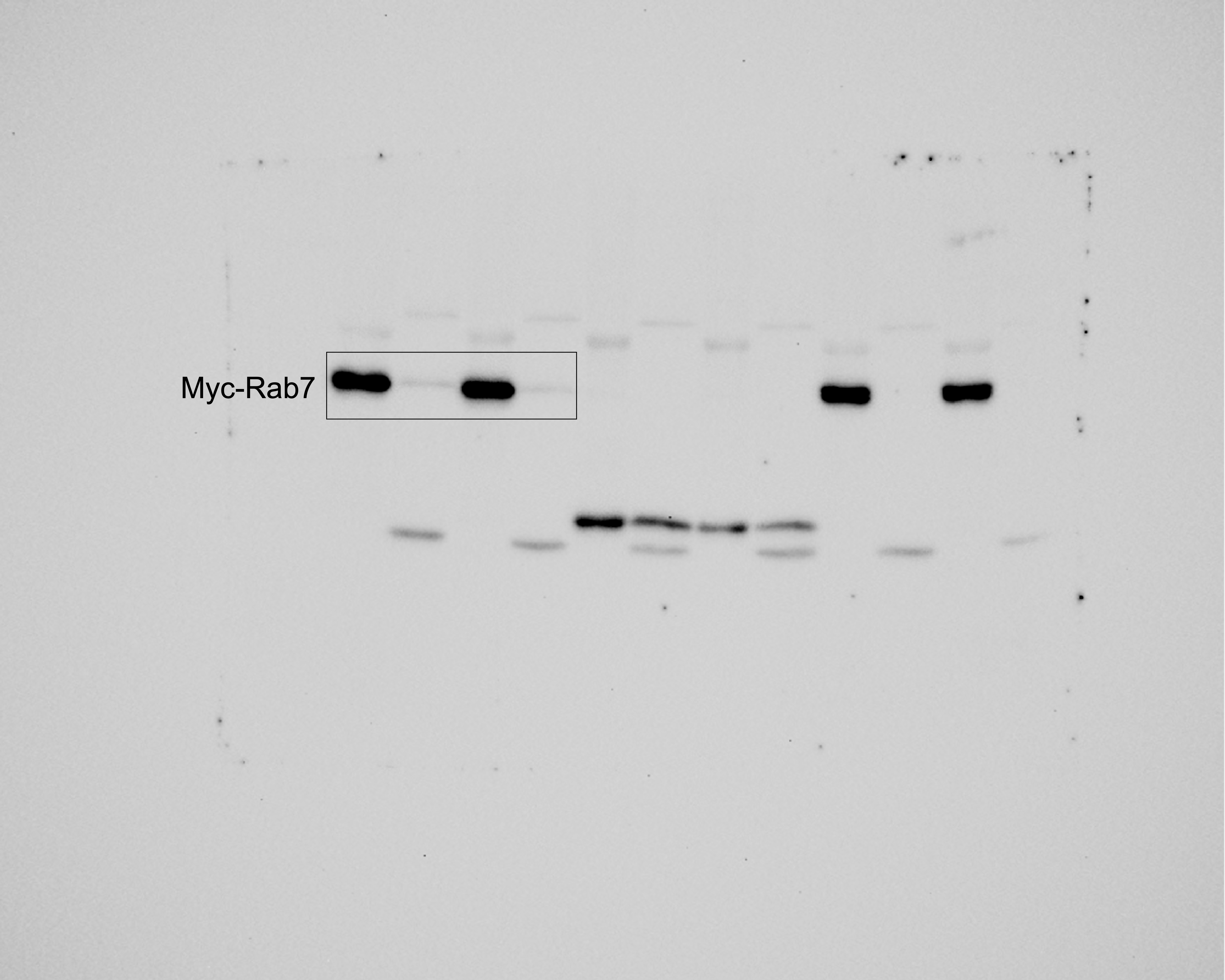

Supplement: Figure 4—figure supplement 1—source data 3. [file elife-101967-fig4-figsupp1-data3.zip › Figure 4-Figure Supplement 1-Source Data 3/Rab7_Tej_Myc_label_2023-06-16.tiff]

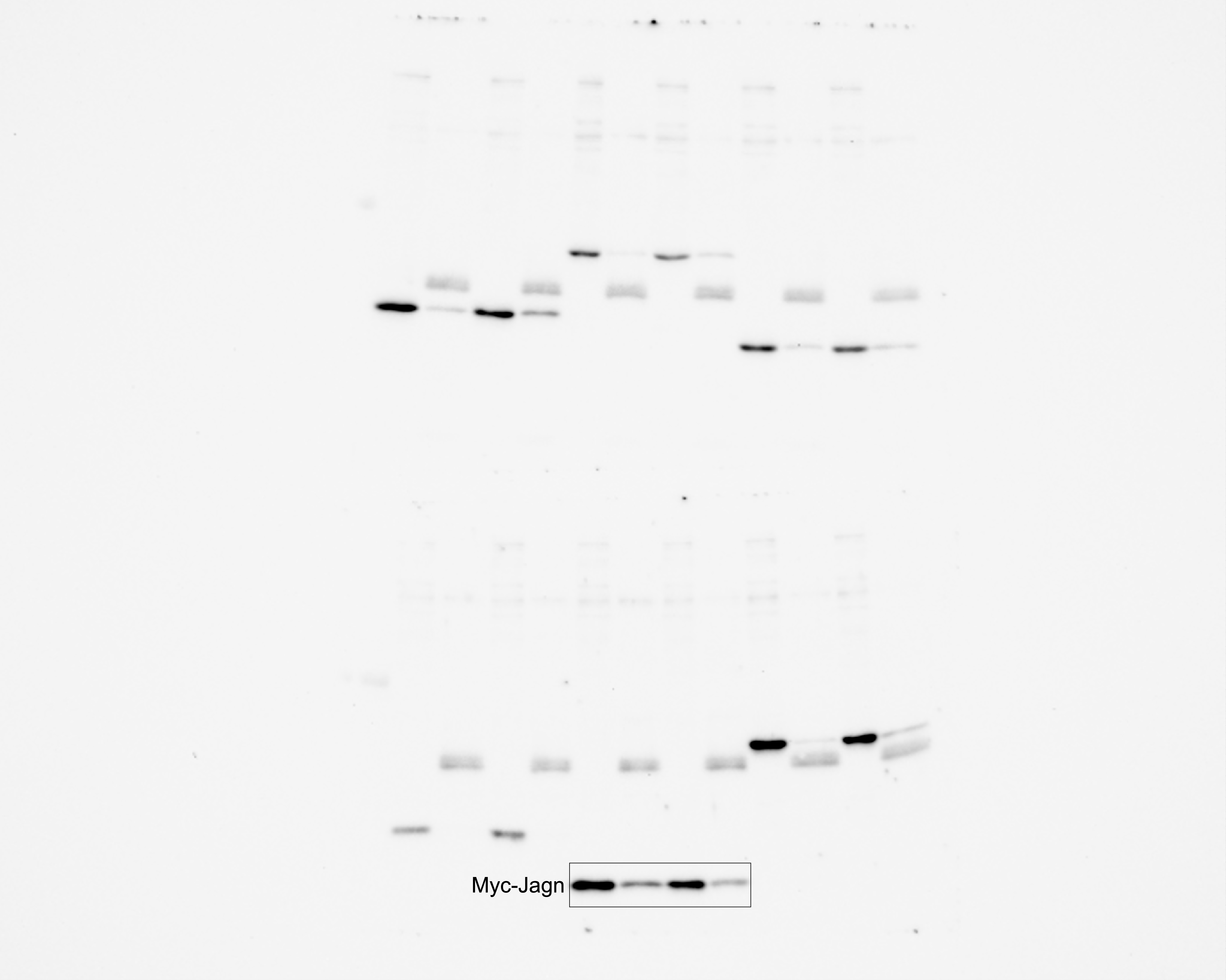

Supplement: Figure 4—figure supplement 1—source data 3. [file elife-101967-fig4-figsupp1-data3.zip › Figure 4-Figure Supplement 1-Source Data 3/Jagn_Tej_Myc_label_20230714.tiff]

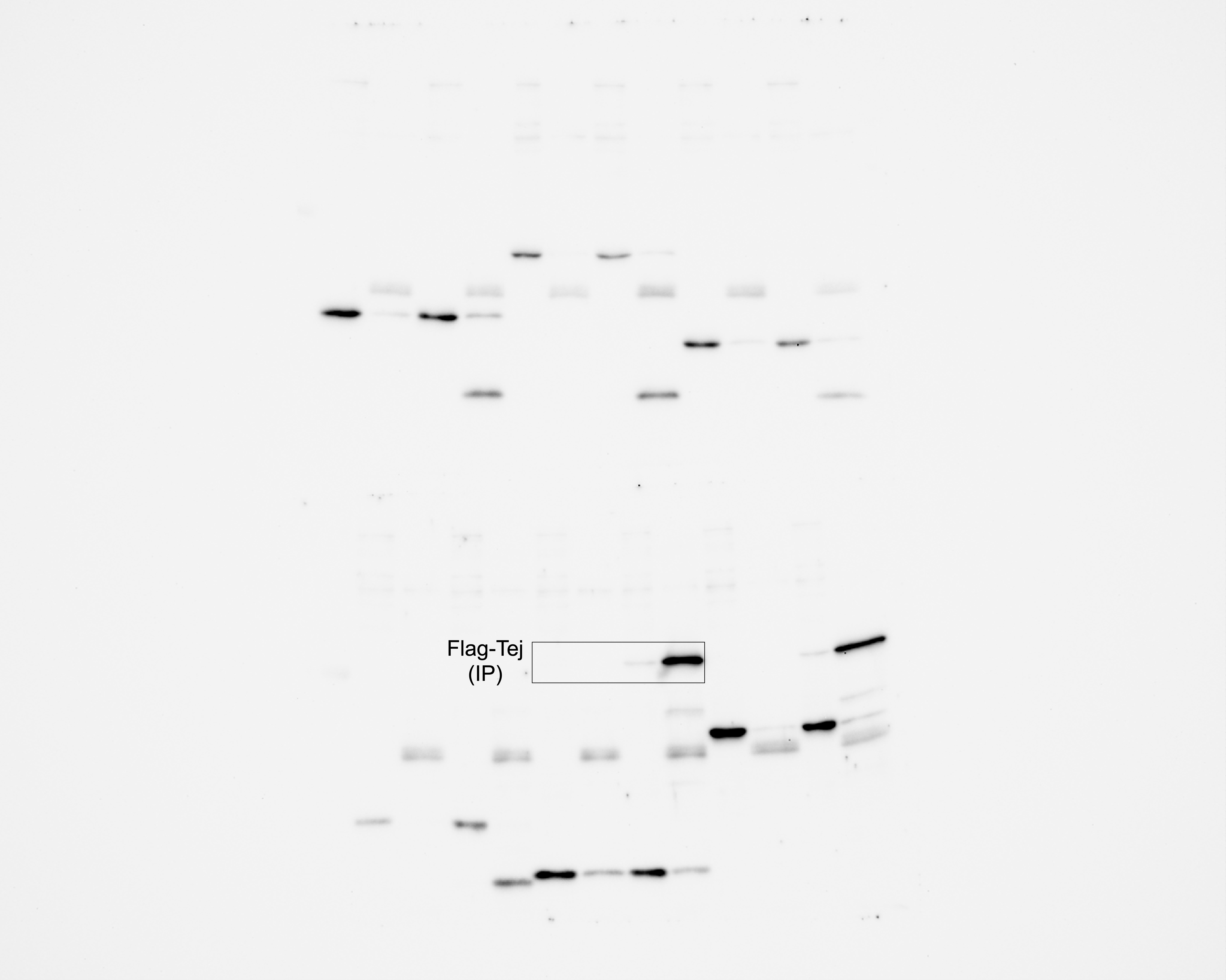

Supplement: Figure 4—figure supplement 1—source data 3. [file elife-101967-fig4-figsupp1-data3.zip › Figure 4-Figure Supplement 1-Source Data 3/Jagn_Tej_FLAG_label_20230714.tiff]

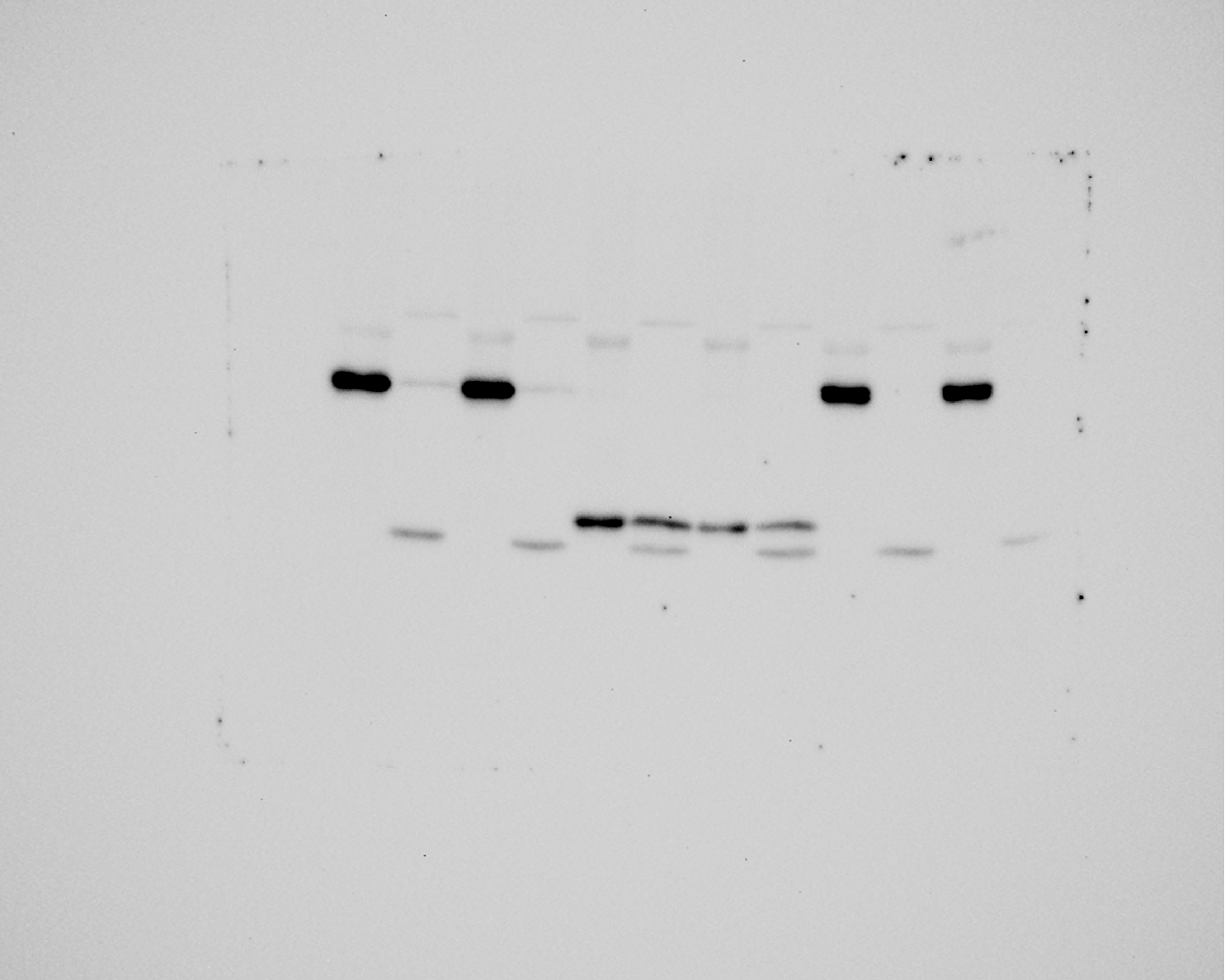

Supplement: Figure 4—figure supplement 1—source data 4. [file elife-101967-fig4-figsupp1-data4.zip › Figure 4-Figure Supplement 1-Source Data 4/Rab7_Tej_Myc_original_2023-06-16.tif]

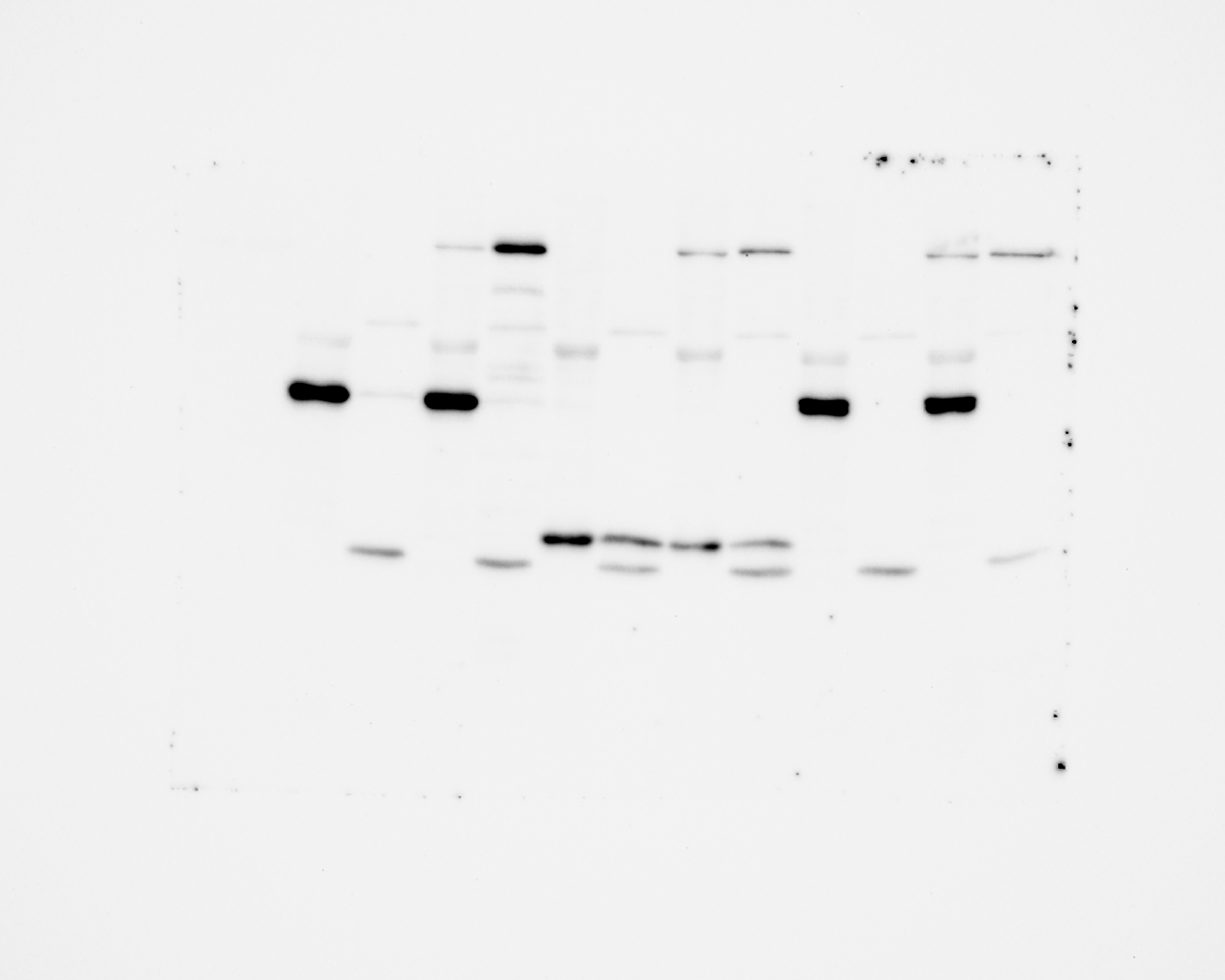

Supplement: Figure 4—figure supplement 1—source data 4. [file elife-101967-fig4-figsupp1-data4.zip › Figure 4-Figure Supplement 1-Source Data 4/Rab7_Tej_Flag_original_2023-06-16.tif]

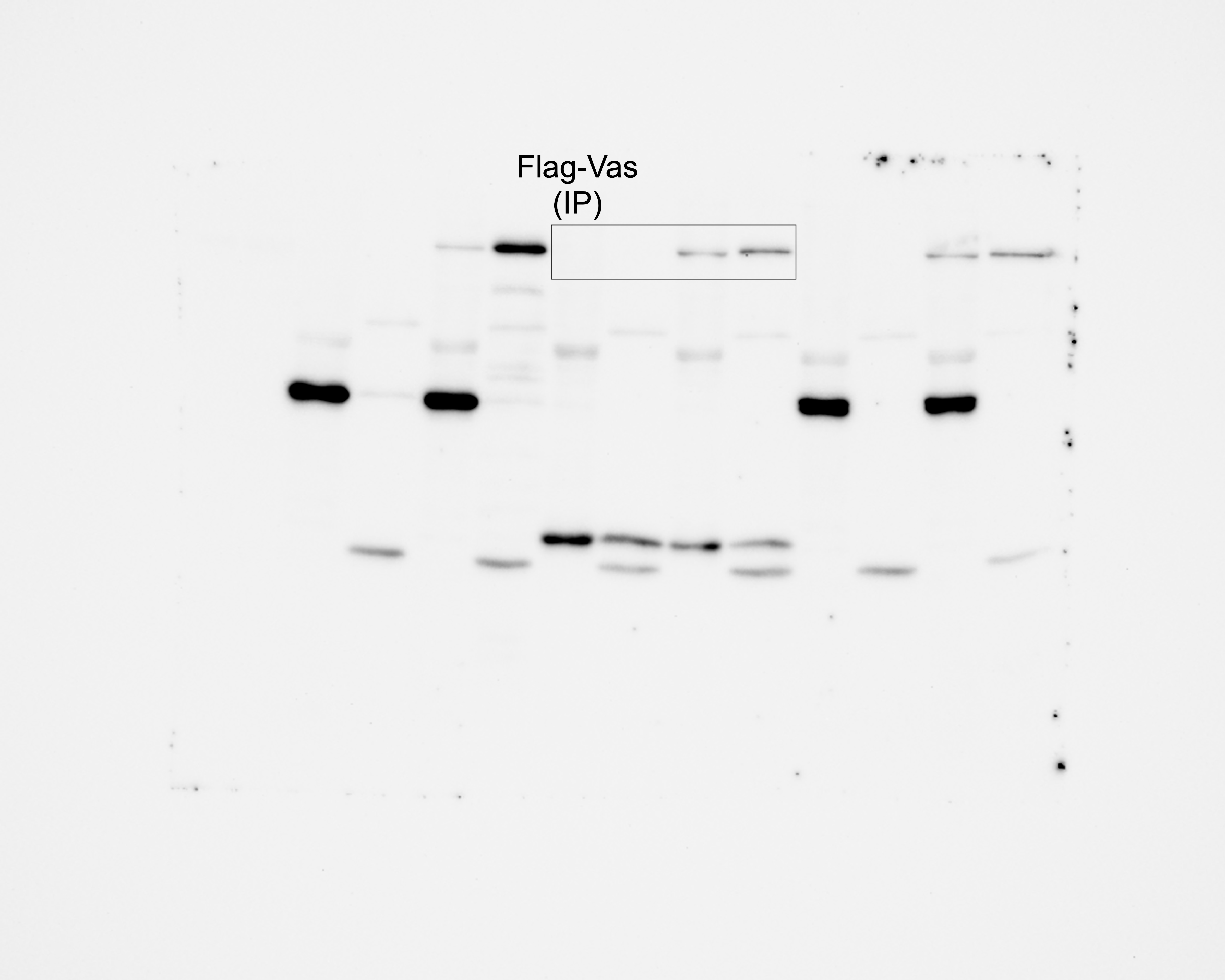

Supplement: Figure 4—figure supplement 1—source data 5. [file elife-101967-fig4-figsupp1-data5.zip › Figure 4-Figure Supplement 1-Source Data 5/Baf_Vas_Flag_label_2023-06-16.tiff]

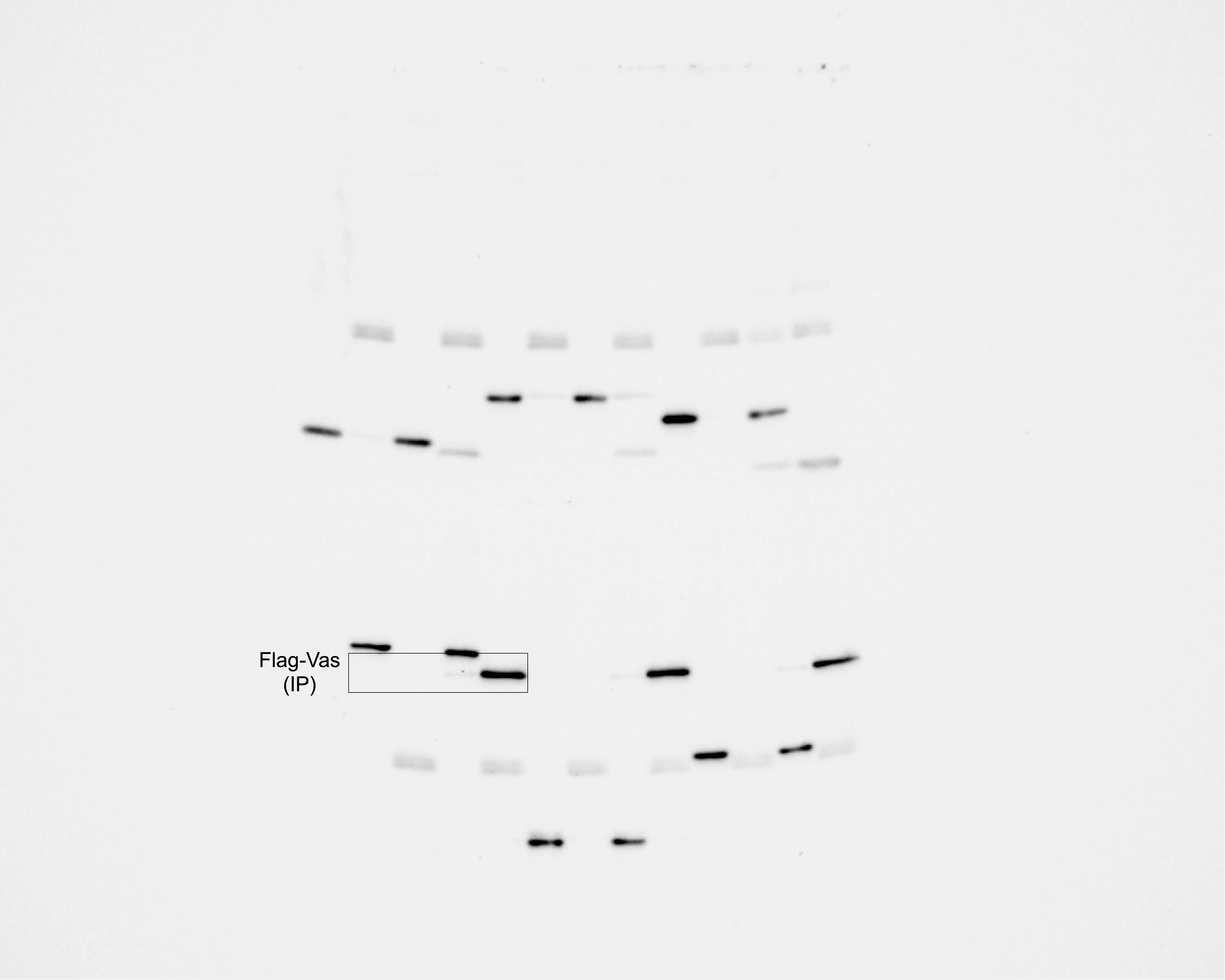

Supplement: Figure 4—figure supplement 1—source data 5. [file elife-101967-fig4-figsupp1-data5.zip › Figure 4-Figure Supplement 1-Source Data 5/Hsc70-4_Vas_Flag_label_2023-06-30.tiff]

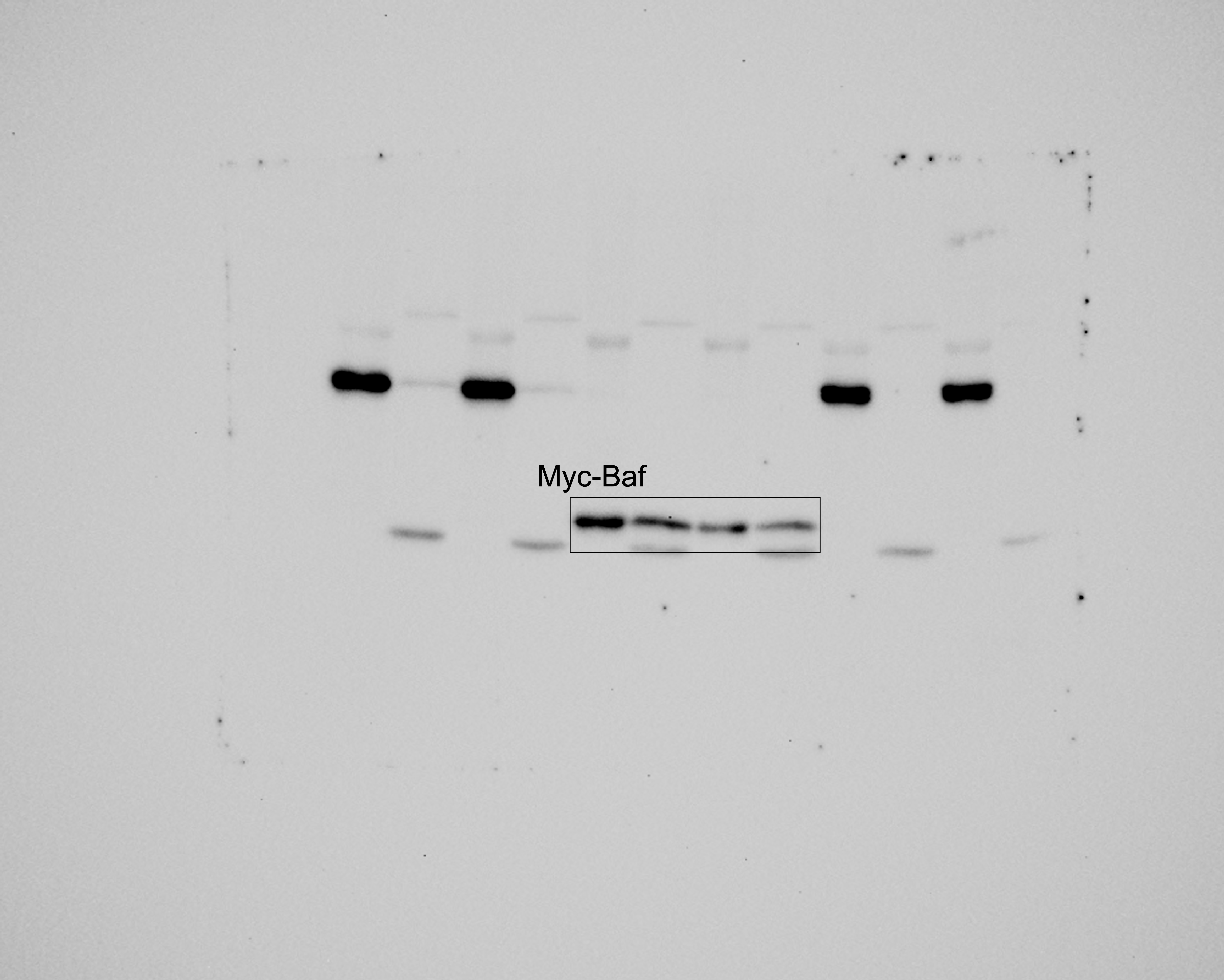

Supplement: Figure 4—figure supplement 1—source data 5. [file elife-101967-fig4-figsupp1-data5.zip › Figure 4-Figure Supplement 1-Source Data 5/Baf_Vas_Myc_label_2023-06-16.tiff]

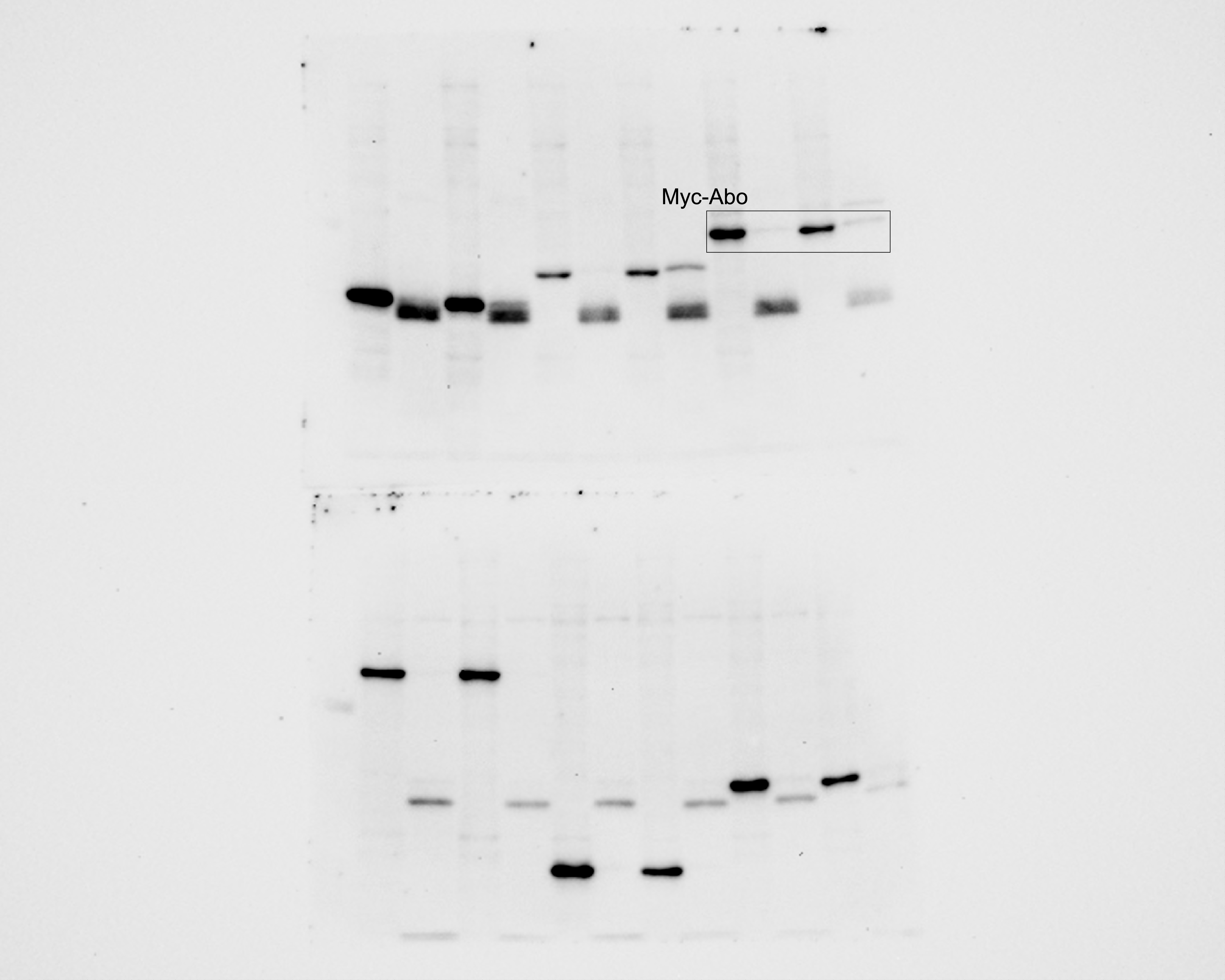

Supplement: Figure 4—figure supplement 1—source data 5. [file elife-101967-fig4-figsupp1-data5.zip › Figure 4-Figure Supplement 1-Source Data 5/Abo_Vas_Myc_label_20230623.tiff]

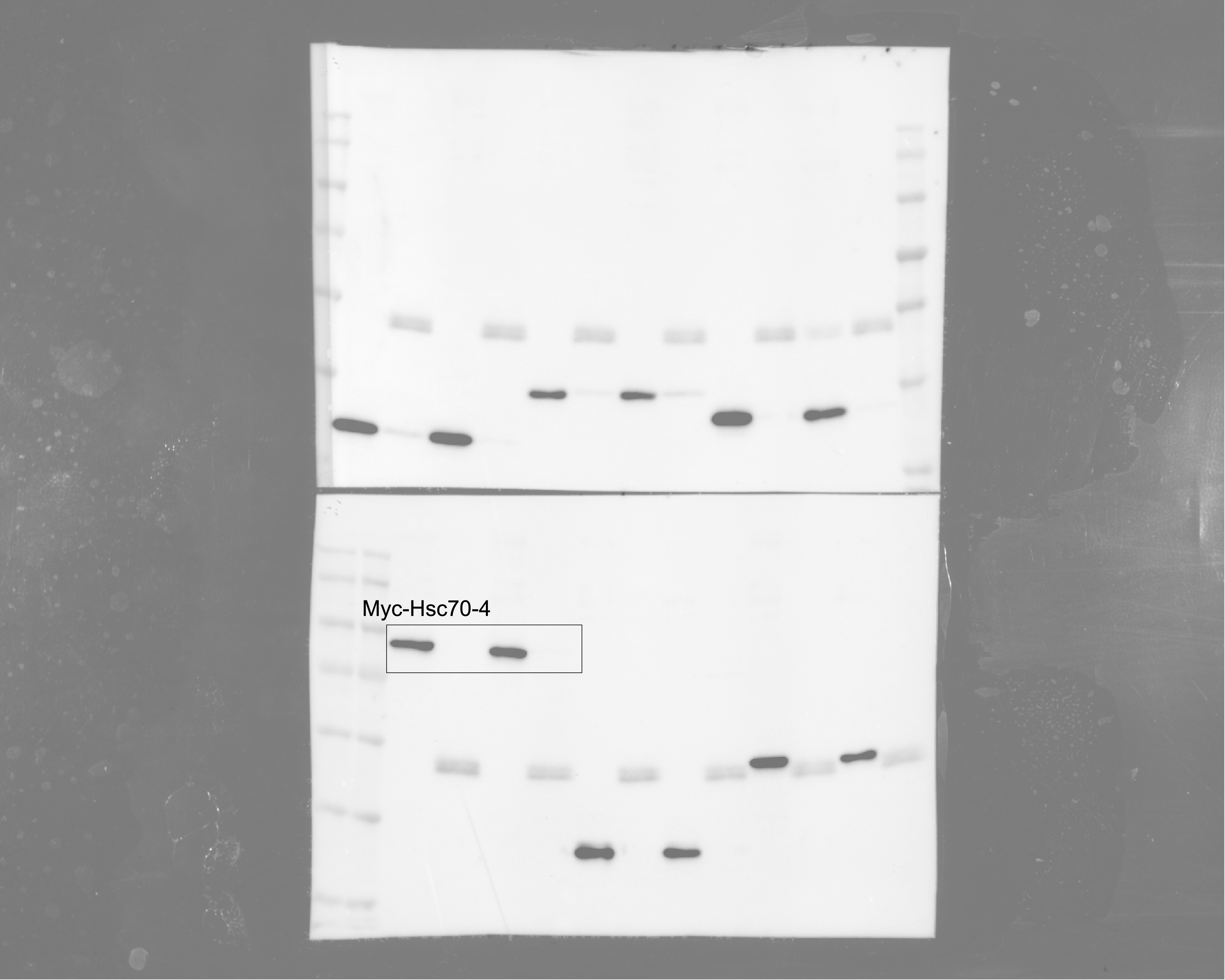

Supplement: Figure 4—figure supplement 1—source data 5. [file elife-101967-fig4-figsupp1-data5.zip › Figure 4-Figure Supplement 1-Source Data 5/Hsc70-4_Vas_Myc_label_2023-06-30.tiff]

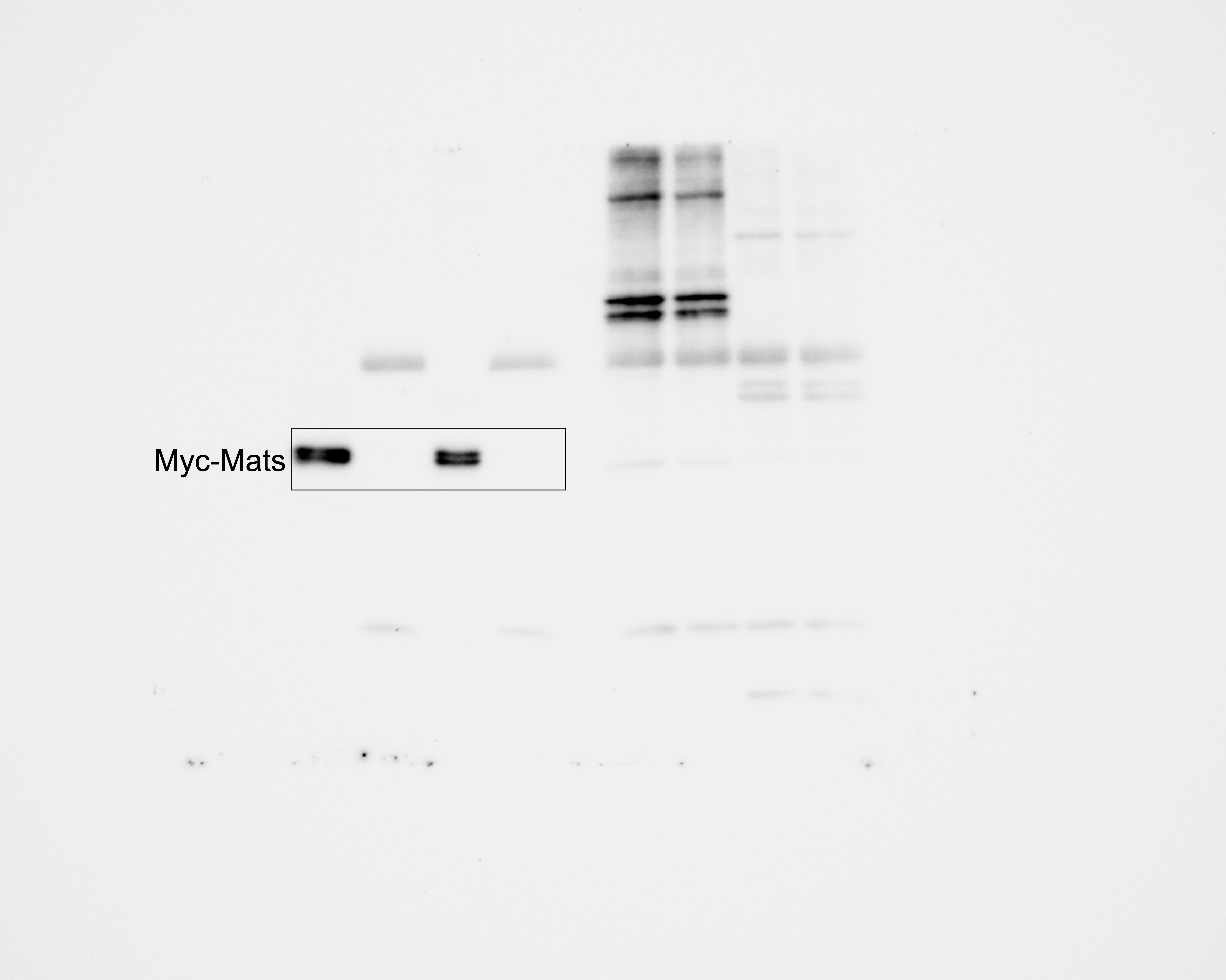

Supplement: Figure 4—figure supplement 1—source data 6. [file elife-101967-fig4-figsupp1-data6.zip › Figure 4-Figure Supplement 1-Source Data 6/Mats_Vas_Myc_label_2023-06-06.tiff]

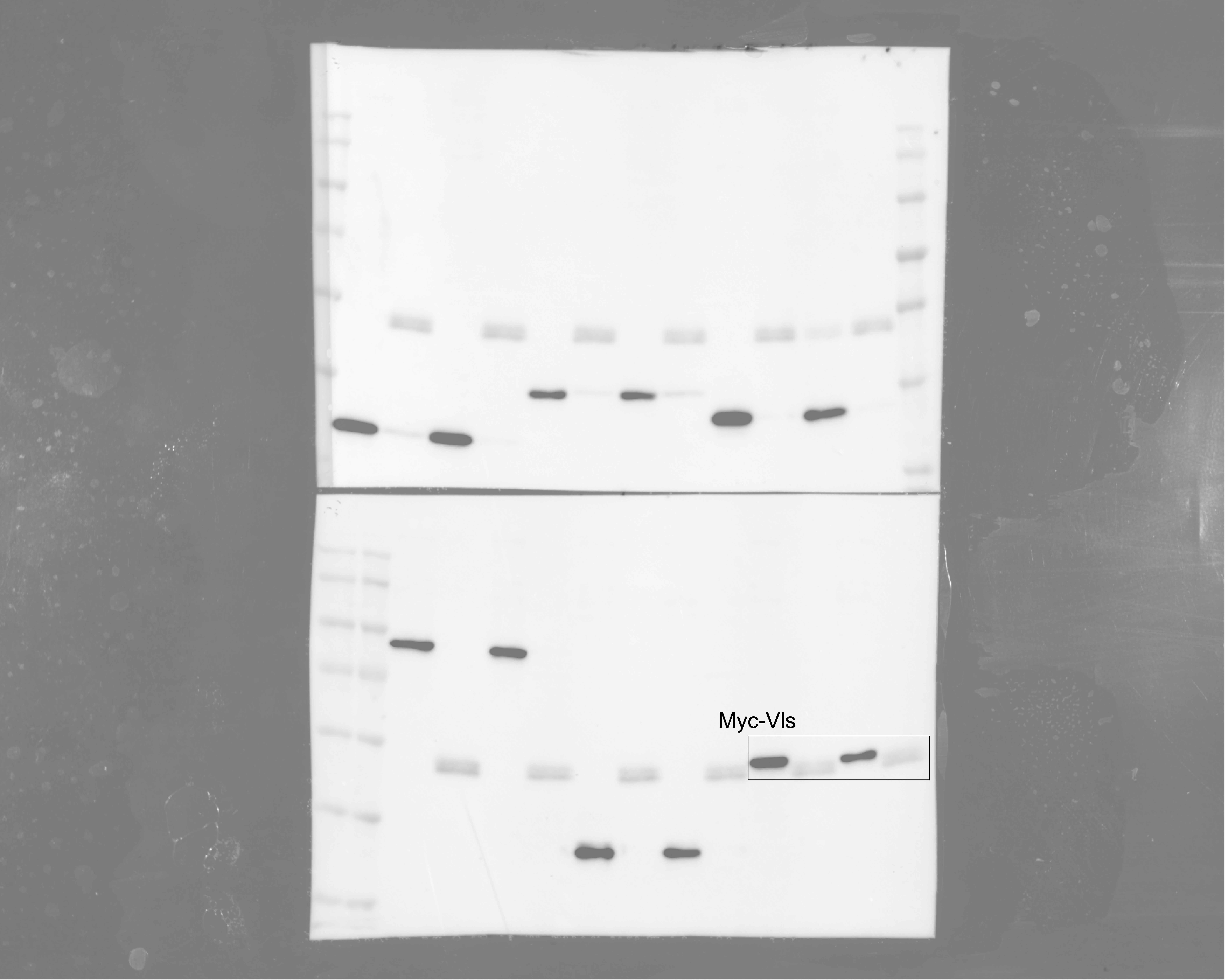

Supplement: Figure 4—figure supplement 1—source data 6. [file elife-101967-fig4-figsupp1-data6.zip › Figure 4-Figure Supplement 1-Source Data 6/Vls_Vas_Myc_label_2023-06-30.tiff]

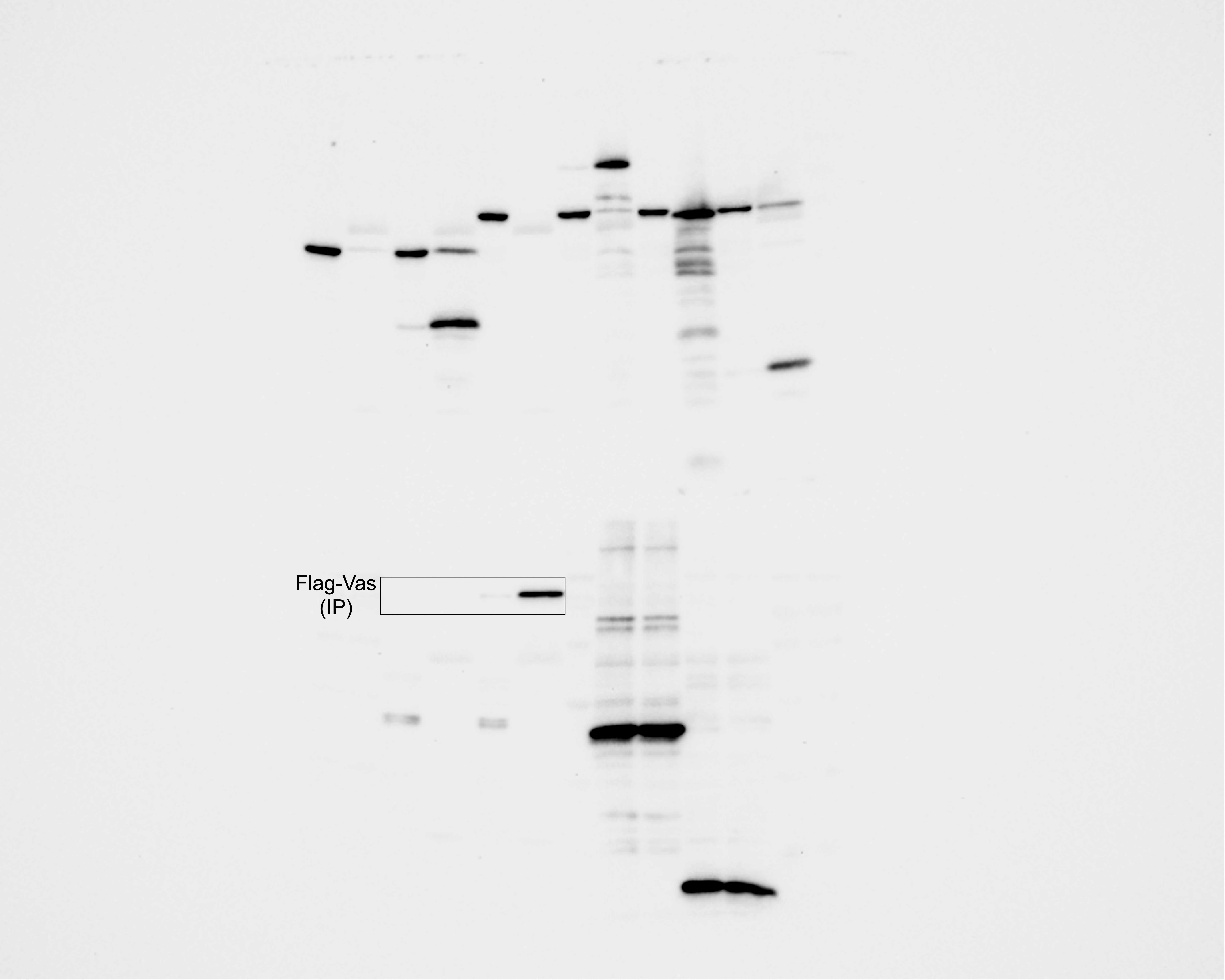

Supplement: Figure 4—figure supplement 1—source data 6. [file elife-101967-fig4-figsupp1-data6.zip › Figure 4-Figure Supplement 1-Source Data 6/Mats_Vas_FLAG_label_2023-06-06.tiff]

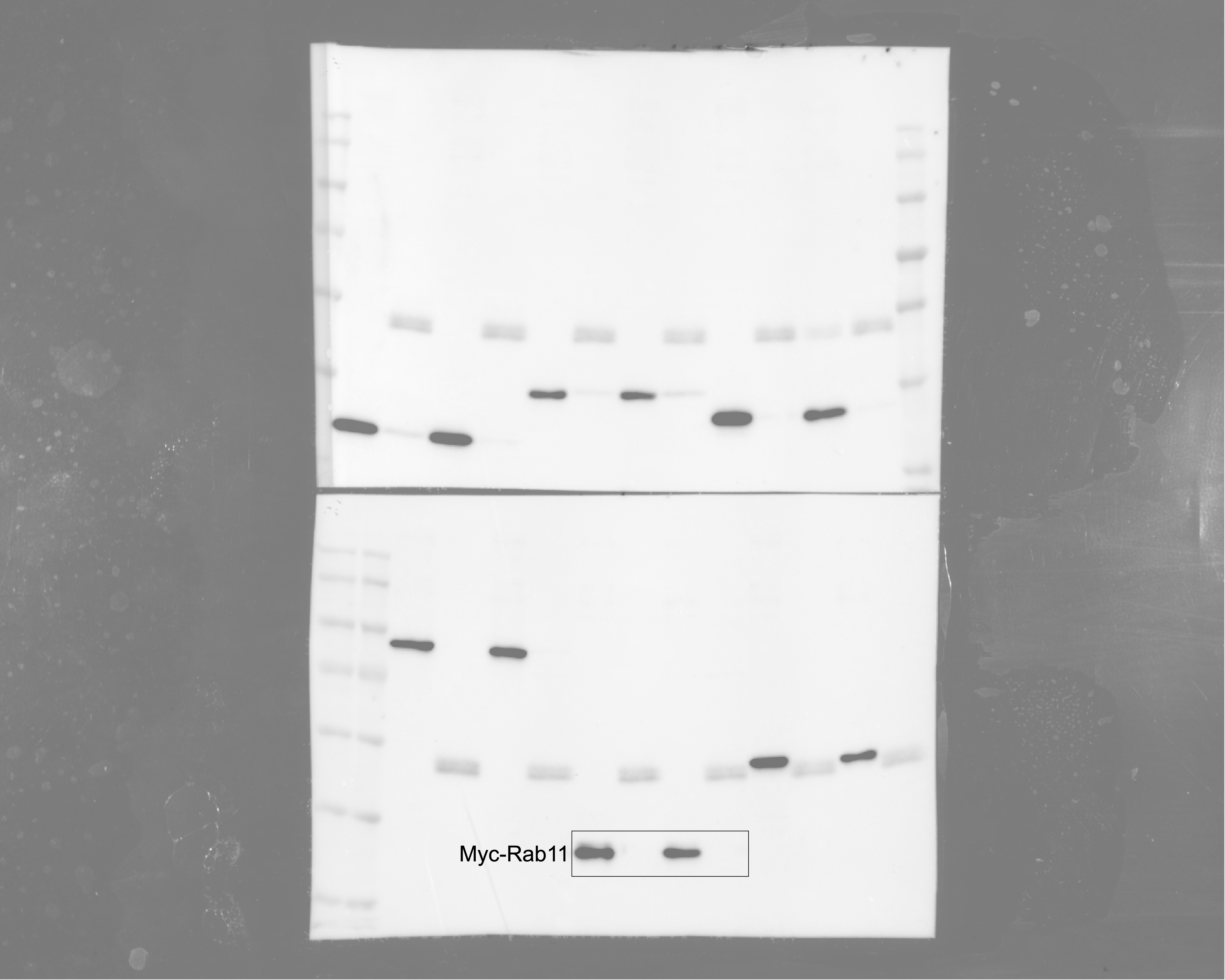

Supplement: Figure 4—figure supplement 1—source data 6. [file elife-101967-fig4-figsupp1-data6.zip › Figure 4-Figure Supplement 1-Source Data 6/Rab11_Vas_Myc_label_2023-06-30.tiff]

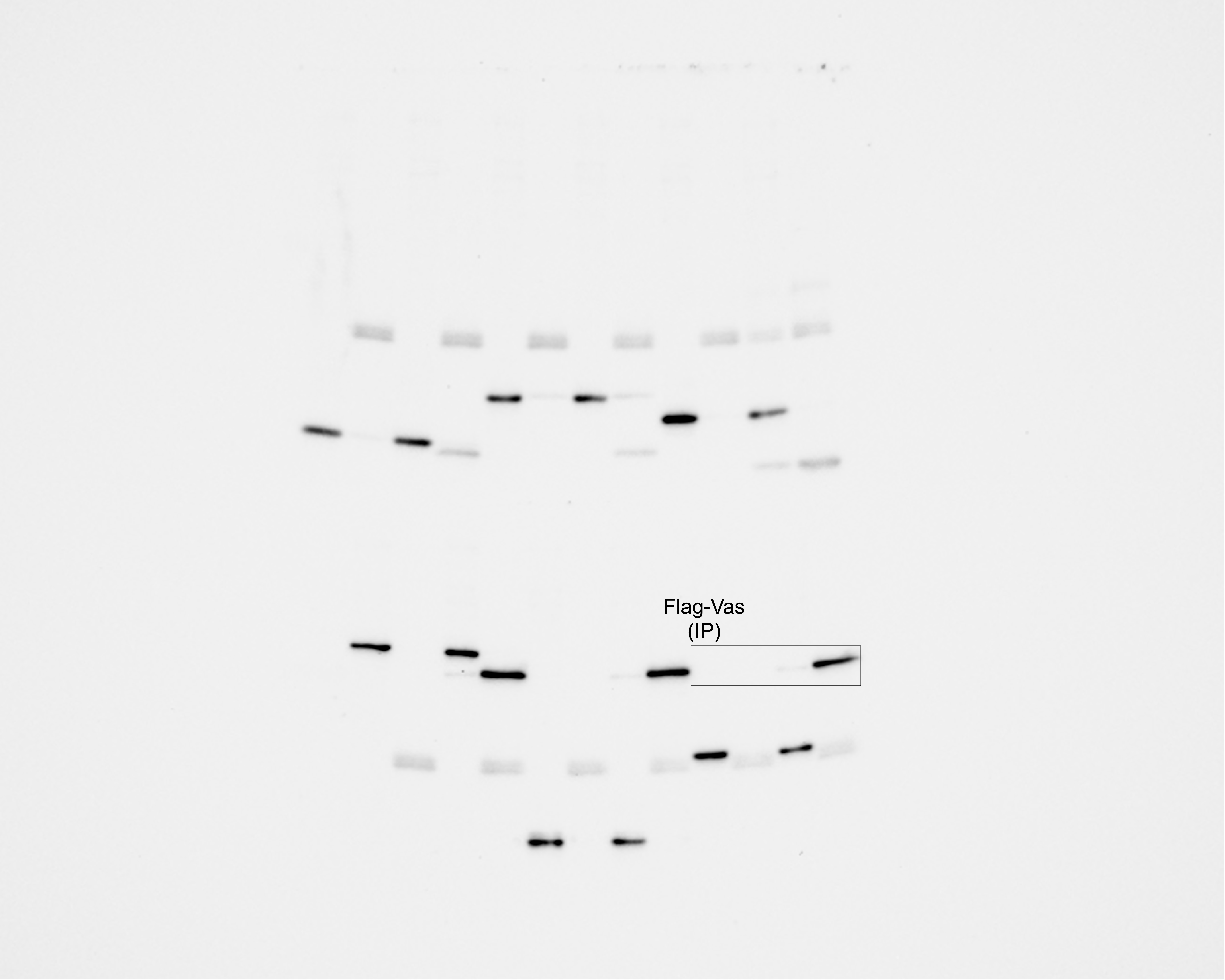

Supplement: Figure 4—figure supplement 1—source data 6. [file elife-101967-fig4-figsupp1-data6.zip › Figure 4-Figure Supplement 1-Source Data 6/Vls_Vas_Flag_label_2023-06-30.tiff]

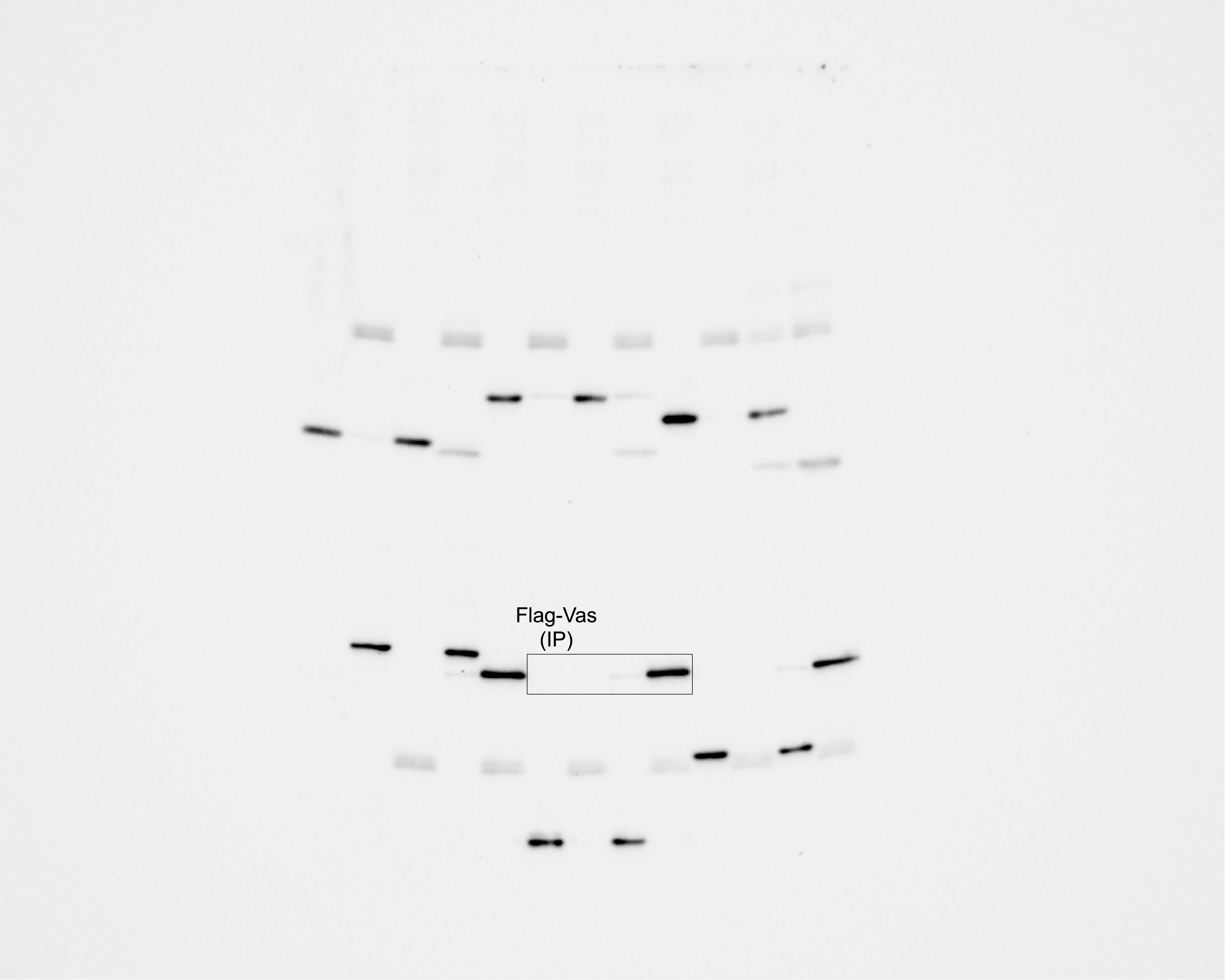

Supplement: Figure 4—figure supplement 1—source data 6. [file elife-101967-fig4-figsupp1-data6.zip › Figure 4-Figure Supplement 1-Source Data 6/Rab11_Vas_Flag_label_2023-06-30.tiff]

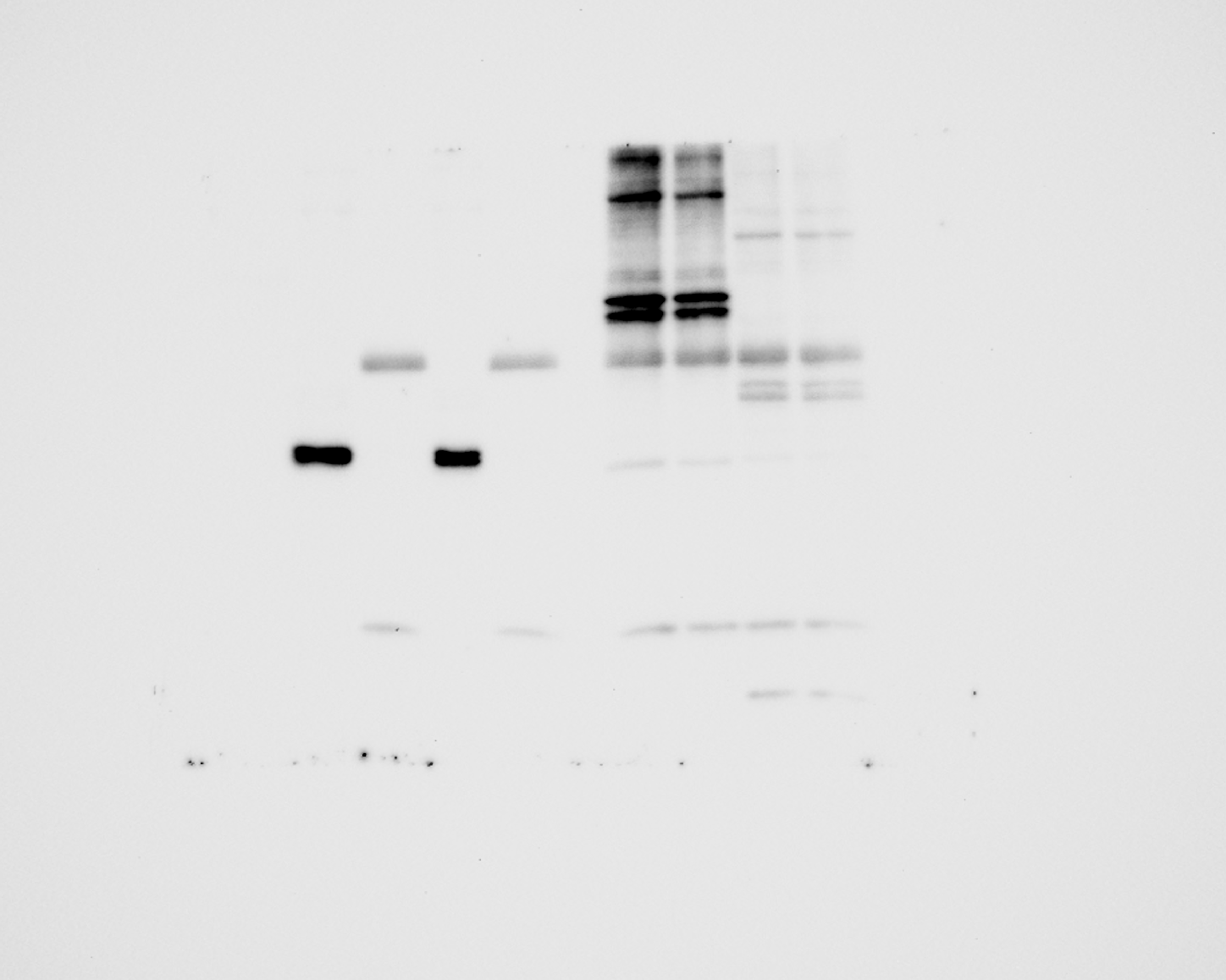

Supplement: Figure 4—figure supplement 1—source data 7. [file elife-101967-fig4-figsupp1-data7.zip › Figure 4-Figure Supplement 1-Source Data 7/Mats_Vas_Myc_original_2023-06-06.tif]

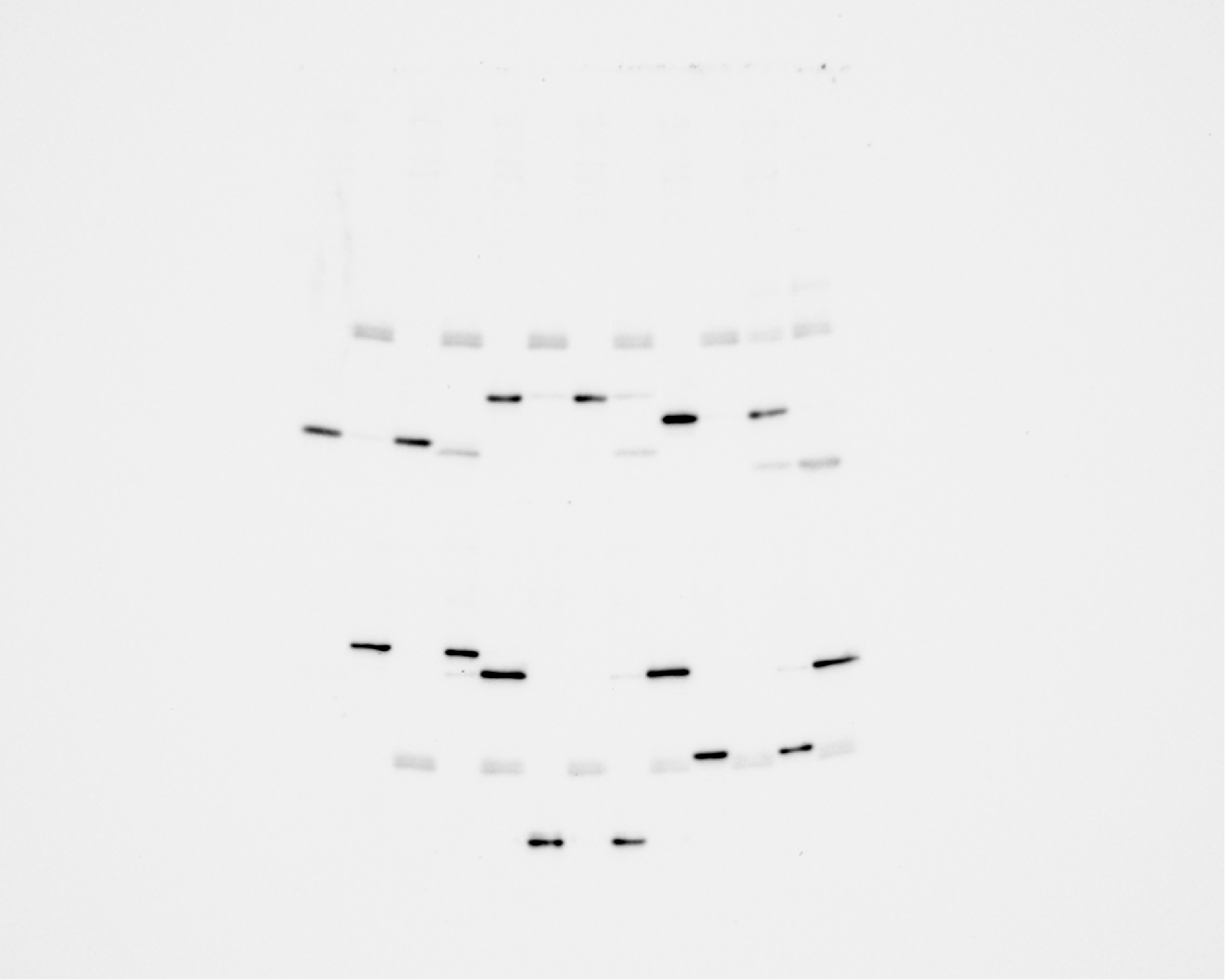

Supplement: Figure 4—figure supplement 1—source data 7. [file elife-101967-fig4-figsupp1-data7.zip › Figure 4-Figure Supplement 1-Source Data 7/Vls_Vas_Flag_original_2023-06-30.tif]

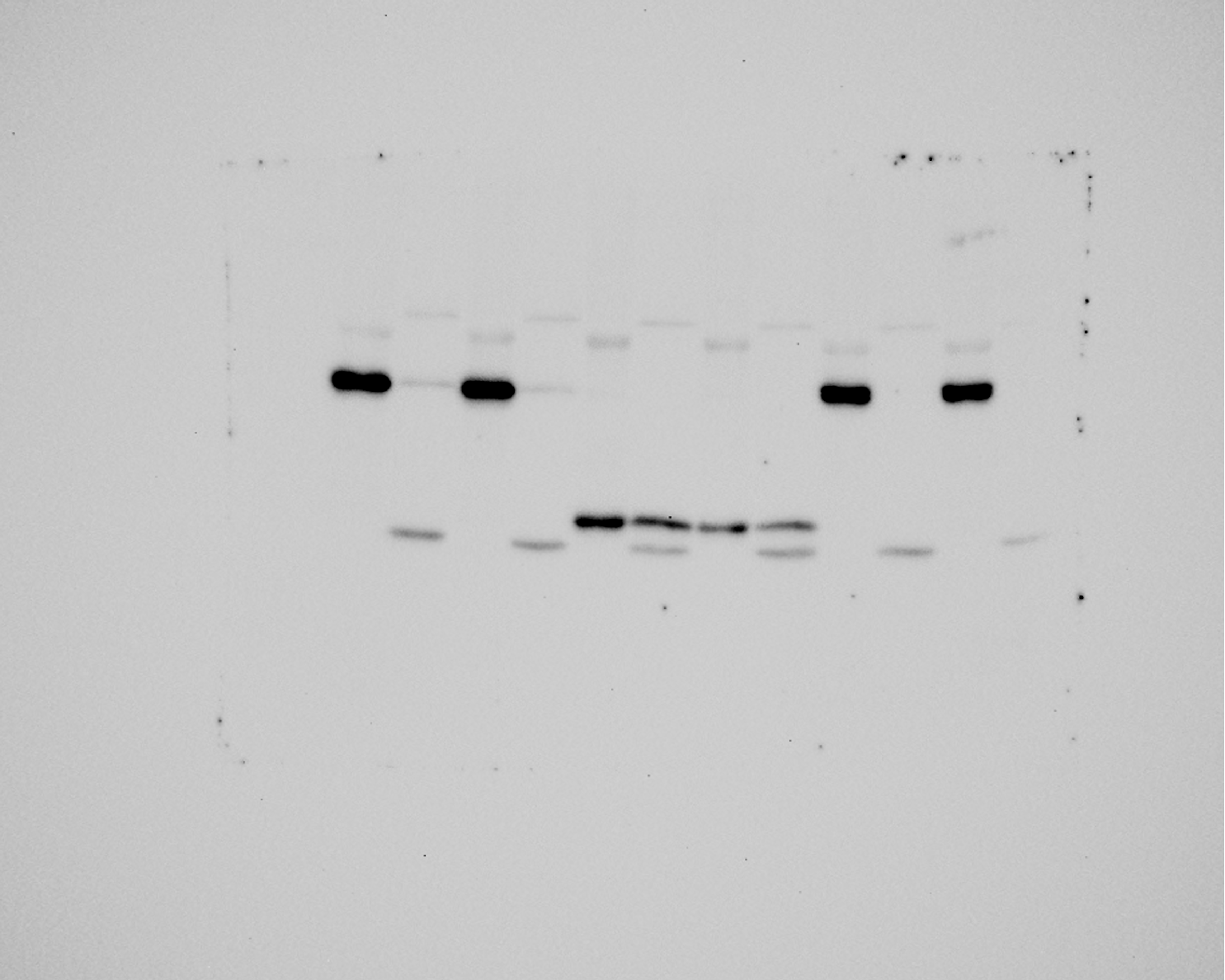

Supplement: Figure 4—figure supplement 1—source data 7. [file elife-101967-fig4-figsupp1-data7.zip › Figure 4-Figure Supplement 1-Source Data 7/Baf_Vas_Myc_original_2023-06-16.tif]

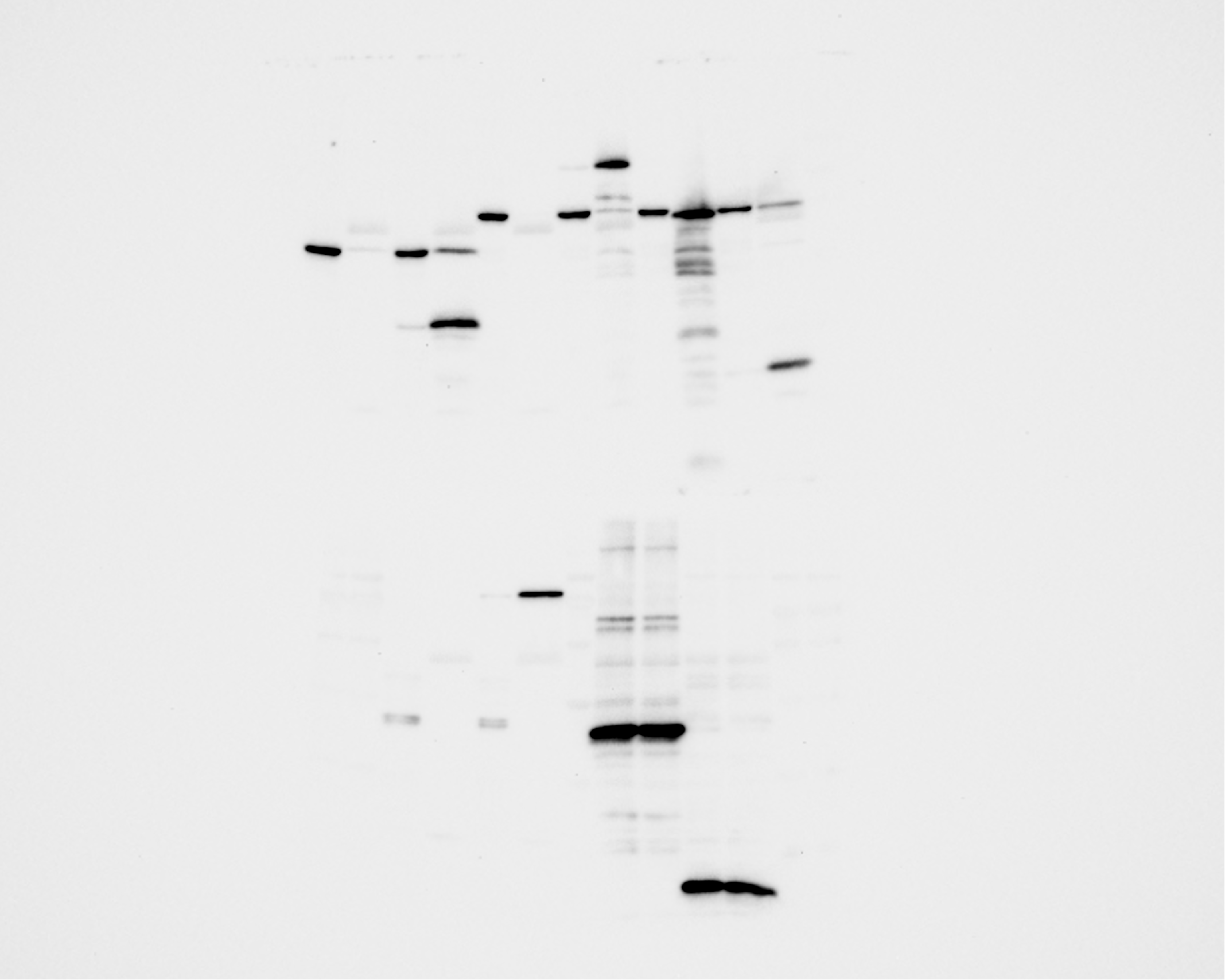

Supplement: Figure 4—figure supplement 1—source data 7. [file elife-101967-fig4-figsupp1-data7.zip › Figure 4-Figure Supplement 1-Source Data 7/Mats_Vas_FLAG_original_2023-06-06.tif]

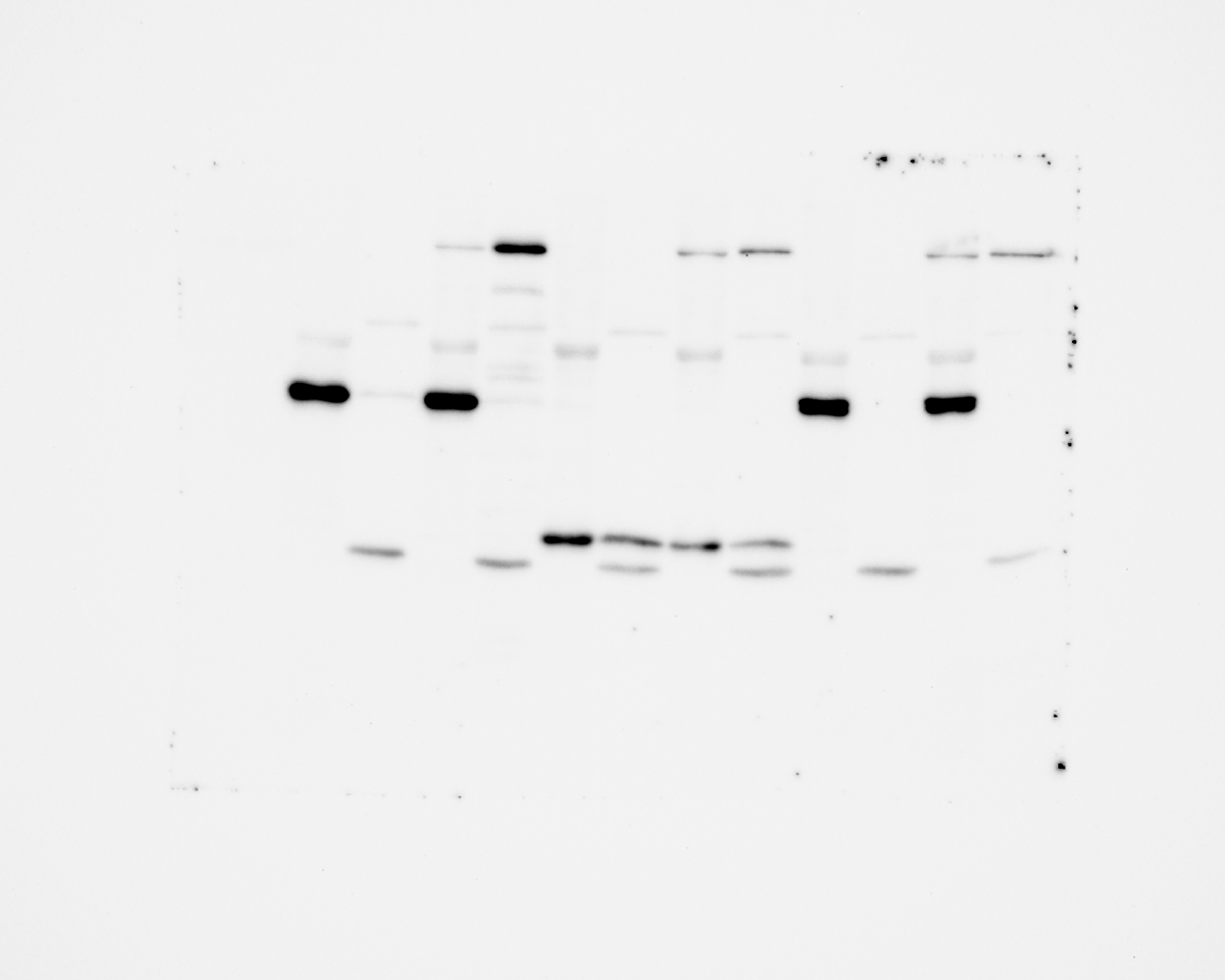

Supplement: Figure 4—figure supplement 1—source data 7. [file elife-101967-fig4-figsupp1-data7.zip › Figure 4-Figure Supplement 1-Source Data 7/Baf_Vas_Flag_original_2023-06-16.tif]

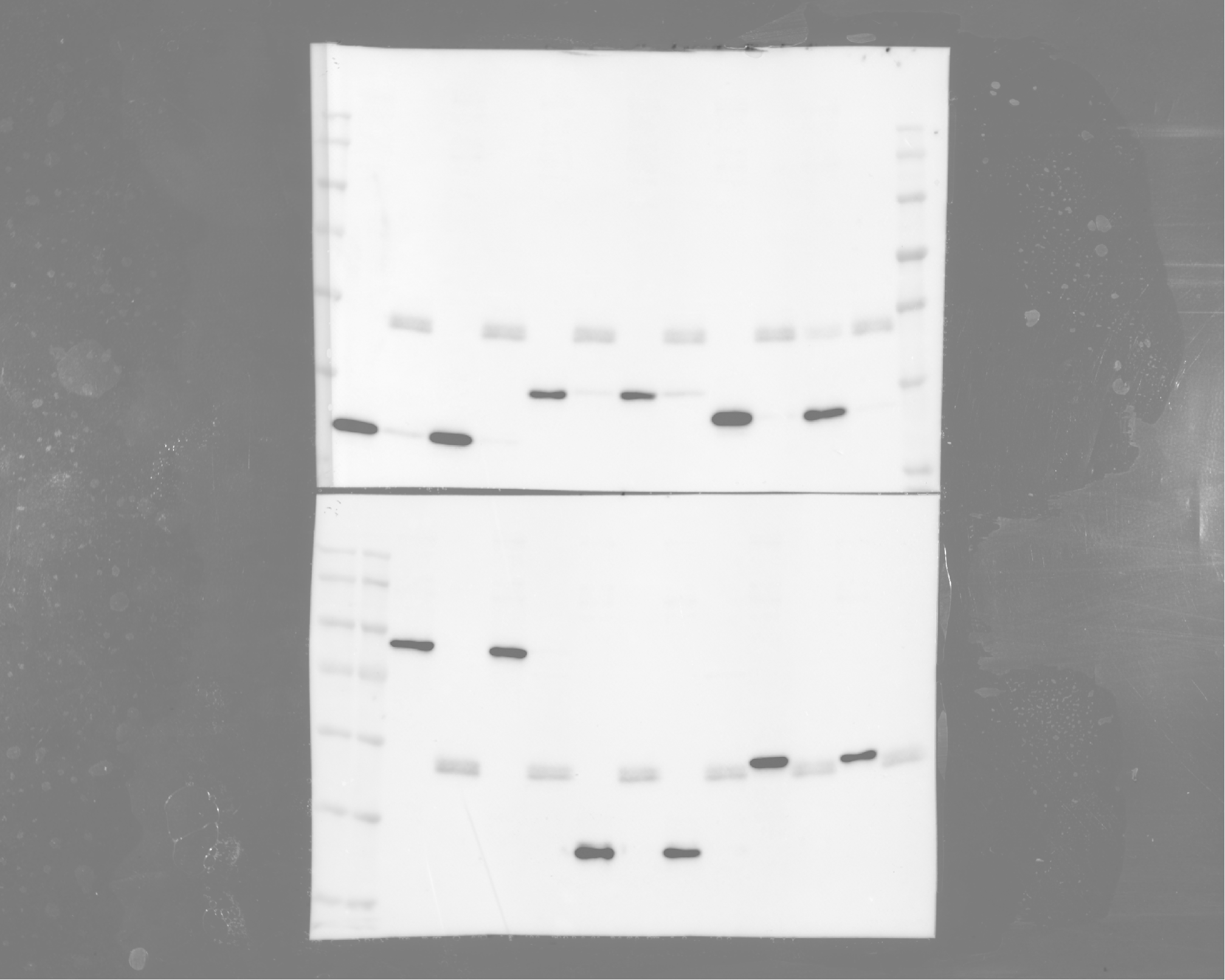

Supplement: Figure 4—figure supplement 1—source data 7. [file elife-101967-fig4-figsupp1-data7.zip › Figure 4-Figure Supplement 1-Source Data 7/Vls_Vas_Myc_original_2023-06-30.tif]

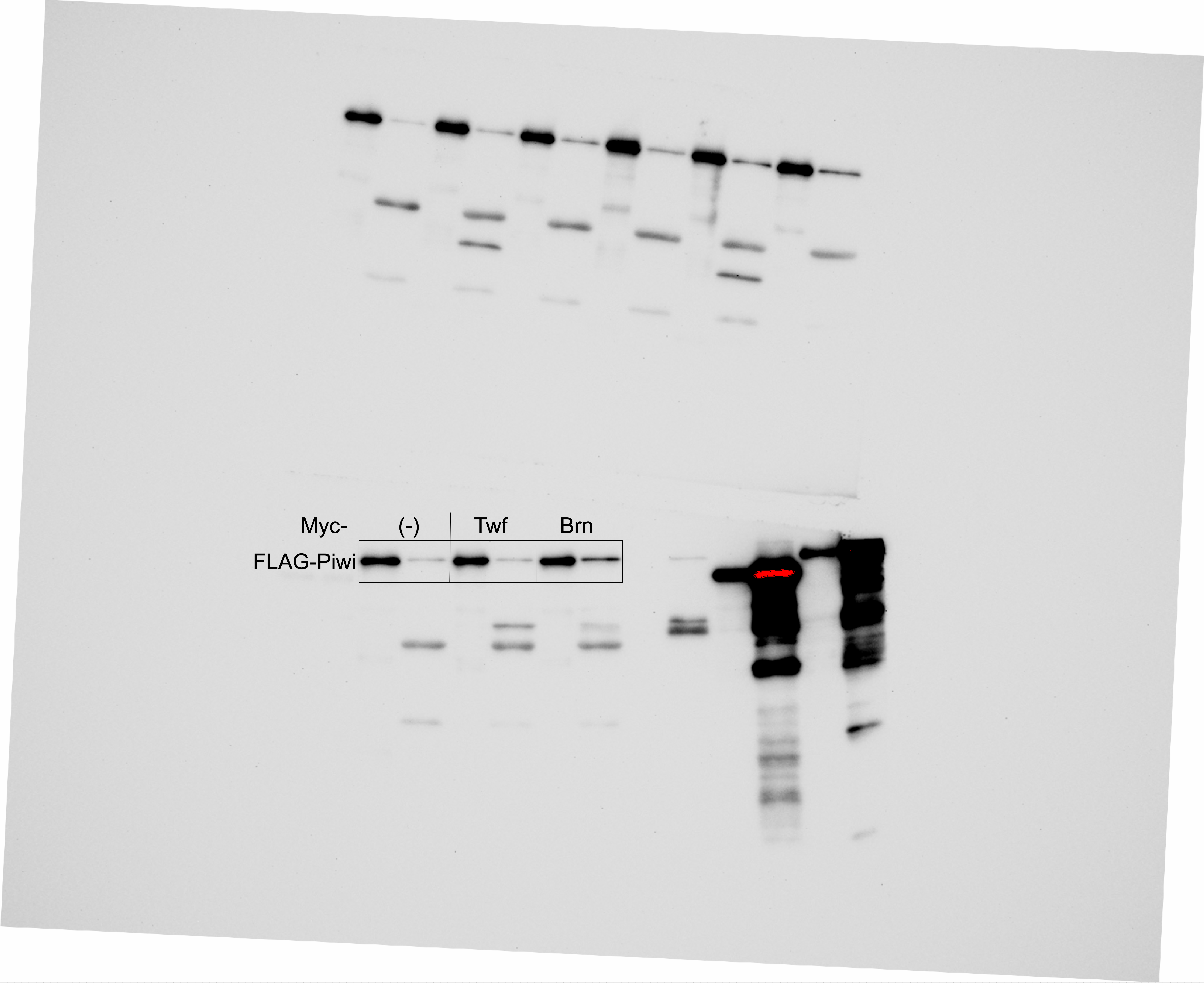

Supplement: Figure 5—source data 2. [file elife-101967-fig5-data2.zip › Figure 5-Source Data 2/Fig5C_rep3_Flag_label_2023-11-03.tiff]

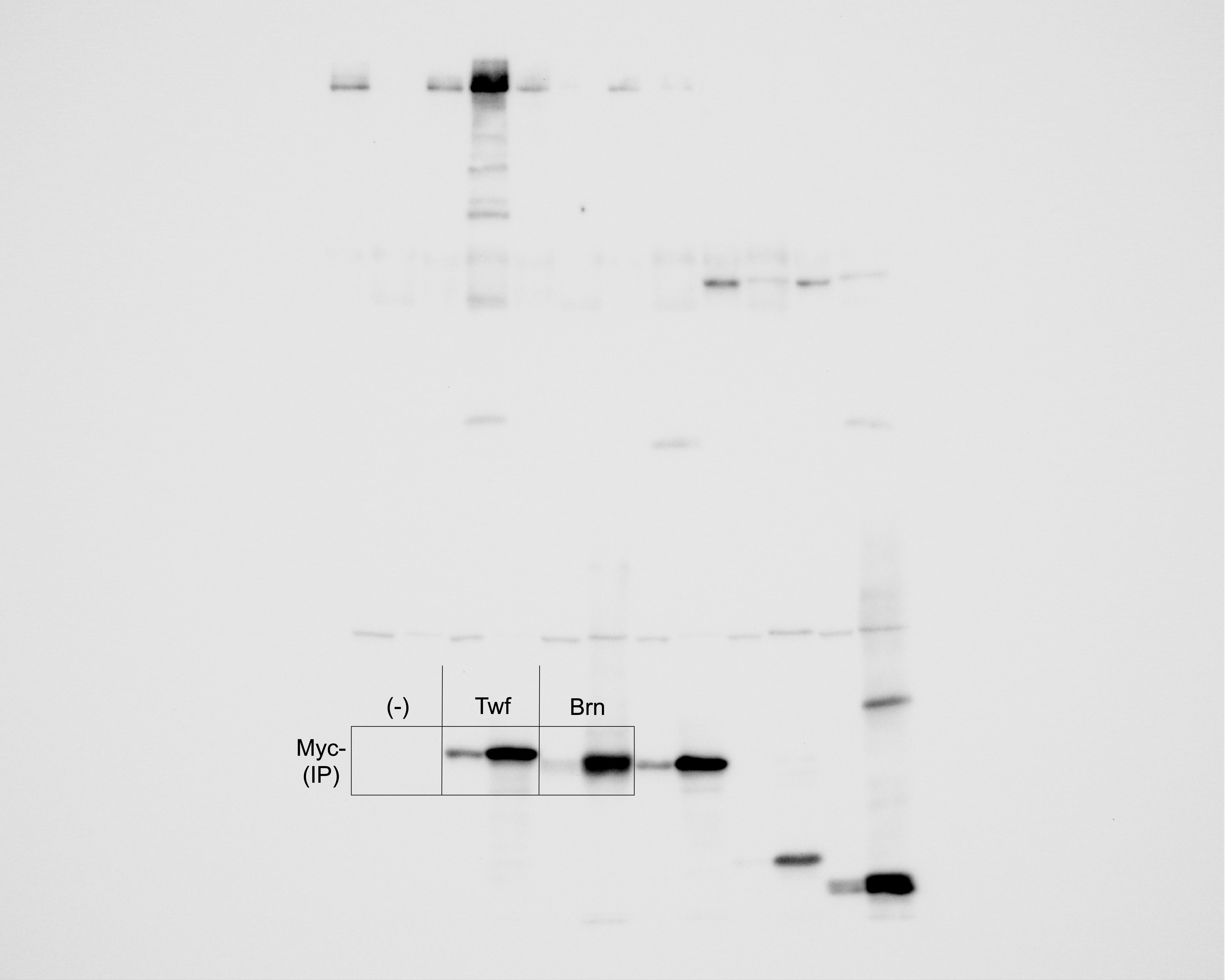

Supplement: Figure 5—source data 2. [file elife-101967-fig5-data2.zip › Figure 5-Source Data 2/Fig5C_rep1_Myc_label_2023-09-27.tiff]

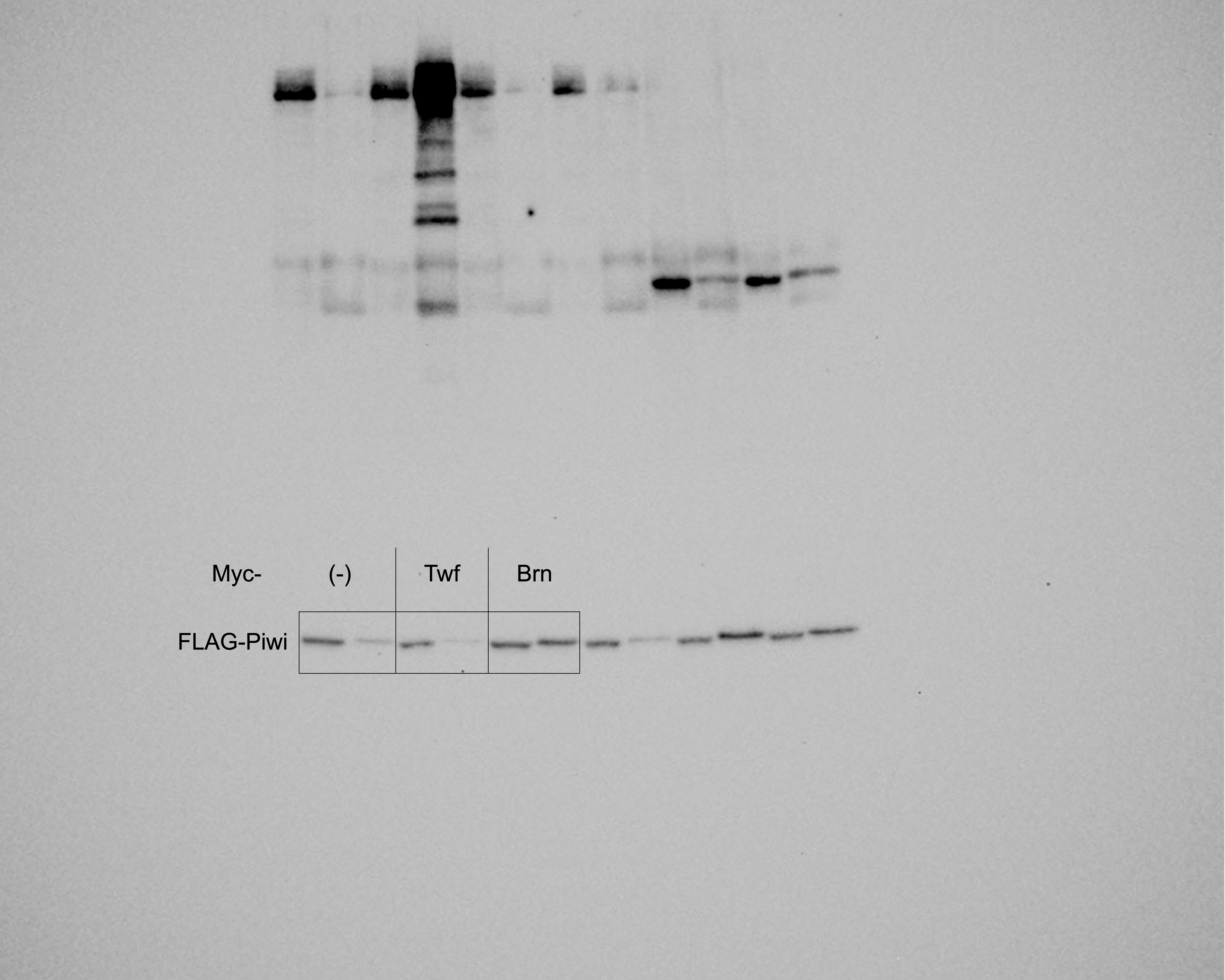

Supplement: Figure 5—source data 2. [file elife-101967-fig5-data2.zip › Figure 5-Source Data 2/Fig5C_rep1_Flag_label_2023-09-27.tiff]

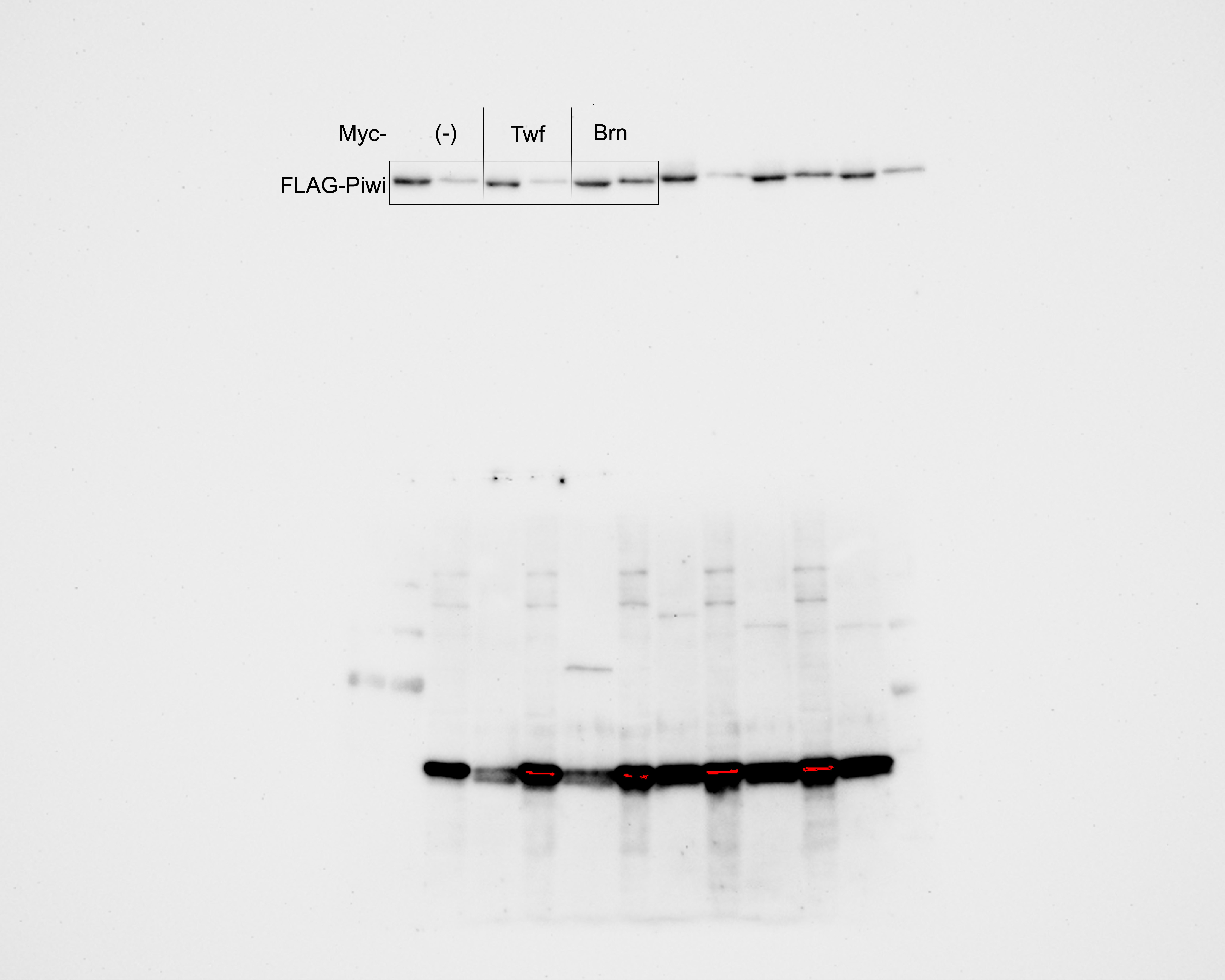

Supplement: Figure 5—source data 2. [file elife-101967-fig5-data2.zip › Figure 5-Source Data 2/Fig5C_rep2_Flag_label_2023-10-20.tiff]

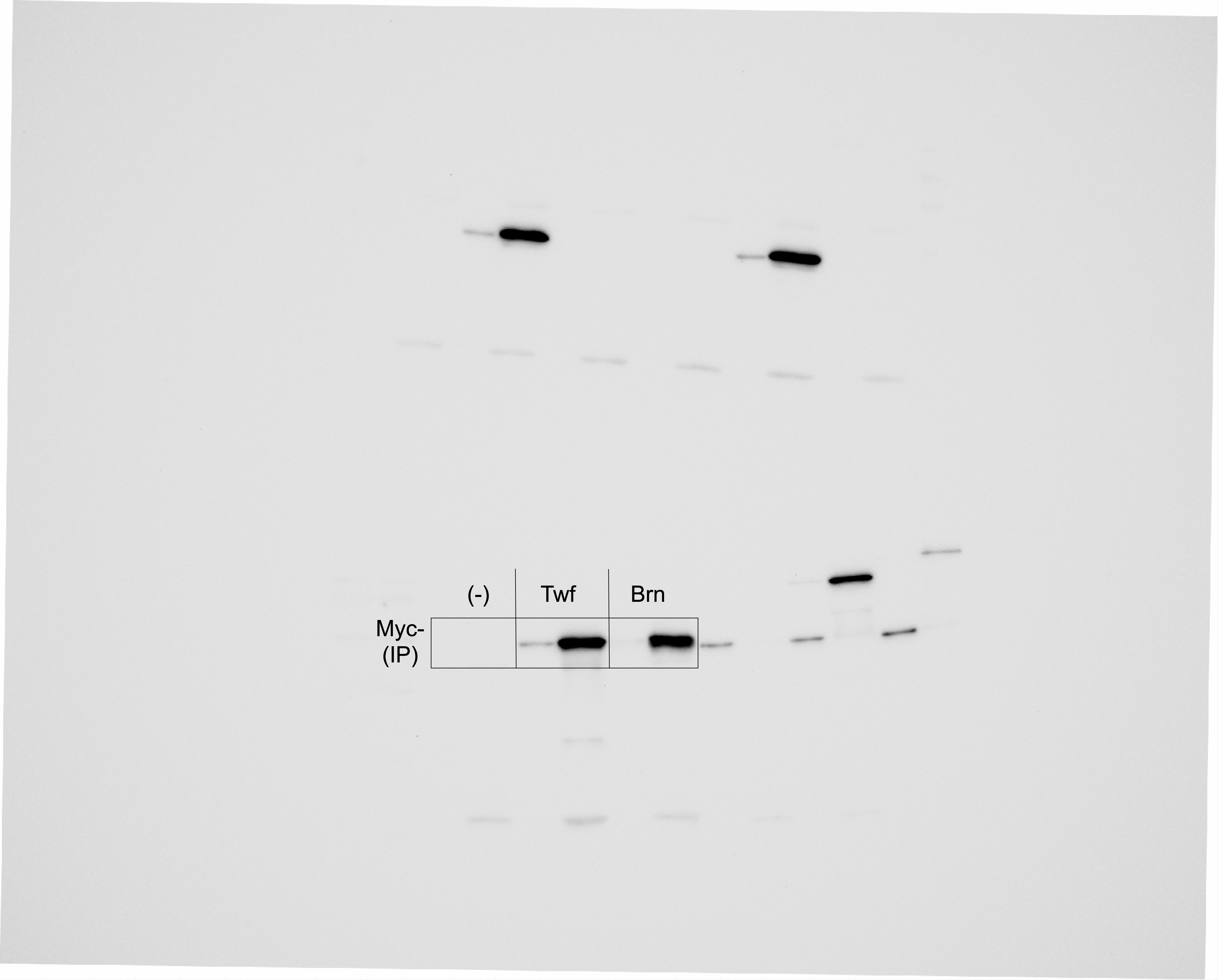

Supplement: Figure 5—source data 2. [file elife-101967-fig5-data2.zip › Figure 5-Source Data 2/Fig5C_rep3_Myc_label_2023-11-03.tiff]

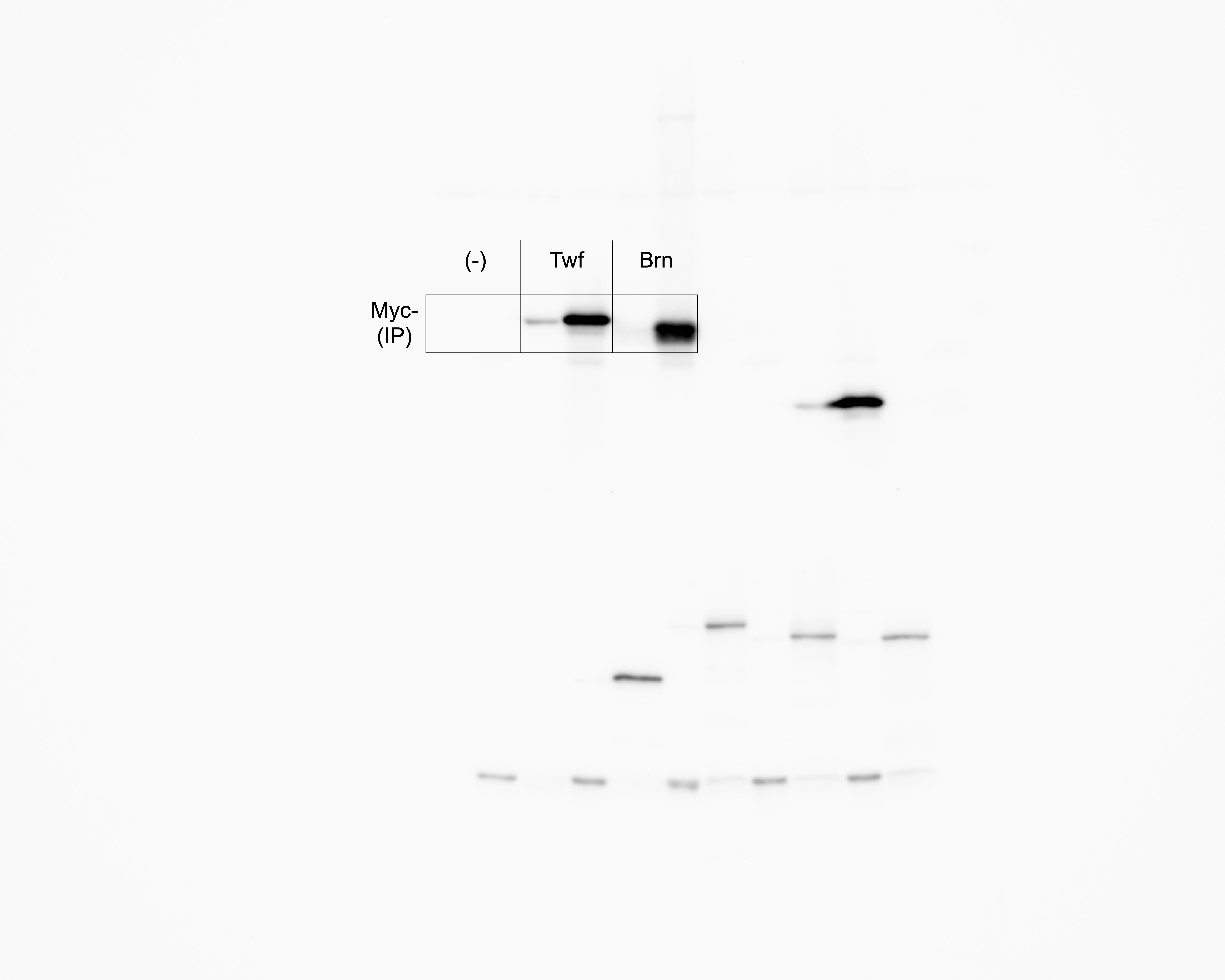

Supplement: Figure 5—source data 2. [file elife-101967-fig5-data2.zip › Figure 5-Source Data 2/Fig5C_rep2_Myc_label_2023-10-20.tiff]

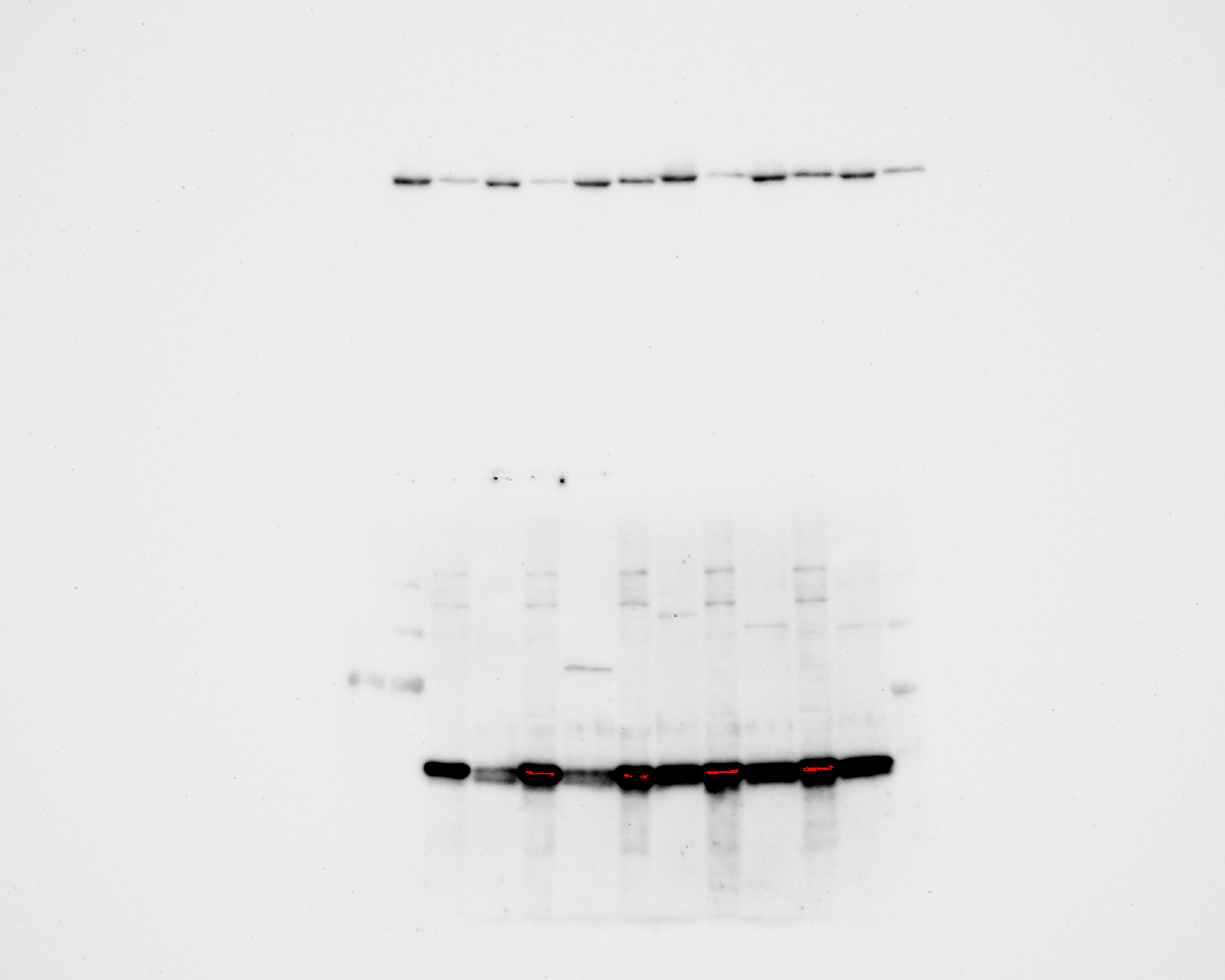

Supplement: Figure 5—source data 3. [file elife-101967-fig5-data3.zip › Figure 5-Source Data 3/Fig5C_rep2_Flag_original_2023-10-20.tif]

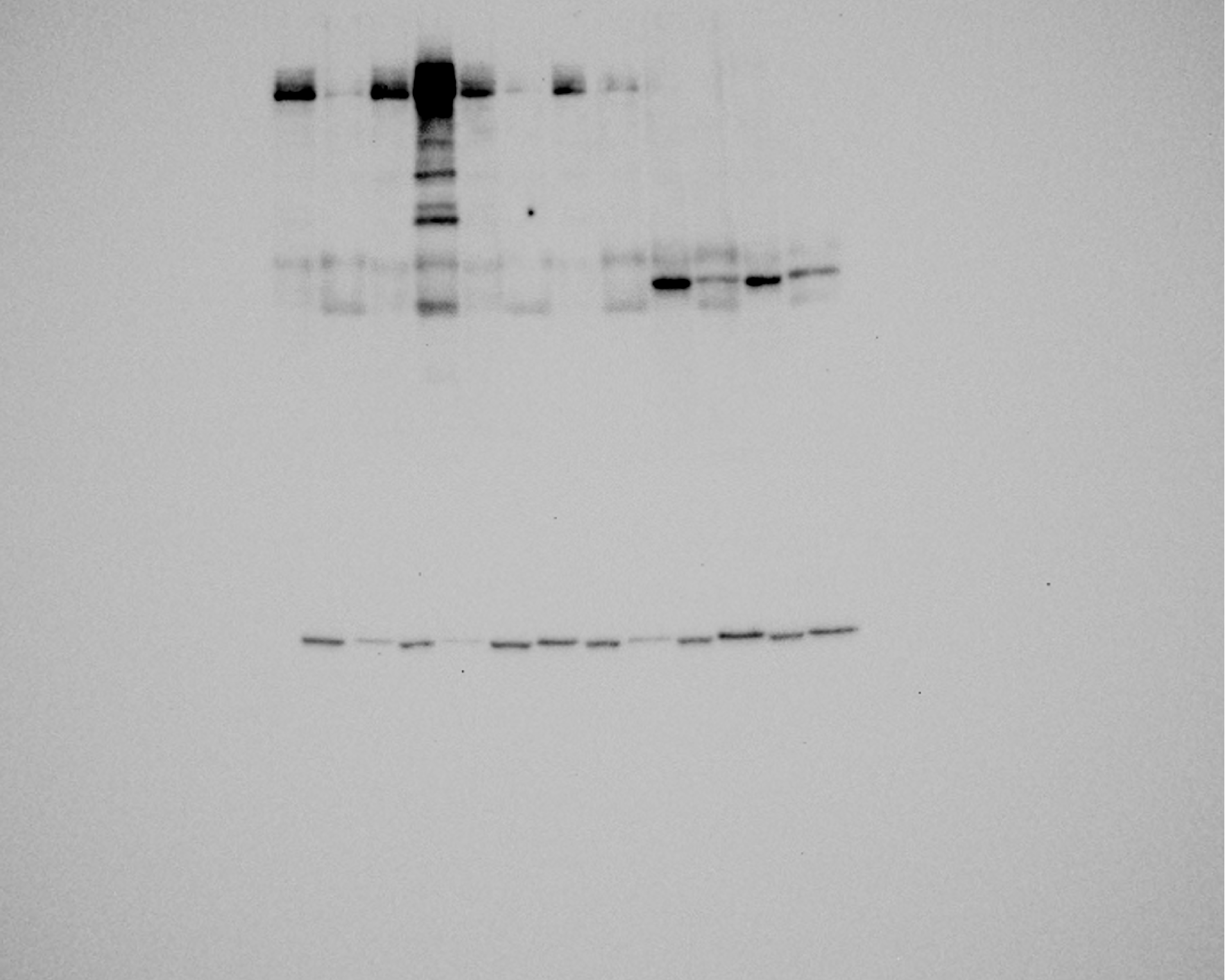

Supplement: Figure 5—source data 3. [file elife-101967-fig5-data3.zip › Figure 5-Source Data 3/Fig5C_rep1_Flag_original_2023-09-27.tif]

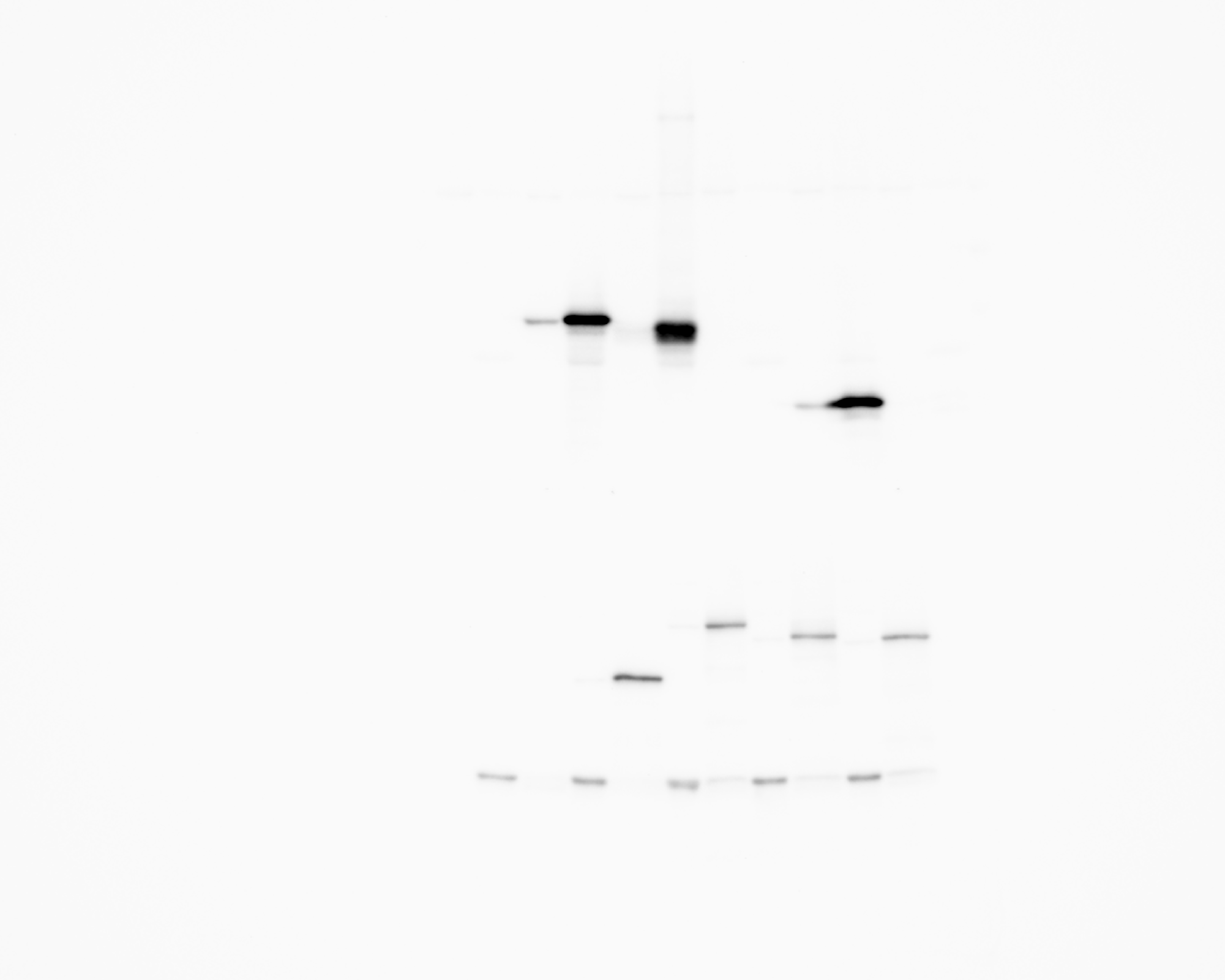

Supplement: Figure 5—source data 3. [file elife-101967-fig5-data3.zip › Figure 5-Source Data 3/Fig5C_rep2_Myc_original_2023-10-20.tif]

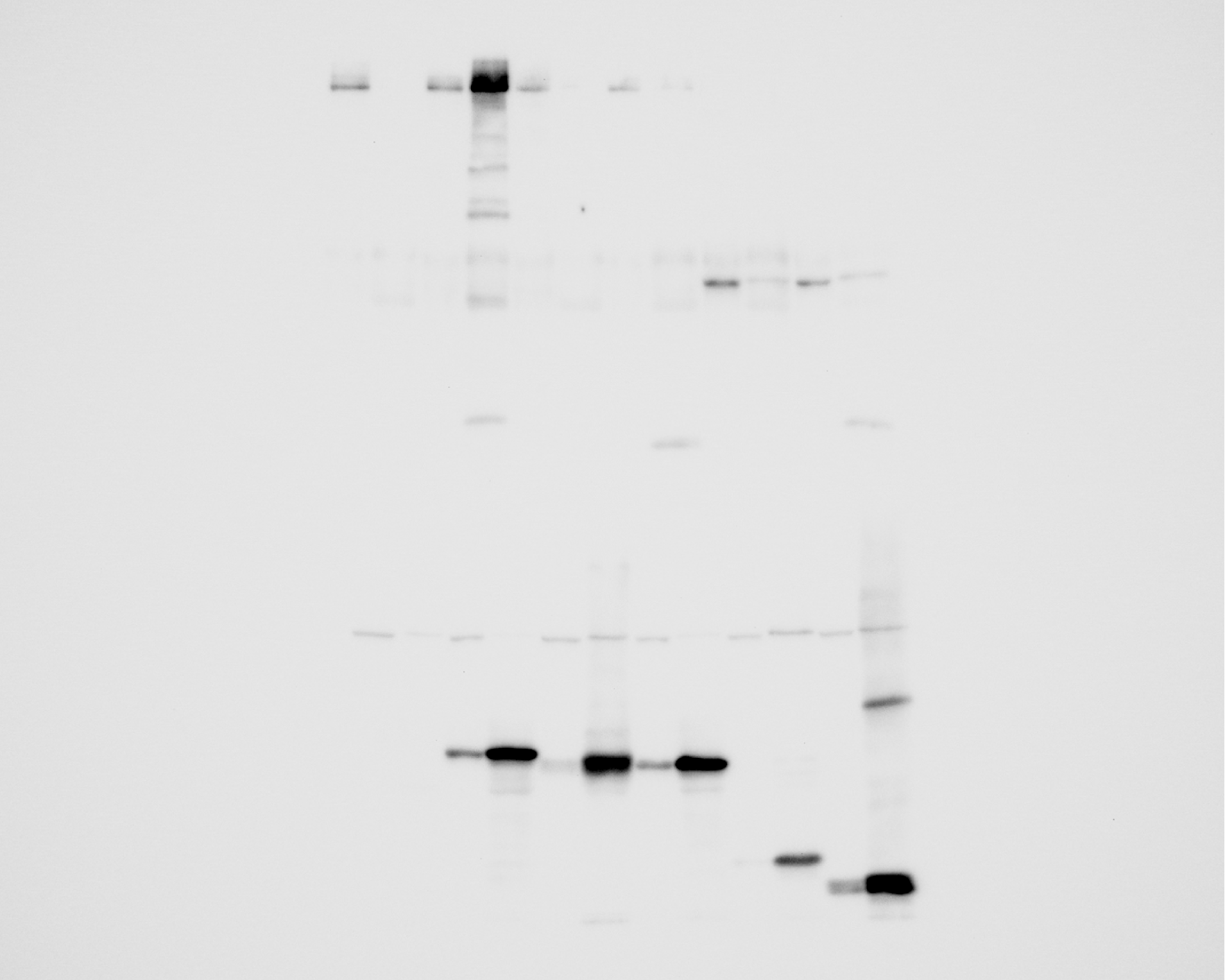

Supplement: Figure 5—source data 3. [file elife-101967-fig5-data3.zip › Figure 5-Source Data 3/Fig5C_rep1_Myc_original_2023-09-27.tif]

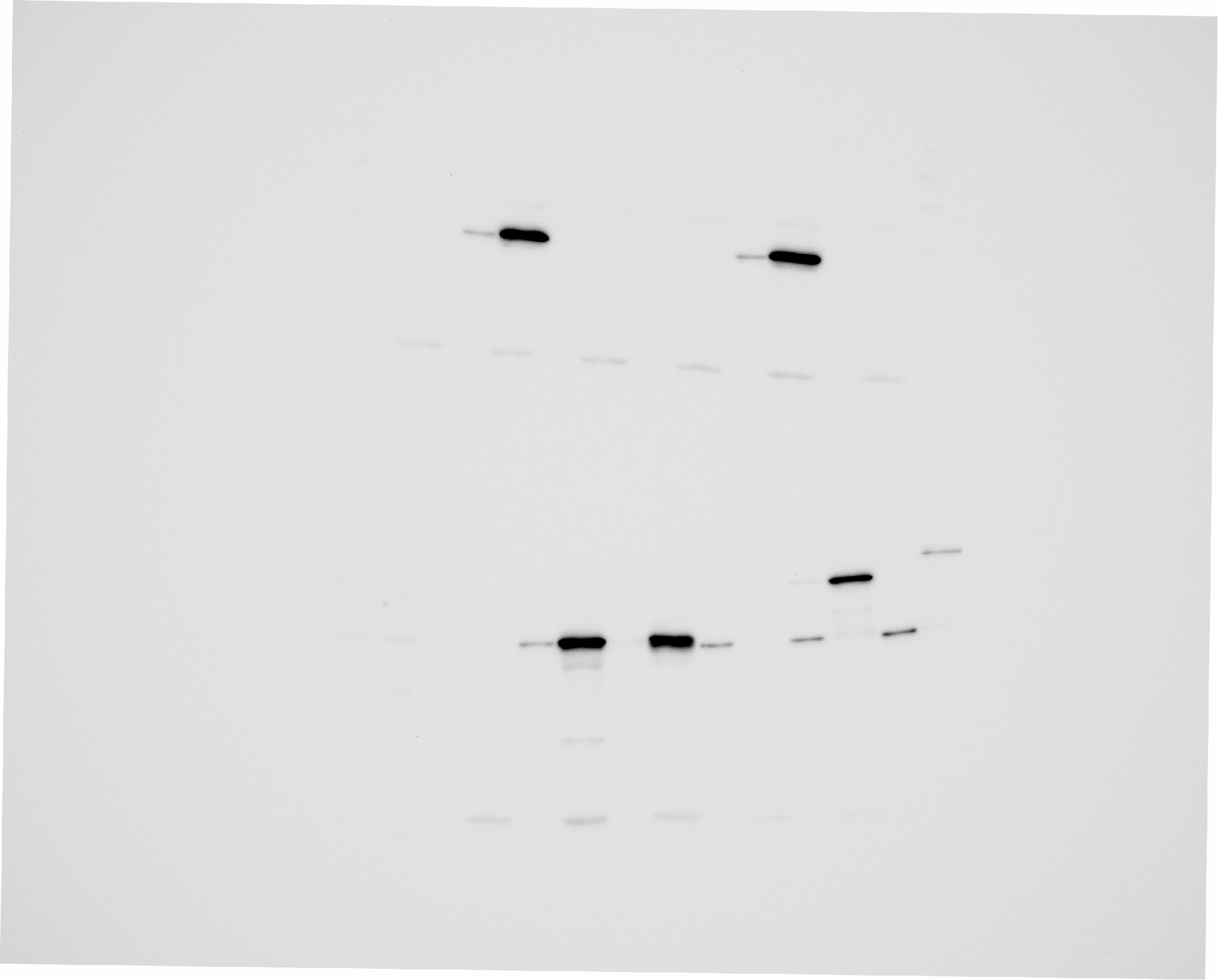

Supplement: Figure 5—source data 3. [file elife-101967-fig5-data3.zip › Figure 5-Source Data 3/Fig5C_rep3_Myc_original_2023-11-03.tif]

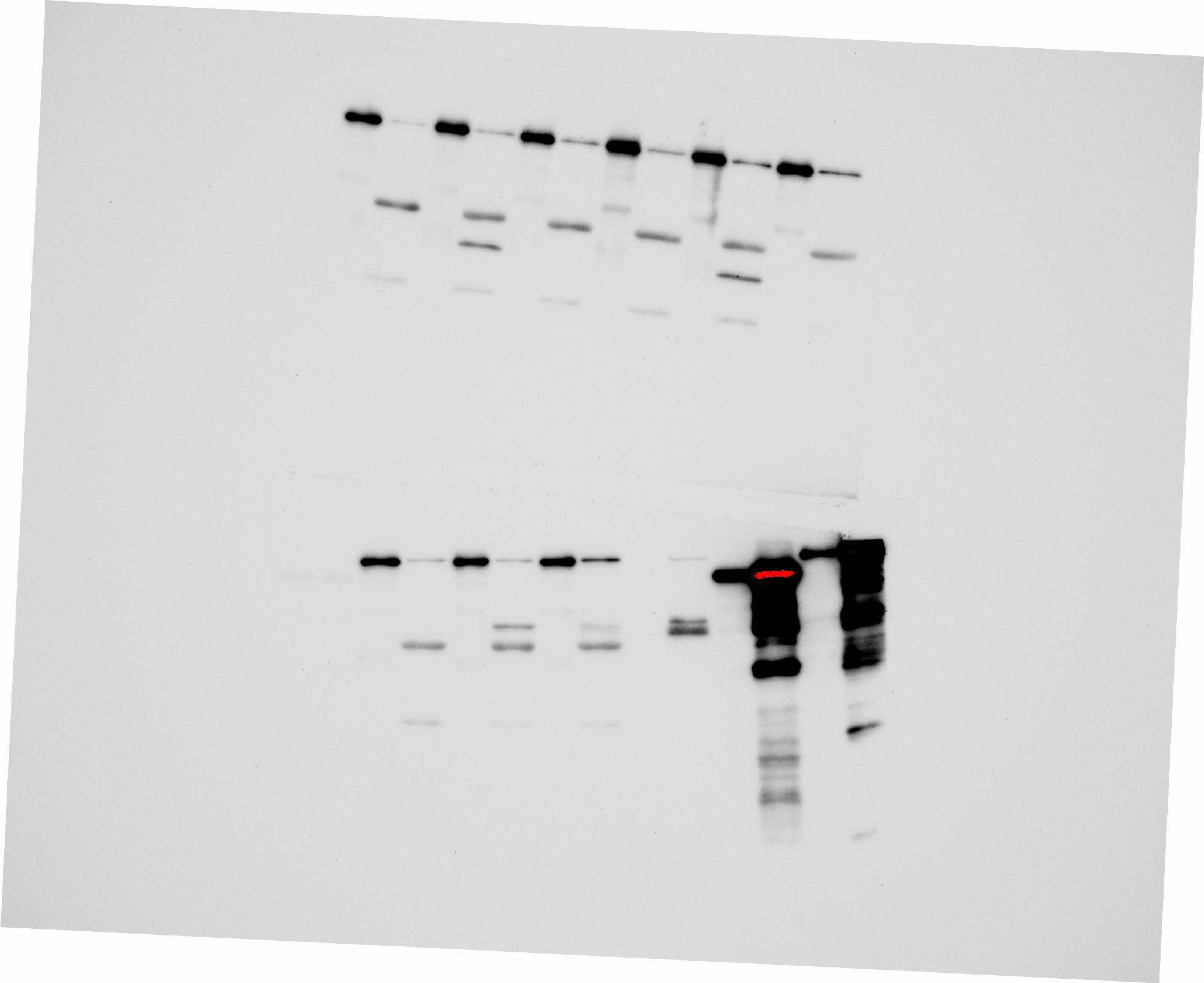

Supplement: Figure 5—source data 3. [file elife-101967-fig5-data3.zip › Figure 5-Source Data 3/Fig5C_rep3_Flag_original_2023-11-03.tif]
